# Ancient genomes reveal insights into ritual life at Chichén Itzá

**DOI:** 10.1038/s41586-024-07509-7

**Published:** 2024-06-12

**Authors:** Rodrigo Barquera, Oana Del Castillo-Chávez, Kathrin Nägele, Patxi Pérez-Ramallo, Diana Iraíz Hernández-Zaragoza, András Szolek, Adam Benjamin Rohrlach, Pablo Librado, Ainash Childebayeva, Raffaela Angelina Bianco, Bridget S. Penman, Victor Acuña-Alonzo, Mary Lucas, Julio César Lara-Riegos, María Ermila Moo-Mezeta, Julio César Torres-Romero, Patrick Roberts, Oliver Kohlbacher, Christina Warinner, Johannes Krause

**Affiliations:** 1 Department of Archaeogenetics, Max-Planck Institute for Evolutionary Anthropology (MPI-EVA), Leipzig, Germany; 2 Molecular Genetics Laboratory, Escuela Nacional de Antropología e Historia (ENAH), Mexico City, Mexico; 3 Centro INAH Yucatán, Instituto Nacional de Antropología e Historia (INAH), Mérida, Yucatán, Mexico; 4 isoTROPIC Research Group, Max Planck Institute of Geoanthropology, Jena, Germany; 5 University of the Basque Country (EHU), San Sebastián-Donostia, Spain; 6 Department of Archaeology, Max Planck Institute of Geoanthropology, Jena, Germany; 7 Applied Bioinformatics, Dept. for Computer Science, University of Tübingen, Tübingen, Germany; 8 Department of Immunology, Interfaculty Institute for Cell Biology, University of Tübingen, Tübingen, Germany; 9 School of Computer and Mathematical Sciences, University of Adelaide, Adelaide, South Australia Australia; 10 Institut de Biologia Evolutiva (CSIC—Universitat Pompeu Fabra), Barcelona, Spain; 11 The Zeeman Institute and the School of Life Sciences, University of Warwick, Coventry, UK; 12 Chemistry Faculty, Universidad Autónoma de Yucatán (UADY), Mérida, Mexico; 13 Nursing Faculty, Universidad Autónoma de Yucatán (UADY), Mérida, Mexico; 14 Institute for Bioinformatics and Medical Informatics, University of Tübingen, Tübingen, Germany; 15 Quantitative Biology Center, University of Tübingen, Tübingen, Germany; 16 Translational Bioinformatics, University Hospital Tübingen, Tübingen, Germany; 17 Department of Anthropology, Harvard University, Cambridge, MA USA; 18 Present Address: Department of Archaeology and Cultural History, University Museum, Norwegian University of Science and Technology (NTNU), Trondheim, Norway; 19 Present Address: Department of Anthropology, University of Texas at Austin, Austin, TX USA

**Keywords:** Archaeology, Immunogenetics, Population genetics, Anthropology

## Abstract

The ancient city of Chichén Itzá in Yucatán, Mexico, was one of the largest and most influential Maya settlements during the Late and Terminal Classic periods (ad 600–1000) and it remains one of the most intensively studied archaeological sites in Mesoamerica^1–4^. However, many questions about the social and cultural use of its ceremonial spaces, as well as its population’s genetic ties to other Mesoamerican groups, remain unanswered^2^. Here we present genome-wide data obtained from 64 subadult individuals dating to around ad 500–900 that were found in a subterranean mass burial near the Sacred Cenote (sinkhole) in the ceremonial centre of Chichén Itzá. Genetic analyses showed that all analysed individuals were male and several individuals were closely related, including two pairs of monozygotic twins. Twins feature prominently in Mayan and broader Mesoamerican mythology, where they embody qualities of duality among deities and heroes^5^, but until now they had not been identified in ancient Mayan mortuary contexts. Genetic comparison to present-day people in the region shows genetic continuity with the ancient inhabitants of Chichén Itzá, except at certain genetic loci related to human immunity, including the human leukocyte antigen complex, suggesting signals of adaptation due to infectious diseases introduced to the region during the colonial period.

## Abstract (web summary)

We present genome-wide data from 64 subadults interred in Chichén Itzá around ad 500–900 that gives insight into burial rituals, and shows that their genomic legacy is still present and has adapted to immune challenges post-1492.

## Main

**Fig. 1:**
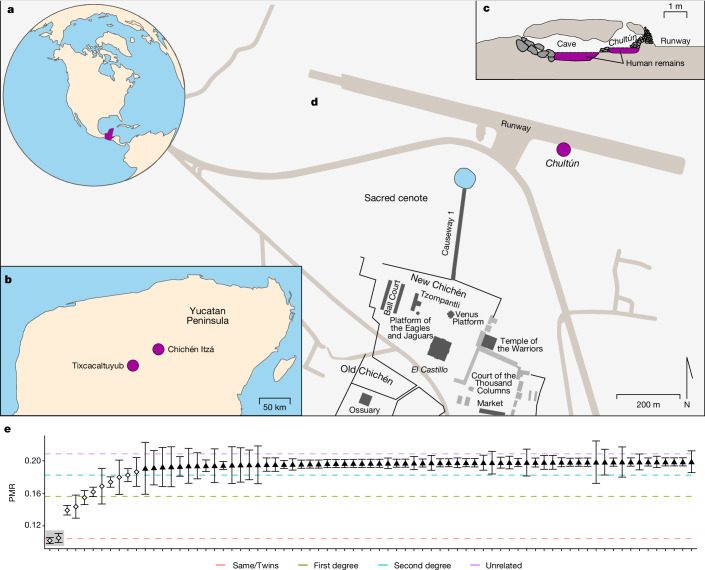
Geographical context for the groups analysed and biological relatedness in the *chultún*. **a**, Location of the Maya region in the Americas. **b**, Geographical locations of Chichén Itzá and Tixcacaltuyub in the Yucatan Peninsula. **c**, Stratigraphy for the *chultún* and the adjacent cave in which the burial was found (adapted from ref. ^4^). **d**, Location of the *chultún* within the archaeological site of Chichén Itzá and its relation to *El Castillo* (adapted from ref. ^10^). Modern roads are marked in light grey; the *chultún* abuts an airport runway. **e**, Genetic pairwise mismatch rate (PMR) for child pairs in the *chultún* identifies 11 close relative pairs (hollow diamonds), including two pairs of monozygotic twins (highlighted in grey). A low overall PMR for unrelated individuals (black triangles) confirms low genetic diversity in the population; only pairs with PMR < 0.20 are visualized in the plot. See Supplementary Fig. 2 for individual annotations.

The ancient Maya city of Chichén Itzá, centrally located in the northern part of the Yucatán Peninsula (Fig. 1a,b), ranks among the largest and most iconic archaeological sites in Mesoamerica but much about its origins and history remains poorly understood^1,2^. First rising to prominence during the Late Classic period (ad 600–800), Chichén Itzá became the dominant political centre of the northern Maya lowlands during the Terminal Classic (ad 800–1000), a period when most other Classic Maya sites in the southern and northern lowlands underwent a political collapse. Most of the inscribed calendar dates on carved monuments at Chichén Itzá fall between ad 850 and 875 and the northern ceremonial centre of the site, known as New Chichén, was largely constructed after ad 900 and includes the site’s largest structure, *El Castillo*, also known as the Temple of Kukulkán. A *sacbe* (limestone causeway) was constructed to connect New Chichén to the Sacred Cenote^6^, an enormous sinkhole containing abundant ritual offerings, including the remains of more than 200 ritually sacrificed individuals, mostly children^1,3,7^. Evidence of ritual killing is extensive throughout the site of Chichén Itzá and includes both the physical remains of sacrificed individuals as well as representations in monumental art^8^. Elite activity at Chichén Itzá declined during the eleventh century ad, with a last inscribed calendar date of ad 998 (refs. ^9,10^), but the site continued to be a prominent ritual and pilgrimage centre during the colonial period and beyond^11–13^.

In 1967, a repurposed *chultún* containing the remains of more than 100 subadults was discovered near the Sacred Cenote (Fig. 1c,d)^4,14,15^. As for cenotes, *chultúns* (underground cisterns) are associated with water storage and also ritual activity^16,17^ and they share symbolism with caves^18^. Such subterranean features have long been associated with water, rain and child sacrifice^14,19,20^ and they are widely viewed as access points to the Maya underworld^21,22^. Given the location and context of the Chichén Itzá *chultún*, which was also connected to a small underground cave, it has been speculated to contain children sacrificed to support maize agricultural cycles^19^ or given as offerings to the Maya rain deity Chaac^23^. Sixteenth century Spanish colonial accounts and early twentieth century investigations following the dredging of the Sacred Cenote popularized the notion that young women and girls were primarily sacrificed at the site^24,25^ but more recent osteological analyses indicate that the bodies of both males and females were deposited in the Sacred Cenote^7,26^. Systematic investigations of sacrificial assemblages across the Maya region have confirmed that both males and females were subject to ritual killing but, because most sacrificed individuals at Classic Maya sites are juveniles, precise sex distributions cannot be determined using conventional osteological approaches alone^19,26^. Sixteenth century Spanish sources record that such children were obtained locally by kidnapping, purchase and gift exchange^19,27,28^, although recent isotopic studies suggest that at least some individuals within the Sacred Cenote were non-local and may have originated as far away as Honduras or Central Mexico^1^. Nevertheless, despite more than a century of research, much about child sacrifice and the ceremonial use of subterranean features as ritual mass graves at Chichén Itzá remains unknown.

To better understand the origin and biological relationships of the sacrificed children to each other and to present-day inhabitants of the region, here we used a combined bioarchaeological and genomics approach to investigate 64 subadults interred within a *chultún* near the Sacred Cenote (Fig. 1c) and compare them to 68 present-day Maya inhabitants of the nearby town of Tixcacaltuyub, as well as to other available ancient and contemporary genetic data from the region. The community of Tixcacaltuyub has been collaborating with our research team for many years and their perspectives informed the development of this manuscript (Supplementary Methods: ‘Community engagement activities’). Our analyses, comprising of ancient human genetic data analysis, bone collagen stable isotope analysis of carbon and nitrogen and radiocarbon dating (Supplementary Table 1) show that all *chultún* subadults were male and that close relatives were present in the mass burial, including two sets of monozygotic twins. Stable isotope analysis indicates that related children consumed more similar diets and that overall the diet of Chichén Itzá children was comparable to that of other Classic period populations throughout the Maya Lowlands (Supplementary Table 2 and Supplementary Fig. 4). Genetic comparison to other ancient and present-day people shows long-term genetic continuity in the Maya region but indicates allele frequency shifts in immunity genes at the human leukocyte antigen (HLA) class II locus, specifically an increase in HLA-DR4 alleles which provide greater resistance to *Salmonella enterica* infection, the causative agent of an enteric fever previously identified in a colonial-era mass grave in Oaxaca, southern Mexico, which was associated with the 1545 *cocoliztli* pandemic^29^.

## Genome and immune-genes data generation

Bone samples from ancient individuals found in the *chultún* burial at Chichén Itzá (from here onwards referred to as YCH) were collected, processed and analysed according to protocols designed for ancient DNA (aDNA) work in dedicated facilities^30^. Because not all skeletal elements could be unequivocally assigned to a single individual, only left petrous bones were collected to avoid sampling individuals more than once. Radiocarbon dating (*n* = 26) showed that the *chultún* was in use for at least 500 years from the initial florescence of the site in the early seventh century ad to its height during the tenth century ad until the mid-twelfth century (Supplementary Fig. 1 and Supplementary Table 1).

Ancient DNA was successfully retrieved from all 64 YCH individuals. In addition, DNA was collected from blood samples of 68 modern-day inhabitants of the town Tixcacaltuyub, Yucatán, Mexico (hereafter TIX) to compare modern and ancient inhabitants of the region. The extracted genomic material was built into either uracil DNA glycosylase (UDG)-treated (for YCH) or non-UDG-treated genomic libraries (for TIX) and sequenced to a depth of about 5–11 million reads to assess DNA preservation and authenticity. We then built 11 single-stranded, UDG-treated libraries to further increase the analysable data for a subset of YCH individuals (Supplementary Methods: ‘Preparation of single-stranded libraries’). We performed quality control assessment to ensure acceptable (less than 5%) contamination amounts with two methods implemented as part of the nf-core/Eager pipeline^31^. All TIX and 56 YCH individuals yielded sufficient human DNA for analysis (more than 0.1%) and after a reconditioning procedure we further enriched these DNA libraries for a panel of 1.2 million ancestry-informative single nucleotide polymorphisms (SNPs, ‘1,240 K’), the mitochondrial genome (mtDNA) and a panel of immune genes^32–34^. After sequencing the enriched genomic libraries, we obtained about 40 million reads per library; on these data we performed further quality control and conducted population genetics analyses and HLA typing.

## Genetic kinship and twins in the *chultún*

Coverage comparisons of X and Y chromosome SNPs assigned all YCH subadult individuals as genetically male and confirmed the recorded sex for all TIX participants^35^. The pairwise mismatch rate analyses^36^ (Fig. 1e and Supplementary Fig. 2) for YCH support the presence of two pairs of identical twins (YCH018–YCH019 and YCH033–YCH054) and nine other close relative pairs (YCH016–YCH017, YCH017–YCH018, YCH017–YCH019, YCH034–YCH041, YCH036–YCH038, YCH042–YCH049, YCH047–YCH057, YCH049–YCH057 and YCH059–YCH060). Overall, 25% (*n* = 16) of the analysed children in the ritual interment are closely related to another child within the *chultún*.

## Isotopic patterns in the children’s diets

The δ^13^C and δ^15^N measurements of bone collagen (Supplementary Table 1 and Supplementary Fig. 3; also Supplementary Methods: ‘Stable isotopes analyses’) provided δ^13^C values between −13.9‰ and −7.6‰ (mean and s.d. = −9.9‰ ± 1.5‰) and δ^15^N values ranging from 5.9‰ to 14.0‰ (mean and s.d. = 9.7‰ ± 1.5‰). Overall, these values are similar to those reported at other Classic period Maya Lowland sites (Supplementary Table 2 and Supplementary Fig. 4) and are consistent with dietary evidence in the Yucatán Peninsula^37,38^ and at other Classic Maya sites^39–42^. It is possible that the diets of some individuals with higher δ^15^N values (for example, YCH004, YCH008, YCH023, YCH039, YCH047 and YCH061) may have included aquatic resources^37^ but could also indicate other dietary variations related to social status^43^ or result from breastfeeding influence^44^. Without any more contextual information or local baseline isotopic data from associated fauna, it remains challenging to more precisely determine individual diets. Nevertheless, the comparison of our data with published results (more than 450 individuals studied from the Late Classic and Terminal Classic periods)^45–47^ suggest that the 54 *chultún* individuals consumed significant amounts of C_4_ terrestrial resources combined with varying amounts of terrestrial C_3_ protein and freshwater and/or marine resources. This is consistent with previous archaeological investigations focusing on documented Classic Maya diets^37–39,41,42,48^. The isotopic values of related individuals fall close to each other, suggesting dietary similarity (Supplementary Fig. 5).

## Genetic continuity in the Maya region

**Fig. 2:**
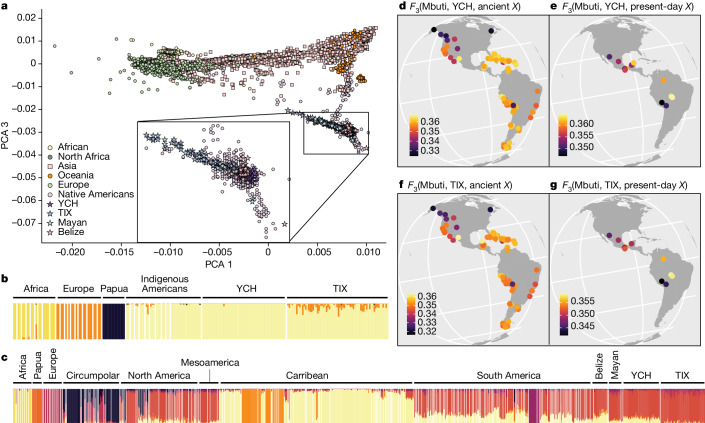
Genetic similarities between Chichén Itzá, Tixcacaltuyub and present-day and ancient American Indigenous groups. **a**, PCA showing ancient Chichén Itzá (YCH) individuals and present-day Tixcacaltuyub (TIX) in a worldwide PCA plot. **b**,**c**, Admixture analyses showing the clustering from *k* = 4 for YCH, TIX and different continental genetic sources (**b**) and the clustering for the newly produced samples at *k* = 8 (lowest cross-validation errors), with a more comprehensive list of Indigenous American populations (**c**). **d**,**e**, Outgroup *F*_3_ statistics in the form *f*_3_(Mbuti; YCH, *X*) where *X* are present-day groups from the Americas (**d**) and published ancient groups and individuals (**e**). High similarities are indicated by warmer colours; low similarities by darker colours. **f**,**g**, Outgroup *F*_3_ statistics in the form *f*_3_(Mbuti; TIX, *X*) where *X* are present-day groups from the Americas (**f**) and published ancient groups and individuals (**g**). High similarities are indicated by warmer colours, low similarities by darker colours. A list for the populations/individuals used in these analyses can be found in the Supplementary Information: source populations for the population genetics analyses.

We performed a principal component analysis (PCA) based on worldwide populations and present-day individuals from the Americas^49^ (Fig. 2). As expected for Mesoamerican populations, YCH cluster closely together on a worldwide PCA with unadmixed Indigenous American populations. Some individuals from TIX are shifted towards Europeans, suggesting genetic admixture (Fig. 2). When both YCH and TIX are projected onto present-day Indigenous American populations from North, Central and South America^50^ they cluster with present-day Maya (Fig. 2a). Unsupervised admixture analyses using a subset of populations from Africa, Europe, Oceania and the Americas (Fig. 2b,c) showed no signs of admixture in the YCH individuals and low contributions of European and African ancestry for the TIX individuals, with some (*n* = 18) showing no indications of non-Indigenous American genetic contributions. It is of interest to note that a genetic component maximized in ancient populations from the Caribbean region is present in both ancient Mayans from Belize^49,51^ and YCH but it is nearly absent in the genetic make-up of present-day Mayans^52^ and TIX. Admixture with other populations from Mesoamerica (where the component has not yet been detected), or genetic drift, could explain this component fading away in present-day Mayans.

**Extended Data Fig. 1:**
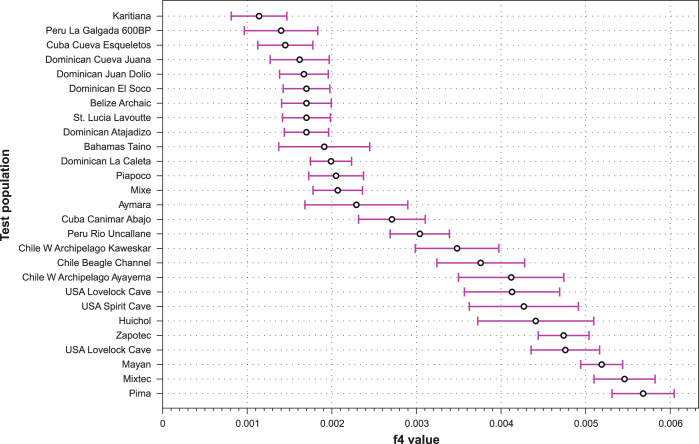
F4 test of the form *f*_*4*_(Mbuti, YCH; *test*, TIX). Values are plotted (circle) with their corresponding error bars (in purple). For all tested populations, the value of the test is positive, indicating that YCH And TIX shared more alleles between them than to any of the Native American populations tested.

To test the genetic affinities to present-day and ancient American populations, we calculated an outgroup *F*_3_-statistic of the form *f*_3_(outgroup, target, test), using Mbuti from Sub-Saharan Africa^52,53^ as an outgroup, a panel of Indigenous American populations as the target and either TIX or YCH as the test. The highest genetic similarities detected for both YCH and TIX (Fig. 2) included Central and South American groups. TIX share the highest drift with the ancient individuals from Chichén Itzá (Fig. 2). Among the ancient populations tested, a 9,300-year-old individual from the Mayahak Cab Pek site in the Maya Mountains in southern Belize^49^ and other analysed individuals, also from Belize but from a more recent context^51^, are genetically most similar to the ancient Chichén Itzá individuals, suggesting long-term genetic continuity in the Maya region. We tested if YCH and TIX are closer to each other than to other Indigenous American populations using an *F*_4_-statistic of the form *f*_4_ (Mbuti, TIX, target, YCH), using a subset of the highest *F*_3_-scoring Indigenous American populations. Our results indicate that a few members of the target Indigenous American populations we tested are more closely related to TIX (Extended Data Fig. 1). This may suggest that, even though TIX are genetically related to the ancient inhabitants of Chichén Itzá, the genetically closest population to Chichén Itzá may no longer exist or has not been sampled yet.

We then applied qpWave^50^, an ancestry composition modelling approach that evaluates the likelihood that two individuals/groups derive from the same ancestry source relative to a given set of population references. The resulting *P* values (Kaqchikel: 0.08, ‘Maya’ (from ref. ^54^): 0.755) suggest that YCH and present-day Mayan groups share the same ancestry. TIX can be modelled as a mixture of YCH, Spanish and Yoruba; the working model (*P* = 0.11) suggests a composition of 92% Indigenous American component, 7% of European genetic contribution and 0.03% African ancestry. Using a maximum-likelihood test for genetic continuity^55^, we could formally test that TIX is a direct genetic descendant population from YCH (average genetic drift across individuals: about 0.5, *P* approximately 1.0 for each TIX individual). For 53 YCH and all 68 TIX individuals, the mtDNA haplogroup could be determined and the frequency of haplogroups (A, B, C and D) is almost identical between both groups. However, from the haplotypes, which represent a higher level of genetic resolution, it is clear that the diversity of mtDNA is higher in YCH than in TIX (Supplementary Fig. 6). The mtDNA haplogroups and haplotype lineages correspond to those previously reported for both ancient^56,57^ and present-day^58,59^ Maya. All Y chromosome (Y-Chr) haplotypes (Supplementary Fig. 6) recovered from the Chichén Itzá individuals (*n* = 51) are part of the Q family (prevalent among Indigenous Americans), whereas more than half of the TIX Y-Chr (*n* = 19) are European (47.37%) and Middle Eastern (5.26%), reflecting a strong sex bias in the admixture process during and after the colonial period, as has been previously described in other Latin American populations^60–63^.

## Genomics of metabolic pathways in Mayans

Using the SNP data generated for both populations, we calculated locus-specific branch lengths (LSBL)^64,65^ to test for genome-wide selection for both YCH and TIX. We performed two LSBL comparisons: first, YCH versus Iberians from Spain (from the 1000 Genomes project^66^) and Han from China (from the 1000 Genomes project); and second, TIX versus Iberians and YCH to test for selection separate from YCH and Iberians. Among the top 0.5% annotated SNPs, we found 29 genes involved in lipid metabolism for YCH, including the previously reported fatty acid desaturases (*FAD*s) genes^67^ and we found 20 genes for TIX, including FTO alpha-ketoglutarate dependent dioxygenase (*FTO*) and transcription factor 7 like 2 (*TCF7L2*), both of which have been associated with metabolic traits in Latin American and particularly Mayan, populations^68–70^ (Supplementary Tables 3–5). The SNPs of certain genes, like those belonging to the adenylate cyclase family (ADCY), were also among the top 0.5% for TIX, which is consistent with previous reports^71^, but not among YCH, which may point to differential selection before and after the colonial period. We then searched for enriched gene ontology pathways using GoWinda^72^. Although both groups exhibit enriched (adjusted *P* < 0.05) gene ontology terms associated with metabolic pathways (Supplementary Tables 6 and 7), YCH shows an increase in fertility-associated biological processes (such as oogenesis, steroid hormone mediated signalling pathway, ovulation cycle and oestrous cycle, among others), whereas cholesterol- and lipid-metabolic pathway terms (such as negative regulation of lipid biosynthetic process, cholesterol homeostasis and sterol homeostasis) appear more prominently in TIX.

## HLA genes point to shifts in immunity

**Fig. 3:**
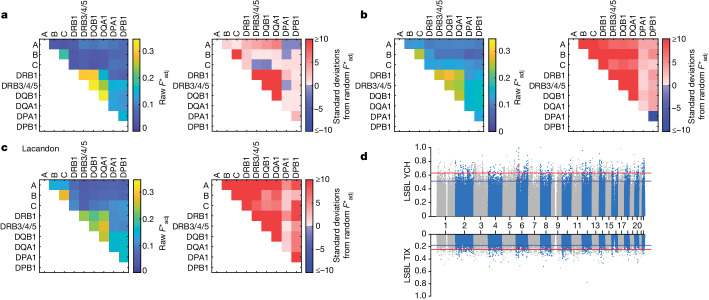
Evidence for selection in the HLA region. **a**–**c**, Pattern of non-overlap associations for the genes in the HLA region as measured by F*_adj_ (heatmaps on the left) and the difference between those observed amounts of non-overlap and randomized allelic associations, in units of standard deviation (heatmaps on the right), for ancient Mayans from Chichén Itzá (YCH) (**a**), present-day Mayans from Tixcacaltuyub (TIX) (**b**) and present-day Lacandon Mayans from Chiapas (southeast Mexico)^76^ (**c**). **d**, Manhattan plots of genome-wide LSBL values for YCH (top) and TIX (bottom). HLA loci are shown in red. Top 0.1% (red line) and 0.5% (blue line) of all statistics are indicated.

For genes involved in immunity, we could detect 15 and 7 HLA region SNPs among the top 0.5% annotated SNPs for YCH and TIX, respectively, showing signs of positive selection (Fig. 3). None of the SNPs were shared by both YCH and TIX and none was found to be under selection in a previous study in ancient and modern Indigenous Americans from the Northwest Coast of North America^73^. The SNPs found on the YCH individuals are located in the *HLA-B*, *-DRB1*, *-DQA1*, *-DQA2, -S, -X, HLA-DOA* and *-DQB1* genes or nearby regions, whereas TIX SNPs are found in or around the *HLA-C*, *-DQA1*, -*DQA2* and *-DQB1* genes (Supplementary Table 8). Using a multilocus model of host–pathogen co-evolution with allele-specific adaptive immunity, it has been shown that, if selection from a pathogen maintains associations between host recognition loci (such as in the HLA system), alleles at those loci would not only be in linkage disequilibrium but might also exhibit non-overlapping associations^74^. For that reason, we analysed the pattern of non-overlapping associations^74,75^ (Fig. 3 and Supplementary Figs. 7 and 8) to test for pathogen-driven selection on HLA associations in YCH, TIX and the previously analysed Lacandon Mayan from the highlands of Chiapas, southeast Mexico^76^. We measured the ${f}_{{\rm{adj}}}^{* }$ metric (a parameter used to rank the strength of non-overlapping associations; Supplementary Methods: ‘Non-overlapping HLA associations’)^74^ between different pairs of HLA loci. We also measured the difference between our observed amounts of non-overlap and that which would be observed for randomized allelic associations, in units of standard deviations.

When compared with the ancient data, the present-day Maya data seem to have a higher degree of non-overlap (Fig. 3a–c), which would suggest that the analysed modern populations have experienced selection on the HLA locus, potentially due to pathogen exposure. When we compare the HLA allelic frequencies (Supplementary Tables 9–19) of YCH and TIX, we detect a statistically significant shift (*P*_corr_ < 0.05) in eight alleles: three HLA class I alleles and five HLA class II alleles, after correcting for several comparisons. There is a significant decrease in the frequency of alleles HLA-B*40:02 (0.2447 versus 0.0821; *P*_corr_ = 0.0296), HLA-DQA1*03:03 (0.1277 versus 0.0224; *P*_corr_ < 0.0218) and HLA-DQB1*04:02 (0.1809 versus 0.0299; *P*_corr_ = 0.0249), whereas HLA-A*68:03 (0.0532 versus 0.2687; *P*_corr_ = 0.0003), HLA-B*39:05 (0.0532 versus 0.2687; *P*_corr_ = 0.0437), HLA-C*07:02 (0.2021 versus 0.3955; *P*_corr_ = 0.0369), HLA-DQB1*03:02 (0.4894 versus 0.7015; *P*_corr_ = 0.0177) and HLA-DRB1*04:07 (0.2340 versus 0.4627; *P*_corr_ = 0.0114) increased in frequency in TIX when compared to YCH. For TIX, we found that 88% of HLA haplotypes have been reported previously in Indigenous American populations, 10% of which are probably of European origin and 2% of which represent African haplotypes. All YCH HLA haplotypes are consistent with those found in Indigenous American populations, particularly among Maya^76–78^.

The HLA class II region has been previously implicated in selection events before and after the contact with Europeans in the sixteenth century in the Americas^73^ and in the resistance to *S. enterica* infection^79–81^. Hence, we were interested to test how the alleles for which there is a significant shift would react to peptides derived from *Salmonella*. To do so, we selected 18 *Salmonella* proteins for which there is evidence of immunogenicity in humans and presented in silico peptides derived from them to the HLA class II molecules found both in YCH and TIX (Supplementary Table 20 and Supplementary Methods: ‘In silico binding prediction assays’) using the NetMHCIIPan binding prediction method^82^ implemented in the Immune Epitope Database (IEDB) Analysis Resource (http://tools.iedb.org/mhcii). Strong binding means the bound peptide is more likely to initiate an immune response against that peptide, whereas weaker binders are not as successful in eliciting an immune response. The best HLA-DR strong binders were HLA-DRB1*14:02, HLA-DRB1*04:07, HLA-DRB1*16:02 and HLA-DRB1*04:17; whereas the best strong binders for HLA-DQ were HLA-DQA1*05:01/DQB1*03:01, HLA-DQA1*05:05/DQB1*03:03 and HLA-DQA1*03:01/DQB1*03:03. The HLA molecules with the fewest strongly bound peptides are represented by HLA-DRB1*14:06, HLA-DRB1*04:04, HLA-DRB1*04:05 for HLA-DR and HLA-DQA1*03:03/DQB1*04:02, HLA-DQA1*04:01/DQB1*04:02 and HLA-DQA1*03:01/DQB1*03:02 for HLA-DQ. Overall, the lowest number of peptides, either strongly or weakly presented, by HLA class II molecules correspond to HLA-DR alleles DRB1*08:02, DRB1*04:04 and DRB1*14:06 and the same HLA-DQ molecules listed before. Broadly, the frequencies of HLA-DRB1*04:07 (strong binder) and HLA-DQB1*03:02 (weak binder, in linkage disequilibrium with HLA-DRB1*04:07) go up in TIX, whereas HLA-DQA1*03:03 and HLA-DQB1*04:02 go down in TIX and are weak binders.

## Discussion

Archaeogenetics offers an opportunity to investigate aspects of past Maya ritual practice, biological kinship, sex and genetic history that can be difficult to infer using more conventional archaeological methods. Here we investigated the subadult remains of 64 individuals who were ritually interred over a period of 500 years in a Classic period *chultún* located near the Sacred Cenote at the ancient Maya city of Chichén Itzá. In contrast to the human remains in the Sacred Cenote, we found that all analysed subadults in the *chultún* were male, demonstrating a strong preference for the ritual sacrifice of male children in this context. Genetic analysis also showed the presence of related individuals within the *chultún*, including two sets of monozygotic twins and nine other close relative pairs. As such twins occur spontaneously in only 0.4% of the general population^83^, the presence of two sets of identical twins in the *chultún* is much higher than would be expected by chance. Overall, 25% of the children had a close relative within the assemblage, suggesting that the sacrificed children may have been specifically selected for their close biological kinship. Moreover, this may underestimate the true number of relatives present in *chultún* as only 64 of the estimated 106 individuals in the *chultún* had a preserved petrous portion of the left temporal bone available for analysis. The further finding that the closely related children in each set seem to have consumed a similar diet and died at a similar age suggests that they have been sacrificed during the same ritual event as a pair or twin sacrifice.

Twins are especially auspicious in Mayan mythology and twin sacrifice is a central theme in the sacred K’iche’ Mayan Book of Council, the *Popol Vuh*^84^, a book whose antecedents can be traced to the Maya Preclassic period^85^. In the *Popol Vuh*, the twins Hun Hunapu and Vucub Hunahpu descend into the underworld and are sacrificed by the gods following defeat in a ballgame. The head of Hun Hunapu is then hung in a calabash tree, where it impregnates a maiden who gives birth to a second set of twins, Hunapu and Xbalanque. These twins, known as the Hero Twins, then go on to avenge their father and uncle by undergoing repeated cycles of sacrifice and resurrection to outwit the gods of the underworld. The Hero Twins and their adventures are amply represented in Classic Maya art^5^ and given that subterranean structures were viewed as entrances to the underworld, the twin and relative sacrifices within the *chultún* at Chichén Itzá may recall rituals involving the Hero Twins.

In comparing the subadults in the *chultún* to other ancient and present-day populations in the Maya region, we find evidence of long-term genetic continuity, which also suggests that the sacrificed children and sibling pairs at Chichén Itzá were obtained from nearby ancient Maya communities. Among present-day individuals at TIX, we detect evidence of European and African admixture since the Contact period. Although ancestry contributions from non-Indigenous American sources are low at the genome-wide level, they are strongly asymmetrical with respect to uniparental markers. Whereas all mtDNA haplogroups are Indigenous American, more than half of the TIX Y-Chr are non-Indigenous American (mostly European and Middle Eastern/Mediterranean in origin, consistent with previous reports^60,61^), indicating that a strong male bias towards non-Indigenous American ancestries occurred during the Contact period.

The genetic similarity among Maya groups determined in the population genetic analyses (admixture profiles, *F*_3_ and *F*_4_ and genetic continuity test) allowed us to explore changes in genomic regions encoding functional variants to test for selection in ancient (YCH) and present-day (TIX) Maya. Our findings support previous hypotheses that both lipid metabolism^86,87^ and fertility^86^ are traits that have been recently selected for in Indigenous Americans, probably due to the strong bottlenecks and caloric restrictions experienced by these populations during the early colonial and settlement periods^86,88^. The observed standard deviation of the δ^15^N values (s.d. = 1.5) obtained from the individuals analysed at Chichén Itzá is the highest of all the Late Classic and Terminal Classic Maya sites analysed so far (Supplementary Figs. 3 and 4 and Supplementary Table 2). The overall picture from the reconstruction of palaeodiet in the Maya region shows the consumption of significant amounts of C_4_ foods, probably maize but with geographic variations reflected in microenvironmental differences in available foods and variability in trade networks^45,47^. To explain this variability, it is possible that a considerable portion of the individuals sacrificed at Chichén Itzá may be non-local Maya consuming slightly different diets. Alternatively, previous research indicates that the dietary patterns of the Classic Maya elite tend to exhibit greater variability than those of the general population over time^43,47,89^. This variability is also reflected in the standard deviations of δ^13^C and δ^15^N observed in other locations, such as Altun Ha or Baking Pot (Supplementary Fig. 4 and Supplementary Table 2). Therefore, the differences in protein intake observed in the analysed Chichén Itzá individuals studied could also indicate variations in social status. It also cannot necessarily be excluded that some of the observed δ^15^N variability is a result of breastfeeding influence^90^, as the sampled remains come from individuals estimated to be between 3 and 6 years of age. Thus, caution in interpreting specific dietary variations is warranted in the absence of other contextual information. Our data indicate that those individuals whose DNA showed a close genetic relationship had more similar δ^13^C and δ^15^N values, suggesting that they may have been raised in an extended family network that provided similar care and feeding (Supplementary Figs. 3 and 5 and Supplementary Table 1).

The values for the genetic drift obtained from the genetic continuity test mean that the TIX ancestors went through a serious population decline sometime in the last 1,000 years or so. Over the course of the sixteenth century, it has been demonstrated that wars, famines and epidemics resulted in a population decline, potentially as high as 90%, from 10–20 million Indigenous people living in Mexico at the time of European contact to only 2 million people by the end of the sixteenth century^91–96^. Infectious diseases, such as smallpox, measles, mumps, influenza, *tabardillo* or *matlalzahuatl* (typhus), typhoid and enteric fever, rubella, pertussis, *garrotillo* (severe diphtheria), endemic dysentery, tertiary fevers (malaria) and syphilis^29,97–101^ are argued to have caused large-scale outbreaks in colonial Mesoamerica, significantly contributing to population decline and possibly causing selection at immune-related loci. The HLA class II region has been reported previously to have undergone selection events during the colonial period in the Americas^73^. Notably, we find that three of the alleles whose frequencies change when we compare YCH to TIX are part of the HLA class II region, a finding that is further supported by the SNPs found in the LSBL analyses. One of those alleles (HLA-DRB1*04:07) belongs to an allele group (HLA-DR4) that has been previously reported to be associated with resistance to enteric fever caused by *S. enterica* subspecies in South America^81^ and East Asia^80^. Recently, an archaeogenetic study identified the presence of *S. enterica* sp. Paratyphi C in a mass burial associated with the ad 1545 *cocoliztli* pandemic, suggesting that it was at least one of the causative agents of this pandemic^29^, which had the highest mortality of all recorded colonial-era epidemics. Genomic analysis of ancient *S. enterica* strains strongly supports an introduction to the Americas during the sixteenth century^102^.

The observed increase of the HLA-DR4 allele group among present-day Maya and Mexicans in general^103^, is consistent with selection caused by an epidemic event and subsequent sustained exposure to the pathogen. Further examination of the non-overlapping associations between HLA alleles likewise agrees with the present-day Maya populations having undergone pathogen selection, which has driven their HLA associations to be less overlapping than for the YCH. The in silico binding prediction assays also point to HLA-DRB1*04:07 as a strong binder for *Salmonella*-derived peptides, whereas HLA-DQB1*04:02 and HLA-DQB1*03:03, significantly reduced in present-day Mayans, are weaker binders for the same peptides. Considered together, each line of evidence points to selection event(s) occurring in the HLA region in response to an epidemic event during the colonial period. Such selection would be expected in the face of the intensity of the 1545 *cocoliztli* pandemic and the high number of epidemic events documented in the Maya region since the beginning of the sixteenth century^104–106^. Although it cannot be ruled out that haplotypes with non-overlapping combinations of alleles survived a non-disease related bottleneck by chance, such a scenario would probably result in an increased frequency of alleles already present in the ancient population, which is not what we observe.

Our study shows an intimate portrait of Late and Terminal Classic Maya children at Chichén Itzá, suggesting that the genomic legacy of the ancient inhabitants of this site is still present in communities inhabiting the region surrounding this ancient city. The discovery of two sets of identical twins, as well as other close relatives, in a ritual mass burial of male children suggests that young boys may have been selected for sacrifice because of their biological kinship and the importance of twins in Maya mythology. We show that, at a genome-wide level, the present-day Maya of Tixcacaltuyub exhibit genetic continuity with the ancient Maya who once inhabited Chichén Itzá and we demonstrate through several lines of evidence the involvement of the HLA region in a pathogen-driven selection event(s) probably caused by infectious diseases brought into the Americas by Europeans during the colonial period.

## Methods

### Archaeological and geographical context

Chichén Itzá was one of the largest and most influential Maya cities of the Terminal Classic Period and today stands as an iconic archaeological site among the most representative of pre-Hispanic monumental architecture in the Americas (Fig. 1). The architecture of the site consists of several different styles, with some of the structures resembling those found in Central Mexico (Teotihuacan, Tula) and in the Puuc and Chenes regions (northeastern and central Yucatan, respectively) of the Maya lowlands^107^. The Maya Puuc style represents the second phase of the Florescent period in the Yucatan Peninsula (ad 750–800)^107,108^. Ceramic types and epigraphic and radiometric data suggest that the city emerged as a chief regional political centre in the first half of the nineth century and stayed as such until probably the first half of the eleventh century^108,109^. The ancient samples used in this study belonged to individuals whose skeletons were recovered from the archaeological excavations of a *chultún* (cistern) connected to a natural cave within the archaeological site of Chichén Itzá, Yucatán, Mexico. *Chultúns* were mostly used from the Middle Preclassic to the Late Classic-Postclassic periods (ad 1000–1600) in Mesoamerica from the northern Maya lowlands of the Yucatan peninsula to modern-day Belize and Guatemala in the southern Maya lowlands^16,110^. The sampled skeletons, which have been studied using anthropometry and osteology guidelines at the Centro INAH Yucatán, were recovered between April and June 1967, during the construction of a new runway close to *El Castillo* (‘The Castle’, the main basament of the archaeological site and a building with special calendrical significance^4,111^) and 300 m northeast to the Sacred Cenote of Chichén Itzá. The *chultún* had stones that had been worked in the style that characterized the Florescent period, which would indicate that it had been built around the Late or Terminal Classic periods of Chichén Itzá^4,110^. The chamber and the adjacent cave contained many skeletal remains, some of them in anatomical position. The many skeletons and the position in which they were found, as well as them being covered by mostly undisturbed bark and limestone powder^4^, are mostly consistent with a mixed secondary/primary burial with periodic clearance, which points to the burial having a cultural motivation, probably as an offering^4^ related to an important and recurring ceremony^4,14^. There was a minimum of 106 skeletons of infants and young children (according to the number of temporal bones) without any apparent ordering or anatomical context, except for a group of skulls and skullcaps aligned from north to south from the wall separating the chamber and the cave to the southern limit of the cave^14^. Many ceramic and clay objects, as well as animal bone remains, were also found in the cave^4^. The ceramic styles and the archaeological context indicate that the *chultún* was used during the Florescent period, contemporary to the use of another ritual cave near Chichén Itzá known as Balankanché (ad 860 ± 130)^4,112^. A radiocarbon test on a sample of the bark covering the bones from the *chultún* burial has also suggested a use date of around ad 920 ± 60 (ref. ^107^). However, our ^14^C dates (Supplementary Fig. 2) suggest that the ritual burial was performed both before (around ad 600) and after (around ad 1100) the Florescent period. According to the age determination by tooth eruption method^113^, most (about 50%) of the subadult individuals were found to belong to the age group of 3–6 years old^4^. The fact that the age of the children differs from the age group normally related to death in children associated with infectious diseases according to palaeodemography^19,113–115^ further supports the interpretation of this burial as a sacrificial offering^4^.

The biological samples from present-day Maya were obtained from individuals of self-reported Native American Maya ancestry from Tixcacaltuyub. The village of Tixcacaltuyub, part of the municipality of Yaxcabá, is located 90 km southeast of Merida, 16 km off Sotuta and is located 55 km southeast of Chichén Itzá (Fig. 1). It has around 2,100 inhabitants. Ethical approval for the collection of blood samples from individuals from Tixcacaltuyub, Yucatán, Mexico, was granted by the Committee of Ethics and Research, Autonomous University of Yucatán (UADY), Mexico. All subjects were informed about the objectives and methods used and signed an informed consent form. To avoid potential bias, participants with any HLA-associated clinical condition or cancer^116,117^ were excluded.

### Inclusion and ethics

The community of Tixcacaltuyub self-identifies as a Mayan community and has been in a years-long cooperative relationship with the Chemistry and Nursing Faculties of UADY, Mérida, Yucatán, following projects investigating the relationship between health and lifestyle in the community. J.C.L.R., M.E.M.M. and J.C.T.R., together with C. Tzec-Puch, have been working in collaboration with the community to communicate results of clinical tests and to develop health interventions in Tixcacaltuyub. As a result of these interventions, healthcare barriers and opportunities have been identified and work is now underway with the active participation of the community to implement a co-responsible model of healthcare. In April 2023, J.C.L.R. and R.B. visited Tixcacaltuyub to hold meetings with participants and elementary, high school and UADY students to return the results of the genetic findings and to collect community feedback, including how they reconcile these results with their own views. Feedback from these engagements has been incorporated into the final manuscript (Supplementary Methods: ‘Community engagement activities’). As part of our strategies for outreach and making our results available to a broader audience, we included a non-peer reviewed, Spanish translation of the main manuscript (Supplementary Note 1).

### Individuals and samples

We acknowledge the Council of Archaeology, National Institute of Anthropology and History (INAH, Mexico City, Mexico) for the permit granted to analyse the individuals from the Chichén Itzá Chultun collection (project: *Caracterización genética de una muestra de población de Chichén Itzá para el periodo Clásico Tardío a partir del análisis del ADN*; official notice no. 401.1 S.3-2017/482; 10.03.2017). We analysed the material recovered from the left petrous bones of 64 individuals (YCH) from Chichén Itzá. The bone specimens were collected under controlled conditions in the Osteology Laboratory of the Centro INAH Yucatán with a protocol devoted to minimize contamination. Photographic records of the samples were maintained throughout the whole procedure. The present-day DNA was extracted from blood samples obtained from 68 unrelated adult individuals (TIX) from Tixcacaltuyub, Yucatán, Mexico under a protocol approved by the Committee of Ethics and Research, UADY, granting permit to collect blood samples and carry out analyses on the genetic material obtained from such samples (project: *Bienestar Comunitario: Proyecto de capacitación para la autogestión de la salud de personas con DT2 y sus familias, en la comunidad de Tixcacaltuyub y Yaxcabá*; official notice no. F-FENC-SAC-14/REV: 04; registry no. 09/17) and performed according to the requisites of the Helsinki Declaration (2008) and the General Health Law of Mexico. For YCH, bone powder (about 90 mg) was obtained from the densest part of each petrous bone in facilities dedicated to ancient DNA protocols and after a bleach/rinse and ultraviolet decontamination protocol^30^. For TIX, 5 ml of peripheral blood were obtained by venepuncture after each participant was informed about the procedure, the aim of the protocol and the potential risks, and after signing a consent letter. For TIX, the DNA was extracted in a dedicated laboratory at UADY. Their samples were anonymized from this point onwards.

### ^14^C dating

According to the report issued by The Curt-Engelhorn-Centre for Archaeometry (Mannheim, Germany), the portions of the petrous bone of 26 YCH samples were pretreated and analysed using a standardized procedure. Collagen was extracted from the bone samples (approximately 1 g, using a modified version of the Longin method^118^), purified by ultrafiltration (fraction greater than 30 kD) and freeze-dried. The collagen was then combusted to CO_2_ in an elemental analyser. The CO_2_ was then converted catalytically to graphite and analysed using a MICADAS-type AMS system (Ionplus AG). The isotopic ratios ^14^C/^12^C and ^13^C/^12^C of samples, calibration standard (oxalic acid II), blanks and control standards were measured simultaneously in the AMS system. ^14^C-ages are normalized to δ^13^C = −25‰ (ref. ^119^) with a typical uncertainty of 2‰ and calibrated using the dataset IntCal20 and the software Oxcal (v.4.3.2)^120,121^.

### Stable isotope analyses

Direct insight into dietary trends among past populations enables the investigation of connections between diet and social status, cultural customs linked to food, environmental impacts on subsistence and perhaps even individual mobility^122,123^. Measurements of bone collagen δ^13^C and δ^15^N disproportionately reflect the protein component of an individual’s diet during the period of tissue formation and, to a lesser extent, the lipid and carbohydrate sources^124^. To reconstruct the diet of the subadult individuals from Chichén Itzá studied, we analysed bone collagen from temporal and petrous bones (Supplementary Fig. 4). Collagen was extracted using a standard procedure^125^ (Supplementary Methods). The atomic C:N ratio along with the collagen yields were used to determine the quality of collagen preservation. Collagen yields of more than 1% in weight are considered acceptable for carbon and nitrogen values^126^, whereas the C:N ratio should range from 2.9 to 3.6 for archaeological samples^127^ (Supplementary Table 1).

### DNA extraction

For YCH, the bone powder was decalcified and proteins were digested by an overnight incubation (more than 16 h) at 37 °C in a buffer containing EDTA and Proteinase K^30^. The DNA was purified from the supernatant by a silica column-based method using a silica column for high volumes assay (High Pure Viral Nucleic Acid Large Volume Kit, Roche Molecular Systems). DNA was eluted in 100 µl of TET (10 mM Tris, 1 mM EDTA and 0.05% Tween) and frozen at −20 °C until library preparation^128^. For the contemporary participants, 5 ml of peripheral blood were collected in BD Vacutainer blood collection tubes containing K_2_-EDTA (Becton, Dickinson and Company) and the DNA was extracted using the Quick-DNA Miniprep Plus kit (Zymo Research Corporation) following the developers’ instructions. Because our protocols are optimized for short-length aDNA and to avoid potential bias through laboratory methods, we sheared the DNA extracted from modern individuals using ultrasonic DNA shearing to an average length comparable to that of aDNA. Therefore, 50 μl of a 50 ng μl^−1^ dilution of each of the modern DNA samples were sheared to an average fragment length of 150 base pairs using a Covaris M220 Focused ultrasonicator (Covaris).

### Library preparation

We built non-UDG-treated libraries using 15 μl of each YCH extract to assess the authenticity of the extracted DNA after obtaining the characteristic damage plots associated with aDNA^129^. We then used 20 μl of each YCH extract to build UDG-half libraries with Illumina-specific adaptors following a modified double-stranded library preparation protocol as previously described^130^. We built non-UDG-treated libraries for TIX extracts using 20 μl of each modern DNA extract. Both YCH and TIX libraries were quantified using quantitative PCR (qPCR) with the IS7 and IS8 primers in a quantification assay using a DyNAmo SYBR Green qPCR Kit (Thermo Fisher Scientific) on the LightCycler 96 platform (Roche Diagnostics).

For DNA extracts from the individuals for which the data obtained by sequencing the UDG-treated libraries was not enough, we used a new library protocol that uses directional splinted ligation of Illumina P5 and P7 adaptors to convert natively single-stranded DNA and heat-denatured double-stranded DNA into sequencing libraries in a single enzymatic reaction using the fully automated version of the protocol^131–133^. We followed the protocol as described by the authors using 25 μl of DNA extract for each individual (Supplementary Methods: ‘Preparation of single-stranded libraries’).

Each library was identified with the respective pair of indexes in double-100 μl reactions using PfuTurbo DNA Polymerase (Agilent Technologies) for the double-stranded libraries and AccuPrime Pfx DNA Polymerase (Thermo Fisher Scientific). The indexed products for each library were pooled, purified over silica columns using the MinElute PCR Purification Kit (QIAGEN N.V.), eluted in 44 µl of TET and again qPCR quantified, now using the IS5 and IS6 primers. Conditioning for sequencing included the amplification of the purified product in 4 × 100 µl reactions using Herculase II Fusion DNA Polymerase (Agilent Technologies) following the manufacturer’s specifications with 0.3 µM of each IS5/IS6 primer, following a purification over silica columns also using the MinElute PCR Purification Kit and elution in a final volume of 22 µl of TET. A total of 2 ml of the conditioned product were diluted 1:10 and quantified using the Agilent 2100 Bioanalyzer DNA 1000 protocol (Agilent Technologies). Independent, equimolar (10 mM final concentration) pools of YCH libraries, TIX libraries and extraction and libraries blanks were then prepared for shotgun sequencing on the Illumina HiSeq 4000 Systems platform (Illumina)^134,135^. YCH libraries were sequenced to 5 million reads depth, whereas TIX libraries were sequenced to 11 million reads depth and then all of them were analysed to obtain basic quality control parameters using nf-core/eager v.2.3.4 (ref. ^31^).

### Whole-genome and immune-genes captures

Using an in-solution capture approach based on modified immortalized probe sequences^136^, target immunity genes sequences, mtDNA, Y chromosome or a panel of 1,237,207 SNPs were enriched from the total DNA in the sequencing libraries^32,33,137–140^. After enrichment, captured library pools were paired-end sequenced on the Illumina Hiseq 4000 (Illumina) platform with 75 cycles providing about 20 million reads per assay.

### Quality control and data processing

For both YCH and TIX libraries, we performed analyses of the captured sequence data using nf-core/eager v.2.3.4 (ref. ^31^). AdapterRemoval v.2 (ref. ^141^) was used to trim adaptor sequences and to remove adaptor dimers and low-quality sequence reads (minimum length, 30; minimum base quality, 20). Preprocessed sequences were mapped to the human genome assembly GRCh37 (hg19) from the Genome Reference Consortium^142^ using BWA v.0.7.12 (ref. ^143^) and a seed length of 32. In the case of the ancient samples, the C to T misincorporation frequencies typical of aDNA were obtained using mapDamage 2.0 (ref. ^144^) to assess the authenticity of the aDNA fragments from the half-UDG-treated libraries. Genetic sex of the analysed individuals was assigned using SNP capture data by calculating the ratio of average X chromosomal and Y chromosomal coverage to the average autosomal coverage normalized by the chromosome length at the targeted SNPs^35^. Samples with an X rate between 0.35 and 0.55 and a Y rate between 0.4 and 0.7 were confirmed male. Analysis of next generation sequencing data(ANGSD) was used to estimate nuclear contamination, as males are expected to be homozygous at each X chromosome position^145^. We used samtools mpileup (parameters –q 30 –Q 30 –B) to generate a pileup file from the merged sequence data of each individual and used a custom script (pileupCaller v.8.2.2; ref. ^146^) to genotype the individuals, using a pseudo-haploid random draw approach. For each position on our capture panel, a random read was drawn for each individual and the allele of that read was assumed to be the homozygous genotype of the individual at that position. To compare with available data from Mesoamerican and Central American populations, we merged our SNPs to the 593,124 SNPs of the Human Origins dataset^52^. Only individuals retaining more than 20,000 SNPs on this assay were kept for downstream analyses.

### Uniparental markers

The mtDNA haplogroups were determined mapping reads to the revised Cambridge reference sequence^147^. For the resulting sequences, we used HaploGrep2 (ref. ^148^) and HAPLOFIND^149^ to assign and confirm the corresponding mtDNA haplogroups (Supplementary Fig. 6). For the Y-haplogroup assignments, we created pileups of reads for each individual which mapped to Y chromosome (Y-Chr) SNPs as listed on the ISOGG Y-DNA Haplogroup Tree (v.15.73; https://isogg.org/tree/). We then manually assigned Y-Chr haplogroups for each individual based on the most downstream SNP retrieved after evaluating the presence of upstream mutations along the Y-Chr haplogroup phylogeny (Supplementary Fig. 6). Full mtDNA haplogroups and Y-Chr genotypes can be found in Supplementary Tables 9 (YCH) and 10 (TIX).

### Genome-wide selection scans

**Extended Data Fig. 2:**
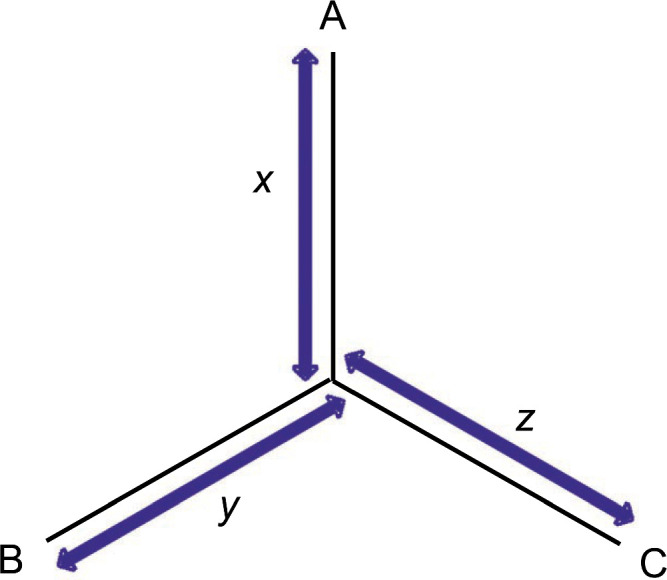
A graphical representation for the locus-specific branch lengths comparisons. The variables *x*, *y* and *z*, (representing either YCH vs. Iberians from Spain and Han from China, or TIX vs. Iberians and YCH, to independently test for selection) are calculated using pairwise F_ST_ distances, d_AB_, d_BC_ and d_AC_, where *x* = (d_AB_ + d_AC_ – d_BC_)/2, *y* = (d_AB_ +d_BC_ – d_AC_)/2, *z* = (d_AC_ + d_BC_ – d_AB_)/2 and A, B and C are the three populations under consideration.

Using the ancient (YCH; *n* = 64) and present-day (TIX; *n* = 68) genomic data, we performed two LSBL comparisons: first YCH versus Iberians from Spain (*n* = 102) and Han from China (*n* = 103) from the 1000 Genomes project^66^ and then TIX versus Iberians and YCH to test for selection separate from YCH and Iberians. After quality control filtering, we removed individual YCH046 based on its position on the PCA. Sex chromosomes were not analysed, SNPs with minor allele frequencies less than 0.01 were excluded, as well as individuals with more than 97% missing data and finally we pruned SNPs for linkage disequilibrium using the following command: --indep-pairwise 200 25 0.4. The top 0.5% SNPs were annotated with biomaRt (release 3.15)^150^. LSBLs *x*, *y* and *z* are calculated using pairwise *F*_ST_ distances, *d*_AB_, *d*_BC_ and *d*_AC_, where *x* = (*d*_AB_ + *d*_AC_ − *d*_BC_)/2, *y* = (*d*_AB_ + *d*_BC_ − *d*_AC_)/2, *z* = (*d*_AC_ + *d*_BC_ − *d*_AB_)/2 and A, B and C are the three populations under consideration (Extended Data Fig. 2).

### HLA allele assignment, haplotype reconstruction, statistical analyses and in silico binding prediction assays

We applied a development version of OptiType^151^ on sequence data from the immune-captured libraries, mapped against a custom HLA reference panel containing 1,025 alleles with ‘common’ or ‘intermediate’ CIWD 3.0 designation (https://github.com/FRED-2/OptiType, tag GRG). The number of possible best matches was set to infinity. In this way we obtained HLA class I and class II alleles for the genes *HLA-A*, *HLA-B*, *HLA-C*, *HLA-DRB1*, *HLA-DQA1*, *HLA-DQB1*, *HLA-DPA1* and *HLA-DPB1*. Of the 882 raw allele calls, 72 (8.1%) with anomalous coverage patterns induced by reads cross-mapping to several loci were overruled in favour of secondary allele predictions. Haplotypes were assigned on the basis of previously reported frequencies and properties such as linkage disequilibrium^77,152,153^ and kinship when possible. To compare the HLA frequencies between precontact Chichén Itzá and postcontact Tixcacaltuyub, we performed Fisher exact tests^154^ and adjusted the *P* values for several comparisons according to the Benjamini–Hochberg correction^155^ which control the false discovery rate (FDR). We use the FDR adjustment as, compared to methods such as the Bonferroni correction, the FDR approach controls for a low proportion of false positives, instead of ensuring the existence of absolutely no false positives, resulting in increased statistical power^155^. It has been argued^74^ that certain types of non-overlapping association between HLA loci may be a signature of pathogen selection. The ${f}_{{\rm{adj}}}^{* }$ metric^74,75,156^ was calculated for each pairwise combination of HLA loci in the dataset, using YCH and TIX haplotypes for which we had data for every HLA locus. To provide a point of comparison for the ${f}_{{\rm{adj}}}^{* }$ score of each locus pair for both YCH and TIX, we generated 5,000 random permutations of the order of the total alleles at one of the loci in each pair and recalculated ${f}_{{\rm{adj}}}^{* }$ for each set of randomized data. We generated distributions of possible ${f}_{{\rm{adj}}}^{* }$ scores for each pair of loci in the dataset that would be obtained if the alleles at those loci were associated entirely at random. We then calculated the difference between the ${f}_{{\rm{adj}}}^{* }$ value calculated from each dataset for each pair of loci and the mean of the ${f}_{{\rm{adj}}}^{* }$ scores calculated from the randomized data for that pair of loci, then divided that difference by the standard deviation of ${f}_{{\rm{adj}}}^{* }$ calculated from the randomized data. The resulting scores allowed us to rank the HLA pairs in order of how extreme an amount of non-overlap they showed relative to entirely random associations between the same alleles (Supplementary Methods: ‘Non-overlapping associations’). To further assess the role of HLA class II alleles in resistance to *S. enterica* infection, we ran in silico binding predictions for the *HLA-DRB1*/*3*/*4*/*5* alleles and *-DQA1*/*DQB1* allele pairs found in YCH individuals and peptides derived from 18 *Salmonella* spp. proteins that have been previously reported to be highly immunogenic in humans (Supplementary Methods*:* ‘In silico binding prediction assays’) using NetMHCIIpan-4.0 (ref. ^82^) as implemented in the IEDB Analysis Resource virtual machine image^157,158^. Binders were classified as strong (rank less than 2%) or weak (2% ≤ rank ≤ 10%) on the basis of the adjusted rank values recommended by the authors and previously published research^82,159^.

### Principal components analysis

Smartpca (v.16000) from the Eigensoft package^160^ was used to calculate principal components (PCs) of variation in the dataset, using the options ‘lsqproject: YES’ and ‘shrinkmode: YES’. Initially, we projected the ancient individuals on PCs calculated on the genetic variation in 371 worldwide populations, to access the continental-level ancestries in the ancient individuals. We then projected the ancient individuals on PCs calculated on variation from 172 Native American populations, both modern and ancient (Supplementary Methods).

### ADMIXTURE analysis

We used ADMIXTURE v.1.3.0 (ref. ^161^), a maximum-likelihood based clustering algorithm, to estimate the genetic structure present in our samples, after excluding variants with minor allele frequency of 0.01 and following linkage disequilibrium pruning using Plink (v.1.90) with a step size of 5, a window size of 200 and an *R*^*2*^ threshold of 0.5 (ref. ^161^). For *K* = 2 to *K* = 13, we estimated the cross-validation error with 100 bootstrap replicates in an unsupervised model using either a panel of populations that are comprised of four ancestry clusters of African, European, Papuan and American origin based on the Simons Genome Diversity Project dataset^54^ plus other relevant populations or a set of the aforementioned populations plus a set of 66 modern and ancient Native American populations to account for different sources of genetic contribution (Supplementary Methods). The lowest cross-validation errors corresponded to *K* = 4 and *K* = 8, respectively.

### *F*-statistics and ancestry modelling tests

To assess the genetic relationships and patterns of admixtures suggested by the PCA and ADMIXTURE analysis, we carried out *F*-statistics analyses using the Xerxes CLI software (v.0.3.0.0) from the Poseidon (v.2.5.0) framework^162^. We calculated *F*_3_-statistics of the form *f*_3_ (Outgroup; target, *X*) to measure the amount of shared genetic drift of populations target and *X* after their divergence from an African outgroup; where *X* is either YCH or TIX and Outgroup corresponds to Mbuti from Congo^52^. We then used a subset of Native American populations (those with the top *F*_3_ scores from our previous test) to perform *F*_4_ tests of the form *f*_4_(Mbuti, YCH; test, TIX) to investigate if the YCH and TIX groups are more closely related to each other or shared an excess of alleles with any population in position test. A negative value implies that either YCH and test or TIX and Mbuti share more alleles than expected under the null hypothesis of a symmetrical relationship between YCH and TIX. On the other hand, a positive value suggests that YCH and TIX share an excess of alleles between themselves. To test whether the newly sequenced individuals were genetically similar enough to be grouped into one group, we used qpWave^50^ v.420, a modelling approach which evaluates the likelihood of two individuals to derive from the same ancestry, relative to a given set of references including worldwide populations. A value below 0.05 indicates a low likelihood, from which we conclude that one of the individuals has a different ancestry profile. For most tests, the respective individuals are consistent with deriving from the same ancestry tested relative to the populations Mbuti.DG, Onge.DG, Papuan.DG, Han.DG, Russia_MA1_HG.SG, USA_Ancient_Beringian.SG, USA_Anzick.SG, Mixe.DG, Mexico_Zapotec.DG, Belize_MayahakCabPek_9300BP, Karitiana.DG, Piapoco.DG, Peru_LaGalgada_4100BP, Pima, Cabecar. There are few exceptions, in which an individual shows low likelihood of sharing ancestry with several other individuals. Particularly high rates of pairs with a low likelihood were produced by the individuals YCH006, YCH011, YCH037, YCH046, YCH59 and YCH064. To investigate which ancestries differentiate the respective individual from most individuals, we calculated the differential affinities using an *F*_4_ statistics of the form *f*_*4*_(Mbuti.DG, *X*; YCH Group, *Y*), in which *X* would be the respective individual and *Y* present-day groups^50^ and published ancient groups and individuals^49,163–172^. In this test, we would assume a positive test score, if the group or individual in *Y* has a higher affinity to the tested individual (*X*) compared to the group. Unexpectedly, there is no ancient or modern group or individual that could explain the behaviour in the qpWave analysis. To test whether present-day Mayan speakers (‘Mayan’ per The Simons Genome Diversity Project^54^, Kaqchikel) were consistent with deriving from the same ancestry as the ancient individuals from Chichén Itzá, we tested them relative to the reference set of tested relative to the populations Mbuti.DG, Onge.DG, Papuan.DG, Han.DG, Russia_MA1_HG.SG, USA_Ancient_Beringian.SG, USA_Anzick.SG, Mixe.DG, Mexico_Zapotec.DG, Belize_MayahakCabPek_9300BP and Karitiana.DG. In both cases, the resulting *P* values (Kaqchikel: 0.08, ‘Mayan’: 0.755) suggest that the ancient and present-day groups share the same ancestry. The same test was not successful for other present-day populations such as Gen-Pano-Carib or Chibchan-Paezan speakers as well as for the seemingly unadmixed individuals from Tixcacaltuyub, possibly attributable to European admixture not detected in the model-based clustering analyses (Fig. 2 and Extended Data Fig. 1). When modelling their ancestry composition relative to the populations Mbuti.DG, Onge.DG, Papuan.DG, Han.DG, Russia_MA1_HG.SG, USA_Ancient_Beringian.SG, USA_Anzick.SG, Mixe.DG, Mexico_Zapotec.DG, Belize_MayahakCabPek_9300BP, Karitiana.DG as a mixture of Yoruba.DG, Spanish.DG and ‘Mayan’, the working model (*P* = 0.11) suggests a composition of 92% Mayan, 7% Spanish and 0.03% Yoruban ancestry.

### Reporting summary

Further information on research design is available in the Nature Portfolio Reporting Summary linked to this article.

## Online content

Any methods, additional references, Nature Portfolio reporting summaries, source data, extended data, supplementary information, acknowledgements, peer review information; details of author contributions and competing interests; and statements of data and code availability are available at 10.1038/s41586-024-07509-7.

### Supplementary information

Supplementary Information Supplementary text, methods, Tables 1–20 and Figs. 1–8.

Reporting Summary

Supplementary Note 1 The non-peer-reviewed translation of the main article into Spanish.

## Data Availability

All the genomic data (including nuclear DNA, mtDNA and HLA alignment sequences) for the ancient Chichén Itzá individuals (YCH) are archived in the European Nucleotide Archive database (accession no. PRJEB73567). Present-day genomic data of Tixcacaltuyub individuals (TIX) are archived in the European Genome-Phenome Archive database (dataset no. EGAD50000000426) and will be made available on request from R.B., K.N., J.C.L.R. and J.K. and subject to a signed agreement to restrict usage to anonymized studies of population history. All HLA data from our sample sets, both frequencies and individual genotypes, can be found at The Allele Frequency Net Database website (www.allelefrequencies.net) under accession nos. 3791 (YCH) and 3790 (TIX).
