# Supplementary material for: Ancient genomes reveal insights into ritual life at Chichén Itzá
Source: Nature. 2024 Jun 12;630(8018):912–9. doi: 10.1038/s41586-024-07509-7 (PMC11208145; doi:10.1038/s41586-024-07509-7)
Supplement: Supplementary file 1 — Supplementary text, methods, Tables 1–20 and Figs. 1–8. [file 41586_2024_7509_MOESM1_ESM.pdf]

nature portfolio

https://doi.org/10.1038/s41586-024-07509-7

Supplementary information

Ancient genomes reveal insights into ritual life at Chichén Itzá

In the format provided by the authors and unedited

Ancient genomes reveal insights into ritual life at Chichén Itzá

Rodrigo Barquera, Oana del Castillo-Chávez, Kathrin Nägele, Patxi Pérez-Ramallo, Diana Iraíz Hernández-Zaragoza, András Szolek, Adam Benjamin Rohrlach, Pablo Librado, Ainash Childebayeva, Raffaela Angelina Bianco, Bridget Penman, Victor Acuña-Alonzo, Mary Lucas, Julio César Lara-Riegos, María Ermila Moo-Mezeta, Julio César Torres-Romero, Patrick Roberts, Oliver Kohlbacher, Christina Warinner, Johannes Krause.

Supplementary Material

Supplementary text: Archaeological context of Chichén Itzá......3

Supplementary methods: Stable isotopes analyses.......4

Table S1. Collagen $\delta^{15}N$ (‰) and $\delta^{13}C$ (‰) data and radiocarbon determinations calibrated using OxCal. v4.4.4 and the IntCal20 atmospheric curve[43].......7

Table S2. Collagen $\delta^{15}N$ and $\delta^{13}C$ mean values, SD, and number of samples for Late and Terminal Classic Maya individuals, excluding those potentially influenced by breastfeeding......9

Figure S1. Radiocarbon dating results calibrated using OxCal. v4.4.4[62], and the IntCal20 atmospheric curve[43].......11

Supplementary methods: Genetic pairwise mismatch rate and genetic kin relationships.......12

Figure S2. Genetic pairwise mismatch rate (PMR) for child pairs......12

Figure S3. Enhanced view of human bone collagen isotopic $\delta^{13}C$ and $\delta^{15}N$ values from the present study.......13

Figure S4. Collagen $\delta^{13}C$ and $\delta^{15}N$ mean values and SD for Late and Terminal Classic Maya from the present study and other Maya populations.......13

Figure S5. Difference between $\delta^{15}N$ and $\delta^{13}C$ values for the related and unrelated individuals found in the chultún.......14

Supplementary methods: Preparation of single stranded libraries.......15

Supplementary methods: Genetic continuity testing......16

Figure S6. The diversity of uniparental markers in ancient Mayans from Chichén Itzá (YCH) and present-day Mayans from Tixcacaltuyub (TIX).......17

Table S3. Top lipid metabolism-associated genes for YCH.......18

Table S4. Top lipid metabolism-associated genes for TIX.......23

Table S5. Top lipid metabolism-associated genes comparison for YCH and TIX.......26

Table S6. GoWinda enrichment analysis results for YCH.......27

Table S7. GoWinda enrichment analysis results for TIX.......49

Table S8: SNP positions enriched in Chichén Itzá, Tixcacaltuyub and the SNPs reported by Lindo et al., 2016[17] for the HLA region. 53

Table S9. HLA, mtDNA and Y-Chr genotypes for YCH. 54

Table S10. HLA, mtDNA and Y-Chr genotypes for TIX. 57

Table S11. Frequencies of HLA-A alleles in Chichén Itzá and Tixcacaltuyub. 61

Table S12. Frequencies of HLA-B alleles in Chichén Itzá and Tixcacaltuyub. 62

Table S13. Frequencies of HLA-C alleles in Chichén Itzá and Tixcacaltuyub. 63

Table S14. Frequencies of HLA-DRB1 alleles in Chichén Itzá and Tixcacaltuyub. 64

Table S15. Frequencies of HLA-DRB3/4/5 alleles in Chichén Itzá and Tixcacaltuyub. 65

Table S16. Frequencies of HLA-DQA1 alleles in Chichén Itzá and Tixcacaltuyub. 65

Table S17. Frequencies of HLA-DQB1 alleles in Chichén Itzá and Tixcacaltuyub. 66

Table S18. Frequencies of HLA-DPA1 alleles in Chichén Itzá and Tixcacaltuyub. 66

Table S19. Frequencies of HLA-DPB1 alleles in Chichén Itzá and Tixcacaltuyub. 67

Supplementary methods: Non-overlapping HLA associations. 68

Figure S7. Frequencies of HLA-B and HLA-C associations in the ancient (YCH) and modern (TIX) populations. 68

Figure S8. Frequencies of HLA-B and HLA-DRB1 and HLA-B and DRB3/4/5 associations in the ancient (YCH) and present day (TIX) populations. 69

Supplementary methods: In-silico binding prediction assays. 70

Table S20. Binding prediction results for the Salmonella enterica peptides presented by HLA class II molecules. 71

Supplementary Information: source populations for the population genetics analyses 72

Supplementary methods: Community engagement activities. 77

Supplementary references. 79

Supplementary text: Archaeological context of Chichén Itzá.

Contact researchers: Rodrigo Barquera, Oana del Castillo-Chávez, Kathrin Nägele, Patxi Pérez-Ramallo, Diana Iraíz Hernández-Zaragoza, and Christina Warinner.

Chichén Itzá became the dominant political centre of the northern Maya lowlands during the Terminal Classic (AD 800-1000), a period characterized by long-distance political and social connections – first to the Puuc Maya in the western Yucatan and later to the city of Tula in Central Mexico; the latter being associated with an ambitious new building program in an architectural style variously described as "International" or "Toltec"[1,2]. *El Castillo*, also known as the Temple of Kukulkán because of the elaborately carved feathered serpent columns adorning its façade, the massive pyramidal structure was constructed in a foreign Central Mexican style honouring the mythical ruler or deity Quetzalcoatl (known as K'uk'ulkan in Mayan), and other distinctive architecture from this period such as colonnades, a large ballcourt and structures explicitly associated with ritual sacrifice[3,4], including a *tzompantli* (skull rack) and anthropomorphic stone sculptures known as *chacmools*.

Evidence of ritual killing is extensive throughout the site of Chichén Itzá, and includes both the physical remains of sacrificed individuals, as well as representations in monumental art[5]. Elite activity at Chichén Itzá declined during the 11th century AD, with a last inscribed calendar date of AD 998[6,7], but the site continued to be a prominent ritual and pilgrimage centre through the colonial period and beyond[8–10]. Male adult crania obtained through ritual violence have been found adorning buildings at Chichén Itzá's Las Monjas complex and along the platform of the Caracol structure, as well as at later sites under Itzá control, such as Ixlú, where shared dental traits suggest at least some of these males were related[11].

Situated on a karst plain in one of the most densely settled regions of the Maya lowlands[12], Chichén Itzá enjoyed ready access to fertile agricultural lands and the resource-rich coastal zone[7], as well as abundant water from its extensive system of *cenotes* (sinkholes), *aguadas* (rainwater reservoirs), and human-made *chultunes* (underground cisterns)[1,13,14]. Throughout Mesoamerica, caves, cenotes, and chultunes have long been associated with water, rain, and child sacrifice[15–17], and such subterranean features are widely viewed as access points to the Maya underworld[18,19]. The chultún analysed in the present work was discovered adjacent to a small structure, perhaps an altar, when a local airstrip was built in the 1960s. Over one hundred human individuals were excavated from the chultún, which was constructed as an artificial cave in the shape of a bottle[15,20].

Supplementary methods: Stable isotopes analyses.

Contact researchers: Patxi Pérez-Ramallo, Adam Benjamin Rohrlach, Mary Lucas, Christina Warinner and Patrick Roberts.

The reconstruction of past human and faunal diets using stale carbon ($\delta^{13}C$) and nitrogen ($\delta^{15}N$) isotope analysis is common and well-established in multidisciplinary archaeological research[21], with its application extending back to the 1970s[22,23]. Direct insights into dietary trends among past populations enables the investigation of connections between diet and social status, cultural customs linked to food, environmental impacts on subsistence, and individual mobility during the course of their life history[24,25]. Bone collagen $\delta^{13}C$ and $\delta^{15}N$ measurements primarily reflect the protein component of an individual's diet during the period of tissue formation and, to a lesser extent, the lipid and carbohydrate sources[26].

The $\delta^{13}C$ variability in terrestrial ecosystems is driven by differences in the two dominant photosynthetic pathways with regards to their discrimination against ^{13}C during CO_2 fixation[27]. C_3 plants (e.g., trees, shrubs, or wheat) have lower $\delta^{13}C$ values ranging from c. -24 to -36‰ (global mean -26.5‰). By contrast, C_4 plants (e.g., maize, millet, sugar cane, or sorghums) have higher $\delta^{13}C$ values, oscillating between c. -9 to -17‰ (global mean -12‰)[27–29]. The $\delta^{13}C$ values of marine plants, which draw CO_2 from a different source, sit towards the range of C_4 plants, while crassulacean acid metabolism (CAM) plants (e.g., succulents) occupy an intermediate position[30,31]. These distinctions are tracked into the tissues of consumers of these plants with an enrichment in $\delta^{13}C$ of approximately 5‰ between dietary plants and consumers, 1-2‰ in subsequent trophic level steps among omnivores and carnivores[21].[13]

Trophic level plays a major role in the variation of $\delta^{15}N$ values measured for animals in both terrestrial and aquatic ecosystems. $\delta^{15}N$ increase of between +3-6‰ from plants to herbivores, and from herbivores to carnivores have been observed in a variety of different ecological settings[30,32]. This trophic effect is most likely linked to the disproportionate loss of ^{15}N-depleted excretion products at each stage in the foodchain[33], although diet-tissue distinctions are highly variable between animals[34]. Marine consumers are, on average, higher in $\delta^{15}N$ compared with terrestrial consumers as a result of longer foodchains and different sources of nitrogen. Meanwhile freshwater consumers often demonstrate higher $\delta^{15}N$ although $\delta^{13}C$ is far more variable[35,36].

Here, we analysed bone collagen from the temporal and petrous bone used to conduct the radiocarbon dating and aDNA analysis on individuals sampled in this study. The samples analysed consisted of approximately 1 g of bone. Collagen was then extracted using a standard procedure[37]. The bone fragments were demineralized in 10 mL aliquots of 0.5M HCl at 4°C. The acid was changed until CO_2 stopped evolving. The residue was rinsed three times in deionized water before being gelatinized in pH 3 HCl at 75°C for 48 hours. The resulting solution was filtered, with the supernatant then being lyophilised over a period of 24 hours. After calculating the collagen yield for each sample, 0.5 mg of purified collagen sample were weighed into tin capsules to be analysed in duplicate, using a Thermo Fisher Elemental

Analyzer (Thermo Fisher Scientific Inc., Waltham, Massachusetts, U.S.) coupled to a Thermo Fisher Delta V Advantage Mass Spectrometer (Thermo Fisher Scientific Inc., Waltham, Massachusetts, U.S.) via a ConFlo IV system (Thermo Fisher Scientific Inc., Waltham, Massachusetts, U.S.) at the Max Planck Institute of Geoanthropology (formerly for the Science of Human History), Jena, Germany.

Isotopic values are reported as the ratio of the heavier isotope to the lighter isotope ($^{13}C/^{12}C$ or $^{15}N/^{14}N$) as δ values in parts per mill (‰) relative to international standards, Vienna Pee Dee Belemnite (VPDB) for $\delta^{13}C$ and atmospheric N_2 (AIR) for $\delta^{15}N$. Results were corrected using a two-point calibration against international standards (IAEA-CH-6 Sucrose, IAEA-N-2 Ammonium Sulphate and USGS40 L-Glutamic Acid); USGS40 $^{13}C_{raw}$ = -26.4 ± 0.1, $^{13}C_{true}$ = -26.4 ± 0.0, $^{15}N_{raw}$ = -4.4 ± 0.1, $^{15}N_{true}$ = -4.5 ± 0.2; IAEA N2 $^{15}N_{raw}$ = 20.2 ± 0.1, $^{15}N_{true}$ = 20.3 ± 0.2; IAEA C6 $^{13}C_{raw}$ = -10.9 ± 0.1, $^{13}C_{true}$ = -10.8 ± 0.0. Replicate analyses of standards suggest that machine measurement error is c. ± 0.2‰ for $\delta^{13}C$ and ± 0.1‰ for $\delta^{15}N$. Overall measurement precision was studied through the measurement of repeat extracts from a fish gelatine standard (n=20, ± 0.1‰ for $\delta^{13}C$ and ± 0.1‰ for $\delta^{15}N$).

The bioarchaeological results obtained from the human sacrifices discovered in the Midnight Terror cave in Belize[38] suggest that the Classic Maya preferred to select outsiders for sacrifice rather than locals. The observed standard deviation of the $\delta^{15}N$ values (SD=1.5) obtained from the individuals analysed at Chichén Itzá (Fig. S3) is the highest of all the Late Classic and Terminal Classic Maya sites analysed to date (see Table S1 and Fig. S4). The overall picture from the reconstruction of palaeodiet in this study reveals the consumption of significant amounts of maize, but with geographic variations reflected in microenvironmental differences in available foods and variability in trade networks[39,40]. Perhaps, as in the case of the Midnight Terror Cave, most of the individuals sacrificed at Chichén Itzá were non-local Maya. On the other hand, other studies have shown that the diet of the Classic Maya elite tends to be more variable than that of the general population over time[41], reflected also in $\delta^{13}C$ and $\delta^{15}N$ standard deviations observed elsewhere (e.g., Altun Ha or Baking Pot; see Table S1 and Fig. S4). Therefore, the differences in protein intake observed in the Chichén Itzá individuals studied could also indicate variations in social status.

Randomisation tests for isotope values for related vs. unrelated individuals

To test the difference between $\delta^{15}N$ and $\delta^{13}C$ values for the related and unrelated individuals, we began by calculating the pairwise differences in the values for all pairs, denoted n_{ij} and c_{ij}, respectively. We then calculated the observed W-statistic from a Wilcoxon Rank Sum test, denoted W_N and W_C. Due to the repeated measures inherent in comparing pairwise values, the assumption of independence between samples was not satisfied. Hence, we performed randomisation tests by randomly reassigning the *related* and *unrelated labels,* and reperforming and recalculating the W-statistic (for 100,000 replications, seed=12345). We then calculated a two-sided empirical p-value[42] for both W_N and W_C.

We found that both of the average δ^{15}N and δ^{13}C values were significantly lower for the related group when compared to the unrelated group ($p = 0.00439$ and $p = 0.00212$, respectively) indicating that related individuals share more similar δ^{15}N and δ^{13}C values than would be expected by random chance.

Table S1. Collagen $\delta^{15}N$ (‰) and $\delta^{13}C$ (‰) data and radiocarbon determinations calibrated using OxCal. v4.4.4 and the IntCal20 atmospheric curve[43].

Sample name	Radiocarbon years BP	Calibrated calendar date (AD, 95.4%)	MAMS reference	$\delta^{15}N$ (‰) (Air)	$\delta^{13}C$ (‰) (VPDB)	%N	%C	C/N ratio
YCH063	1437±17	595-650	35832	9.6	-10.2	13.10	36.12	3.2
YCH037	1289±22	665-775	35824	9.7	-8.9	13.49	37.15	3.2
YCH007	1228±22	700-885	35811	5.9	-9.0	14.66	42.70	3.4
YCH036	1224±22	700-885	35823	11.8	-9.1	15.36	42.33	3.2
YCH060	1210±17	770-885	35831	8.2	-9.0	15.16	41.29	3.2
YCH056	1198±23	770-895	35829	10.4	-13.4	15.13	43.90	3.4
YCH018	1188±24	770-945	35816	8.4	-9.9	15.09	42.68	3.3
YCH030	1178±22	770-950	35820	8.3	-8.9	15.63	43.21	3.2
YCH003	1177±24	770-950	35810	8.5	-9.7	12.67	35.58	3.3
YCH034	1157±23	770-980	35822	9.6	-9.2	15.54	42.91	3.2
YCH024	1157±21	770-980	35818	10.1	-13.3	14.92	41.93	3.3
YCH001	1156±22	770-980	35808	-	-10.5	-	-	3.2
YCH002	1154±21	770-980	35809	8.1	-10.4	14.91	43.09	3.4
YCH048	1144±23	770-990	35826	10.9	-9.1	15.60	43.06	3.2
YCH033	1100±22	890-995	35821	10.0	-8.1	13.32	38.80	3.4
YCH011	1098±21	890-995	35813	10.5	-8.1	14.26	40.90	3.3
YCH054	1091±23	890-1020	35828	10.1	-7.6	14.45	41.70	3.4
YCH016	1083±23	890-1025	35815	9.9	-9.5	14.57	40.73	3.3
YCH020	1070±24	895-1025	35817	9.1	-11.2	15.44	42.29	3.2
YCH040	1065±23	895-1025	35825	8.2	-10.3	13.66	40.01	3.4
YCH058	1060±22	995-1030	35830	11.2	-9.5	15.05	42.00	3.3
YCH012	1058±23	895-1030	35814	8.4	-9.3	15.94	43.28	3.2
YCH010	1054±23	895-1030	35812	11.3	-8.4	14.42	41.23	3.3
YCH027	1044±21	975-1035	35819	9.2	-8.5	16.26	44.16	3.2
YCH051	970±17	1025-1155	56233	8.2	-9.3	15.72	43.54	3.2
YCH004	-	-	-	12.1	-11.5	15.67	43.41	3.2
YCH005	-	-	-	8.6	-9.8	13.22	38.58	3.4
YCH006	-	-	-	8.4	-9.1	15.70	43.14	3.2
YCH008	-	-	-	12.1	-10.9	13.78	39.40	3.3
YCH009	-	-	-	8.9	-10.7	14.91	40.79	3.2
YCH015	-	-	-	8.5	-7.9	14.12	40.18	3.3
YCH017	-	-	-	9.6	-9.2	9.97	28.90	3.4
YCH022	-	-	-	9.9	-13.0	6.69	20.84	3.6
YCH023	-	-	-	12.2	-11.2	12.61	38.64	3.6
YCH025	-	-	-	10.4	-8.0	11.43	31.29	3.2
YCH029	-	-	-	10.6	-9.5	13.88	38.69	3.3
YCH031	-	-	-	9.4	-8.2	15.87	44.58	3.3

Sample name	Radiocarbon years BP	Calibrated calendar date (AD, 95.4%)	MAMS reference	$\delta^{15}N$ (‰) (Air)	$\delta^{13}C$ (‰) (VPDB)	%N	%C	C/N ratio
YCH032	-	-	-	14.0	-10.5	15.16	42.85	3.3
YCH038	-	-	-	10.6	-10.5	8.37	23.81	3.3
YCH039	-	-	-	11.7	-11.9	14.01	38.35	3.2
YCH041	-	-	-	9.5	-9.4	13.68	38.38	3.3
YCH042	-	-	-	8.8	-8.5	12.04	35.85	3.5
YCH043	-	-	-	8.4	-8.8	14.31	41.14	3.4
YCH045	-	-	-	11.4	-8.9	14.54	40.23	3.2
YCH046	-	-	-	8.6	-13.9	13.79	40.47	3.4
YCH047	-	-	-	11.0	-12.1	9.99	28.61	3.3
YCH049	-	-	-	8.0	-9.1	13.15	36.48	3.2
YCH050	-	-	-	9.8	-10.9	13.69	40.09	3.4
YCH053	-	-	-	8.6	-10.5	10.75	29.84	3.2
YCH055	-	-	-	9.3	-7.6	15.44	43.48	3.3
YCH059	-	-	-	8.4	-10.2	10.56	29.84	3.3
YCH061	-	-	-	11.1	-11.6	12.51	34.43	3.2
YCH062	-	-	-	11.1	-10.8	5.97	17.37	3.4
YCH064	-	-	-	9.8	-9.3	12.97	36.17	3.3

MAMS: Radiocarbon Lab, CEZA, Mannheim, Germany laboratory number; AMS: accelerator mass spectrometry. BP: before present.

Table S2. Collagen δ^{15}N and δ^{13}C mean values, SD, and number of samples for Late and Terminal Classic Maya individuals, excluding those potentially influenced by breastfeeding. Original citations to published studies are provided in the column "Reference".

Site	n	δ^{13}C (‰) (VPDB)	SD	n	δ^{15}N (‰) (Air)	SD	Reference
Chichén Itzá	53	-9.9	1.5	53	9.7	1.5	Present Study
Aguateca	5	-9.9	0.7	4	9.1	0.9	44
Altar de Sacrificios	22	-8.9	1.1	22	8.8	1.1	44
Altun Ha	47	-11.9	2.0	47	10.4	0.6	45
Baking Pot	9	-11.0	1.1	9	9.2	1.3	46
Barton Ramie	31	-11.2	1.2	31	8.9	0.4	46
Cahal Pech	9	-10.0	1.7	9	9.5	0.8	47–49
Caledonia	22	-9.9	1.9	22	9.1	1.1	50
Caracol	14	-9.6	1.3	14	9.3	1.1	49
Chac Balam	10	-8.5	1.5	10	11.5	0.7	51
Chau Hiix	18	-12.0	1.9	18	10.9	0.8	52
Chinikihá	7	-9.9	0.4	7	9.1	0.8	53
Copán	16	-10.0	1.0	15	7.6	0.7	46
Copán Valley	23	-10.3	0.8	22	7.5	0.8	46
Dos Pilas	19	-9.1	1.0	19	9.6	1.1	44
Holmul	2	-9.0	0.0	2	9.1	0.4	46
Itzán	4	-9.2	0.4	4	8.1	1.0	44
Lamanai	10	-14.8	1.1	8	10.0	0.4	54
Lower Dover	1	-11.9	-	1	10.6	-	49
Marco González	31	-7.9	1.3	31	10.2	1.1	55
Mayapán	68	-9.8	0.7	68	8.6	0.5	56,57
Minanha	24	-10.9	1.7	24	9.0	0.8	58
Pacbitun	19	-10.3	1.6	19	9.3	0.7	49,59
Peligroso (Chalillo)	1	-10.4	-	1	10.4	-	49
Piedras Negras	35	-9.2	0.8	35	8.9	0.9	60
Pook's Hill	6	-11.4	0.7	6	8.4	0.3	49
Ramonal (Chalillo)	1	-11.1	-	1	8.9	-	49
San Juan	3	-8.6	0.2	4	11.1	1.0	51
San Lorenzo	1	-12.2	-	1	9.5	-	49

Site	n	$\delta^{13}C$ (‰) (VPDB)	SD	n	$\delta^{15}N$ (‰) (Air)	SD	Reference
San Pedro	20	-6.8	1.1	20	9.7	0.5	[55]
Seibal	26	-9.6	1.4	26	9.3	1.0	[46]
Uaxactún	5	-10.3	0.9	5	9.4	1.0	[46]
Xunantunich	6	-10.8	1.8	6	9.7	0.9	[49,50]
Yaxuná	2	-12.4	0.8	2	7.0	0.5	[61]

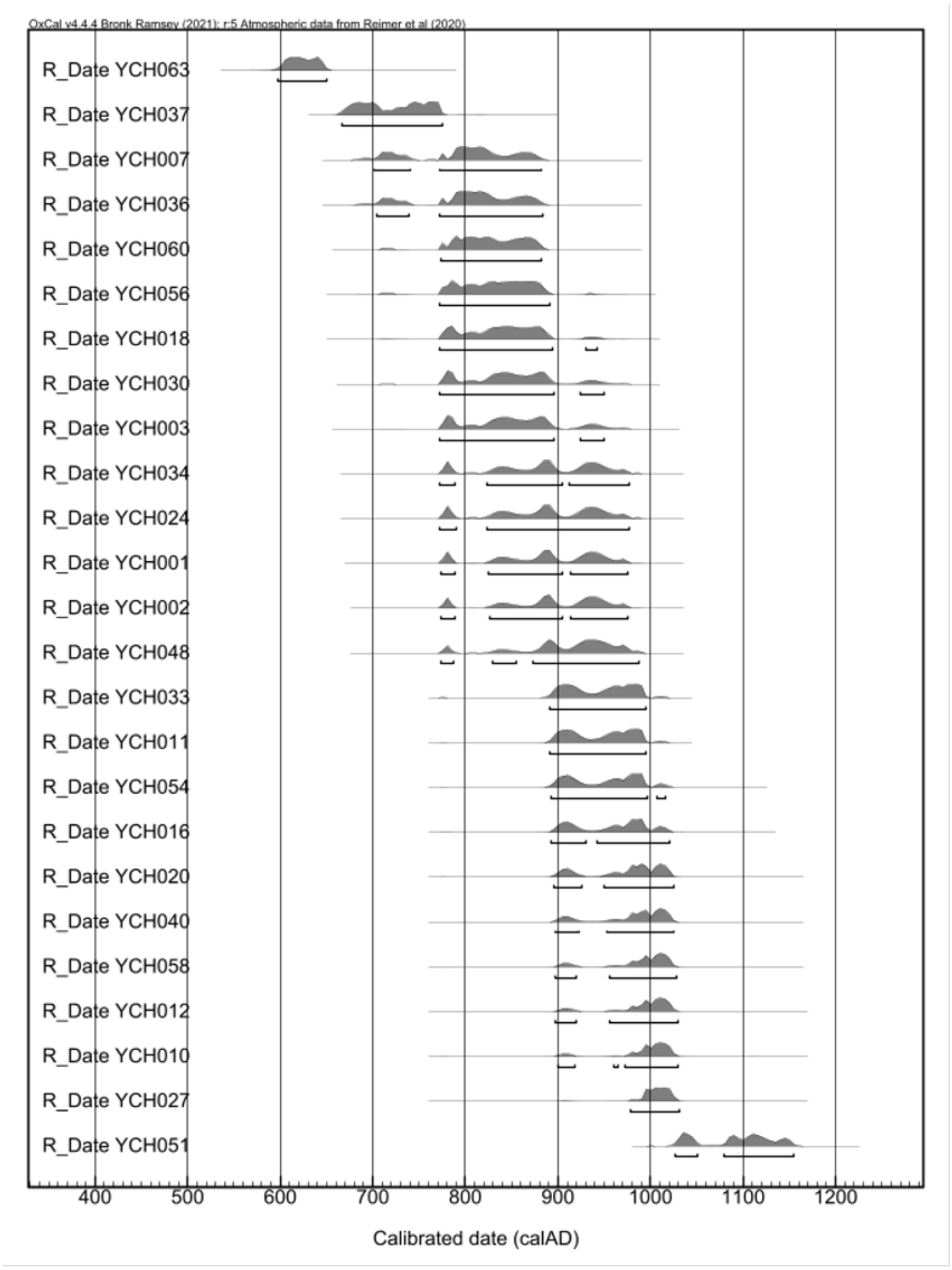

Figure S1. Radiocarbon dating results calibrated using OxCal. v4.4.4[62], and the IntCal20 atmospheric curve[43].

Supplementary methods: Genetic pairwise mismatch rate and genetic kin relationships.

Contact researchers: Rodrigo Barquera, Kathrin Nägele, Adam Benjamin Rohrlach and Christina Warinner.

We employed a method for statistically testing the genetic relatedness between individuals called BREADR (v.1.0.1) to estimating the pairwise-mismatch rate PMR for a pair of individuals based on the assumption of a binomial distribution for the PMR, for pseudo-haploid data, available. Here, for individuals *i* and *j*, we thinned the data such that all sites were at least 200K bases apart to best satisfy the assumption of independence. We also used the median PMR as a baseline for the expected PMR of two unrelated individuals. For a full description of the method, see Rohrlach et al., 2023[63].

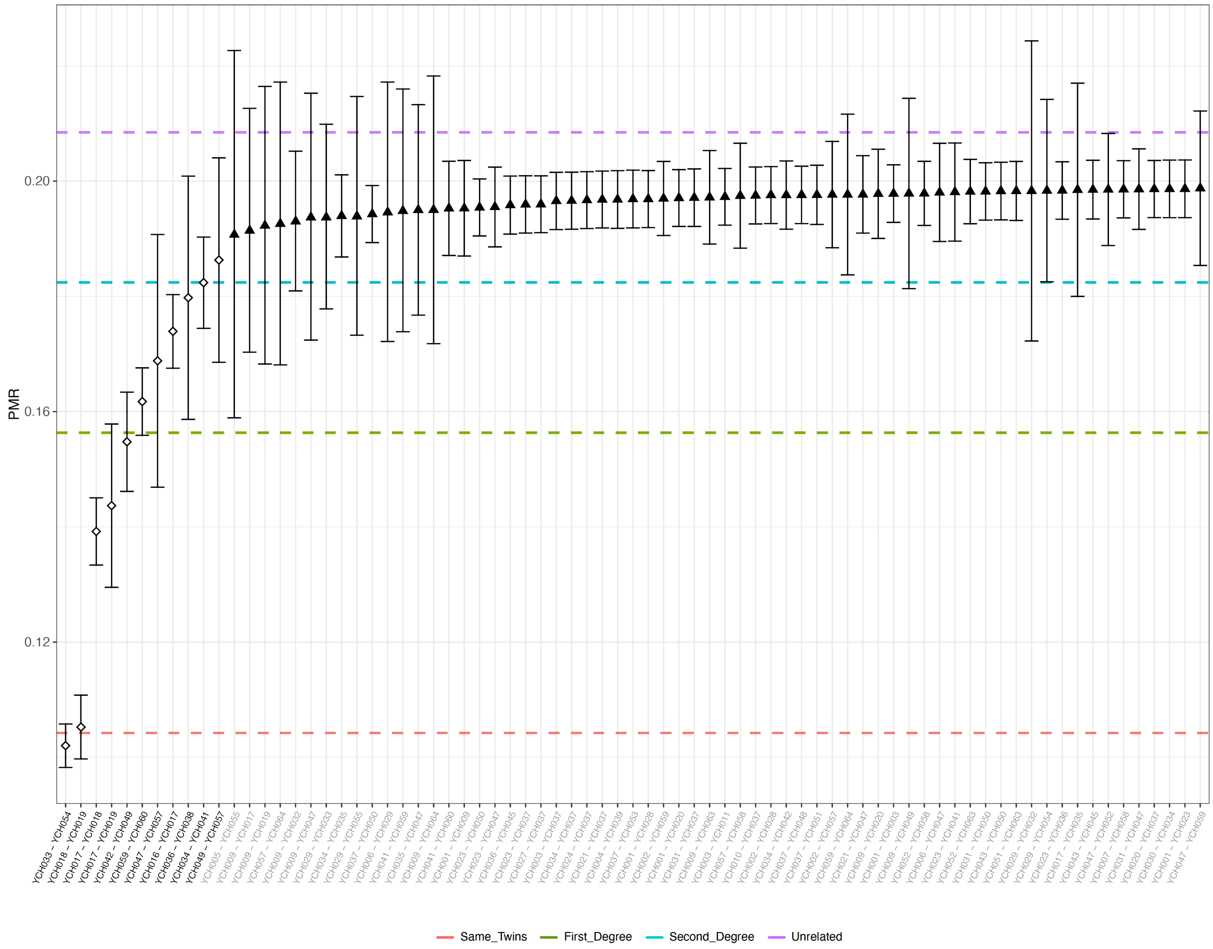

Figure S2. Genetic pairwise mismatch rate (PMR) for child pairs within the chultún identifies eleven close relative pairs (hollow diamonds, black text), including two pairs of monozygotic twins. A low overall PMR for unrelated individuals (black triangles, grey text) confirms low genetic diversity in the population; only pairs with a PMR significantly <0.20 are visualized in the plot.

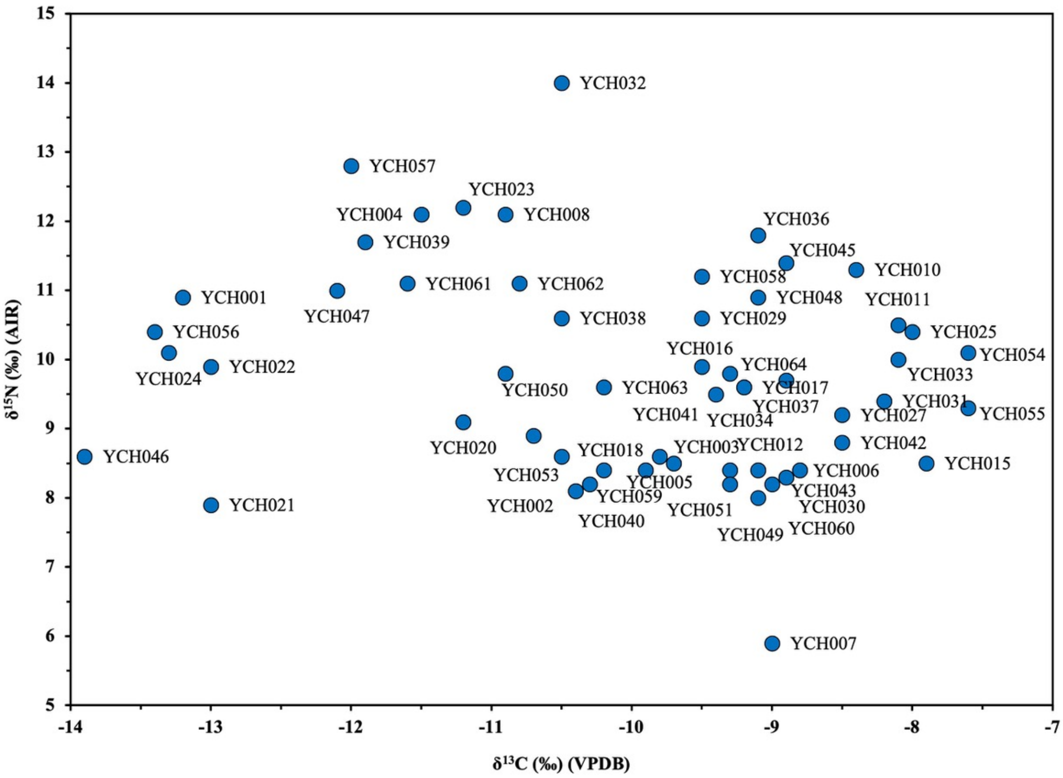

Figure S3. Enhanced view of human bone collagen isotopic $\delta^{13}C$ and $\delta^{15}N$ values from the present study.

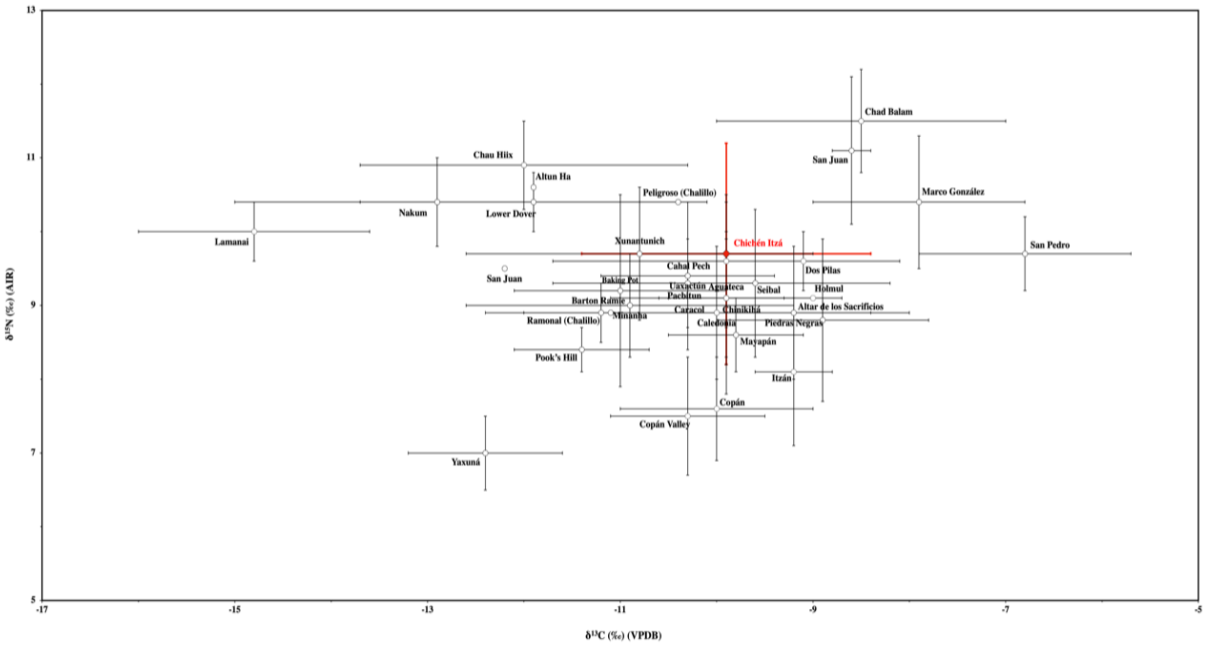

Figure S4. Collagen $\delta^{13}C$ and $\delta^{15}N$ mean values and SD for Late and Terminal Classic Maya from the present study and other Maya populations. See Table S2 for citations.

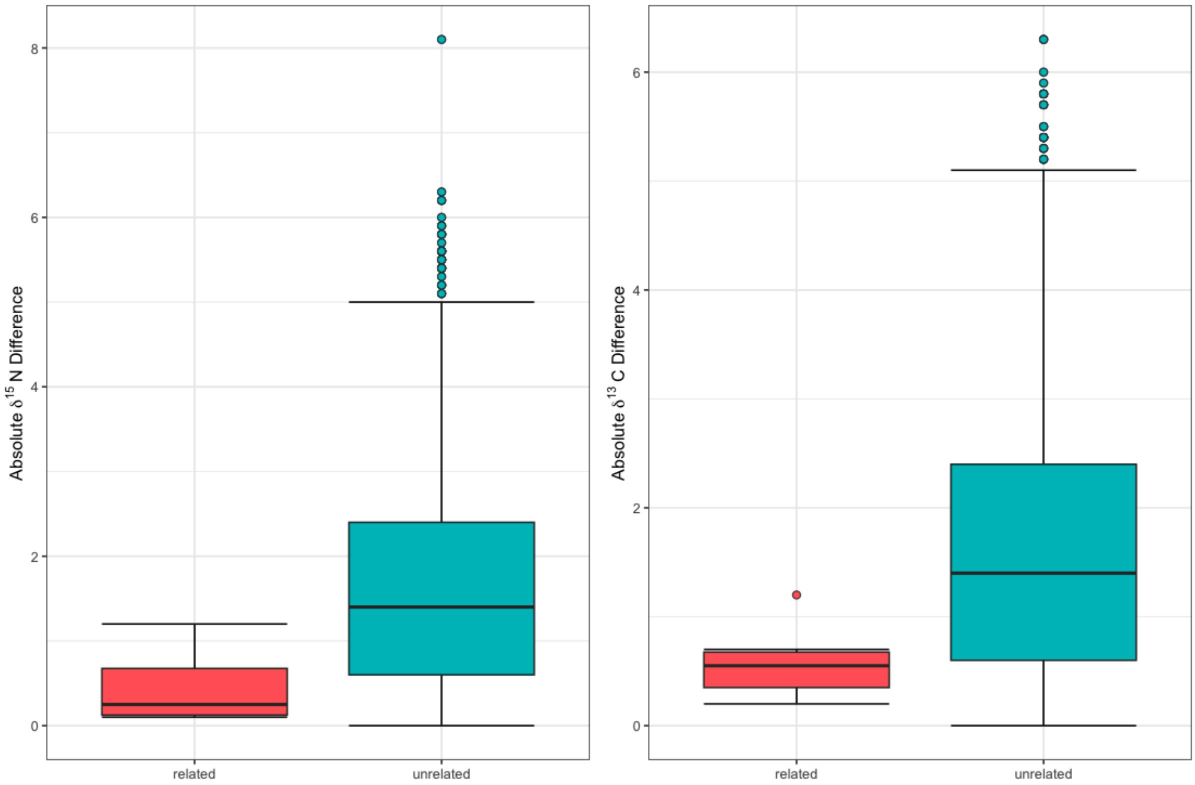

Figure S5. Difference between $\delta^{15}N$ and $\delta^{13}C$ values for the related and unrelated individuals found in the chultún.

Supplementary methods: Preparation of single stranded libraries.

Contact researchers: Rodrigo Barquera & Raffaela Angelina Bianco.

We built single-stranded, UDG-half treated libraries using a protocol implemented for the automated library preparation[64,65] on the Bravo-B NGS Workstation (Agilent Technologies, Inc., Santa Clara CA, USA) for the extracts for which not enough data could be obtained from the double-stranded, UDG-half treated libraries. Briefly, DNA fragments present in the extract are dephosphorylated at the 5′ and 3′ ends and separated into single strands by heat denaturation. 3′-biotinylated adapter molecules are attached to the 3′ ends of the DNA fragments using T4 DNA ligase and a splinter oligonucleotide carrying a stretch of six random nucleotides (marked as "N"). Following the immobilization of the ligation products on streptavidin-coated beads, the splinter oligonucleotide is removed by a bead wash at elevated temperature. Synthesis of the second strand is carried out using the Klenow fragment of *Escherichia coli* DNA polymerase I. Not incorporated primers are removed through a bead wash at elevated temperature. Following the blunt-end ligation of the second adapter, the final synthesised strand is released from the beads by heat denaturation.

Supplementary methods: Genetic continuity testing.

Contact researchers: Rodrigo Barquera, Pablo Librado & Adam Benjamin Rohrlach.

In order to formally assess whether TIX descends from CHI, we employed the direct ancestry test developed by J. Schraiber[66]. To prepare the input file as required, we began by mapping the sequence data from the YCH and TIX individuals to the *Pan troglodytes* reference genome (release: GCF_028858775.1-RS_2023_03)[67], to polarise the alleles as ancestral or derived. We then calculated the allele frequencies for chromosomes 1-22 for the TIX individuals, and the allele counts for the YCH and TIX individuals, using angsd[68] with -minQ 20. We then took the set of all 664,145,998 genomic sites covered by the YCH and TIX individuals and filtered these such that the site was covered by at least two individuals from YCH, were covered by at least 50 from TIX, and such that the site was not at fixation for TIX. This yielded a total of 23,904,864 sites which were analysed for continuity, 930,464 of which representing nucleotide transversions that segregated at MAF > 1%. Using this subset of genomic sites, we then produced genotype data using Plink[69] on the filtered genomic sites from above. The second step focused then on the ancient YCH specimens, to compute the number of reads that support each allele, at these specific 930,464 positions (-doCounts 1 and -dumpCounts 4). These read counts were subsequently categorized, for each SNP and YCH individual, as derived, ancestral or other allele. All SNPs showing an abnormal sequencing depth were filtered out (95% confidence interval), following the recommendation of the developer (possibly representing hidden structural variation). This last filter yielded a final panel of 883,941 high-quality nucleotide transversions, a SNP set representing an excellent trade-off between SNP quality and density, especially when considering that the method assumes linkage equilibrium and pruning is recommended. Using in-house scripts, the results from both steps were finally merged, and formatted according to the specifications.

Based on this input file, we next contrasted two competing population models: the null hypothesis assumes that YCH and TIX are the same population evolving over time (direct ancestry model), while the alternative one posits that YCH was instead sister to the true ancestral population (ancient but not ancestral model). The likelihood of both models and the underlying parameters were found indistinguishable, for each individual, and consequently the likelihood ratio tests returned *p*-values close to one. The null hypothesis of genetic continuity, therefore, cannot be rejected. Given the high sensitivity of this test, these results establish that TIX unequivocally descends from YCH.

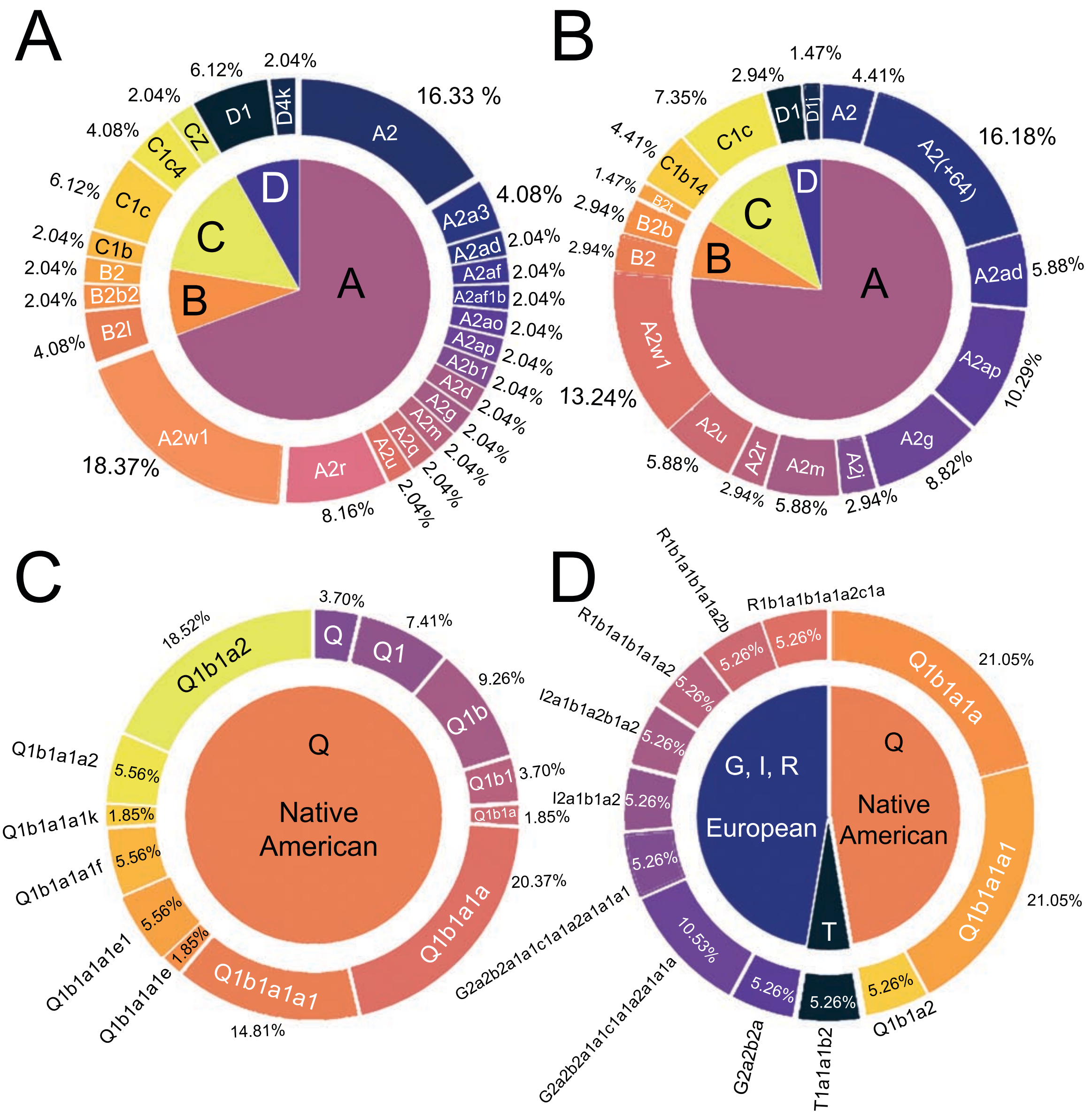

Figure S6. The diversity of uniparental markers in ancient Mayans from Chichén Itzá (YCH) and present-day Mayans from Tixcacaltuyub (TIX). **A.** Mitochondrial DNA (mtDNA) diversity in YCH. Inner circle represents the proportions of each of the main haplogroups found in the YCH group (N=64). Outer circle represents the high resolution mtDNA haplogroup diversity found in this group. **B.** The diversity of mtDNA lineages in TIX. Inner circle represents the proportions of each of the main haplogroups found in the TIX group (N=68). Outer circle represents the high resolution mtDNA haplogroup diversity found in this group. **C.** Y chromosome (Y-Chr) diversity in YCH. Inner circle represents the family of Y-Chr lineages found in the YCH group (N=64; they all belonged to haplotype Q). Outer circle represents the highest resolution available for the Y-Chr diversity found in this group. **D.** The diversity of Y-Chr haplogroups in TIX. Inner circle represents the proportions of each of the main continental origin for the Y-Chr haplotype lineages found in the TIX group (N=19). Outer circle represents the highest resolution available for the Y-Chr diversity found in this group. For the individual mtDNA and Y-Chr genotypes, please refer to Tables S9 & S10.

Table S3. Top lipid metabolism-associated genes for YCH.

SNP	Chr	Gene	Gene symbol	Phenotype	Assoc. genes	Clinical Significance
**rs988832**	1	formin 2	FMN2	Body Mass Index	FMN2	
**rs7542755**	1	NULL	NULL	Body Mass Index, Lithium clearance adjusted for clinical predictors	COX6A1P1, RPL7P10	
**rs1805152**	1	chloride voltage-gated channel Ka	CLCNKA	Cholesterol, ClinVar: phenotype not specified, Bartter disease type 4B	CLCNKA, LOC106501712, CLCNKA	benign
**rs7517847**	1	interleukin 23 receptor	IL23R	Inflammatory Bowel Diseases, Plasma omega-6 polyunsaturated fatty acid levels (linoleic acid), Crohn's disease vs rheumatoid arthritis ordinary least squares OLS, Crohn's disease, Chronic inflammatory diseases (ankylosing spondylitis Crohn's disease psoriasis primary sclerosing cholangitis ulcerative colitis) (pleiotropy)	IL23R	
**rs10925809**	1	NULL	NULL	Lipoproteins	RPL39P10, CHRM3	
**rs17321999**	2	LBH regulator of WNT signaling pathway	LBH	Apolipoprotein A1 levels, Systemic lupus erythematosus	LBH	
**rs1864430**	2	interferon induced with helicase C domain 1	IFIH1	Cholesterol	IFIH1	
**rs10490002**	2	WD repeat, sterile alpha motif and U-box domain containing 1	WDSUB1	Cholesterol HDL	WDSUB1	
**rs2118507**	2	NULL	NULL	Cholesterol LDL	BIN1, CYP27C1	
**rs6706926**	2	fibroblast activation protein alpha	FAP	Cholesterol, Echocardiography	FAP	
**rs2338545**	2	phospholipase B1	PLB1	Diabetes mellitus type 2	PLB1	
**rs4404290**	2	NULL	NULL	Triglycerides	GLULP6, SLC39A10	
**rs10199914**	2	NULL	NULL	Triglycerides	intergenic	
**rs1371555**	2	NULL	NULL	Triglycerides	GLULP6, SLC39A10	
**rs2419407**	2	NULL	NULL	Waist-hip ratio		
**rs10488961**	4	calcium/calmodulin dependent protein kinase II delta	CAMK2D	Body Mass Index	CAMK2D	
**rs1363396**	5	NULL	NULL	Body Mass Index	PRR16, RPL23AP44	
**rs6580146**	5	NULL	NULL	Body Mass Index	KIF4B,P PIGP1	
**rs162307**	5	NULL	NULL	Cholesterol LDL	FAM170A, PRR16	
**rs1822489**	5	MCC regulator of WNT signaling pathway	MCC	Gamma glutamyl transferase levels, Waist-hip ratio		
**rs1432723**	5	NULL	NULL	Obesity-related traits	SGCD	
**rs9275393**	6	NULL	NULL	Cholesterol total		
**rs9275371**	6	NULL	NULL	Cholesterol total		
**rs9275418**	6	NULL	NULL	Cholesterol total		

SNP	Chr	Gene	Gene symbol	Phenotype	Assoc. genes	Clinical Significance
**rs2293289**	6	1-acylglycerol-3-phosphate O-acyltransferase 4	AGPAT4	Lipoprotein (a) - cholesterol levels	AGPAT4	
**rs1488**	6	mitogen-activated protein kinase kinase kinase 4	MAP3K4	Lipoproteins	MAP3K4	
**rs10952650**	7	contactin associated protein 2	CNTNAP2	Diabetes Mellitus	CNTNAP2	
**rs2971760**	7	NULL	NULL	Type 2 diabetes		
**rs4737384**	8	staufen double-stranded RNA binding protein 2	STAU2	Body Mass Index, Body Weight Changes	RDH10,STAU2	
**rs2169385**	8	NULL	NULL	Cholesterol total, High-density lipoprotein cholesterol		
**rs12546198**	8	NULL	NULL	Cholesterol total, High-density lipoprotein cholesterol, Cake icing liking		
**rs4620259**	8	NULL	NULL	LDL cholesterol levels	TRHR	
**rs12546225**	8	NULL	NULL	Waist-hip ratio	RPL26P26, UBXN2B	
**rs3861048**	10	NULL	NULL	Body Fat Distribution, Body Mass Index, Body Weight, Waist circumference	GAPDHP21, ZWINT	
**rs10509165**	10	NULL	NULL	Cholesterol HDL, Receptors Tumor Necrosis Factor Type II	ARID5B, RTKN2	
**rs10509138**	10	NULL	NULL	Cholesterol LDL	ANK3	
**rs2393726**	10	AT-rich interaction domain 5B	ARID5B	Serum metabolite levels	ARID5B	
**rs2254069**	10	NULL	NULL	Waist-hip ratio, Waist-to-hip ratio adjusted for BMI		
**rs174601**	11	fatty acid desaturase 2	FADS2	Cholesterol total, High-density lipoprotein cholesterol, Red blood cell fatty acid levels, Fatty acid levels, Total cholesterol levels, Trans fatty acid levels, Triglycerides	C11orf9, C11orf10,FEN1, FADS1,FADS2, RAB3IL1,DAG LA,BEST1	
**rs174570**	11	fatty acid desaturase 2	FADS2	Cholesterol total, High-density lipoprotein cholesterol, Red blood cell fatty acid levels, Fatty acid levels, Total cholesterol levels, Trans fatty acid levels, Triglycerides	FADS2, FADS3	
**rs174574**	11	fatty acid desaturase 2	FADS2	Cholesterol total, High-density lipoprotein cholesterol, Red blood cell fatty acid levels, Fatty acid levels, Total cholesterol levels, Trans fatty acid levels, Triglycerides	FADS2, FADS1	
**rs11041816**	11	NULL	NULL	Fasting blood glucose (BMI interaction), Fasting blood glucose	LMO1	
**rs174583**	11	fatty acid desaturase 2	FADS2	Cholesterol total, High-density lipoprotein cholesterol, Red blood cell fatty acid levels, Fatty acid levels, Total cholesterol levels, Trans fatty acid levels, Triglycerides	FADS2, C11orf10, C11orf9,FADS1, FEN1, FADS3, FADS1	
**rs174576**	11	fatty acid desaturase 2	FADS2	Cholesterol total, High-density lipoprotein cholesterol, Red blood cell fatty acid levels, Fatty acid levels, Total cholesterol levels, Trans fatty acid levels, Triglycerides	FADS2,FADS1, FADS3, FADS2,C11orf9, C11orf10,FEN1	
**rs102275**	11	transmembrane protein 258	TMEM258	Cholesterol total, High-density lipoprotein cholesterol, Red blood cell fatty acid levels, Fatty acid levels, Total cholesterol levels,	C11orf10, FADS1,FADS2, FEN1FADS3, TMEM258, FTH1,INCENP,	

SNP	Chr	Gene	Gene symbol	Phenotype	Assoc. genes	Clinical Significance
				Trans fatty acid levels, Triglycerides	SCGB2A1,SCGB1D1,SCGB2A2,RAB3IL1,AHNAK,C11orf9,DAGLA,SYT7,BEST1, MYRF	
**rs174556**	11	fatty acid desaturase 1	FADS1	Cholesterol total, High-density lipoprotein cholesterol, Red blood cell fatty acid levels, Fatty acid levels, Total cholesterol levels, Trans fatty acid levels, Triglycerides	FADS1, FADS2	
**rs1535**	11	fatty acid desaturase 2	FADS2	Cholesterol total, High-density lipoprotein cholesterol, Red blood cell fatty acid levels, Fatty acid levels, Total cholesterol levels, Trans fatty acid levels, Triglycerides	FADS2, FEN,FADS1, FADS3	
**rs174546**	11	fatty acid desaturase 1	FADS1	Cholesterol total, High-density lipoprotein cholesterol, Red blood cell fatty acid levels, Fatty acid levels, Total cholesterol levels, Trans fatty acid levels, Triglycerides	FADS1, FADS2, FADS3	
**rs174537**	11	myelin regulatory factor	MYRF	Cholesterol total, High-density lipoprotein cholesterol, Red blood cell fatty acid levels, Fatty acid levels, Total cholesterol levels, Trans fatty acid levels, Triglycerides	C11orf9, MYRF,FADS1,FADS2,FADS3, FEN1	
**rs174550**	11	fatty acid desaturase 1	FADS1	Cholesterol total, High-density lipoprotein cholesterol, Red blood cell fatty acid levels, Fatty acid levels, Total cholesterol levels, Trans fatty acid levels, Triglycerides	FADS1,FADS2, C11orf10,C11orf9,FEN1,RAB3IL1, FTH1,INCENP, FADS1,SCGB1D1,SCGB2A1,DAGLA,RAB3IL, FADS3,SYT7,BEST1,SCGB1D2, BEST1	
**rs174535**	11	myelin regulatory factor	MYRF	Cholesterol total, High-density lipoprotein cholesterol, Red blood cell fatty acid levels, Fatty acid levels, Total cholesterol levels, Trans fatty acid levels, Triglycerides	MYRF, C11orf9,C11orf10,FADS2,FADS1,FEN1	benign
**rs174549**	11	fatty acid desaturase 1	FADS1	Cholesterol total, High-density lipoprotein cholesterol, Red blood cell fatty acid levels, Fatty acid levels, Total cholesterol levels, Trans fatty acid levels, Triglycerides	FADS2, FADS1,C11orf9, C11orf10,FEN1, FADS3	
**rs174536**	11	myelin regulatory factor	MYRF	Cholesterol total, High-density lipoprotein cholesterol, Red blood cell fatty acid levels, Fatty acid levels, Total cholesterol levels, Trans fatty acid levels, Triglycerides	FEN1,FADS2,TMEM258,MYRF,C11orf9	benign
**rs174547**	11	fatty acid desaturase 1	FADS1	Cholesterol total, High-density lipoprotein cholesterol, Red blood cell fatty acid levels, Fatty acid levels, Total cholesterol levels, Trans fatty acid levels, Triglycerides	FADS1,FADS2, FADS3, C11orf10,C11orf9,FEN1, FTH1,C11orf9,DAGLA,RAB3IL1,BEST1, FTH1,INCENP, SCGB2A1,SCGB1D1,SCGB2A2,AHNAK,SYT7,	

SNP	Chr	Gene	Gene symbol	Phenotype	Assoc. genes	Clinical Significance
**rs174541**	11	NULL	NULL	Cholesterol total, High-density lipoprotein cholesterol, Red blood cell fatty acid levels, Fatty acid levels, Total cholesterol levels, Trans fatty acid levels, Triglycerides	VRK2,FANCL, FEN1,FADS1, C11orf10,C11orf 9,FADS2, FEN1, FADS3, MAP2	
**rs174548**	11	fatty acid desaturase 1	FADS1	Cholesterol total, High-density lipoprotein cholesterol, Red blood cell fatty acid levels, Fatty acid levels, Total cholesterol levels, Trans fatty acid levels, Triglycerides	FADS1, FEN1, FADS2	
**rs174450**	11	fatty acid desaturase 3	FADS3	Cholesterol total, High-density lipoprotein cholesterol, Red blood cell fatty acid levels, Fatty acid levels, Total cholesterol levels, Trans fatty acid levels, Triglycerides	FADS3	
**rs108499**	11	myelin regulatory factor	MYRF	Cholesterol total, High-density lipoprotein cholesterol, Red blood cell fatty acid levels, Fatty acid levels, Total cholesterol levels, Trans fatty acid levels, Triglycerides	MYRF	benign
**rs174534**	11	myelin regulatory factor	MYRF	Cholesterol total, High-density lipoprotein cholesterol, Red blood cell fatty acid levels, Fatty acid levels, Total cholesterol levels, Trans fatty acid levels, Triglycerides	MYRF	benign
**rs174538**	11	NULL	NULL	Cholesterol total, High-density lipoprotein cholesterol, Red blood cell fatty acid levels, Fatty acid levels, Total cholesterol levels, Trans fatty acid levels, Triglycerides	ALPK1,NEURO G2,C4orf21,LA RP7, TMEM258, C11orf10, MYRF,TMEM2 58,FEN1,FADS2 , FADS1	
**rs7115739**	11	fatty acid desaturase 3	FADS3	Cholesterol total, High-density lipoprotein cholesterol, Red blood cell fatty acid levels, Fatty acid levels, Total cholesterol levels, Trans fatty acid levels, Triglycerides		
**rs198462**	11	myelin regulatory factor	MYRF	Cholesterol total, High-density lipoprotein cholesterol, Red blood cell fatty acid levels, Fatty acid levels, Total cholesterol levels, Trans fatty acid levels, Triglycerides		
**rs174448**	11	NULL	NULL	Cholesterol total, High-density lipoprotein cholesterol, Red blood cell fatty acid levels, Fatty acid levels, Total cholesterol levels, Trans fatty acid levels, Triglycerides	FADS3	
**rs2732494**	12	NULL	NULL	Body Fat Distribution, Body Mass Index, Body Weight, Waist circumference	PGBD3P3,TME M132C	
**rs7138792**	12	NULL	NULL	Body Fat Distribution, Body Weight	PGBD3P3,TME M132C	
**rs2701248**	12	NULL	NULL	Body Fat Distribution, Body Weight	PGBD3P3,TME M132C	
**rs10744625**	12	NULL	NULL	Homeostasis model assessment of insulin resistance (dietary factor interaction), Fasting insulin (dietary factor interaction)	EFCAB4B,PAR P11	
**rs7309378**	12	G protein-coupled receptor 19	GPR19	Protein levels in obesity		
**rs7989336**	13	heparan sulfate 6-O-sulfotransferase 3	HS6ST3	Obesity	HS6ST3	

SNP	Chr	Gene	Gene symbol	Phenotype	Assoc. genes	Clinical Significance
**rs5008128**	13	NULL	NULL	Waist circumference	KLF12	
**rs3848**	14	neuronal PAS domain protein 3	NPAS3	Body Composition, Body Mass Index	NPAS3	
**rs1041857**	14	NULL	NULL	Cholesterol HDL	SALL2,OR10G3	
**rs10484197**	14	MAM domain containing glycosylphosphatidylinositol anchor 2	MDGA2	Cholesterol LDL	MDGA2	
**rs10518907**	15	NULL	NULL	Body Weights and Measures	TCF12,CGNL1	
**rs737008**	16	protamine 1	PRM1	Obesity-related traits, ClinVar: phenotype not specified	PRM1	benign
**rs9330248**	17	acetyl-CoA carboxylase alpha	ACACA	Body Mass Index, Age at menopause	ACACA,	
**rs9330248**	17	NULL	NULL	Body Mass Index, Age at menopause	ACACA,	
**rs797973**	17	NULL	NULL	Type 2 diabetes		
**rs12104221**	19	NULL	NULL	Obesity-related traits	MATK	
**rs138354**	22	X-prolyl aminopeptidase 3	XPNPEP3	Apolipoprotein B levels	XPNPEP3	
**rs733381**	22	trinucleotide repeat containing adaptor 6B	TNRC6B	Body Mass Index, Waist-hip ratio		
**rs2076674**	22	solute carrier family 25 member 17	SLC25A17	Low density lipoprotein cholesterol levels	SLC25A17	

SNP: Single Nucleotide Polymorphism identifier; Chr: Chromosome number.

Table S4. Top lipid metabolism-associated genes for TIX.

SNP	Chr	Gene	Gene symbol	Phenotype	Assoc. genes	Clinical Significance
**rs11102002**	1	EPS8 like 3	EPS8L3	Apolipoprotein B levels		
**rs1165226**	1	ubiquitin specific peptidase 24	USP24	Cholesterol total		
**rs7551981**	1	NULL	NULL	Cholesterol total		
**rs11102002**	1	EPS8 like 3	EPS8L3	Low density lipoprotein cholesterol levels		
**rs1165226**	1	ubiquitin specific peptidase 24	USP24	Low-density lipoprotein cholesterol		
**rs6719729**	2	raftlin family member 2	RFTN2	Body Fat Distribution	RFTN2	
**rs10497870**	2	neurobeachin like 1	NBEAL1	Body Mass Index	NA	
**rs3755157**	2	ATP binding cassette subfamily B member 11	ABCB11	Fasting Glucose		benign
**rs3755157**	2	NULL	NULL	Fasting Glucose		benign
**rs7645613**	3	NULL	NULL	Type 2 diabetes		
**rs4833079**	4	NULL	NULL	Body Mass Index		
**rs4833079**	4	NULL	NULL	Body Mass Index	FLJ13197	
**rs6876835**	5	NULL	NULL	Body Fat Distribution	NSUN2	
**rs12652687**	5	F-box and leucine rich repeat protein 17	FBXL17	Body Mass Index	FBXL17	
**rs3776717**	5	ADP ribosylation factor like GTPase 15	ARL15	Body mass index and HDL-C pairwise	ARL15	
**rs3776717**	5	ADP ribosylation factor like GTPase 15	ARL15	Body mass index and type 2 diabetes pairwise	ARL15	
**rs4460176**	5	NULL	NULL	Cholesterol HDL	MAN2A1,TMEM232	
**rs2963826**	5	phosphodiesterase 4D	PDE4D	Obesity-related traits	PDE4D	
**rs4916749**	5	NULL	NULL	Waist circumference adjusted for body mass index	MEF2C-AS1	
**rs7752021**	6	NULL	NULL	Cholesterol total		
**rs9275524**	6	NULL	NULL	Cholesterol total		
**rs9275555**	6	NULL	NULL	Cholesterol total		
**rs9275578**	6	NULL	NULL	Cholesterol total		
**rs9275595**	6	NULL	NULL	Cholesterol total		
**rs434841**	6	notch receptor 4	NOTCH4	Diabetes mellitus type 1	NOTCH4	benign
**rs434841**	6	NULL	NULL	Diabetes mellitus type 1	NOTCH4	benign
**rs12214416**	6	NULL	NULL	Lp a levels	LPAL2	
**rs434841**	6	notch receptor 4	NOTCH4	Waist-to-hip ratio adjusted for BMI	NOTCH4	benign
**rs434841**	6	NULL	NULL	Waist-to-hip ratio adjusted for BMI	NOTCH4	benign
**rs2040369**	7	NULL	NULL	Body Weight	TRBV5-6, TRBV7-6	
**rs4719818**	7	NULL	NULL	Hip circumference adjusted for BMI	AC003090.1	
**rs4719818**	7	NULL	NULL	Hip index	AC003090.1	
**rs1528036**	7	inner mitochondrial	IMMP2L	Lipoproteins	IMMP2L	

SNP	Chr	Gene	Gene symbol	Phenotype	Assoc. genes	Clinical Significance
		membrane peptidase subunit 2				
**rs10487878**	7	semaphorin 3C	SEMA3C	Lipoproteins VLDL	SEMA3C	
**rs10950840**	7	NULL	NULL	Obesity-related traits	ASS1P11	
**rs9987000**	7	NULL	NULL	Waist-hip ratio		
**rs822318**	8	NULL	NULL	Cholesterol HDL	MRPL49P2, FGF20	
**rs4876361**	8	NULL	NULL	Hip circumference adjusted for BMI	EIF3H	
**rs4994**	8	adrenoceptor beta 3	ADRB3	Obesity	ADRB3	benign, risk factor
**rs4876361**	8	NULL	NULL	Waist circumference adjusted for body mass index	EIF3H	
**rs7858161**	9	NULL	NULL	Hip circumference adjusted for BMI	YBX1P6	
**rs1458495**	9	NULL	NULL	Waist circumference	ANXA1, RORB	
**rs7039360**	9	NULL	NULL	Waist circumference	TLE4, RPS19P6	
**rs4506565**	10	transcription factor 7 like 2	TCF7L2	Body Mass Index	NA	
**rs4506565**	10	transcription factor 7 like 2	TCF7L2	Fasting blood glucose	TCF7L2	
**rs4506565**	10	transcription factor 7 like 2	TCF7L2	Fasting Glucose		
**rs4506565**	10	transcription factor 7 like 2	TCF7L2	Fasting Glucose	TCF7L2	
**rs1937353**	10	NULL	NULL	Hip	ST8SIA6,PTPLA	
**rs4506565**	10	transcription factor 7 like 2	TCF7L2	Type 2 diabetes	TCF7L2	
**rs4506565**	10	transcription factor 7 like 2	TCF7L2	Type 2 diabetes		
**rs4938362**	11	proprotein convertase subtilisin/kexin type 7	PCSK7	Apolipoprotein A1 levels		
**rs4938362**	11	proprotein convertase subtilisin/kexin type 7	PCSK7	Apolipoprotein B levels		
**rs1003081**	11	NULL	NULL	Body Mass Index	NA	
**rs10502222**	11	SIK family kinase 3	SIK3	Cholesterol total		
**rs4938362**	11	proprotein convertase subtilisin/kexin type 7	PCSK7	Low density lipoprotein cholesterol levels		
**rs4938362**	11	proprotein convertase subtilisin/kexin type 7	PCSK7	Total cholesterol levels		
**rs4938362**	11	proprotein convertase subtilisin/kexin type 7	PCSK7	Triglyceride levels		
**rs2728641**	12	NULL	NULL	BMI at 3 months old		
**rs2728641**	12	NULL	NULL	BMI at 6 months old		
**rs17201502**	12	Fas apoptotic inhibitory molecule 2	FAIM2	Body Mass Index		
**rs7302017**	12	NULL	NULL	Waist circumference	PPM1H	
**rs10149366**	14	NULL	NULL	Lipids	RPL3P3,EXOC5	
**rs10149366**	14	NULL	NULL	Triglycerides	RPL3P3,EXOC5	
**rs242105**	14	NULL	NULL	Type 2 diabetes		

SNP	Chr	Gene	Gene symbol	Phenotype	Assoc. genes	Clinical Significance
**rs8037818**	15	Rho GTPase activating protein 11A	ARHGAP11A	Obesity-related traits	ARHGAP11A,SCG5	
**rs8037818**	15	NULL	NULL	Obesity-related traits	ARHGAP11A,SCG5	
**rs9939973**	16	FTO alpha-ketoglutarate dependent dioxygenase	FTO	Body Mass Index		
**rs9939973**	16	FTO alpha-ketoglutarate dependent dioxygenase	FTO	Hip circumference	FTO	
**rs11152166**	18	collagen and calcium binding EGF domains 1	CCBE1	Body Mass Index	CCBE1	
**rs1865063**	19	dedicator of cytokinesis 6	DOCK6	HDL cholesterol levels	ANGPTL8	
**rs166988**	19	NULL	NULL	Obesity-related traits	KCTD15	

SNP: Single Nucleotide Polymorphism identifier; Chr: Chromosome number.

Table S5. Top lipid metabolism-associated genes comparison for YCH and TIX.

YCH	TIX
**ACACA**	ABCB11
**AGPAT4**	ADRB3
**ARID5B**	ARHGAP11A
**CAMK2D**	ARL15
**CLCNKA**	CCBE1
**CNTNAP2**	DOCK6
**FADS1**	EPS8L3
**FADS2**	FAIM2
**FADS3**	FBXL17
**FAP**	FTO
**FMN2**	IMMP2L
**GPR19**	NBEAL1
**HS6ST3**	NOTCH4
**IFIH1**	PCSK7
**IL23R**	PDE4D
**LBH**	RFTN2
**MAP3K4**	SEMA3C
**MCC**	SIK3
**MDGA2**	TCF7L2
**MYRF**	USP24
**NPAS3**	
**PLB1**	
**PRM1**	
**SLC25A17**	
**STAU2**	
**TMEM258**	
**TNRC6B**	
**WDSUB1**	
**XPNPEP3**	

Table S6. GoWinda enrichment analysis results for YCH.

GO Term	ANG	Can	FDR	Unique	Max	Total	Description
**GO:2000113**	133.14	210	0.00014269	82	1069	1371	negative regulation of cellular macromolecule biosynthetic process
**GO:2000112**	359.48	474	0.00014269	197	3081	3826	regulation of cellular macromolecule biosynthetic process
**GO:2000116**	25.09	54	0.00014269	15	190	237	regulation of cysteine-type endopeptidase activity
**GO:0035150**	5.55	25	0.00014269	5	47	58	regulation of tube size
**GO:0072126**	0.77	7	0.00014269	1	5	5	positive regulation of glomerular mesangial cell proliferation
**GO:0006636**	3.32	24	0.00014269	5	39	50	unsaturated fatty acid biosynthetic process
**GO:0032927**	0.56	6	0.00014269	2	8	14	positive regulation of activin receptor signaling pathway
**GO:0019941**	25.20	64	0.00014269	19	314	406	modification-dependent protein catabolic process
**GO:1901215**	21.39	49	0.00014269	16	159	186	negative regulation of neuron death
**GO:1901214**	36.85	68	0.00014269	21	237	286	regulation of neuron death
**GO:0006633**	8.83	26	0.00014269	6	80	101	fatty acid biosynthetic process
**GO:0006631**	24.86	52	0.00014269	21	254	289	fatty acid metabolic process
**GO:0048477**	3.33	17	0.00014269	2	27	29	oogenesis
**GO:0006689**	0.46	7	0.00014269	1	5	6	ganglioside catabolic process
**GO:0016010**	4.04	20	0.00014269	3	14	15	dystrophin-associated glycoprotein complex
**GO:0006690**	6.38	20	0.00014269	9	78	92	icosanoid metabolic process
**GO:0043632**	25.75	64	0.00014269	19	318	410	modification-dependent macromolecule catabolic process
**GO:0042311**	2.53	12	0.00014269	3	19	22	vasodilation
**GO:0098793**	11.17	31	0.00014269	9	50	58	presynapse
**GO:0043651**	1.00	20	0.00014269	2	15	17	linoleic acid metabolic process
**GO:0031668**	16.35	37	0.00014269	11	163	200	cellular response to extracellular stimulus
**GO:0031669**	14.38	37	0.00014269	11	140	167	cellular response to nutrient levels
**GO:0031667**	32.90	83	0.00014269	27	309	363	response to nutrient levels
**GO:0016032**	40.25	73	0.00014269	28	414	517	viral process
**GO:0043615**	0.37	7	0.00014269	1	4	8	astrocyte cell migration
**GO:0060179**	0.31	7	0.00014269	1	4	5	male mating behavior
**GO:0060180**	1.06	13	0.00014269	1	4	7	female mating behavior
**GO:0031594**	11.82	30	0.00014269	13	40	46	neuromuscular junction
**GO:0000422**	5.03	21	0.00014269	4	22	25	mitophagy
**GO:0030282**	3.13	17	0.00014269	5	31	39	bone mineralization
**GO:0030275**	2.39	14	0.00014269	2	14	18	LRR domain binding
**GO:0000460**	0.50	6	0.00014269	1	7	8	maturation of 5.8S rRNA
**GO:0035112**	2.00	14	0.00014269	3	11	13	genitalia morphogenesis
**GO:0050709**	12.99	32	0.00014269	5	94	139	negative regulation of protein secretion
**GO:0060255**	571.60	680	0.00014269	296	4684	5767	regulation of macromolecule metabolic process
**GO:0060205**	24.41	48	0.00014269	21	282	379	cytoplasmic membrane-bounded vesicle lumen
**GO:0050880**	5.46	25	0.00014269	5	46	56	regulation of blood vessel size
**GO:0050896**	486.92	575	0.00014269	252	4122	5595	response to stimulus
**GO:0060211**	1.00	8	0.00014269	1	8	9	regulation of nuclear-transcribed mRNA poly(A) tail shortening
**GO:0006750**	0.88	12	0.00014269	1	13	14	glutathione biosynthetic process
**GO:0060213**	1.00	8	0.00014269	1	8	9	positive regulation of nuclear-transcribed mRNA poly(A) tail shortening
**GO:0090090**	21.07	58	0.00014269	13	142	180	negative regulation of canonical Wnt signaling pathway
**GO:0090091**	0.93	8	0.00014269	2	6	7	positive regulation of extracellular matrix disassembly
**GO:0006749**	1.60	12	0.00014269	1	35	45	glutathione metabolic process
**GO:1901165**	0.28	5	0.00014269	1	6	6	positive regulation of trophoblast cell migration
**GO:0042383**	19.78	43	0.00014269	12	79	87	sarcolemma
**GO:0042398**	2.86	12	0.00014269	1	41	46	cellular modified amino acid biosynthetic process
**GO:0048519**	562.40	656	0.00014269	273	3923	4898	negative regulation of biological process
**GO:0048523**	526.03	632	0.00014269	262	3674	4581	negative regulation of cellular process
**GO:0050815**	0.34	5	0.00014269	1	4	5	phosphoserine binding
**GO:0000280**	2.71	14	0.00014269	6	48	55	nuclear division
**GO:0036293**	40.73	74	0.00014269	23	244	276	response to decreased oxygen levels
**GO:0006403**	0.98	8	0.00014269	2	10	12	RNA localization
**GO:0051851**	6.85	22	0.00014269	5	52	71	modification by host of symbiont morphology or physiology
**GO:0048227**	0.52	6	0.00014269	1	8	8	plasma membrane to endosome transport
**GO:0019752**	76.82	133	0.00014269	53	725	821	carboxylic acid metabolic process
**GO:0018401**	2.00	14	0.00014269	1	5	5	peptidyl-proline hydroxylation to 4-hydroxy-L-proline
**GO:0048285**	9.60	26	0.00014269	8	71	79	organelle fission

GO Term	ANG	Can	FDR	Unique	Max	Total	Description
**GO:0005164**	2.15	14	0.00014269	5	26	46	tumor necrosis factor receptor binding
**GO:0031418**	3.75	16	0.00014269	2	20	20	L-ascorbic acid binding
**GO:0019787**	32.31	74	0.00014269	19	300	345	ubiquitin-like protein transferase activity
**GO:0044703**	12.84	34	0.00014269	11	116	147	multi-organism reproductive process
**GO:0044706**	11.19	30	0.00014269	9	102	124	multi-multicellular organism process
**GO:0044705**	4.96	22	0.00014269	3	23	27	multi-organism reproductive behavior
**GO:0031406**	27.06	68	0.00014269	22	181	206	carboxylic acid binding
**GO:0043374**	0.30	10	0.00014269	2	6	7	CD8-positive, alpha-beta T cell differentiation
**GO:0031331**	28.56	58	0.00014269	14	310	369	positive regulation of cellular catabolic process
**GO:0070979**	5.16	24	0.00014269	3	24	28	protein K11-linked ubiquitination
**GO:0031327**	144.84	225	0.00014269	87	1179	1522	negative regulation of cellular biosynthetic process
**GO:0031326**	385.31	492	0.00014269	204	3314	4127	regulation of cellular biosynthetic process
**GO:0031390**	0.36	7	0.00014269	1	6	6	Ctf18 RFC-like complex
**GO:0014823**	6.74	26	0.00014269	6	50	59	response to activity
**GO:0017080**	5.34	17	0.00014269	6	22	24	sodium channel regulator activity
**GO:0030046**	1.75	11	0.00014269	2	5	5	parallel actin filament bundle assembly
**GO:0070936**	7.92	27	0.00014269	6	43	54	protein K48-linked ubiquitination
**GO:0051817**	10.97	36	0.00014269	11	84	107	modification of morphology or physiology of other organism involved in symbiotic interaction
**GO:0000209**	23.84	67	0.00014269	16	233	284	protein polyubiquitination
**GO:0001573**	1.67	11	0.00014269	3	15	20	ganglioside metabolic process
**GO:0050685**	2.57	15	0.00014269	3	28	33	positive regulation of mRNA processing
**GO:0050680**	13.97	32	0.00014269	8	101	124	negative regulation of epithelial cell proliferation
**GO:0006520**	33.47	68	0.00014269	25	306	342	cellular amino acid metabolic process
**GO:0006534**	0.60	12	0.00014269	1	8	10	cysteine metabolic process
**GO:1903506**	328.15	430	0.00014269	181	2776	3476	regulation of nucleic acid-templated transcription
**GO:1903507**	121.47	185	0.00014269	76	952	1236	negative regulation of nucleic acid-templated transcription
**GO:0006511**	24.92	64	0.00014269	19	309	401	ubiquitin-dependent protein catabolic process
**GO:1903508**	185.93	245	0.00014269	99	1270	1562	positive regulation of nucleic acid-templated transcription
**GO:0006508**	113.19	173	0.00014269	67	974	1208	proteolysis
**GO:0044849**	1.55	13	0.00014269	4	14	14	estrous cycle
**GO:0006575**	16.88	39	0.00014269	11	168	196	cellular modified amino acid metabolic process
**GO:0043523**	24.68	53	0.00014269	15	159	190	regulation of neuron apoptotic process
**GO:0006536**	2.07	15	0.00014269	2	24	26	glutamate metabolic process
**GO:0032813**	3.16	15	0.00014269	6	39	60	tumor necrosis factor receptor superfamily binding
**GO:0019852**	1.10	12	0.00014269	1	10	10	L-ascorbic acid metabolic process
**GO:0031545**	2.33	14	0.00014269	1	6	6	peptidyl-proline 4-dioxygenase activity
**GO:0031543**	2.78	14	0.00014269	1	9	9	peptidyl-proline dioxygenase activity
**GO:0044827**	4.13	19	0.00014269	3	12	13	modulation by host of viral genome replication
**GO:0044829**	0.30	5	0.00014269	1	7	7	positive regulation by host of viral genome replication
**GO:0042133**	3.93	18	0.00014269	6	24	34	neurotransmitter metabolic process
**GO:0042136**	2.87	15	0.00014269	4	14	21	neurotransmitter biosynthetic process
**GO:0031461**	6.65	29	0.00014269	6	101	110	cullin-RING ubiquitin ligase complex
**GO:0030130**	1.61	10	0.00014269	1	6	6	clathrin coat of trans-Golgi network vesicle
**GO:0030123**	0.60	9	0.00014269	2	7	7	AP-3 adaptor complex
**GO:0030125**	1.77	10	0.00014269	1	9	9	clathrin vesicle coat
**GO:0032787**	44.72	85	0.00014269	33	427	485	monocarboxylic acid metabolic process
**GO:0031442**	1.25	11	0.00014269	2	15	17	positive regulation of mRNA 3'-end processing
**GO:0044764**	40.85	73	0.00014269	28	427	534	multi-organism cellular process
**GO:0043436**	91.76	149	0.00014269	61	822	925	oxoacid metabolic process
**GO:0044770**	18.95	44	0.00014269	12	218	251	cell cycle phase transition
**GO:0030175**	14.76	35	0.00014269	10	58	71	filopodium
**GO:0030178**	28.34	59	0.00014269	14	169	211	negative regulation of Wnt signaling pathway
**GO:0044772**	18.68	44	0.00014269	12	211	243	mitotic cell cycle phase transition
**GO:0097267**	0.41	7	0.00014269	3	9	9	omega-hydroxylase P450 pathway
**GO:0031490**	4.77	18	0.00014269	3	47	64	chromatin DNA binding
**GO:0030163**	24.38	60	0.00014269	18	285	354	protein catabolic process
**GO:0030162**	70.61	112	0.00014269	38	629	797	regulation of proteolysis
**GO:0044788**	4.73	19	0.00014269	3	21	22	modulation by host of viral process
**GO:0001666**	40.16	72	0.00014269	22	237	267	response to hypoxia
**GO:0001676**	8.65	35	0.00014269	10	79	90	long-chain fatty acid metabolic process
**GO:0001678**	5.36	20	0.00014269	6	59	63	cellular glucose homeostasis
**GO:0051900**	2.05	13	0.00014269	2	18	19	regulation of mitochondrial depolarization
**GO:0071242**	8.13	26	0.00014269	6	31	42	cellular response to ammonium ion
**GO:0034284**	12.30	34	0.00014269	13	121	139	response to monosaccharide
**GO:0071230**	7.62	27	0.00014269	5	46	61	cellular response to amino acid stimulus
**GO:0005766**	0.74	7	0.00014269	1	10	11	primary lysosome
**GO:1901699**	66.45	106	0.00014269	37	383	456	cellular response to nitrogen compound

GO Term	ANG	Can	FDR	Unique	Max	Total	Description
**GO:0016458**	6.56	21	0.00014269	6	70	111	gene silencing
**GO:0042737**	1.19	12	0.00014269	4	12	16	drug catabolic process
**GO:0042738**	1.11	12	0.00014269	4	10	12	exogenous drug catabolic process
**GO:0042765**	0.23	6	0.00014269	1	5	5	GPI-anchor transamidase complex
**GO:0090329**	4.14	17	0.00014269	3	39	45	regulation of DNA-dependent DNA replication
**GO:0010265**	1.01	18	0.00014269	1	4	5	SCF complex assembly
**GO:0060548**	95.59	142	0.00014269	59	775	952	negative regulation of cell death
**GO:0060509**	1.88	13	0.00014269	1	4	5	Type I pneumocyte differentiation
**GO:0035578**	5.21	17	0.00014269	9	75	104	azurophil granule lumen
**GO:1901605**	23.73	62	0.00014269	21	219	244	alpha-amino acid metabolic process
**GO:0071372**	2.11	14	0.00014269	2	11	11	cellular response to follicle-stimulating hormone stimulus
**GO:0071371**	2.75	14	0.00014269	2	17	18	cellular response to gonadotropin stimulus
**GO:1900262**	0.36	7	0.00014269	1	6	6	regulation of DNA-directed DNA polymerase activity
**GO:1900264**	0.36	7	0.00014269	1	6	6	positive regulation of DNA-directed DNA polymerase activity
**GO:0003254**	8.10	29	0.00014269	7	37	42	regulation of membrane depolarization
**GO:0071333**	4.66	20	0.00014269	6	51	54	cellular response to glucose stimulus
**GO:0071331**	4.81	20	0.00014269	6	54	57	cellular response to hexose stimulus
**GO:0071322**	6.28	21	0.00014269	7	60	63	cellular response to carbohydrate stimulus
**GO:0071326**	4.84	20	0.00014269	6	55	58	cellular response to monosaccharide stimulus
**GO:0016567**	52.93	96	0.00014269	30	583	720	protein ubiquitination
**GO:1901568**	6.38	20	0.00014269	9	78	92	fatty acid derivative metabolic process
**GO:0004563**	0.55	7	0.00014269	1	5	5	beta-N-acetylhexosaminidase activity
**GO:0030897**	0.53	6	0.00014269	2	6	6	HOPS complex
**GO:0016597**	16.05	37	0.00014269	11	94	112	amino acid binding
**GO:0009313**	0.77	9	0.00014269	3	10	20	oligosaccharide catabolic process
**GO:0009311**	3.98	17	0.00014269	8	43	56	oligosaccharide metabolic process
**GO:0010389**	15.07	34	0.00014269	7	161	204	regulation of G2/M transition of mitotic cell cycle
**GO:0071315**	0.36	7	0.00014269	1	2	7	cellular response to morphine
**GO:0071317**	0.36	7	0.00014269	1	2	7	cellular response to isoquinoline alkaloid
**GO:0005828**	0.62	7	0.00014269	1	6	6	kinetochore microtubule
**GO:0033057**	4.29	20	0.00014269	2	17	21	multicellular organismal reproductive behavior
**GO:0060359**	16.43	37	0.00014269	9	75	94	response to ammonium ion
**GO:1900151**	1.03	8	0.00014269	1	11	14	regulation of nuclear-transcribed mRNA catabolic process, deadenylation-dependent decay
**GO:1900153**	1.03	8	0.00014269	1	11	14	positive regulation of nuclear-transcribed mRNA catabolic process, deadenylation-dependent decay
**GO:2001234**	17.63	37	0.00014269	11	171	208	negative regulation of apoptotic signaling pathway
**GO:1902750**	6.31	23	0.00014269	3	83	109	negative regulation of cell cycle G2/M phase transition
**GO:0034097**	45.55	78	0.00014269	37	414	503	response to cytokine
**GO:0016255**	0.33	6	0.00014269	1	7	7	attachment of GPI anchor to protein
**GO:0016236**	11.21	33	0.00014269	7	95	110	macroautophagy
**GO:0030522**	22.21	46	0.00014269	15	148	182	intracellular receptor signaling pathway
**GO:0001959**	19.91	42	0.00014269	13	135	178	regulation of cytokine-mediated signaling pathway
**GO:0001961**	6.20	22	0.00014269	6	38	49	positive regulation of cytokine-mediated signaling pathway
**GO:0022010**	0.47	7	0.00014269	1	8	12	central nervous system myelination
**GO:0048609**	45.91	91	0.00014269	32	429	558	multicellular organismal reproductive process
**GO:0009057**	57.69	102	0.00014269	41	673	868	macromolecule catabolic process
**GO:0009064**	4.94	23	0.00014269	6	57	68	glutamine family amino acid metabolic process
**GO:0009069**	2.58	13	0.00014269	2	32	36	serine family amino acid metabolic process
**GO:0035329**	4.38	18	0.00014269	3	26	29	hippo signaling
**GO:1902679**	122.24	187	0.00014269	77	961	1261	negative regulation of RNA biosynthetic process
**GO:1900019**	2.13	11	0.00014269	2	4	5	regulation of protein kinase C activity
**GO:1902680**	186.77	245	0.00014269	99	1291	1584	positive regulation of RNA biosynthetic process
**GO:1900020**	2.13	11	0.00014269	2	4	5	positive regulation of protein kinase C activity
**GO:0006974**	55.28	90	0.00014269	30	586	737	cellular response to DNA damage stimulus
**GO:0060479**	3.26	14	0.00014269	2	20	21	lung cell differentiation
**GO:0034162**	1.06	8	0.00014269	4	12	15	toll-like receptor 9 signaling pathway
**GO:0006950**	246.48	320	0.00014269	124	2302	3024	response to stress
**GO:2001141**	328.74	430	0.00014269	181	2790	3502	regulation of RNA biosynthetic process
**GO:1903976**	0.34	7	0.00014269	1	4	5	negative regulation of glial cell migration
**GO:0003018**	10.78	32	0.00014269	8	72	89	vascular process in circulatory system
**GO:0005654**	293.58	370	0.00014269	172	2721	3336	nucleoplasm
**GO:0006979**	36.76	66	0.00014269	18	300	351	response to oxidative stress
**GO:0005663**	0.37	7	0.00014269	1	6	6	DNA replication factor C complex
**GO:0030669**	3.27	19	0.00014269	4	25	71	clathrin-coated endocytic vesicle membrane
**GO:0031983**	25.09	49	0.00014269	22	283	380	vesicle lumen

GO Term	ANG	Can	FDR	Unique	Max	Total	Description
**GO:1900087**	4.34	15	0.00014269	4	22	31	positive regulation of G1/S transition of mitotic cell cycle
**GO:0042582**	0.74	7	0.00014269	1	10	11	azurophil granule
**GO:0042594**	13.93	36	0.00014269	11	140	168	response to starvation
**GO:0061726**	5.03	21	0.00014269	4	22	25	mitochondrion disassembly
**GO:1903935**	0.04	3	0.00014269	1	3	9	response to sodium arsenite
**GO:1903936**	0.04	3	0.00014269	1	3	8	cellular response to sodium arsenite
**GO:0006914**	20.54	45	0.00014269	13	182	212	autophagy
**GO:0006919**	10.43	30	0.00014269	6	73	86	activation of cysteine-type endopeptidase activity involved in apoptotic process
**GO:0016881**	2.09	12	0.00014269	1	20	20	acid-amino acid ligase activity
**GO:0004887**	1.23	13	0.00014269	1	7	7	thyroid hormone receptor activity
**GO:0039528**	0.29	5	0.00014269	1	5	7	cytoplasmic pattern recognition receptor signaling pathway in response to virus
**GO:1903008**	6.24	21	0.00014269	4	39	44	organelle disassembly
**GO:1990247**	0.29	5	0.00014269	1	7	8	N6-methyladenosine-containing RNA binding
**GO:0051253**	125.81	194	0.00014269	81	1000	1307	negative regulation of RNA metabolic process
**GO:0051254**	190.79	259	0.00014269	103	1340	1638	positive regulation of RNA metabolic process
**GO:0051252**	337.51	453	0.00014269	192	2899	3629	regulation of RNA metabolic process
**GO:0009749**	11.63	33	0.00014269	12	111	129	response to glucose
**GO:0010715**	1.86	13	0.00014269	5	14	19	regulation of extracellular matrix disassembly
**GO:0009743**	14.91	35	0.00014269	14	138	158	response to carbohydrate
**GO:0009746**	11.83	33	0.00014269	12	116	134	response to hexose
**GO:0019005**	2.51	25	0.00014269	3	27	30	SCF ubiquitin ligase complex
**GO:0007026**	2.05	14	0.00014269	2	15	20	negative regulation of microtubule depolymerization
**GO:0010639**	34.53	64	0.00014269	21	262	305	negative regulation of organelle organization
**GO:0051172**	146.90	230	0.00014269	91	1217	1564	negative regulation of nitrogen compound metabolic process
**GO:0051171**	396.49	514	0.00014269	217	3430	4253	regulation of nitrogen compound metabolic process
**GO:0019048**	7.36	25	0.00014269	7	32	36	modulation by virus of host morphology or physiology
**GO:1990234**	41.36	78	0.00014269	24	459	543	transferase complex
**GO:0070324**	1.30	13	0.00014269	1	6	6	thyroid hormone binding
**GO:0060992**	0.53	6	0.00014269	1	6	6	response to fungicide
**GO:0034698**	4.13	16	0.00014269	3	26	29	response to gonadotropin
**GO:0032020**	0.66	8	0.00014269	1	4	6	ISG15-protein conjugation
**GO:0044068**	6.13	23	0.00014269	6	26	28	modulation by symbiont of host cellular process
**GO:0019054**	5.74	23	0.00014269	6	22	24	modulation by virus of host process
**GO:0045324**	0.52	6	0.00014269	2	5	5	late endosome to vacuole transport
**GO:0044003**	8.12	25	0.00014269	7	41	47	modification by symbiont of host morphology or physiology
**GO:0046685**	1.42	22	0.00014269	4	24	31	response to arsenic-containing substance
**GO:0046686**	3.28	19	0.00014269	5	48	62	response to cadmium ion
**GO:0004842**	30.85	71	0.00014269	18	280	322	ubiquitin-protein transferase activity
**GO:0019098**	5.18	22	0.00014269	3	24	28	reproductive behavior
**GO:0051305**	1.45	10	0.00014269	1	6	6	chromosome movement towards spindle pole
**GO:0070482**	42.62	82	0.00014269	25	262	301	response to oxygen levels
**GO:0001071**	98.48	143	0.00014269	55	787	987	nucleic acid binding transcription factor activity
**GO:0010823**	7.99	25	0.00014269	6	43	50	negative regulation of mitochondrion organization
**GO:0010821**	22.76	49	0.00014269	17	208	249	regulation of mitochondrion organization
**GO:0009889**	391.14	495	0.00014269	206	3373	4194	regulation of biosynthetic process
**GO:0007141**	1.13	8	0.00014269	3	21	23	male meiosis I
**GO:0007126**	2.71	14	0.00014269	6	48	55	meiotic nuclear division
**GO:0007127**	1.34	10	0.00014269	4	26	28	meiosis I
**GO:0034711**	0.11	5	0.00014269	1	4	5	inhibin binding
**GO:0051295**	1.75	11	0.00014269	2	4	5	establishment of meiotic spindle localization
**GO:0034774**	23.87	47	0.00014269	20	269	362	secretory granule lumen
**GO:1901987**	34.35	62	0.00014269	19	344	429	regulation of cell cycle phase transition
**GO:0071774**	2.93	18	0.00014269	4	28	37	response to fibroblast growth factor
**GO:1901990**	32.09	61	0.00014269	18	315	393	regulation of mitotic cell cycle phase transition
**GO:1901991**	12.80	30	0.00014269	7	165	209	negative regulation of mitotic cell cycle phase transition
**GO:0019184**	0.93	12	0.00014269	1	14	15	nonribosomal peptide biosynthetic process
**GO:0004977**	0.67	8	0.00014269	1	5	6	melanocortin receptor activity
**GO:0045454**	5.47	20	0.00014269	5	53	64	cell redox homeostasis
**GO:0004985**	0.84	8	0.00014269	1	8	9	opioid receptor activity
**GO:0071496**	23.44	56	0.00014269	16	228	270	cellular response to external stimulus
**GO:0071453**	19.13	42	0.00014269	14	117	129	cellular response to oxygen levels
**GO:0071455**	1.00	8	0.00014269	2	6	7	cellular response to hyperoxia
**GO:0038003**	0.84	8	0.00014269	1	8	9	opioid receptor signaling pathway
**GO:0022402**	80.95	124	0.00014269	46	879	1026	cell cycle process
**GO:0009410**	0.64	15	0.00014269	3	10	13	response to xenobiotic stimulus

GO Term	ANG	Can	FDR	Unique	Max	Total	Description
**GO:1901800**	8.23	29	0.00014269	5	69	83	positive regulation of proteasomal protein catabolic process
**GO:0010468**	400.64	496	0.00014269	216	3368	4226	regulation of gene expression
**GO:0035728**	1.65	15	0.00014269	2	14	15	response to hepatocyte growth factor
**GO:0035729**	1.45	15	0.00014269	2	12	13	cellular response to hepatocyte growth factor stimulus
**GO:0060760**	6.46	27	0.00014269	7	41	54	positive regulation of response to cytokine stimulus
**GO:1901858**	1.79	12	0.00014269	3	5	6	regulation of mitochondrial DNA metabolic process
**GO:0071417**	63.67	99	0.00014269	35	354	413	cellular response to organonitrogen compound
**GO:0010498**	17.44	52	0.00014269	13	215	271	proteasomal protein catabolic process
**GO:0060759**	20.58	47	0.00014269	14	141	187	regulation of response to cytokine stimulus
**GO:0072738**	0.04	3	0.00014269	1	2	6	cellular response to diamide
**GO:0071407**	53.12	88	0.00014269	31	318	381	cellular response to organic cyclic compound
**GO:0072737**	0.04	3	0.00014269	1	2	6	response to diamide
**GO:1901796**	15.53	45	0.00014269	14	158	192	regulation of signal transduction by p53 class mediator
**GO:0009628**	117.93	167	0.00014269	59	881	1032	response to abiotic stimulus
**GO:0010629**	146.18	226	0.00014269	89	1179	1534	negative regulation of gene expression
**GO:0010604**	343.01	418	0.00014269	169	2491	3062	positive regulation of macromolecule metabolic process
**GO:0010605**	247.02	320	0.00014269	135	1949	2471	negative regulation of macromolecule metabolic process
**GO:0008239**	0.48	6	0.00014269	4	8	8	dipeptidyl-peptidase activity
**GO:0075044**	0.65	11	0.00014269	2	5	5	autophagy of host cells involved in interaction with symbiont
**GO:0035821**	11.60	36	0.00014269	11	112	150	modification of morphology or physiology of other organism
**GO:0060828**	33.30	65	0.00014269	16	209	261	regulation of canonical Wnt signaling pathway
**GO:0075071**	0.65	11	0.00014269	2	5	5	autophagy involved in symbiotic interaction
**GO:0051051**	58.22	94	0.00014269	33	390	506	negative regulation of transport
**GO:0033206**	1.70	11	0.00014269	2	6	6	meiotic cytokinesis
**GO:0010556**	365.84	474	0.00014269	197	3171	3951	regulation of macromolecule biosynthetic process
**GO:0010558**	138.71	215	0.00014269	85	1130	1458	negative regulation of macromolecule biosynthetic process
**GO:0008250**	1.25	11	0.00014269	3	9	11	oligosaccharyltransferase complex
**GO:0010564**	57.24	91	0.00014269	29	607	762	regulation of cell cycle process
**GO:0008298**	0.74	8	0.00014269	2	4	5	intracellular mRNA localization
**GO:0016717**	0.78	20	0.00014269	2	5	5	oxidoreductase activity, acting on paired donors, with oxidation of a pair of donors resulting in the reduction of molecular oxygen to two molecules of water
**GO:0016705**	17.45	55	0.00014269	12	128	145	oxidoreductase activity, acting on paired donors, with incorporation or reduction of molecular oxygen
**GO:1901724**	0.83	7	0.00014269	1	7	7	positive regulation of cell proliferation involved in kidney development
**GO:0051603**	28.28	70	0.00014269	23	346	446	proteolysis involved in cellular protein catabolic process
**GO:0036037**	0.30	10	0.00014269	2	6	11	CD8-positive, alpha-beta T cell activation
**GO:0000096**	3.46	15	0.00014269	2	29	33	sulfur amino acid metabolic process
**GO:0019511**	3.02	14	0.00014269	1	14	14	peptidyl-proline hydroxylation
**GO:0085020**	4.17	16	0.00014269	2	7	8	protein K6-linked ubiquitination
**GO:0007584**	14.48	39	0.00014269	15	141	158	response to nutrient
**GO:0006167**	0.43	6	0.00014269	1	5	6	AMP biosynthetic process
**GO:0044433**	118.86	170	0.00014269	73	952	1242	cytoplasmic vesicle part
**GO:0031111**	2.70	14	0.00014269	2	24	29	negative regulation of microtubule polymerization or depolymerization
**GO:0031114**	2.14	14	0.00014269	2	16	21	regulation of microtubule depolymerization
**GO:0032446**	58.43	106	0.00014269	34	654	799	protein modification by small protein conjugation
**GO:0021782**	6.05	19	0.00014269	3	39	56	glial cell development
**GO:0019471**	2.41	15	0.00014269	2	11	12	4-hydroxyproline metabolic process
**GO:0032436**	7.71	29	0.00014269	5	56	67	positive regulation of proteasomal ubiquitin-dependent protein catabolic process
**GO:1902075**	0.05	3	0.00014269	1	4	9	cellular response to salt
**GO:0032486**	1.06	11	0.00014269	2	9	10	Rap protein signal transduction
**GO:0045732**	19.10	48	0.00014269	12	222	282	positive regulation of protein catabolic process
**GO:0044403**	40.25	73	0.00014269	28	414	517	symbiosis, encompassing mutualism through parasitism
**GO:0031146**	3.80	25	0.00014269	3	61	85	SCF-dependent proteasomal ubiquitin-dependent protein catabolic process
**GO:0044419**	51.26	89	0.00014269	38	529	662	interspecies interaction between organisms
**GO:0006189**	0.23	6	0.00014269	1	6	7	'de novo' IMP biosynthetic process
**GO:0080135**	77.25	118	0.00014269	46	688	823	regulation of cellular response to stress

GO Term	ANG	Can	FDR	Unique	Max	Total	Description
**GO:0003950**	1.86	13	0.00014269	4	20	21	NAD+ ADP-ribosyltransferase activity
**GO:0043161**	16.89	51	0.00014269	12	206	261	proteasome-mediated ubiquitin-dependent protein catabolic process
**GO:0043177**	27.10	68	0.00014269	22	182	207	organic acid binding
**GO:0043186**	1.10	8	0.00014269	4	14	16	P granule
**GO:2000489**	0.63	12	0.00014269	1	6	6	regulation of hepatic stellate cell activation
**GO:0036109**	1.23	21	0.00014269	3	11	13	alpha-linolenic acid metabolic process
**GO:0051726**	90.28	132	0.00014269	47	904	1122	regulation of cell cycle
**GO:0045924**	1.06	13	0.00014269	1	4	6	regulation of female receptivity
**GO:1903364**	13.23	32	0.00014269	7	155	196	positive regulation of cellular protein catabolic process
**GO:0045935**	211.28	277	0.00014269	111	1534	1866	positive regulation of nucleobase-containing compound metabolic process
**GO:0045934**	137.85	217	0.00014269	87	1113	1448	negative regulation of nucleobase-containing compound metabolic process
**GO:0006355**	326.78	430	0.00014269	181	2755	3450	regulation of transcription, DNA-templated
**GO:0006357**	194.41	273	0.00014269	107	1510	1871	regulation of transcription from RNA polymerase II promoter
**GO:0045892**	118.74	182	0.00014269	74	921	1198	negative regulation of transcription, DNA-templated
**GO:0045893**	185.93	245	0.00014269	99	1270	1562	positive regulation of transcription, DNA-templated
**GO:0097067**	1.21	14	0.00014269	2	14	16	cellular response to thyroid hormone stimulus
**GO:0097066**	1.81	15	0.00014269	3	23	25	response to thyroid hormone
**GO:0097068**	0.51	12	0.00014269	1	5	5	response to thyroxine
**GO:0006271**	0.51	7	0.00014269	1	6	7	DNA strand elongation involved in DNA replication
**GO:0043200**	11.90	32	0.00014269	8	92	112	response to amino acid
**GO:0045862**	38.45	74	0.00014269	20	326	395	positive regulation of proteolysis
**GO:0051704**	103.43	160	0.00014269	65	1021	1321	multi-organism process
**GO:0051702**	7.04	22	0.00014269	5	55	75	interaction with symbiont
**GO:0043280**	13.96	35	0.00014269	8	105	130	positive regulation of cysteine-type endopeptidase activity involved in apoptotic process
**GO:0043281**	19.71	54	0.00014269	15	169	207	regulation of cysteine-type endopeptidase activity involved in apoptotic process
**GO:0000151**	15.52	47	0.00014269	11	177	202	ubiquitin ligase complex
**GO:0051409**	0.68	16	0.00014269	2	7	8	response to nitrosative stress
**GO:0002475**	0.61	6	0.00014269	1	5	17	antigen processing and presentation via MHC class Ib
**GO:1903265**	3.81	16	0.00014269	3	6	6	positive regulation of tumor necrosis factor-mediated signaling pathway
**GO:0009991**	34.91	83	0.00014269	27	333	398	response to extracellular stimulus
**GO:1903203**	5.99	20	0.00014269	4	20	23	regulation of oxidative stress-induced neuron death
**GO:1903204**	5.72	20	0.00014269	4	15	18	negative regulation of oxidative stress-induced neuron death
**GO:0010972**	5.28	22	0.00014269	2	72	97	negative regulation of G2/M transition of mitotic cell cycle
**GO:0007341**	0.56	9	0.00014269	2	7	7	penetration of zona pellucida
**GO:0008641**	0.57	7	0.00014269	1	6	9	small protein activating enzyme activity
**GO:0019219**	373.07	500	0.00014269	209	3212	3995	regulation of nucleobase-containing compound metabolic process
**GO:0009892**	272.78	350	0.00014269	145	2166	2737	negative regulation of metabolic process
**GO:0009890**	147.13	225	0.00014269	87	1200	1544	negative regulation of biosynthetic process
**GO:0009896**	34.78	66	0.00014269	17	363	435	positive regulation of catabolic process
**GO:0009894**	80.73	129	0.00014269	47	713	853	regulation of catabolic process
**GO:0007292**	4.32	17	0.00014269	2	37	43	female gamete generation
**GO:0046827**	1.31	11	0.00014269	3	17	20	positive regulation of protein export from nucleus
**GO:0007276**	32.00	62	0.00014269	25	332	447	gamete generation
**GO:0033554**	131.91	185	0.00014269	65	1226	1511	cellular response to stress
**GO:0033559**	7.70	37	0.00014269	12	89	106	unsaturated fatty acid metabolic process
**GO:0007281**	16.69	38	0.00014269	11	123	140	germ cell development
**GO:0032291**	0.47	7	0.00014269	1	8	12	axon ensheathment in central nervous system
**GO:0070555**	10.06	27	0.00014269	7	60	85	response to interleukin-1
**GO:0003700**	98.48	143	0.00014269	55	787	987	transcription factor activity, sequence-specific DNA binding
**GO:0002438**	0.94	8	0.00014269	1	7	8	acute inflammatory response to antigenic stimulus
**GO:0044248**	94.62	147	0.00014269	62	1038	1282	cellular catabolic process
**GO:0044265**	40.92	77	0.00014269	28	555	727	cellular macromolecule catabolic process
**GO:0070534**	6.48	26	0.00014269	4	27	42	protein K63-linked ubiquitination
**GO:0080090**	565.21	671	0.00014269	292	4699	5776	regulation of primary metabolic process
**GO:0051580**	5.02	17	0.00014269	3	12	14	regulation of neurotransmitter uptake
**GO:0045649**	3.13	13	0.00014269	2	17	20	regulation of macrophage differentiation
**GO:0046974**	0.43	6	0.00014269	1	4	10	histone methyltransferase activity (H3-K9 specific)
**GO:0046976**	0.43	6	0.00014269	1	3	8	histone methyltransferase activity (H3-K27 specific)
**GO:0045651**	2.64	13	0.00014269	2	10	13	positive regulation of macrophage differentiation

GO Term	ANG	Can	FDR	Unique	Max	Total	Description
**GO:0044344**	2.14	15	0.00014269	3	22	30	cellular response to fibroblast growth factor stimulus
**GO:0006082**	94.68	155	0.00014269	62	838	942	organic acid metabolic process
**GO:1903046**	10.01	31	0.00014269	10	120	139	meiotic cell cycle process
**GO:1904386**	0.51	12	0.00014269	1	5	5	response to L-phenylalanine derivative
**GO:1903052**	12.62	32	0.00014269	7	143	181	positive regulation of proteolysis involved in cellular protein catabolic process
**GO:0032354**	3.35	16	0.00014269	3	16	17	response to follicle-stimulating hormone
**GO:0019373**	0.63	7	0.00014269	3	11	11	epoxygenase P450 pathway
**GO:0031047**	3.65	20	0.00014269	5	40	53	gene silencing by RNA
**GO:0070647**	71.84	121	0.00014269	44	815	1020	protein modification by small protein conjugation or removal
**GO:0042340**	0.82	7	0.00026985	1	11	11	keratan sulfate catabolic process
**GO:0097202**	12.70	30	0.00026985	6	81	94	activation of cysteine-type endopeptidase activity
**GO:0031329**	65.26	105	0.00026985	40	614	739	regulation of cellular catabolic process
**GO:0019842**	11.45	28	0.00026985	7	76	82	vitamin binding
**GO:0042176**	41.77	73	0.00026985	21	350	435	regulation of protein catabolic process
**GO:0035547**	0.32	5	0.00026985	1	4	6	regulation of interferon-beta secretion
**GO:0016595**	2.79	12	0.00026985	1	10	10	glutamate binding
**GO:0030662**	15.07	34	0.00026985	11	111	183	coated vesicle membrane
**GO:0030666**	16.12	36	0.00026985	11	123	225	endocytic vesicle membrane
**GO:0030665**	11.40	28	0.00026985	8	76	126	clathrin-coated vesicle membrane
**GO:0042692**	14.65	35	0.00026985	13	80	90	muscle cell differentiation
**GO:0046631**	7.49	21	0.00026985	4	34	43	alpha-beta T cell activation
**GO:0051303**	3.86	14	0.00026985	2	58	62	establishment of chromosome localization
**GO:0046479**	0.79	7	0.00026985	1	8	10	glycosphingolipid catabolic process
**GO:0005922**	0.34	5	0.00026985	2	14	21	connexon complex
**GO:0046514**	0.83	7	0.00026985	1	9	11	ceramide catabolic process
**GO:0018126**	3.74	14	0.00026985	1	23	23	protein hydroxylation
**GO:0043154**	4.98	16	0.00026985	6	65	79	negative regulation of cysteine-type endopeptidase activity involved in apoptotic process
**GO:0000178**	0.70	7	0.00026985	2	16	18	exosome (RNase complex)
**GO:1903313**	3.50	15	0.00026985	3	41	48	positive regulation of mRNA metabolic process
**GO:0007631**	7.79	22	0.00026985	8	61	81	feeding behavior
**GO:0046914**	119.97	165	0.00026985	63	887	1091	transition metal ion binding
**GO:0019377**	0.81	7	0.00026985	1	10	12	glycolipid catabolic process
**GO:0016053**	25.13	49	0.00037914	18	215	253	organic acid biosynthetic process
**GO:0098852**	33.43	59	0.00037914	29	299	411	lytic vacuole membrane
**GO:0098858**	30.08	56	0.00037914	16	117	139	actin-based cell projection
**GO:1903708**	18.03	37	0.00037914	13	136	182	positive regulation of hemopoiesis
**GO:0036295**	1.18	8	0.00037914	2	11	14	cellular response to increased oxygen levels
**GO:1902305**	11.71	29	0.00037914	7	42	46	regulation of sodium ion transmembrane transport
**GO:0098588**	267.98	335	0.00037914	153	2150	2630	bounding membrane of organelle
**GO:1901028**	3.29	13	0.00037914	6	41	47	regulation of mitochondrial outer membrane permeabilization involved in apoptotic signaling pathway
**GO:0031440**	1.86	11	0.00037914	2	27	34	regulation of mRNA 3'-end processing
**GO:0002933**	0.16	4	0.00037914	1	6	7	lipid hydroxylation
**GO:0005765**	33.43	59	0.00037914	29	299	411	lysosomal membrane
**GO:0042698**	3.58	14	0.00037914	5	22	22	ovulation cycle
**GO:0046394**	25.13	49	0.00037914	18	215	253	carboxylic acid biosynthetic process
**GO:0046329**	5.99	18	0.00037914	2	31	32	negative regulation of JNK cascade
**GO:0005506**	16.24	37	0.00037914	11	126	159	iron ion binding
**GO:0072330**	16.72	35	0.00037914	11	140	168	monocarboxylic acid biosynthetic process
**GO:1902749**	16.66	35	0.00037914	8	178	222	regulation of cell cycle G2/M phase transition
**GO:0030539**	2.67	12	0.00037914	3	14	17	male genitalia development
**GO:1902741**	0.41	5	0.00037914	1	6	7	positive regulation of interferon-alpha secretion
**GO:1902739**	0.41	5	0.00037914	1	6	7	regulation of interferon-alpha secretion
**GO:0042593**	12.50	31	0.00037914	11	141	153	glucose homeostasis
**GO:1902617**	0.75	7	0.00037914	1	5	5	response to fluoride
**GO:0071673**	1.01	7	0.00037914	1	3	8	positive regulation of smooth muscle cell chemotaxis
**GO:1900748**	2.24	11	0.00037914	2	5	5	positive regulation of vascular endothelial growth factor signaling pathway
**GO:0010803**	6.89	20	0.00037914	5	47	62	regulation of tumor necrosis factor-mediated signaling pathway
**GO:0034453**	3.26	13	0.00037914	2	37	40	microtubule anchoring
**GO:0005930**	11.58	28	0.00037914	10	71	74	axoneme
**GO:0044446**	870.48	979	0.00037914	460	7410	9166	intracellular organelle part
**GO:0033500**	12.50	31	0.00037914	11	141	153	carbohydrate homeostasis
**GO:0044283**	38.02	67	0.00037914	30	383	456	small molecule biosynthetic process
**GO:0043069**	86.25	126	0.00037914	54	707	876	negative regulation of programmed cell death
**GO:0050765**	1.00	7	0.0004864	2	15	16	negative regulation of phagocytosis
**GO:0035278**	1.16	8	0.0004864	1	11	11	miRNA mediated inhibition of translation

GO Term	ANG	Can	FDR	Unique	Max	Total	Description
**GO:1901163**	0.43	5	0.0004864	1	9	11	regulation of trophoblast cell migration
**GO:0031325**	339.76	412	0.0004864	168	2502	3044	positive regulation of cellular metabolic process
**GO:0031323**	575.05	666	0.0004864	296	4750	5846	regulation of cellular metabolic process
**GO:0045974**	1.16	8	0.0004864	1	11	11	regulation of translation, ncRNA-mediated
**GO:0097179**	0.51	7	0.0004864	3	5	5	protease inhibitor complex
**GO:0040033**	1.16	8	0.0004864	1	11	11	negative regulation of translation, ncRNA-mediated
**GO:0097677**	0.38	5	0.0004864	2	5	6	STAT family protein binding
**GO:0060487**	3.20	13	0.0004864	1	19	20	lung epithelial cell differentiation
**GO:0010822**	17.36	36	0.0004864	12	153	184	positive regulation of mitochondrion organization
**GO:0007223**	7.23	20	0.0004864	4	34	36	Wnt signaling pathway, calcium modulating pathway
**GO:1901988**	13.94	31	0.0004864	8	183	229	negative regulation of cell cycle phase transition
**GO:0015301**	2.30	11	0.0004864	4	22	25	anion:anion antiporter activity
**GO:0009408**	9.51	25	0.0004864	4	80	100	response to heat
**GO:0009636**	14.56	32	0.0004864	11	120	140	response to toxic substance
**GO:0032395**	0.85	7	0.0004864	3	7	53	MHC class II receptor activity
**GO:0005452**	1.62	9	0.00059176	3	15	15	inorganic anion exchanger activity
**GO:0048522**	604.78	693	0.00059176	288	4172	5143	positive regulation of cellular process
**GO:2001020**	12.61	29	0.00059176	13	153	184	regulation of response to DNA damage stimulus
**GO:0045060**	2.72	12	0.00059176	3	10	10	negative thymic T cell selection
**GO:1902808**	4.69	15	0.00059176	4	28	40	positive regulation of cell cycle G1/S phase transition
**GO:0022616**	0.90	7	0.00059176	1	11	12	DNA strand elongation
**GO:0032095**	1.29	8	0.00059176	1	14	18	regulation of response to food
**GO:0008542**	6.22	19	0.00059176	4	45	50	visual learning
**GO:0050000**	4.09	14	0.00059176	2	60	64	chromosome localization
**GO:0010765**	6.02	18	0.00059176	4	26	29	positive regulation of sodium ion transport
**GO:0044422**	909.97	1015	0.00059176	475	7619	9401	organelle part
**GO:1903202**	7.69	20	0.00059176	4	36	42	negative regulation of oxidative stress-induced cell death
**GO:0008654**	22.61	44	0.00059176	17	226	281	phospholipid biosynthetic process
**GO:0003714**	21.26	42	0.00059176	14	171	205	transcription corepressor activity
**GO:0061458**	3.04	12	0.000685	3	18	21	reproductive system development
**GO:0006996**	225.41	284	0.000685	117	1910	2253	organelle organization
**GO:0007005**	24.70	46	0.000685	15	234	286	mitochondrion organization
**GO:0010458**	0.88	7	0.000685	1	10	11	exit from mitosis
**GO:0005902**	8.57	22	0.000685	5	51	60	microvillus
**GO:0038092**	0.66	6	0.000685	2	7	9	nodal signaling pathway
**GO:1900408**	7.70	20	0.000685	4	37	44	negative regulation of cellular response to oxidative stress
**GO:0007520**	6.07	18	0.000685	3	17	19	myoblast fusion
**GO:0019369**	3.03	12	0.000685	6	39	42	arachidonic acid metabolic process
**GO:0031638**	13.32	30	0.000685	6	98	114	zymogen activation
**GO:1903747**	15.65	34	0.000685	11	113	140	regulation of establishment of protein localization to mitochondrion
**GO:0015296**	5.23	16	0.000685	4	39	42	anion:cation symporter activity
**GO:0051173**	223.14	280	0.000685	114	1626	1985	positive regulation of nitrogen compound metabolic process
**GO:0008186**	0.95	7	0.000685	3	22	34	RNA-dependent ATPase activity
**GO:0002162**	1.02	7	0.000685	1	7	9	dystroglycan binding
**GO:0043383**	2.95	12	0.00090392	3	11	12	negative T cell selection
**GO:0009056**	135.78	185	0.00090392	86	1327	1630	catabolic process
**GO:0000777**	6.23	18	0.00090392	7	80	91	condensed chromosome kinetochore
**GO:0044437**	51.04	80	0.00090392	41	471	641	vacuolar part
**GO:0070848**	42.66	70	0.00090392	28	250	283	response to growth factor
**GO:0097062**	2.61	11	0.00090392	1	10	10	dendritic spine maintenance
**GO:0051879**	3.95	14	0.00100244	6	29	33	Hsp90 protein binding
**GO:0070987**	1.06	7	0.00100244	1	18	19	error-free translesion synthesis
**GO:0031324**	245.53	306	0.00100244	134	1970	2493	negative regulation of cellular metabolic process
**GO:0035032**	0.44	5	0.00100244	1	5	5	phosphatidylinositol 3-kinase complex, class III
**GO:0043900**	39.07	64	0.00100244	27	358	466	regulation of multi-organism process
**GO:1900736**	0.73	6	0.00100244	1	5	6	regulation of phospholipase C-activating G-protein coupled receptor signaling pathway
**GO:0002042**	3.18	12	0.00100244	3	15	16	cell migration involved in sprouting angiogenesis
**GO:0070734**	0.73	6	0.00100244	1	9	16	histone H3-K27 methylation
**GO:0048149**	1.49	8	0.00100244	1	8	9	behavioral response to ethanol
**GO:0008610**	54.24	84	0.00100244	35	485	594	lipid biosynthetic process
**GO:1902308**	0.45	5	0.00110314	1	4	6	regulation of peptidyl-serine dephosphorylation
**GO:0045061**	3.40	13	0.00110314	4	17	17	thymic T cell selection
**GO:0002039**	6.72	19	0.00110314	8	54	74	p53 binding
**GO:0071547**	0.46	5	0.00110314	2	5	5	piP-body
**GO:0044249**	358.69	430	0.00110314	192	3473	4277	cellular biosynthetic process

GO Term	ANG	Can	FDR	Unique	Max	Total	Description
**GO:0017144**	2.82	12	0.00120093	4	31	35	drug metabolic process
**GO:0015294**	10.46	25	0.00120093	8	79	86	solute:cation symporter activity
**GO:0034124**	0.11	3	0.00120093	1	4	5	regulation of MyD88-dependent toll-like receptor signaling pathway
**GO:1901797**	4.71	15	0.00120093	4	23	32	negative regulation of signal transduction by p53 class mediator
**GO:0031110**	5.61	16	0.00120093	3	56	67	regulation of microtubule polymerization or depolymerization
**GO:0046825**	2.71	11	0.00120093	3	27	31	regulation of protein export from nucleus
**GO:1901030**	3.19	12	0.0012761	5	35	38	positive regulation of mitochondrial outer membrane permeabilization involved in apoptotic signaling pathway
**GO:0071260**	7.28	19	0.0012761	5	70	75	cellular response to mechanical stimulus
**GO:0090241**	0.28	4	0.0012761	1	5	5	negative regulation of histone H4 acetylation
**GO:0010628**	225.28	280	0.0012761	116	1578	1954	positive regulation of gene expression
**GO:0009607**	51.36	80	0.0012761	35	604	810	response to biotic stimulus
**GO:0031214**	5.87	17	0.0012761	5	62	74	biomineral tissue development
**GO:0043014**	4.84	15	0.0012761	4	24	29	alpha-tubulin binding
**GO:0042276**	1.07	7	0.0012761	1	17	18	error-prone translesion synthesis
**GO:0043566**	6.62	18	0.0012761	3	65	86	structure-specific DNA binding
**GO:0051865**	7.45	20	0.0012761	5	47	55	protein autoubiquitination
**GO:0030027**	36.68	61	0.0012761	18	152	175	lamellipodium
**GO:0051701**	16.87	34	0.0012761	15	130	151	interaction with host
**GO:0043066**	83.43	119	0.0012761	53	695	863	negative regulation of apoptotic process
**GO:0043679**	8.56	22	0.00135519	6	43	52	axon terminus
**GO:0015185**	0.74	6	0.00135519	1	4	5	gamma-aminobutyric acid transmembrane transporter activity
**GO:0005829**	547.77	630	0.00135519	268	4208	5076	cytosol
**GO:0019079**	3.25	12	0.00135519	4	21	25	viral genome replication
**GO:0032094**	1.41	8	0.00135519	1	13	16	response to food
**GO:0003677**	189.14	241	0.00135519	104	1872	2326	DNA binding
**GO:0018208**	4.39	14	0.00135519	1	47	50	peptidyl-proline modification
**GO:2000649**	10.01	24	0.00135519	6	31	35	regulation of sodium ion transmembrane transporter activity
**GO:0045944**	126.40	168	0.00135519	67	881	1087	positive regulation of transcription from RNA polymerase II promoter
**GO:0010948**	18.85	37	0.00135519	9	255	339	negative regulation of cell cycle process
**GO:0014874**	2.83	11	0.00144495	3	14	15	response to stimulus involved in regulation of muscle adaptation
**GO:0050684**	6.82	19	0.00144495	7	91	114	regulation of mRNA processing
**GO:0032873**	6.56	18	0.00144495	2	39	41	negative regulation of stress-activated MAPK cascade
**GO:0030877**	0.79	6	0.00144495	3	9	10	beta-catenin destruction complex
**GO:0070303**	6.56	18	0.00144495	2	39	41	negative regulation of stress-activated protein kinase signaling cascade
**GO:0010557**	204.20	256	0.00144495	104	1476	1821	positive regulation of macromolecule biosynthetic process
**GO:0051589**	5.43	16	0.00144495	2	12	14	negative regulation of neurotransmitter transport
**GO:0070989**	1.47	8	0.00165073	4	11	13	oxidative demethylation
**GO:0023057**	159.47	205	0.00165073	72	1059	1262	negative regulation of signaling
**GO:0016725**	0.50	5	0.00165073	2	8	8	oxidoreductase activity, acting on CH or CH2 groups
**GO:0018024**	6.57	18	0.00165073	4	39	45	histone-lysine N-methyltransferase activity
**GO:0072124**	1.13	7	0.00174155	1	8	8	regulation of glomerular mesangial cell proliferation
**GO:0060136**	0.11	3	0.00174155	1	4	6	embryonic process involved in female pregnancy
**GO:1902254**	4.26	14	0.00174155	3	15	22	negative regulation of intrinsic apoptotic signaling pathway by p53 class mediator
**GO:0042755**	3.31	12	0.00174155	3	22	27	eating behavior
**GO:0044306**	10.05	24	0.00174155	8	57	68	neuron projection terminus
**GO:0005243**	0.27	4	0.00181965	1	8	12	gap junction channel activity
**GO:0010648**	161.36	207	0.00181965	74	1075	1281	negative regulation of cell communication
**GO:0071456**	17.03	34	0.00181965	12	99	106	cellular response to hypoxia
**GO:0007632**	7.10	19	0.00181965	4	50	55	visual behavior
**GO:1903320**	23.56	43	0.00181965	13	255	316	regulation of protein modification by small protein conjugation or removal
**GO:0007346**	46.64	73	0.00181965	24	484	602	regulation of mitotic cell cycle
**GO:0033599**	1.98	9	0.00181965	1	12	16	regulation of mammary gland epithelial cell proliferation
**GO:0018026**	0.84	6	0.00181965	1	9	9	peptidyl-lysine monomethylation
**GO:0048490**	0.83	6	0.0019009	1	13	16	anterograde synaptic vesicle transport
**GO:0003229**	0.84	6	0.0019009	2	5	6	ventricular cardiac muscle tissue development
**GO:0016879**	6.02	17	0.0019009	5	47	51	ligase activity, forming carbon-nitrogen bonds

GO Term	ANG	Can	FDR	Unique	Max	Total	Description
**GO:1900740**	2.84	11	0.0019009	4	28	30	positive regulation of protein insertion into mitochondrial membrane involved in apoptotic signaling pathway
**GO:1900739**	2.84	11	0.0019009	4	28	30	regulation of protein insertion into mitochondrial membrane involved in apoptotic signaling pathway
**GO:1904781**	1.17	7	0.0019009	2	5	6	positive regulation of protein localization to centrosome
**GO:0032434**	16.39	33	0.0019009	7	108	133	regulation of proteasomal ubiquitin-dependent protein catabolic process
**GO:0055093**	1.54	8	0.0019009	2	18	23	response to hyperoxia
**GO:2000104**	2.43	10	0.00198869	2	16	19	negative regulation of DNA-dependent DNA replication
**GO:0097237**	5.73	16	0.00198869	5	22	25	cellular response to toxic substance
**GO:1902253**	4.32	14	0.00198869	3	20	28	regulation of intrinsic apoptotic signaling pathway by p53 class mediator
**GO:0052547**	39.97	65	0.00198869	22	328	422	regulation of peptidase activity
**GO:0035865**	0.55	5	0.00198869	2	8	8	cellular response to potassium ion
**GO:0045639**	9.87	23	0.00198869	7	67	87	positive regulation of myeloid cell differentiation
**GO:1901570**	2.42	10	0.00206708	4	29	38	fatty acid derivative biosynthetic process
**GO:0046456**	2.42	10	0.00206708	4	29	38	icosanoid biosynthetic process
**GO:0007275**	38.93	63	0.00206708	27	350	427	multicellular organismal development
**GO:0044281**	179.77	230	0.00206708	107	1508	1733	small molecule metabolic process
**GO:0046040**	0.83	6	0.00214836	1	9	11	IMP metabolic process
**GO:0001967**	1.27	7	0.00214836	3	11	13	suckling behavior
**GO:0007200**	7.50	19	0.00214836	5	46	51	phospholipase C-activating G-protein coupled receptor signaling pathway
**GO:0006188**	0.83	6	0.00214836	1	9	11	IMP biosynthetic process
**GO:0032201**	1.60	8	0.00214836	2	24	25	telomere maintenance via semi-conservative replication
**GO:0035194**	1.56	8	0.00221991	1	17	17	posttranscriptional gene silencing by RNA
**GO:0098531**	11.84	26	0.00221991	6	45	54	transcription factor activity, direct ligand regulated sequence-specific DNA binding
**GO:0043312**	38.58	62	0.00221991	30	419	548	neutrophil degranulation
**GO:0006505**	1.22	7	0.00221991	2	18	24	GPI anchor metabolic process
**GO:0005774**	35.74	59	0.00221991	29	322	437	vacuolar membrane
**GO:0009058**	378.97	448	0.00221991	206	3634	4479	biosynthetic process
**GO:0004879**	11.84	26	0.00221991	6	45	54	RNA polymerase II transcription factor activity, ligand-activated sequence-specific DNA binding
**GO:1903311**	8.50	21	0.00221991	9	108	134	regulation of mRNA metabolic process
**GO:0031396**	21.52	40	0.00230055	10	232	292	regulation of protein ubiquitination
**GO:1901983**	6.00	17	0.00230055	5	52	66	regulation of protein acetylation
**GO:0001829**	0.59	5	0.00233693	2	12	22	trophectodermal cell differentiation
**GO:0030207**	1.25	7	0.00233693	1	12	14	chondroitin sulfate catabolic process
**GO:0030120**	3.44	12	0.00233693	2	35	35	vesicle coat
**GO:0006986**	6.64	18	0.00233693	6	43	55	response to unfolded protein
**GO:1904259**	1.21	7	0.00233693	1	5	5	regulation of basement membrane assembly involved in embryonic body morphogenesis
**GO:1904261**	1.21	7	0.00233693	1	5	5	positive regulation of basement membrane assembly involved in embryonic body morphogenesis
**GO:0016840**	0.73	6	0.00233693	1	9	9	carbon-nitrogen lyase activity
**GO:0045180**	1.26	7	0.00233693	1	5	5	basal cortex
**GO:0045744**	3.34	12	0.00233693	3	26	31	negative regulation of G-protein coupled receptor protein signaling pathway
**GO:0071229**	23.01	42	0.00233693	11	163	190	cellular response to acid chemical
**GO:0031996**	0.53	5	0.00233693	2	9	11	thioesterase binding
**GO:1900746**	2.90	11	0.00233693	2	13	15	regulation of vascular endothelial growth factor signaling pathway
**GO:0008970**	0.86	6	0.00233693	3	9	9	phosphatidylcholine 1-acylhydrolase activity
**GO:0061136**	17.84	35	0.00233693	8	138	170	regulation of proteasomal protein catabolic process
**GO:1903146**	6.14	17	0.00233693	5	38	43	regulation of mitophagy
**GO:0003823**	3.44	12	0.00233693	4	50	155	antigen binding
**GO:0090032**	0.52	5	0.00248159	1	5	5	negative regulation of steroid hormone biosynthetic process
**GO:0051881**	7.31	19	0.00248159	5	41	53	regulation of mitochondrial membrane potential
**GO:0044699**	1359.12	1467	0.00248159	670	9732	11942	single-organism process
**GO:0035631**	0.57	5	0.00248159	2	11	11	CD40 receptor complex
**GO:0031944**	0.52	5	0.00248159	1	5	5	negative regulation of glucocorticoid metabolic process
**GO:0031947**	0.52	5	0.00248159	1	5	5	negative regulation of glucocorticoid biosynthetic process

GO Term	ANG	Can	FDR	Unique	Max	Total	Description
**GO:0007191**	1.63	8	0.00248159	1	7	8	adenylate cyclase-activating dopamine receptor signaling pathway
**GO:0045930**	18.95	36	0.00248159	11	224	292	negative regulation of mitotic cell cycle
**GO:0006282**	7.96	20	0.00248159	8	82	90	regulation of DNA repair
**GO:0022898**	47.64	73	0.00248159	23	180	200	regulation of transmembrane transporter activity
**GO:0032098**	1.63	8	0.00248159	1	16	20	regulation of appetite
**GO:0032412**	46.82	72	0.00248159	22	174	194	regulation of ion transmembrane transporter activity
**GO:2000064**	0.55	5	0.0026263	1	6	6	regulation of cortisol biosynthetic process
**GO:0045055**	41.33	65	0.0026263	31	447	584	regulated secretory pathway
**GO:0000731**	5.17	15	0.0026263	3	36	37	DNA synthesis involved in DNA repair
**GO:0000086**	12.20	26	0.0026263	5	117	139	G2/M transition of mitotic cell cycle
**GO:0016491**	65.58	96	0.0026263	32	573	687	oxidoreductase activity
**GO:0051017**	8.17	20	0.0026263	6	39	48	actin filament bundle assembly
**GO:0009968**	144.38	186	0.0026263	68	966	1153	negative regulation of signal transduction
**GO:0010952**	21.86	40	0.0026263	11	145	175	positive regulation of peptidase activity
**GO:0032727**	0.93	6	0.00278752	2	17	21	positive regulation of interferon-alpha production
**GO:0035966**	6.89	18	0.00278752	6	47	62	response to topologically incorrect protein
**GO:0002028**	15.43	31	0.00278752	9	63	67	regulation of sodium ion transport
**GO:0005921**	0.84	6	0.00278752	1	14	18	gap junction
**GO:0007517**	17.36	34	0.00278752	11	98	109	muscle organ development
**GO:1904779**	1.25	7	0.00278752	2	6	7	regulation of protein localization to centrosome
**GO:0052548**	38.41	62	0.00286484	20	311	401	regulation of endopeptidase activity
**GO:0071295**	2.10	9	0.00294056	4	18	20	cellular response to vitamin
**GO:0080134**	148.71	192	0.00294056	83	1294	1633	regulation of response to stress
**GO:1903201**	8.35	20	0.00294056	4	50	59	regulation of oxidative stress-induced cell death
**GO:0043534**	4.23	13	0.0030032	4	19	25	blood vessel endothelial cell migration
**GO:0009065**	2.24	9	0.0030032	3	21	28	glutamine family amino acid catabolic process
**GO:0042613**	1.15	7	0.0030032	3	11	77	MHC class II protein complex
**GO:0010463**	1.67	8	0.0030032	4	15	17	mesenchymal cell proliferation
**GO:0009605**	129.18	170	0.0030032	69	1061	1330	response to external stimulus
**GO:0036294**	17.51	34	0.00307962	12	105	113	cellular response to decreased oxygen levels
**GO:0042054**	7.44	19	0.00307962	5	51	57	histone methyltransferase activity
**GO:0016571**	9.42	22	0.00307962	6	80	91	histone methylation
**GO:0051705**	22.95	41	0.00307962	9	64	83	multi-organism behavior
**GO:0045648**	1.77	8	0.00307962	3	20	25	positive regulation of erythrocyte differentiation
**GO:0036296**	1.72	8	0.00315947	2	23	30	response to increased oxygen levels
**GO:0044839**	12.27	26	0.00315947	5	119	141	cell cycle G2/M phase transition
**GO:0031644**	14.51	29	0.00322793	7	62	72	regulation of neurological system process
**GO:0031527**	3.59	12	0.00322793	3	14	20	filopodium membrane
**GO:2001258**	5.32	15	0.00322793	3	26	33	negative regulation of cation channel activity
**GO:0090193**	1.28	7	0.00322793	1	8	10	positive regulation of glomerulus development
**GO:0000768**	7.09	18	0.00322793	3	24	28	syncytium formation by plasma membrane fusion
**GO:0008374**	4.64	14	0.00322793	7	41	65	O-acyltransferase activity
**GO:2000117**	5.97	16	0.00329667	6	70	86	negative regulation of cysteine-type endopeptidase activity
**GO:0006487**	4.15	13	0.00329667	4	43	50	protein N-linked glycosylation
**GO:2001056**	18.33	35	0.00329667	8	120	145	positive regulation of cysteine-type endopeptidase activity
**GO:0003013**	17.01	33	0.00329667	9	113	133	circulatory system process
**GO:0042060**	12.94	27	0.00335858	13	70	78	wound healing
**GO:1901698**	96.39	131	0.00335858	50	635	749	response to nitrogen compound
**GO:0002283**	39.23	62	0.00335858	30	423	555	neutrophil activation involved in immune response
**GO:0051145**	1.32	7	0.00335858	3	19	19	smooth muscle cell differentiation
**GO:0070507**	14.55	29	0.00335858	7	145	168	regulation of microtubule cytoskeleton organization
**GO:0031090**	322.11	383	0.00335858	179	2745	3362	organelle membrane
**GO:0043524**	17.73	34	0.00342582	10	114	134	negative regulation of neuron apoptotic process
**GO:1901575**	118.49	157	0.00342582	74	1217	1501	organic substance catabolic process
**GO:0031005**	3.14	11	0.00342582	3	12	13	filamin binding
**GO:0061512**	2.19	9	0.00348124	3	22	24	protein localization to cilium
**GO:0030008**	2.24	9	0.00348124	2	10	11	TRAPP complex
**GO:0006949**	7.18	18	0.00348124	3	25	29	syncytium formation
**GO:0009597**	0.61	5	0.00348124	1	5	5	detection of virus
**GO:1901722**	1.31	7	0.00348124	1	12	12	regulation of cell proliferation involved in kidney development
**GO:0061061**	17.76	34	0.00348124	11	100	111	muscle structure development
**GO:0033655**	0.96	6	0.00348124	1	5	5	host cell cytoplasm part
**GO:0000785**	26.59	46	0.00357115	23	271	337	chromatin
**GO:0016604**	75.67	107	0.00357115	47	638	810	nuclear body
**GO:0030149**	1.36	7	0.00365548	1	16	19	sphingolipid catabolic process
**GO:0018027**	0.97	6	0.00365548	1	12	18	peptidyl-lysine dimethylation
**GO:0044728**	6.06	16	0.00371956	7	56	72	DNA methylation or demethylation

GO Term	ANG	Can	FDR	Unique	Max	Total	Description
**GO:0030119**	2.18	9	0.00371956	2	33	37	AP-type membrane coat adaptor complex
**GO:0016416**	0.37	4	0.00371956	1	5	5	O-palmitoyltransferase activity
**GO:0061013**	2.65	10	0.00371956	3	24	29	regulation of mRNA catabolic process
**GO:0098743**	2.69	10	0.00378529	1	18	20	cell aggregation
**GO:0001502**	2.68	10	0.00378529	1	17	19	cartilage condensation
**GO:0007020**	1.32	7	0.00378529	1	14	16	microtubule nucleation
**GO:0033646**	0.99	6	0.00378529	1	6	7	host intracellular part
**GO:0048245**	0.36	4	0.00391525	1	7	12	eosinophil chemotaxis
**GO:1901606**	9.97	22	0.00391525	10	91	102	alpha-amino acid catabolic process
**GO:0005643**	2.76	10	0.00391525	5	43	47	nuclear pore
**GO:0031946**	0.62	5	0.00391525	1	7	7	regulation of glucocorticoid biosynthetic process
**GO:0032353**	0.62	5	0.00391525	1	7	7	negative regulation of hormone biosynthetic process
**GO:0003073**	4.34	13	0.00398586	3	22	32	regulation of systemic arterial blood pressure
**GO:0032409**	49.16	74	0.00398586	24	193	213	regulation of transporter activity
**GO:0034620**	5.34	15	0.00405521	3	7	11	cellular response to unfolded protein
**GO:1902589**	155.92	198	0.00411391	82	1342	1599	single-organism organelle organization
**GO:0006305**	4.39	13	0.00411391	5	43	57	DNA alkylation
**GO:0006306**	4.39	13	0.00411391	5	43	57	DNA methylation
**GO:0044238**	835.91	925	0.00411391	446	7207	8768	primary metabolic process
**GO:0042339**	2.75	10	0.00414149	3	31	32	keratan sulfate metabolic process
**GO:0031943**	0.64	5	0.00414149	1	8	9	regulation of glucocorticoid metabolic process
**GO:0032109**	9.06	21	0.00414149	4	58	63	positive regulation of response to nutrient levels
**GO:0032106**	9.06	21	0.00414149	4	58	63	positive regulation of response to extracellular stimulus
**GO:0010519**	1.35	7	0.00414149	2	6	7	negative regulation of phospholipase activity
**GO:0051567**	0.97	6	0.00414149	1	9	15	histone H3-K9 methylation
**GO:0043531**	4.30	13	0.00414149	2	28	38	ADP binding
**GO:0051716**	276.13	330	0.00414149	138	2198	2655	cellular response to stimulus
**GO:0007254**	4.91	14	0.00414149	3	42	57	JNK cascade
**GO:0014877**	2.30	9	0.00426545	2	9	10	response to muscle inactivity involved in regulation of muscle adaptation
**GO:0014894**	2.30	9	0.00426545	2	9	10	response to denervation involved in regulation of muscle adaptation
**GO:1902230**	1.39	7	0.00426545	3	23	31	negative regulation of intrinsic apoptotic signaling pathway in response to DNA damage
**GO:0009267**	10.50	23	0.00426545	6	112	136	cellular response to starvation
**GO:1900034**	6.10	16	0.00426545	6	74	80	regulation of cellular response to heat
**GO:0015929**	1.42	7	0.00426545	1	13	15	hexosaminidase activity
**GO:0032232**	7.51	18	0.00426545	2	19	22	negative regulation of actin filament bundle assembly
**GO:0044255**	102.97	139	0.00426545	68	821	984	cellular lipid metabolic process
**GO:0006506**	0.97	6	0.00432556	1	17	21	GPI anchor biosynthetic process
**GO:0010035**	53.25	79	0.00432556	29	389	465	response to inorganic substance
**GO:0007224**	8.48	20	0.00432556	7	60	69	smoothened signaling pathway
**GO:2001243**	7.97	19	0.00438999	5	74	90	negative regulation of intrinsic apoptotic signaling pathway
**GO:0035770**	14.01	28	0.00438999	10	152	199	ribonucleoprotein granule
**GO:1990511**	0.16	3	0.00438999	1	3	5	piRNA biosynthetic process
**GO:0044711**	115.15	152	0.00452964	72	1072	1278	single-organism biosynthetic process
**GO:1901576**	372.24	435	0.00452964	200	3566	4398	organic substance biosynthetic process
**GO:0034058**	1.01	6	0.00458545	2	8	8	endosomal vesicle fusion
**GO:0045141**	0.36	4	0.00458545	1	9	9	meiotic telomere clustering
**GO:0043299**	39.59	62	0.00458545	30	434	571	leukocyte degranulation
**GO:0046470**	7.32	18	0.0046395	12	68	85	phosphatidylcholine metabolic process
**GO:0014870**	2.35	9	0.0046395	2	10	11	response to muscle inactivity
**GO:1902883**	8.73	20	0.0046395	4	39	46	negative regulation of response to oxidative stress
**GO:0031670**	2.22	9	0.00475608	4	22	24	cellular response to nutrient
**GO:0005109**	2.78	10	0.00475608	1	20	26	frizzled binding
**GO:1903975**	1.41	7	0.00475608	1	7	8	regulation of glial cell migration
**GO:0015630**	10.99	23	0.00475608	6	110	120	microtubule cytoskeleton
**GO:0019222**	683.48	763	0.00475608	334	5407	6639	regulation of metabolic process
**GO:0044237**	817.98	904	0.00475608	438	7162	8739	cellular metabolic process
**GO:0071947**	0.04	2	0.00475608	1	1	8	protein deubiquitination involved in ubiquitin-dependent protein catabolic process
**GO:0048806**	5.40	15	0.00480754	4	30	38	genitalia development
**GO:0045786**	43.01	66	0.00480754	24	447	571	negative regulation of cell cycle
**GO:0043207**	47.89	72	0.00480754	33	579	770	response to external biotic stimulus
**GO:0000122**	83.37	114	0.00480754	48	619	780	negative regulation of transcription from RNA polymerase II promoter
**GO:0016868**	0.67	5	0.00487561	3	8	10	intramolecular transferase activity, phosphotransferases
**GO:0010635**	4.93	14	0.00487561	2	9	9	regulation of mitochondrial fusion

GO Term	ANG	Can	FDR	Unique	Max	Total	Description
**GO:0002526**	4.14	13	0.00487561	4	53	63	acute inflammatory response
**GO:0015106**	1.40	7	0.00495243	1	11	11	bicarbonate transmembrane transporter activity
**GO:0072677**	0.39	4	0.00501314	1	8	13	eosinophil migration
**GO:0010469**	43.14	66	0.00501314	24	410	576	regulation of receptor activity
**GO:0031072**	11.94	25	0.00501314	7	94	118	heat shock protein binding
**GO:0090169**	0.65	5	0.00508222	1	15	19	regulation of spindle assembly
**GO:0051026**	0.38	4	0.00508222	1	5	6	chiasma assembly
**GO:0042119**	39.97	62	0.00515755	30	430	564	neutrophil activation
**GO:0031328**	217.52	265	0.00521783	110	1579	1942	positive regulation of cellular biosynthetic process
**GO:0035967**	5.54	15	0.00521783	3	10	16	cellular response to topologically incorrect protein
**GO:1903706**	36.85	58	0.00527792	23	357	486	regulation of hemopoiesis
**GO:1903053**	4.48	13	0.00534353	5	32	38	regulation of extracellular matrix organization
**GO:0097165**	0.18	3	0.00539527	1	5	5	nuclear stress granule
**GO:0043507**	8.00	19	0.00539527	7	58	78	positive regulation of JUN kinase activity
**GO:0045202**	65.09	93	0.00539527	40	196	239	synapse
**GO:0044428**	417.07	481	0.00539527	228	3668	4485	nuclear part
**GO:2001032**	0.70	5	0.00545669	2	13	15	regulation of double-strand break repair via nonhomologous end joining
**GO:0061631**	1.91	8	0.00552214	1	23	27	ubiquitin conjugating enzyme activity
**GO:0051136**	1.47	7	0.00558991	2	6	6	regulation of NK T cell differentiation
**GO:0046545**	0.71	5	0.00558991	1	3	5	development of primary female sexual characteristics
**GO:0070840**	1.90	8	0.00558991	2	21	23	dynein complex binding
**GO:0006766**	12.74	26	0.00567489	7	110	122	vitamin metabolic process
**GO:0043217**	1.48	7	0.00573876	1	12	12	myelin maintenance
**GO:0016441**	1.88	8	0.00577578	1	19	19	posttranscriptional gene silencing
**GO:0034397**	0.40	4	0.00577578	1	10	10	telomere localization
**GO:0033043**	116.21	152	0.00577578	62	976	1142	regulation of organelle organization
**GO:0090220**	0.40	4	0.00577578	1	10	10	chromosome localization to nuclear envelope involved in homologous chromosome segregation
**GO:0006297**	1.50	7	0.00577578	1	22	23	nucleotide-excision repair, DNA gap filling
**GO:1900372**	4.50	13	0.00581658	3	22	31	negative regulation of purine nucleotide biosynthetic process
**GO:0030809**	4.50	13	0.00581658	3	22	31	negative regulation of nucleotide biosynthetic process
**GO:0000989**	56.53	82	0.00581658	34	470	558	transcription factor activity, transcription factor binding
**GO:0000988**	56.53	82	0.00581658	34	470	558	transcription factor activity, protein binding
**GO:0034587**	1.05	6	0.00581658	3	15	19	piRNA metabolic process
**GO:0004859**	0.69	5	0.00588249	2	9	11	phospholipase inhibitor activity
**GO:0051050**	128.51	166	0.00588249	67	854	1019	positive regulation of transport
**GO:0050700**	1.28	7	0.00593468	3	13	14	CARD domain binding
**GO:0036230**	40.18	62	0.00593468	30	432	570	granulocyte activation
**GO:0061650**	1.94	8	0.00597363	1	24	28	ubiquitin-like protein conjugating enzyme activity
**GO:0032107**	19.21	35	0.00597363	11	176	216	regulation of response to nutrient levels
**GO:0032104**	19.21	35	0.00597363	11	176	216	regulation of response to extracellular stimulus
**GO:0014070**	93.66	126	0.00597363	50	656	777	response to organic cyclic compound
**GO:1903050**	22.86	40	0.00597363	11	217	278	regulation of proteolysis involved in cellular protein catabolic process
**GO:0045069**	8.79	20	0.00604125	4	77	92	regulation of viral genome replication
**GO:0061572**	8.95	20	0.00609374	6	41	51	actin filament bundle organization
**GO:0045010**	3.44	11	0.00609374	4	21	27	actin nucleation
**GO:0016279**	7.47	18	0.00609374	4	51	58	protein-lysine N-methyltransferase activity
**GO:0042221**	277.76	329	0.00614433	145	2058	2480	response to chemical
**GO:0061014**	1.89	8	0.00614433	1	20	23	positive regulation of mRNA catabolic process
**GO:0010243**	92.45	124	0.00625759	48	594	694	response to organonitrogen compound
**GO:0016579**	20.21	36	0.00625759	14	230	314	protein deubiquitination
**GO:0016278**	7.48	18	0.00625759	4	52	59	lysine N-methyltransferase activity
**GO:0045137**	0.74	5	0.00625759	1	4	6	development of primary sexual characteristics
**GO:0051099**	18.83	34	0.00634315	17	137	158	positive regulation of binding
**GO:0061640**	6.84	17	0.00641311	4	52	57	cytoskeleton-dependent cytokinesis
**GO:0044794**	0.75	5	0.00647322	1	13	13	positive regulation by host of viral process
**GO:0070301**	5.11	14	0.00647322	4	52	60	cellular response to hydrogen peroxide
**GO:1901374**	0.40	4	0.00652304	2	4	10	acetate ester transport
**GO:0046676**	6.96	17	0.00652304	2	33	38	negative regulation of insulin secretion
**GO:0015695**	1.51	7	0.00652304	3	18	24	organic cation transport
**GO:0023026**	1.10	6	0.00659605	2	16	47	MHC class II protein complex binding
**GO:0035666**	1.10	6	0.00666291	2	25	30	TRIF-dependent toll-like receptor signaling pathway
**GO:1902494**	70.17	98	0.00673157	35	725	888	catalytic complex
**GO:0051052**	29.29	48	0.00673157	21	327	389	regulation of DNA metabolic process
**GO:0000790**	19.61	35	0.00679964	15	172	211	nuclear chromatin
**GO:0044724**	6.39	16	0.00687201	9	76	105	single-organism carbohydrate catabolic process
**GO:0034045**	1.14	6	0.0070081	2	14	14	pre-autophagosomal structure membrane

GO Term	ANG	Can	FDR	Unique	Max	Total	Description
**GO:0060192**	1.50	7	0.00706435	2	12	15	negative regulation of lipase activity
**GO:0006903**	2.54	9	0.00706435	2	14	15	vesicle targeting
**GO:0017166**	1.95	8	0.00712845	1	9	10	vinculin binding
**GO:0060742**	1.14	6	0.00712845	3	7	8	epithelial cell differentiation involved in prostate gland development
**GO:0098586**	1.56	7	0.00727148	2	26	31	cellular response to virus
**GO:0022414**	125.72	161	0.00732391	68	1099	1341	reproductive process
**GO:0001101**	34.21	54	0.00732391	16	274	317	response to acid chemical
**GO:0016055**	49.79	73	0.00734978	22	288	349	Wnt signaling pathway
**GO:1904063**	11.74	24	0.00734978	7	51	62	negative regulation of cation transmembrane transport
**GO:0015101**	1.13	6	0.00734978	2	14	14	organic cation transmembrane transporter activity
**GO:0035580**	5.81	15	0.00734978	5	52	80	specific granule lumen
**GO:0051276**	23.48	40	0.00734978	17	340	420	chromosome organization
**GO:0045505**	1.14	6	0.00747764	1	8	8	dynein intermediate chain binding
**GO:0019985**	2.50	9	0.00751782	2	37	40	translesion synthesis
**GO:0033132**	3.54	11	0.00751782	1	4	5	negative regulation of glucokinase activity
**GO:1903300**	3.54	11	0.00751782	1	4	5	negative regulation of hexokinase activity
**GO:0019915**	2.05	8	0.00774758	2	23	30	lipid storage
**GO:0005126**	13.01	26	0.00774758	12	174	234	cytokine receptor binding
**GO:1903689**	1.56	7	0.00780924	1	6	6	regulation of wound healing, spreading of epidermal cells
**GO:0090407**	41.63	63	0.00786965	28	406	495	organophosphate biosynthetic process
**GO:0055102**	0.76	5	0.00786965	2	13	16	lipase inhibitor activity
**GO:0045637**	22.57	39	0.00786965	14	182	249	regulation of myeloid cell differentiation
**GO:0016052**	7.14	17	0.00822922	10	81	111	carbohydrate catabolic process
**GO:0045058**	4.65	13	0.0082726	4	24	25	T cell selection
**GO:0035973**	0.45	4	0.0082726	1	5	6	aggrephagy
**GO:2001021**	3.56	11	0.00833096	5	53	71	negative regulation of response to DNA damage stimulus
**GO:0070120**	0.45	4	0.00840568	1	5	5	ciliary neurotrophic factor-mediated signaling pathway
**GO:2000779**	4.13	12	0.0086051	5	47	52	regulation of double-strand break repair
**GO:0048562**	13.90	27	0.00868963	12	101	122	embryonic organ morphogenesis
**GO:0015747**	1.18	6	0.00868963	1	5	5	urate transport
**GO:0010994**	3.64	11	0.00868963	1	4	5	free ubiquitin chain polymerization
**GO:0032351**	0.79	5	0.00868963	1	8	8	negative regulation of hormone metabolic process
**GO:0009314**	40.94	62	0.00876218	26	367	424	response to radiation
**GO:0035519**	3.59	11	0.00879376	1	4	5	protein K29-linked ubiquitination
**GO:0070646**	20.64	36	0.00879376	14	243	327	protein modification by small protein removal
**GO:0042611**	1.52	7	0.00882936	3	16	101	MHC protein complex
**GO:0033131**	3.63	11	0.00882936	1	7	9	regulation of glucokinase activity
**GO:0009891**	219.93	265	0.00882936	110	1600	1969	positive regulation of biosynthetic process
**GO:0043401**	11.95	24	0.00894433	5	51	59	steroid hormone mediated signaling pathway
**GO:0008134**	50.01	73	0.00894433	34	428	511	transcription factor binding
**GO:0006471**	1.59	7	0.00897723	3	20	21	protein ADP-ribosylation
**GO:0042733**	9.10	20	0.00897723	7	51	58	embryonic digit morphogenesis
**GO:1903377**	3.62	11	0.00897723	1	4	5	negative regulation of oxidative stress-induced neuron intrinsic apoptotic signaling pathway
**GO:0043687**	54.08	78	0.00902577	30	376	463	post-translational protein modification
**GO:0070842**	3.61	11	0.00902577	1	4	8	aggresome assembly
**GO:0048029**	7.91	18	0.00907047	4	62	65	monosaccharide binding
**GO:0043648**	8.58	19	0.00919914	5	83	86	dicarboxylic acid metabolic process
**GO:1902510**	0.21	3	0.00941453	1	6	7	regulation of apoptotic DNA fragmentation
**GO:0090192**	1.63	7	0.00941453	1	11	13	regulation of glomerulus development
**GO:0044424**	1432.29	1528	0.00954113	710	11426	14183	intracellular part
**GO:0032924**	1.15	6	0.00968625	2	18	22	activin receptor signaling pathway
**GO:0033147**	1.67	7	0.00968625	3	11	14	negative regulation of intracellular estrogen receptor signaling pathway
**GO:0015701**	3.04	10	0.00968625	4	33	36	bicarbonate transport
**GO:0070585**	5.92	15	0.00968625	3	42	55	protein localization to mitochondrion
**GO:0055114**	80.70	109	0.00975156	44	750	892	oxidation-reduction process
**GO:0051494**	18.55	33	0.00975156	7	101	116	negative regulation of cytoskeleton organization
**GO:0023023**	1.21	6	0.00980001	2	17	50	MHC protein complex binding
**GO:0043388**	7.37	17	0.0098467	6	41	49	positive regulation of DNA binding
**GO:0000910**	7.24	17	0.0098467	4	58	64	cytokinesis
**GO:0097602**	3.64	11	0.00991074	1	9	11	cullin family protein binding
**GO:0004497**	9.19	20	0.00995754	8	80	95	monooxygenase activity
**GO:1990381**	4.21	12	0.00995754	2	15	21	ubiquitin-specific protease binding
**GO:0071671**	1.67	7	0.01008223	1	6	11	regulation of smooth muscle cell chemotaxis
**GO:1902018**	0.82	5	0.01008223	1	9	9	negative regulation of cilium assembly

GO Term	ANG	Can	FDR	Unique	Max	Total	Description
**GO:1903299**	3.69	11	0.01008223	1	8	10	regulation of hexokinase activity
**GO:0051224**	20.96	36	0.01012243	8	171	232	negative regulation of protein transport
**GO:1903321**	9.98	21	0.01012243	6	138	179	negative regulation of protein modification by small protein conjugation or removal
**GO:0014854**	2.66	9	0.01026956	2	13	14	response to inactivity
**GO:0015696**	9.94	21	0.01026956	7	53	65	ammonium transport
**GO:0010566**	1.64	7	0.01026956	2	13	13	regulation of ketone biosynthetic process
**GO:0005789**	93.14	125	0.01031114	57	794	957	endoplasmic reticulum membrane
**GO:0016706**	7.30	17	0.01036564	3	37	39	oxidoreductase activity, acting on paired donors, with incorporation or reduction of molecular oxygen, 2-oxoglutarate as one donor, and incorporation of one atom each of oxygen into both donors
**GO:0012506**	82.59	111	0.01041474	49	602	780	vesicle membrane
**GO:0030154**	198.34	241	0.01041474	106	1353	1651	cell differentiation
**GO:0012505**	8.68	19	0.01045298	8	47	54	endomembrane system
**GO:0061734**	3.67	11	0.01045298	1	4	5	parkin-mediated mitophagy in response to mitochondrial depolarization
**GO:0007015**	27.73	45	0.01048546	17	144	175	actin filament organization
**GO:1901880**	6.12	15	0.01048546	3	47	54	negative regulation of protein depolymerization
**GO:0018119**	0.80	5	0.01048546	1	5	6	peptidyl-cysteine S-nitrosylation
**GO:0017014**	0.80	5	0.01048546	1	5	6	protein nitrosylation
**GO:0019904**	85.23	114	0.01082923	39	566	693	protein domain specific binding
**GO:0072393**	1.24	6	0.01095426	1	9	10	microtubule anchoring at microtubule organizing center
**GO:0097413**	3.72	11	0.01101217	1	5	5	Lewy body
**GO:0044314**	3.73	11	0.01113954	1	4	5	protein K27-linked ubiquitination
**GO:0051354**	4.86	13	0.01126205	2	17	22	negative regulation of oxidoreductase activity
**GO:2000483**	0.51	4	0.01130406	1	6	7	negative regulation of interleukin-8 secretion
**GO:1902307**	3.23	10	0.01147227	2	14	17	positive regulation of sodium ion transmembrane transport
**GO:0046466**	1.73	7	0.01168848	1	20	23	membrane lipid catabolic process
**GO:0030742**	2.67	9	0.0117367	4	20	23	GTP-dependent protein binding
**GO:0050764**	9.54	20	0.01178107	7	61	88	regulation of phagocytosis
**GO:0008637**	5.60	14	0.01178107	3	47	52	apoptotic mitochondrial changes
**GO:0070841**	3.78	11	0.01182274	1	5	10	inclusion body assembly
**GO:0034452**	1.71	7	0.01196273	2	11	12	dynactin binding
**GO:0044828**	3.83	11	0.01199735	1	5	5	negative regulation by host of viral genome replication
**GO:0010950**	20.33	35	0.01199735	8	134	163	positive regulation of endopeptidase activity
**GO:0016866**	0.87	5	0.01203055	3	13	16	intramolecular transferase activity
**GO:0034968**	8.13	18	0.01203055	4	63	73	histone lysine methylation
**GO:0043506**	8.78	19	0.01205675	7	71	92	regulation of JUN kinase activity
**GO:1904264**	3.77	11	0.01205675	1	11	18	ubiquitin protein ligase activity involved in ERAD pathway
**GO:0043242**	6.23	15	0.01205675	3	50	63	negative regulation of protein complex disassembly
**GO:0002437**	2.19	8	0.01217953	1	18	28	inflammatory response to antigenic stimulus
**GO:0033643**	1.28	6	0.01217953	1	9	11	host cell part
**GO:0048518**	682.35	753	0.01221971	321	4744	5866	positive regulation of biological process
**GO:0098779**	3.78	11	0.01226184	1	7	8	mitophagy in response to mitochondrial depolarization
**GO:0044233**	0.84	5	0.01226184	2	9	11	ER-mitochondrion membrane contact site
**GO:0016192**	150.54	187	0.01235923	88	1240	1500	vesicle-mediated transport
**GO:0000776**	8.13	18	0.01235923	7	108	121	kinetochore
**GO:0032590**	2.24	8	0.01235923	1	13	14	dendrite membrane
**GO:1903376**	3.81	11	0.01260118	1	7	8	regulation of oxidative stress-induced neuron intrinsic apoptotic signaling pathway
**GO:0005346**	0.26	3	0.01263803	1	5	6	purine ribonucleotide transmembrane transporter activity
**GO:0000295**	0.26	3	0.01263803	1	5	6	adenine nucleotide transmembrane transporter activity
**GO:1903362**	26.50	43	0.01263803	13	238	302	regulation of cellular protein catabolic process
**GO:0006513**	7.62	17	0.0126847	5	49	65	protein monoubiquitination
**GO:0051588**	12.41	24	0.01273272	9	57	63	regulation of neurotransmitter transport
**GO:1904469**	0.90	5	0.01284421	1	9	11	positive regulation of tumor necrosis factor secretion
**GO:2001242**	10.83	22	0.01289394	7	124	148	regulation of intrinsic apoptotic signaling pathway
**GO:0000791**	1.29	6	0.01292973	3	23	30	euchromatin
**GO:0003676**	294.83	344	0.01292973	160	3102	3882	nucleic acid binding
**GO:0036120**	2.18	8	0.01306076	2	18	20	cellular response to platelet-derived growth factor stimulus
**GO:0051403**	6.31	15	0.01310577	4	62	77	stress-activated MAPK cascade
**GO:0004111**	0.25	3	0.0131573	1	6	6	creatine kinase activity
**GO:0044723**	54.22	79	0.0131573	35	468	559	single-organism carbohydrate metabolic process

GO Term	ANG	Can	FDR	Unique	Max	Total	Description
**GO:0071420**	2.28	8	0.0131573	4	8	8	cellular response to histamine
**GO:0043267**	6.87	16	0.0131573	5	24	28	negative regulation of potassium ion transport
**GO:0071704**	876.03	954	0.01319302	464	7537	9182	organic substance metabolic process
**GO:2000045**	13.83	26	0.01329932	10	123	154	regulation of G1/S transition of mitotic cell cycle
**GO:0046632**	5.67	14	0.01332468	3	30	33	alpha-beta T cell differentiation
**GO:0015812**	1.77	7	0.01332468	2	7	8	gamma-aminobutyric acid transport
**GO:0055015**	0.55	4	0.01332468	1	10	10	ventricular cardiac muscle cell development
**GO:0042165**	1.77	7	0.01338462	2	12	13	neurotransmitter binding
**GO:0090141**	3.86	11	0.01343074	1	14	14	positive regulation of mitochondrial fission
**GO:0005876**	2.25	8	0.0136248	2	34	41	spindle microtubule
**GO:0090494**	3.87	11	0.01371017	1	4	5	dopamine uptake
**GO:0090493**	3.87	11	0.01371017	1	4	5	catecholamine uptake
**GO:0051048**	25.98	42	0.01371017	12	160	224	negative regulation of secretion
**GO:0043046**	1.77	7	0.01371017	4	20	20	DNA methylation involved in gamete generation
**GO:0008276**	8.86	19	0.01377423	5	77	84	protein methyltransferase activity
**GO:0032092**	13.19	25	0.01392364	10	81	90	positive regulation of protein binding
**GO:0031016**	2.81	9	0.01392364	5	18	21	pancreas development
**GO:0012507**	4.43	12	0.01392364	5	40	104	ER to Golgi transport vesicle membrane
**GO:0010637**	3.87	11	0.01392364	1	6	6	negative regulation of mitochondrial fusion
**GO:1902916**	3.85	11	0.01392364	1	9	10	positive regulation of protein polyubiquitination
**GO:0032413**	12.51	24	0.01392364	7	51	59	negative regulation of ion transmembrane transporter activity
**GO:0031397**	9.68	20	0.01396659	5	131	169	negative regulation of protein ubiquitination
**GO:0003211**	0.55	4	0.01402638	2	9	10	cardiac ventricle formation
**GO:0044444**	1026.62	1109	0.01415051	510	7964	9722	cytoplasmic part
**GO:0033121**	0.91	5	0.01430756	1	6	6	regulation of purine nucleotide catabolic process
**GO:1903749**	10.92	22	0.01447022	9	100	126	positive regulation of establishment of protein localization to mitochondrion
**GO:1901985**	4.51	12	0.01450486	3	28	35	positive regulation of protein acetylation
**GO:1901203**	1.78	7	0.01452151	1	9	9	positive regulation of extracellular matrix assembly
**GO:0005737**	422.43	479	0.01452151	207	2879	3500	cytoplasm
**GO:0006767**	9.02	19	0.01481476	5	75	85	water-soluble vitamin metabolic process
**GO:0034063**	1.32	6	0.01481476	1	11	14	stress granule assembly
**GO:0001965**	2.25	8	0.01481476	1	17	18	G-protein alpha-subunit binding
**GO:0071877**	0.90	5	0.01486501	1	4	6	regulation of adrenergic receptor signaling pathway
**GO:0051301**	35.76	54	0.01495937	14	364	409	cell division
**GO:1902229**	1.77	7	0.01506546	3	31	39	regulation of intrinsic apoptotic signaling pathway in response to DNA damage
**GO:0002693**	1.75	7	0.01506546	1	11	14	positive regulation of cellular extravasation
**GO:0006607**	1.83	7	0.01508802	5	14	15	NLS-bearing protein import into nucleus
**GO:0061630**	14.78	27	0.01508802	7	115	143	ubiquitin protein ligase activity
**GO:0003406**	1.79	7	0.01508802	2	4	6	retinal pigment epithelium development
**GO:0044793**	3.98	11	0.01520076	1	8	8	negative regulation by host of viral process
**GO:0035520**	0.55	4	0.01523566	1	7	9	monoubiquitinated protein deubiquitination
**GO:0006304**	7.06	16	0.01523566	7	76	94	DNA modification
**GO:0002020**	13.93	26	0.01528694	13	102	145	protease binding
**GO:0030336**	49.65	71	0.01552776	24	203	249	negative regulation of cell migration
**GO:0032344**	0.92	5	0.01552776	1	6	6	regulation of aldosterone metabolic process
**GO:0032347**	0.92	5	0.01552776	1	6	6	regulation of aldosterone biosynthetic process
**GO:0030118**	3.42	10	0.01581447	1	14	14	clathrin coat
**GO:0016098**	0.28	3	0.01586756	2	5	7	monoterpenoid metabolic process
**GO:0019903**	15.45	28	0.01596254	12	108	121	protein phosphatase binding
**GO:0035864**	0.93	5	0.01596254	2	14	14	response to potassium ion
**GO:0005779**	0.91	5	0.0160005	3	15	16	integral component of peroxisomal membrane
**GO:0031231**	0.91	5	0.0160005	3	15	16	intrinsic component of peroxisomal membrane
**GO:2000146**	51.51	73	0.01601967	25	216	266	negative regulation of cell motility
**GO:0003207**	0.58	4	0.01601967	2	10	11	cardiac chamber formation
**GO:0030818**	3.37	10	0.01601967	2	17	26	negative regulation of cAMP biosynthetic process
**GO:0043274**	4.60	12	0.01609421	2	12	15	phospholipase binding
**GO:0030803**	3.38	10	0.01617513	2	19	28	negative regulation of cyclic nucleotide biosynthetic process
**GO:1902547**	4.06	11	0.01654089	2	14	16	regulation of cellular response to vascular endothelial growth factor stimulus
**GO:0000423**	4.01	11	0.01654089	1	12	13	macromitophagy
**GO:1903599**	3.97	11	0.01654089	1	7	7	positive regulation of mitophagy
**GO:0043067**	165.30	202	0.01663588	86	1200	1468	regulation of programmed cell death
**GO:0006622**	1.36	6	0.01675344	1	16	18	protein targeting to lysosome
**GO:0006955**	61.89	85	0.01686161	45	684	1131	immune response
**GO:0051059**	1.82	7	0.01686161	3	25	28	NF-kappaB binding
**GO:0044427**	51.94	73	0.01699062	35	601	780	chromosomal part
**GO:0097300**	1.36	6	0.01711415	3	20	20	programmed necrotic cell death

GO Term	ANG	Can	FDR	Unique	Max	Total	Description
**GO:0071287**	3.99	11	0.01715248	1	7	9	cellular response to manganese ion
**GO:0030594**	9.84	20	0.01724015	8	53	65	neurotransmitter receptor activity
**GO:0051937**	5.85	14	0.01726224	2	10	12	catecholamine transport
**GO:0008188**	7.09	16	0.01726224	4	30	38	neuropeptide receptor activity
**GO:0089720**	0.29	3	0.01726224	1	7	20	caspase binding
**GO:0051087**	10.47	21	0.01734631	9	78	92	chaperone binding
**GO:0016763**	6.57	15	0.01753194	5	41	45	transferase activity, transferring pentosyl groups
**GO:1903624**	0.28	3	0.01782227	1	7	9	regulation of DNA catabolic process
**GO:0008170**	9.13	19	0.01782227	5	77	86	N-methyltransferase activity
**GO:0030214**	2.38	8	0.01786236	2	15	16	hyaluronan catabolic process
**GO:0044710**	433.03	489	0.01798923	236	3598	4337	single-organism metabolic process
**GO:0007610**	116.68	147	0.01817045	53	452	536	behavior
**GO:0016747**	12.71	24	0.01822693	15	141	193	transferase activity, transferring acyl groups other than amino-acyl groups
**GO:0006464**	340.38	390	0.01824045	176	2525	3029	cellular protein modification process
**GO:0036211**	340.38	390	0.01824045	176	2525	3029	protein modification process
**GO:0043229**	890.29	965	0.01827775	443	7527	9229	intracellular organelle
**GO:1902235**	5.31	13	0.01830369	2	26	30	regulation of endoplasmic reticulum stress-induced intrinsic apoptotic signaling pathway
**GO:0042605**	1.30	6	0.01830369	2	15	58	peptide antigen binding
**GO:0006497**	28.16	44	0.01848568	16	108	141	protein lipidation
**GO:0051246**	287.55	334	0.01851995	137	2183	2644	regulation of protein metabolic process
**GO:0051926**	5.22	13	0.0185704	4	44	61	negative regulation of calcium ion transport
**GO:0003006**	58.75	81	0.0185704	31	499	589	developmental process involved in reproduction
**GO:0032962**	0.60	4	0.0185704	1	5	5	positive regulation of inositol trisphosphate biosynthetic process
**GO:0006479**	11.39	22	0.0185704	6	121	136	protein methylation
**GO:0061647**	1.37	6	0.0185704	1	13	19	histone H3-K9 modification
**GO:0002275**	42.68	62	0.0185704	30	445	585	myeloid cell activation involved in immune response
**GO:0008213**	11.39	22	0.0185704	6	121	136	protein alkylation
**GO:0061659**	15.08	27	0.01860419	7	120	148	ubiquitin-like protein ligase activity
**GO:1903531**	21.88	36	0.01869807	9	145	203	negative regulation of secretion by cell
**GO:0008089**	1.40	6	0.01872284	1	21	26	anterograde axon cargo transport
**GO:0071546**	0.59	4	0.01880255	2	7	7	pi-body
**GO:0048232**	28.98	45	0.019175	23	309	410	male gamete generation
**GO:0009063**	11.54	22	0.01925357	10	103	115	cellular amino acid catabolic process
**GO:0004601**	4.07	11	0.01925357	1	32	40	peroxidase activity
**GO:0090201**	4.12	11	0.01929576	1	14	17	negative regulation of release of cytochrome c from mitochondria
**GO:0034384**	1.41	6	0.01932116	3	8	15	high-density lipoprotein particle clearance
**GO:1902930**	6.67	15	0.01950098	5	73	85	regulation of alcohol biosynthetic process
**GO:0032886**	17.45	30	0.01967837	8	168	192	regulation of microtubule-based process
**GO:0051010**	1.92	7	0.01967837	1	12	12	microtubule plus-end binding
**GO:0030111**	52.16	73	0.0197202	22	277	340	regulation of Wnt signaling pathway
**GO:0019841**	0.61	4	0.01989258	1	13	14	retinol binding
**GO:0034618**	0.99	5	0.02003419	1	6	6	arginine binding
**GO:0016874**	16.54	29	0.02008847	10	150	168	ligase activity
**GO:0003264**	0.62	4	0.02033191	2	6	8	regulation of cardioblast proliferation
**GO:0003266**	0.62	4	0.02033191	2	6	8	regulation of secondary heart field cardioblast proliferation
**GO:0046328**	27.73	43	0.02057155	13	144	176	regulation of JNK cascade
**GO:0006623**	1.44	6	0.0206075	1	17	19	protein targeting to vacuole
**GO:0001818**	16.57	29	0.0206075	12	208	281	negative regulation of cytokine production
**GO:0022829**	0.62	4	0.02069037	1	17	22	wide pore channel activity
**GO:1902806**	14.47	26	0.02070561	10	133	169	regulation of cell cycle G1/S phase transition
**GO:1900407**	10.04	20	0.02070561	4	60	77	regulation of cellular response to oxidative stress
**GO:0090199**	5.35	13	0.02074539	2	39	45	regulation of release of cytochrome c from mitochondria
**GO:0006301**	3.00	9	0.02090961	2	43	47	postreplication repair
**GO:0003712**	51.53	72	0.02090961	30	421	503	transcription cofactor activity
**GO:0010506**	30.64	47	0.02118789	17	256	303	regulation of autophagy
**GO:0006776**	1.91	7	0.02120792	2	8	8	vitamin A metabolic process
**GO:0090140**	5.39	13	0.02128778	2	22	25	regulation of mitochondrial fission
**GO:0070050**	4.78	12	0.02159259	2	8	8	neuron cellular homeostasis
**GO:0015216**	0.32	3	0.02159259	1	6	7	purine nucleotide transmembrane transporter activity
**GO:0046033**	1.46	6	0.02159259	1	10	11	AMP metabolic process
**GO:0007212**	3.02	9	0.02196051	2	18	25	dopamine receptor signaling pathway
**GO:0048585**	189.32	227	0.02199633	86	1241	1520	negative regulation of response to stimulus
**GO:0071897**	8.03	17	0.02202702	4	93	98	DNA biosynthetic process
**GO:0036464**	13.68	25	0.02205588	9	143	189	cytoplasmic ribonucleoprotein granule
**GO:0034464**	1.02	5	0.02205588	1	8	8	BBSome
**GO:0001768**	1.98	7	0.02227373	1	5	6	establishment of T cell polarity

GO Term	ANG	Can	FDR	Unique	Max	Total	Description
**GO:0009893**	422.67	476	0.02227373	196	3000	3658	positive regulation of metabolic process
**GO:0003707**	13.03	24	0.02227373	5	50	59	steroid hormone receptor activity
**GO:0010833**	2.51	8	0.02236881	2	43	49	telomere maintenance via telomere lengthening
**GO:0002376**	176.25	212	0.02244308	111	1692	2391	immune system process
**GO:0016684**	4.18	11	0.02244308	1	35	43	oxidoreductase activity, acting on peroxide as acceptor
**GO:0043954**	5.44	13	0.02262042	2	31	33	cellular component maintenance
**GO:0010042**	4.18	11	0.02272828	1	12	16	response to manganese ion
**GO:0043414**	16.85	29	0.02282317	10	192	222	macromolecule methylation
**GO:0009060**	1.49	6	0.02282317	1	16	20	aerobic respiration
**GO:0072666**	1.48	6	0.02301858	1	18	20	establishment of protein localization to vacuole
**GO:0019538**	433.44	487	0.02304528	222	3335	4052	protein metabolic process
**GO:1902260**	2.53	8	0.02307915	2	7	7	negative regulation of delayed rectifier potassium channel activity
**GO:0015872**	4.22	11	0.02307915	1	7	8	dopamine transport
**GO:0016485**	24.50	39	0.02314886	12	174	204	protein processing
**GO:0097164**	19.92	33	0.02314886	17	149	181	ammonium ion metabolic process
**GO:1904385**	1.99	7	0.02332494	2	11	11	cellular response to angiotensin
**GO:1901992**	9.49	19	0.02336199	6	51	63	positive regulation of mitotic cell cycle phase transition
**GO:0004623**	2.53	8	0.02336199	5	31	33	phospholipase A2 activity
**GO:0044389**	30.23	46	0.02349756	21	232	301	ubiquitin-like protein ligase binding
**GO:0008152**	910.51	983	0.02360793	481	7887	9618	metabolic process
**GO:0009635**	0.10	2	0.02367303	1	6	6	response to herbicide
**GO:1900543**	7.47	16	0.02379442	5	41	53	negative regulation of purine nucleotide metabolic process
**GO:2000271**	1.04	5	0.02387669	2	6	7	positive regulation of fibroblast apoptotic process
**GO:0032872**	30.39	46	0.02398525	15	180	215	regulation of stress-activated MAPK cascade
**GO:0032647**	1.52	6	0.0240446	2	23	30	regulation of interferon-alpha production
**GO:1900825**	1.99	7	0.0240446	2	3	5	regulation of membrane depolarization during cardiac muscle cell action potential
**GO:0043618**	8.86	18	0.02405966	3	96	130	regulation of transcription from RNA polymerase II promoter in response to stress
**GO:0040029**	16.78	29	0.02417562	13	161	232	regulation of gene expression, epigenetic
**GO:0010569**	2.00	7	0.02464128	3	22	25	regulation of double-strand break repair via homologous recombination
**GO:0044271**	277.46	321	0.02464128	141	2702	3364	cellular nitrogen compound biosynthetic process
**GO:0006887**	65.66	88	0.02486193	44	616	786	exocytosis
**GO:2000378**	7.51	16	0.02486193	3	43	51	negative regulation of reactive oxygen species metabolic process
**GO:0001773**	3.65	10	0.02496375	3	20	25	myeloid dendritic cell activation
**GO:0015844**	6.84	15	0.02499057	3	18	20	monoamine transport
**GO:1902176**	4.27	11	0.02503392	1	16	19	negative regulation of oxidative stress-induced intrinsic apoptotic signaling pathway
**GO:1903047**	46.82	66	0.02513616	25	549	651	mitotic cell cycle process
**GO:0015866**	0.33	3	0.02513616	1	6	9	ADP transport
**GO:1903055**	2.54	8	0.02517418	2	17	19	positive regulation of extracellular matrix organization
**GO:2000269**	1.47	6	0.02520714	3	15	16	regulation of fibroblast apoptotic process
**GO:0032648**	3.12	9	0.0254832	3	38	51	regulation of interferon-beta production
**GO:0050866**	16.91	29	0.02562218	11	146	194	negative regulation of cell activation
**GO:0050905**	21.45	35	0.02562218	9	63	78	neuromuscular process
**GO:0045184**	119.67	149	0.02573649	57	990	1184	establishment of protein localization
**GO:0009620**	1.53	6	0.02575451	3	35	47	response to fungus
**GO:0030176**	7.58	16	0.02582999	6	88	180	integral component of endoplasmic reticulum membrane
**GO:0090370**	0.33	3	0.02582999	1	4	5	negative regulation of cholesterol efflux
**GO:0043412**	353.19	401	0.02585628	183	2665	3199	macromolecule modification
**GO:0051213**	9.60	19	0.0259796	5	74	85	dioxygenase activity
**GO:0045980**	7.56	16	0.02605498	5	42	55	negative regulation of nucleotide metabolic process
**GO:0001767**	2.06	7	0.02605498	1	6	7	establishment of lymphocyte polarity
**GO:0014002**	3.73	10	0.02628847	1	17	27	astrocyte development
**GO:0045059**	2.04	7	0.0263431	1	8	8	positive thymic T cell selection
**GO:0006541**	1.06	5	0.02639847	3	19	21	glutamine metabolic process
**GO:0005344**	3.16	11	0.02657754	1	9	14	oxygen transporter activity
**GO:0032410**	13.32	24	0.02684482	7	59	67	negative regulation of transporter activity
**GO:1903541**	4.35	11	0.02684482	1	16	16	regulation of exosomal secretion
**GO:0015671**	3.17	11	0.02684482	1	10	15	oxygen transport
**GO:0007283**	28.10	43	0.02691529	22	307	408	spermatogenesis
**GO:0031098**	6.93	15	0.02702131	4	67	82	stress-activated protein kinase signaling cascade
**GO:1902107**	16.36	28	0.02725976	9	111	151	positive regulation of leukocyte differentiation

GO Term	ANG	Can	FDR	Unique	Max	Total	Description
**GO:0071556**	1.48	6	0.0272844	2	19	85	integral component of lumenal side of endoplasmic reticulum membrane
**GO:0043522**	1.10	5	0.02732503	1	8	12	leucine zipper domain binding
**GO:0006672**	6.24	14	0.02740138	4	63	74	ceramide metabolic process
**GO:0043620**	8.99	18	0.02746844	3	100	136	regulation of DNA-templated transcription in response to stress
**GO:2001033**	0.35	3	0.02746844	1	5	6	negative regulation of double-strand break repair via nonhomologous end joining
**GO:1903817**	2.62	8	0.02756954	2	8	8	negative regulation of voltage-gated potassium channel activity
**GO:0090030**	1.10	5	0.02759264	1	9	9	regulation of steroid hormone biosynthetic process
**GO:0006656**	3.15	9	0.02782708	5	30	45	phosphatidylcholine biosynthetic process
**GO:0051247**	174.53	209	0.02784814	86	1288	1564	positive regulation of protein metabolic process
**GO:1903214**	12.53	23	0.02784814	7	82	106	regulation of protein targeting to mitochondrion
**GO:0070302**	30.75	46	0.02788213	15	181	216	regulation of stress-activated protein kinase signaling cascade
**GO:1902115**	14.11	25	0.02790987	10	130	157	regulation of organelle assembly
**GO:0034776**	2.61	8	0.02795824	4	10	11	response to histamine
**GO:0018022**	9.01	18	0.02815771	4	74	85	peptidyl-lysine methylation
**GO:0017034**	1.56	6	0.02835996	2	5	5	Rap guanyl-nucleotide exchange factor activity
**GO:0072488**	2.59	8	0.02839638	4	20	23	ammonium transmembrane transport
**GO:0008347**	2.09	7	0.02839638	1	14	20	glial cell migration
**GO:1904467**	1.12	5	0.02846358	1	15	18	regulation of tumor necrosis factor secretion
**GO:0070265**	1.56	6	0.02860496	3	21	21	necrotic cell death
**GO:0006296**	2.08	7	0.02867212	1	33	44	nucleotide-excision repair, DNA incision, 5'-to lesion
**GO:0015605**	1.11	5	0.02872181	2	18	21	organophosphate ester transmembrane transporter activity
**GO:0009755**	13.46	24	0.02880966	5	78	97	hormone-mediated signaling pathway
**GO:0051258**	6.99	15	0.02884034	3	46	58	protein polymerization
**GO:0035067**	0.69	4	0.02893847	1	13	16	negative regulation of histone acetylation
**GO:0015293**	14.85	26	0.02943504	9	114	123	symporter activity
**GO:0005544**	5.02	12	0.02943504	5	24	25	calcium-dependent phospholipid binding
**GO:0043201**	2.07	7	0.02963347	2	10	10	response to leucine
**GO:0030131**	1.59	6	0.0296907	1	22	24	clathrin adaptor complex
**GO:0050717**	0.36	3	0.0297093	1	4	5	positive regulation of interleukin-1 alpha secretion
**GO:0032730**	0.36	3	0.0297093	1	4	7	positive regulation of interleukin-1 alpha production
**GO:0036119**	2.61	8	0.03016499	2	19	21	response to platelet-derived growth factor
**GO:0045945**	1.60	6	0.03028334	2	10	11	positive regulation of transcription from RNA polymerase III promoter
**GO:2000427**	0.36	3	0.0304497	1	4	19	positive regulation of apoptotic cell clearance
**GO:1900117**	2.11	7	0.03049542	4	21	23	regulation of execution phase of apoptosis
**GO:0032480**	2.64	8	0.03100368	2	39	52	negative regulation of type I interferon production
**GO:0030659**	81.27	105	0.03121272	47	586	762	cytoplasmic vesicle membrane
**GO:0010677**	7.08	15	0.03121272	4	30	37	negative regulation of cellular carbohydrate metabolic process
**GO:0035025**	3.21	9	0.03150848	1	13	14	positive regulation of Rho protein signal transduction
**GO:0061608**	0.37	3	0.03153442	3	9	10	nuclear import signal receptor activity
**GO:0010523**	1.59	6	0.03153442	2	6	7	negative regulation of calcium ion transport into cytosol
**GO:0001756**	3.82	10	0.03165255	1	34	40	somitogenesis
**GO:0021591**	2.67	8	0.03168861	3	12	22	ventricular system development
**GO:0015867**	0.36	3	0.03190223	1	8	11	ATP transport
**GO:1902236**	4.46	11	0.03251271	1	16	18	negative regulation of endoplasmic reticulum stress-induced intrinsic apoptotic signaling pathway
**GO:0030575**	1.16	5	0.03264501	3	10	11	nuclear body organization
**GO:0006644**	44.03	62	0.03273098	31	352	416	phospholipid metabolic process
**GO:0016198**	4.43	11	0.03301113	2	6	6	axon choice point recognition
**GO:0034766**	13.53	24	0.03302501	7	69	84	negative regulation of ion transmembrane transport
**GO:0045502**	1.64	6	0.0330418	1	17	19	dynein binding
**GO:0036503**	4.45	11	0.03312169	1	17	24	ERAD pathway
**GO:0042981**	162.31	195	0.03318441	85	1188	1453	regulation of apoptotic process
**GO:0043226**	1071.35	1144	0.03337241	538	8790	10801	organelle
**GO:0033273**	9.18	18	0.03340459	10	78	83	response to vitamin
**GO:0005351**	1.63	6	0.03376985	1	10	11	sugar:proton symporter activity
**GO:0005402**	1.63	6	0.03376985	1	10	11	cation:sugar symporter activity
**GO:0070742**	1.65	6	0.03384394	1	11	21	C2H2 zinc finger domain binding
**GO:0070330**	1.16	5	0.0339174	2	19	24	aromatase activity
**GO:0044441**	42.67	60	0.03435593	24	318	354	ciliary part
**GO:0005942**	1.15	5	0.03437212	1	11	12	phosphatidylinositol 3-kinase complex
**GO:0042415**	4.55	11	0.03464883	1	6	9	norepinephrine metabolic process
**GO:0051184**	1.17	5	0.03464883	2	15	18	cofactor transporter activity

GO Term	ANG	Can	FDR	Unique	Max	Total	Description
**GO:0005355**	1.63	6	0.03477995	1	14	15	glucose transmembrane transporter activity
**GO:0050433**	7.13	15	0.03497438	4	34	41	regulation of catecholamine secretion
**GO:0033683**	2.18	7	0.03531689	1	35	47	nucleotide-excision repair, DNA incision
**GO:2001233**	34.26	50	0.0354671	17	315	378	regulation of apoptotic signaling pathway
**GO:0015669**	3.47	11	0.0354671	1	14	19	gas transport
**GO:0033209**	7.82	16	0.03553746	6	108	160	tumor necrosis factor-mediated signaling pathway
**GO:0015149**	1.65	6	0.0359503	1	15	16	hexose transmembrane transporter activity
**GO:0032612**	0.37	3	0.0359503	1	4	5	interleukin-1 production
**GO:0032611**	0.37	3	0.0359503	1	4	5	interleukin-1 beta production
**GO:1990776**	2.20	7	0.03596362	2	13	14	response to angiotensin
**GO:1902931**	1.18	5	0.03596362	1	16	18	negative regulation of alcohol biosynthetic process
**GO:0044432**	136.60	167	0.03596362	76	1108	1378	endoplasmic reticulum part
**GO:0033138**	12.94	23	0.03612741	10	76	91	positive regulation of peptidyl-serine phosphorylation
**GO:0072520**	0.74	4	0.03632153	1	9	12	seminiferous tubule development
**GO:0004622**	2.23	7	0.0367358	5	19	19	lysophospholipase activity
**GO:1901989**	10.06	19	0.03680121	6	62	77	positive regulation of cell cycle phase transition
**GO:0046929**	4.58	11	0.03680121	1	10	11	negative regulation of neurotransmitter secretion
**GO:0046683**	17.51	29	0.037043	11	117	129	response to organophosphorus
**GO:0071243**	0.40	3	0.03712357	1	11	17	cellular response to arsenic-containing substance
**GO:0051604**	26.30	40	0.03736539	13	194	229	protein maturation
**GO:0009987**	1469.91	1549	0.03737271	723	11284	13780	cellular process
**GO:0008306**	10.06	19	0.03740041	4	63	73	associative learning
**GO:2000573**	4.53	11	0.03740041	5	47	61	positive regulation of DNA biosynthetic process
**GO:0009084**	0.78	4	0.03755671	2	10	13	glutamine family amino acid biosynthetic process
**GO:0090279**	2.12	7	0.03755671	4	25	37	regulation of calcium ion import
**GO:1902017**	5.21	12	0.03757876	4	41	42	regulation of cilium assembly
**GO:0072006**	0.75	4	0.03762416	1	7	9	nephron development
**GO:1903409**	3.95	10	0.03762416	3	21	30	reactive oxygen species biosynthetic process
**GO:0006283**	3.37	9	0.03821249	3	66	81	transcription-coupled nucleotide-excision repair
**GO:0001505**	21.46	34	0.03821249	12	79	94	regulation of neurotransmitter levels
**GO:0034763**	13.73	24	0.03821512	7	77	92	negative regulation of transmembrane transport
**GO:0008285**	90.92	115	0.03824024	48	572	701	negative regulation of cell proliferation
**GO:0032183**	1.21	5	0.03839375	1	13	17	SUMO binding
**GO:1902175**	4.61	11	0.03839375	1	23	27	regulation of oxidative stress-induced intrinsic apoptotic signaling pathway
**GO:0032839**	4.62	11	0.0387362	2	22	23	dendrite cytoplasm
**GO:0055069**	4.59	11	0.03911883	1	13	14	zinc ion homeostasis
**GO:0035577**	5.24	12	0.03915508	8	52	58	azurophil granule membrane
**GO:0005975**	58.82	80	0.03915508	36	544	647	carbohydrate metabolic process
**GO:1900449**	10.06	19	0.03915508	6	35	37	regulation of glutamate receptor signaling pathway
**GO:0000723**	5.95	13	0.03931513	3	75	98	telomere maintenance
**GO:0045907**	2.77	8	0.03932299	3	22	25	positive regulation of vasoconstriction
**GO:0007568**	24.70	38	0.03932299	13	201	241	aging
**GO:0071363**	38.70	55	0.03945202	25	222	250	cellular response to growth factor stimulus
**GO:0031579**	3.99	10	0.03945202	3	17	21	membrane raft organization
**GO:0098902**	2.24	7	0.03945202	2	4	6	regulation of membrane depolarization during action potential
**GO:0046638**	4.65	11	0.03945282	5	33	38	positive regulation of alpha-beta T cell differentiation
**GO:0051293**	4.62	11	0.03945602	2	30	31	establishment of spindle localization
**GO:0044260**	635.23	693	0.03945602	325	5541	6783	cellular macromolecule metabolic process
**GO:0045333**	2.82	8	0.03960556	2	35	39	cellular respiration
**GO:0044451**	94.43	119	0.0396288	56	873	1084	nucleoplasm part
**GO:0005871**	1.22	5	0.03972054	1	18	19	kinesin complex
**GO:0010603**	0.40	3	0.03980437	1	6	6	regulation of cytoplasmic mRNA processing body assembly
**GO:0045920**	6.60	14	0.04000007	4	26	32	negative regulation of exocytosis
**GO:0072559**	0.40	3	0.04007753	1	4	6	NLRP3 inflammasome complex
**GO:0016746**	16.16	27	0.04013628	17	173	226	transferase activity, transferring acyl groups
**GO:0090278**	8.68	17	0.04025989	2	36	42	negative regulation of peptide hormone secretion
**GO:2000310**	6.62	14	0.04041365	5	16	18	regulation of N-methyl-D-aspartate selective glutamate receptor activity
**GO:0007218**	7.99	16	0.04049104	4	50	72	neuropeptide signaling pathway
**GO:0070888**	2.79	8	0.04058854	3	28	34	E-box binding
**GO:0015980**	8.70	17	0.04062535	9	88	104	energy derivation by oxidation of organic compounds
**GO:0031641**	9.50	18	0.04064314	6	32	35	regulation of myelination
**GO:0031227**	8.05	16	0.04090165	6	92	184	intrinsic component of endoplasmic reticulum membrane
**GO:0002792**	8.71	17	0.04090165	2	37	43	negative regulation of peptide secretion

GO Term	ANG	Can	FDR	Unique	Max	Total	Description
**GO:0042826**	11.63	21	0.04100749	7	85	103	histone deacetylase binding
**GO:0007528**	9.46	18	0.04105777	6	22	26	neuromuscular junction development
**GO:0032838**	4.68	11	0.04116932	2	24	25	cell projection cytoplasm
**GO:0043623**	35.63	51	0.04132566	14	299	383	cellular protein complex assembly
**GO:1903351**	4.67	11	0.04144578	1	11	15	cellular response to dopamine
**GO:1903350**	4.67	11	0.04144578	1	11	15	response to dopamine
**GO:0035065**	5.34	12	0.04167737	4	42	52	regulation of histone acetylation
**GO:0051503**	0.42	3	0.0416977	1	10	13	adenine nucleotide transport
**GO:0015868**	0.42	3	0.0416977	1	10	14	purine ribonucleotide transport
**GO:0002753**	2.29	7	0.04174332	3	30	36	cytoplasmic pattern recognition receptor signaling pathway
**GO:0044273**	2.81	8	0.04174332	2	39	47	sulfur compound catabolic process
**GO:0046885**	2.28	7	0.04205247	2	17	18	regulation of hormone biosynthetic process
**GO:0045646**	2.83	8	0.04220368	3	33	41	regulation of erythrocyte differentiation
**GO:0070403**	1.23	5	0.04222247	2	13	14	NAD+ binding
**GO:0032182**	10.95	20	0.04222247	5	85	104	ubiquitin-like protein binding
**GO:0044232**	1.24	5	0.04222247	2	13	15	organelle membrane contact site
**GO:0007033**	7.39	15	0.04227162	5	74	82	vacuole organization
**GO:0032438**	1.73	6	0.04230231	1	18	23	melanosome organization
**GO:0030433**	5.31	12	0.04250867	2	48	66	ER-associated ubiquitin-dependent protein catabolic process
**GO:0003062**	1.26	5	0.04256022	1	5	6	regulation of heart rate by chemical signal
**GO:0061462**	1.74	6	0.04318245	1	23	26	protein localization to lysosome
**GO:0042608**	2.28	7	0.04337331	1	6	10	T cell receptor binding
**GO:0021987**	14.83	25	0.04361102	9	52	60	cerebral cortex development
**GO:0043271**	20.99	33	0.04361102	12	105	131	negative regulation of ion transport
**GO:0044267**	356.16	400	0.04366048	179	2759	3356	cellular protein metabolic process
**GO:0048709**	5.39	12	0.04400058	4	24	26	oligodendrocyte differentiation
**GO:0042220**	4.75	11	0.04402821	3	27	33	response to cocaine
**GO:2001022**	6.72	14	0.04409399	7	68	73	positive regulation of response to DNA damage stimulus
**GO:0007143**	0.43	3	0.04431272	1	8	10	female meiotic division
**GO:0031528**	1.77	6	0.044595	4	20	25	microvillus membrane
**GO:0005776**	5.42	12	0.04482625	3	52	63	autophagosome
**GO:0061418**	6.78	14	0.044915	1	72	95	regulation of transcription from RNA polymerase II promoter in response to hypoxia
**GO:0003008**	207.63	242	0.04495726	97	1047	1233	system process
**GO:0002682**	132.08	160	0.04497605	77	1197	1731	regulation of immune system process
**GO:0005775**	15.56	26	0.04530668	16	145	200	vacuolar lumen
**GO:1900426**	0.43	3	0.04530668	2	8	9	positive regulation of defense response to bacterium
**GO:0030815**	4.10	10	0.04543792	2	21	30	negative regulation of cAMP metabolic process
**GO:0030117**	5.40	12	0.04556002	2	46	47	membrane coat
**GO:0051969**	2.88	8	0.04558371	2	12	15	regulation of transmission of nerve impulse
**GO:1901679**	0.43	3	0.04580246	1	11	12	nucleotide transmembrane transport
**GO:0001504**	4.73	11	0.04596102	1	11	11	neurotransmitter uptake
**GO:0070887**	190.49	223	0.04628908	100	1309	1543	cellular response to chemical stimulus
**GO:0030800**	4.11	10	0.04629919	2	23	32	negative regulation of cyclic nucleotide metabolic process
**GO:1902116**	2.91	8	0.04635625	3	28	32	negative regulation of organelle assembly
**GO:0010906**	13.31	23	0.04635625	8	81	97	regulation of glucose metabolic process
**GO:0032200**	6.12	13	0.04635625	3	79	111	telomere organization
**GO:0005938**	26.83	40	0.04641969	14	116	125	cell cortex
**GO:0090184**	3.50	9	0.04644444	3	33	41	positive regulation of kidney development
**GO:0010269**	0.15	2	0.04646787	1	4	6	response to selenium ion
**GO:0035082**	3.41	9	0.04689062	5	25	25	axoneme assembly
**GO:0002053**	2.91	8	0.04691013	5	26	28	positive regulation of mesenchymal cell proliferation
**GO:2000757**	0.83	4	0.04699005	1	16	20	negative regulation of peptidyl-lysine acetylation
**GO:0060455**	0.15	2	0.04792232	1	2	6	negative regulation of gastric acid secretion
**GO:0008083**	14.88	25	0.04792232	9	123	147	growth factor activity
**GO:0005720**	1.30	5	0.04802303	2	25	39	nuclear heterochromatin
**GO:0048738**	5.43	12	0.04822555	4	21	24	cardiac muscle tissue development
**GO:2000756**	5.47	12	0.0482897	4	45	57	regulation of peptidyl-lysine acetylation
**GO:0071248**	20.36	32	0.04831301	12	128	155	cellular response to metal ion
**GO:0010038**	41.03	57	0.04831959	22	277	326	response to metal ion
**GO:0045912**	7.53	15	0.04841457	4	38	46	negative regulation of carbohydrate metabolic process
**GO:0035282**	4.16	10	0.04848376	1	35	42	segmentation
**GO:0042439**	9.63	18	0.04853833	12	90	109	ethanolamine-containing compound metabolic process
**GO:0008270**	93.69	117	0.04860615	45	674	836	zinc ion binding
**GO:0032922**	6.86	14	0.04862608	4	47	56	circadian regulation of gene expression
**GO:0034622**	52.20	70	0.04913809	23	525	737	cellular macromolecular complex assembly

GO Term	ANG	Can	FDR	Unique	Max	Total	Description
**GO:0032270**	166.74	197	0.04924155	80	1205	1451	positive regulation of cellular protein metabolic process
**GO:0008094**	6.22	13	0.04924155	4	44	54	DNA-dependent ATPase activity
**GO:0050662**	22.03	34	0.04924155	13	168	191	coenzyme binding
**GO:0001016**	1.28	5	0.04924155	1	4	5	RNA polymerase III regulatory region DNA binding
**GO:0042572**	3.54	9	0.04924155	4	27	30	retinol metabolic process
**GO:0048753**	2.34	7	0.04924155	2	19	24	pigment granule organization
**GO:0033691**	0.85	4	0.04924155	2	8	9	sialic acid binding
**GO:0044390**	5.47	12	0.04925865	2	18	19	ubiquitin-like protein conjugating enzyme binding
**GO:0017147**	4.85	11	0.04971424	2	21	25	Wnt-protein binding
**GO:0031466**	0.45	3	0.04991748	2	5	6	Cul5-RING ubiquitin ligase complex
**GO:0010225**	0.85	4	0.04995983	2	13	13	response to UV-C
**GO:0005996**	14.14	27	0.04997778	12	160	192	monosaccharide metabolic process

GO: Gene ontology; ANG: Average number of genes involved; Can.: Number of candidate genes; FDR: *p*-value adjusted for false discovery rate (only FDR values <0.05 were taken as statistically significant); Unique: Unique genes detected; Max: Maximum possible number of genes detected; Total: total number of genes detected.

Table S7. GoWinda enrichment analysis results for TIX.

GO Term	ANG	Can	FDR	Unique	Max	Total	Description
**GO:0035145**	0.265	6	0.00127286	1	7	9	exon-exon junction complex
**GO:0016174**	0.532	7	0.00127286	2	5	6	NAD(P)H oxidase activity
**GO:0050810**	6.87	31	0.00127286	11	77	89	regulation of steroid biosynthetic process
**GO:0061333**	2.215	11	0.00127286	2	13	20	renal tubule morphogenesis
**GO:1901678**	0.568	10	0.00127286	2	8	11	iron coordination entity transport
**GO:0016427**	0.372	6	0.00127286	1	6	7	tRNA (cytosine) methyltransferase activity
**GO:0016469**	1.009	10	0.00127286	2	25	40	proton-transporting two-sector ATPase complex
**GO:0016471**	0.501	9	0.00127286	1	8	16	vacuolar proton-transporting V-type ATPase complex
**GO:0072512**	2.439	12	0.00127286	4	35	45	trivalent inorganic cation transport
**GO:0015232**	0.553	10	0.00127286	2	6	8	heme transporter activity
**GO:0060323**	0.839	9	0.00127286	1	5	9	head morphogenesis
**GO:0048671**	4.233	17	0.00127286	3	8	9	negative regulation of collateral sprouting
**GO:0046148**	4.716	17	0.00127286	3	38	46	pigment biosynthetic process
**GO:0042632**	5.976	20	0.00127286	5	57	67	cholesterol homeostasis
**GO:0030670**	4.612	17	0.00127286	7	55	105	phagocytic vesicle membrane
**GO:1904251**	1.506	20	0.00127286	3	12	12	regulation of bile acid metabolic process
**GO:0051184**	1.172	10	0.00127286	2	15	18	cofactor transporter activity
**GO:0015682**	2.439	12	0.00127286	4	35	45	ferric iron transport
**GO:0071498**	2.207	12	0.00127286	3	15	17	cellular response to fluid shear stress
**GO:0038007**	4.683	21	0.00127286	4	8	8	netrin-activated signaling pathway
**GO:0033179**	0.807	10	0.00127286	2	8	8	proton-transporting V-type ATPase, V0 domain
**GO:0033177**	0.862	10	0.00127286	2	11	14	proton-transporting two-sector ATPase complex, proton-transporting domain
**GO:0033176**	0.541	9	0.00127286	1	9	17	proton-transporting V-type ATPase complex
**GO:0051055**	3.392	28	0.00127286	8	43	47	negative regulation of lipid biosynthetic process
**GO:0035850**	2.479	12	0.00127286	3	8	9	epithelial cell differentiation involved in kidney development
**GO:0010565**	14.967	38	0.00127286	15	175	209	regulation of cellular ketone metabolic process
**GO:0045833**	5.589	29	0.00127286	9	71	84	negative regulation of lipid metabolic process
**GO:0044429**	59.181	95	0.00127286	58	794	975	mitochondrial part
**GO:0015991**	2.038	11	0.00127286	3	25	27	ATP hydrolysis coupled proton transport
**GO:0015988**	2.038	11	0.00127286	3	25	27	energy coupled proton transmembrane transport, against electrochemical gradient
**GO:0045939**	2.458	26	0.00127286	6	23	25	negative regulation of steroid metabolic process
**GO:0005042**	4.285	20	0.00127286	3	6	6	netrin receptor activity
**GO:0097090**	7.456	21	0.00127286	3	6	9	presynaptic membrane organization
**GO:0043249**	0.695	9	0.00127286	1	8	12	erythrocyte maturation
**GO:0070857**	1.342	20	0.00127286	3	10	10	regulation of bile acid biosynthetic process

GO Term	ANG	Can	FDR	Unique	Max	Total	Description
**GO:0010894**	2.16	26	0.00127286	6	21	23	negative regulation of steroid biosynthetic process
**GO:0046890**	13.506	40	0.00127286	16	153	170	regulation of lipid biosynthetic process
**GO:0019218**	10.874	38	0.00127286	13	99	114	regulation of steroid metabolic process
**GO:0033572**	2.419	12	0.00127286	4	34	43	transferrin transport
**GO:0015886**	0.553	10	0.00127286	2	6	8	heme transport
**GO:0055092**	5.976	20	0.00127286	5	57	67	sterol homeostasis
**GO:0015801**	2.544	12	0.00127286	1	4	5	aromatic amino acid transport
**GO:0042162**	2.032	11	0.00223708	4	26	32	telomeric DNA binding
**GO:0009226**	2.175	11	0.00223708	4	16	20	nucleotide-sugar biosynthetic process
**GO:0000777**	6.226	19	0.00223708	8	80	91	condensed chromosome kinetochore
**GO:0051181**	2.09	10	0.00223708	2	22	30	cofactor transport
**GO:0071705**	63.573	101	0.00223708	37	425	523	nitrogen compound transport
**GO:0055088**	11.079	28	0.00223708	10	92	104	lipid homeostasis
**GO:0006470**	34.837	61	0.00311462	25	163	187	protein dephosphorylation
**GO:0004721**	31.176	55	0.00311462	21	142	165	phosphoprotein phosphatase activity
**GO:0000096**	3.462	14	0.00311462	5	29	33	sulfur amino acid metabolic process
**GO:0043020**	0.628	6	0.00311462	1	7	10	NADPH oxidase complex
**GO:0050667**	1.056	7	0.00361117	2	11	12	homocysteine metabolic process
**GO:0030218**	4.539	16	0.00361117	4	43	61	erythrocyte differentiation
**GO:0031966**	33.66	58	0.00361117	34	496	620	mitochondrial membrane
**GO:0007185**	7.2	20	0.00361117	3	6	6	transmembrane receptor protein tyrosine phosphatase signaling pathway
**GO:0019198**	15.678	34	0.00361117	7	15	16	transmembrane receptor protein phosphatase activity
**GO:0051654**	0.843	7	0.00361117	2	8	12	establishment of mitochondrion localization
**GO:0005001**	15.678	34	0.00361117	7	15	16	transmembrane receptor protein tyrosine phosphatase activity
**GO:0007420**	40.535	70	0.00361117	23	151	193	brain development
**GO:0048670**	5.642	17	0.00438339	3	17	20	regulation of collateral sprouting
**GO:0090662**	2.521	11	0.00438339	3	32	34	ATP hydrolysis coupled transmembrane transport
**GO:0050673**	14.991	32	0.00488299	10	71	87	epithelial cell proliferation
**GO:0005640**	3.94	14	0.00488299	5	18	24	nuclear outer membrane
**GO:0090235**	0.952	7	0.00488299	1	5	5	regulation of metaphase plate congression
**GO:0004725**	25.856	47	0.00488299	15	80	97	protein tyrosine phosphatase activity
**GO:0061024**	124.741	169	0.00488299	81	746	899	membrane organization
**GO:0047497**	0.673	6	0.00622486	1	6	8	mitochondrion transport along microtubule
**GO:0034643**	0.673	6	0.00622486	1	6	8	establishment of mitochondrion localization, microtubule-mediated
**GO:0019673**	1.463	8	0.00622486	1	6	8	GDP-mannose metabolic process
**GO:0050775**	12.754	28	0.00671548	4	28	33	positive regulation of dendrite morphogenesis

GO Term	ANG	Can	FDR	Unique	Max	Total	Description
**GO:0030488**	1.015	7	0.00671548	2	18	19	tRNA methylation
**GO:0008175**	1.013	7	0.00671548	2	19	21	tRNA methyltransferase activity
**GO:0043515**	1.143	7	0.00718408	1	5	5	kinetochore binding
**GO:0090383**	2.12	10	0.00718408	2	26	34	phagosome acidification
**GO:0016311**	42.846	69	0.00718408	31	237	275	dephosphorylation
**GO:2000977**	0.086	3	0.00765	1	2	5	regulation of forebrain neuron differentiation
**GO:0045851**	2.541	11	0.00765	3	39	50	pH reduction
**GO:0051452**	2.541	11	0.00765	3	39	49	intracellular pH reduction
**GO:0010467**	1.571	8	0.00827838	2	5	14	gene expression
**GO:0035335**	26.885	48	0.00885716	16	83	98	peptidyl-tyrosine dephosphorylation
**GO:0042438**	3.324	12	0.01312553	1	11	13	melanin biosynthetic process
**GO:0071480**	1.225	7	0.01312553	2	22	28	cellular response to gamma radiation
**GO:0019216**	38.588	63	0.01312553	28	328	366	regulation of lipid metabolic process
**GO:0033564**	4.369	14	0.01312553	2	6	8	anterior/posterior axon guidance
**GO:0033130**	2.491	10	0.01363756	2	8	8	acetylcholine receptor binding
**GO:0019068**	0.285	4	0.01411414	1	10	11	virion assembly
**GO:0050658**	17.065	34	0.01475137	11	137	168	RNA transport
**GO:0050657**	17.065	34	0.01475137	11	137	168	nucleic acid transport
**GO:0051236**	17.065	34	0.01475137	11	137	168	establishment of RNA localization
**GO:0044550**	3.385	12	0.01475137	1	13	15	secondary metabolite biosynthetic process
**GO:0016791**	39.014	62	0.01475137	27	214	249	phosphatase activity
**GO:0090305**	16.193	33	0.01475137	22	224	283	nucleic acid phosphodiester bond hydrolysis
**GO:0033162**	3.45	12	0.01475137	1	11	12	melanosome membrane
**GO:0097105**	7.34	19	0.01475137	2	5	8	presynaptic membrane assembly
**GO:0031968**	15.068	31	0.01578021	17	149	180	organelle outer membrane
**GO:0008173**	1.683	8	0.0162773	3	36	42	RNA methyltransferase activity
**GO:1902931**	1.179	7	0.0162773	4	16	18	negative regulation of alcohol biosynthetic process
**GO:0042554**	0.888	6	0.0162773	1	10	13	superoxide anion generation
**GO:0060850**	3.034	11	0.0162773	4	14	18	regulation of transcription involved in cell fate commitment
**GO:0048536**	3.611	12	0.01757137	4	32	34	spleen development
**GO:0006879**	4.652	14	0.01757137	6	48	61	cellular iron ion homeostasis
**GO:0090129**	4.03	13	0.01776087	2	8	9	positive regulation of synapse maturation
**GO:1900006**	20.732	38	0.01776087	10	58	67	positive regulation of dendrite development
**GO:0090128**	4.096	13	0.0191617	2	10	11	regulation of synapse maturation
**GO:0000794**	1.693	8	0.0191617	3	21	25	condensed nuclear chromosome
**GO:0002067**	1.74	8	0.01994537	3	20	22	glandular epithelial cell differentiation
**GO:0051028**	8.826	21	0.01994537	8	117	144	mRNA transport
**GO:0070129**	0.905	6	0.020649	1	17	18	regulation of mitochondrial translation
**GO:0010510**	0.608	5	0.020649	2	10	13	regulation of acetyl-CoA biosynthetic process from pyruvate
**GO:0071709**	13.66	28	0.0230736	5	21	28	membrane assembly
**GO:0019867**	15.474	31	0.0238077	17	151	182	outer membrane
**GO:0044802**	97.039	131	0.0238077	58	587	723	single-organism membrane organization

GO Term	ANG	Can	FDR	Unique	Max	Total	Description
**GO:0050664**	1.372	7	0.02452175	2	10	14	oxidoreductase activity, acting on NAD(P)H, oxygen as acceptor
**GO:0034497**	0.366	4	0.02484557	2	5	5	protein localization to pre-autophagosomal structure
**GO:0055072**	7.767	19	0.02514802	7	66	82	iron ion homeostasis
**GO:0016175**	1.031	6	0.0259594	1	6	9	superoxide-generating NADPH oxidase activity
**GO:0051453**	3.76	12	0.02600521	4	52	64	regulation of intracellular pH
**GO:0032353**	0.619	5	0.02600521	3	7	7	negative regulation of hormone biosynthetic process
**GO:0007219**	13.249	27	0.0262065	10	94	118	Notch signaling pathway
**GO:0033540**	1.892	8	0.02917049	3	13	16	fatty acid beta-oxidation using acyl-CoA oxidase
**GO:0009295**	1.796	8	0.02917049	3	38	43	nucleoid
**GO:0042645**	1.796	8	0.02917049	3	38	43	mitochondrial nucleoid
**GO:0008138**	1.835	8	0.02935145	3	35	46	protein tyrosine/serine/threonine phosphatase activity
**GO:0070567**	1.043	6	0.0311	3	8	9	cytidylyltransferase activity
**GO:1902369**	1.016	6	0.03328087	1	7	10	negative regulation of RNA catabolic process
**GO:0006582**	3.942	12	0.03442866	1	12	14	melanin metabolic process
**GO:0006826**	3.974	12	0.03483992	4	49	66	iron ion transport
**GO:0000776**	8.129	19	0.03483992	8	108	121	kinetochore
**GO:0097119**	5.065	14	0.03483992	3	6	6	postsynaptic density protein 95 clustering
**GO:0010332**	2.929	10	0.03592412	4	46	53	response to gamma radiation
**GO:0016836**	4.534	13	0.03743394	3	44	47	hydro-lyase activity
**GO:0009067**	1.522	7	0.0374894	3	16	16	aspartate family amino acid biosynthetic process
**GO:0016004**	0.411	4	0.03875807	1	9	14	phospholipase activator activity
**GO:0015931**	19.24	35	0.03875807	12	168	204	nucleobase-containing compound transport
**GO:0001510**	1.928	8	0.03891934	3	39	45	RNA methylation
**GO:0034405**	4.073	12	0.04308577	3	30	33	response to fluid shear stress
**GO:0009225**	4.058	12	0.04406087	5	28	32	nucleotide-sugar metabolic process
**GO:0061005**	4.033	12	0.04553993	3	20	23	cell differentiation involved in kidney development
**GO:0030641**	4.083	12	0.04744207	4	55	68	regulation of cellular pH

GO: Gene ontology; ANG: Average number of genes involved; Can.: Number of candidate genes; FDR: *p*-value adjusted for false discovery rate (only FDR values <0.05 were taken as statistically significant); Unique: Unique genes detected; Max: Maximum possible number of genes detected; Total: total number of genes detected.

Table S8: SNP positions enriched in Chichén Itzá, Tixcacaltuyub and the SNPs reported by Lindo et al., 2016[17] for the HLA region.

Chichén Itzá (YCH)		Tixcacaltuyub (TIX)		Lindo et al., 2016	
SNP	Gene	SNP	Gene	SNP	Gene
rs1063349	*HLA-DQB1*	rs2647045	*HLA-DQA2, HLA-DQB1*	rs9272426	*HLA-DQA1*
rs1063355	*HLA-DQB1, HLA-DQB1, HLA-DRA, HLA-DQA1*	rs3130425	*HCG27, HLA-C*	rs3207966	*HLA-DQA1*
rs1071630	*HLA-DQA1*	rs6906021	*HLA-DQB1, HLA-DQA1*	rs3187964	*HLA-DQA1*
rs2394990	*DHFRP2, HLA-S*	rs7382297	*HLA-C, RPL3P2*	rs1047985	*HLA-DQA1*
rs3129304	*HLA-DOA*	rs7454108	*HLA-DQA2*	rs1047989	*HLA-DQA1*
rs34826728	*HLA-DQA1*	rs9275524	*HLA-DQB1*	rs1047993	*HLA-DQA1*
rs5000634	*HLA-DQB1, HLA-DQA2,*	rs9275595	*HLA-DQA2*	rs10093	*HLA-DQA1*
rs6928482	*HLA-DQB1*			rs1129765	*HLA-DQA1*
rs7745040	*HLA-DQA2, HLA-DRB1*			rs1142323	*HLA-DQA1*
rs9272484	*HLA-DQA1*				
rs9272547	*HLA-DQA1*				
rs9273373	*HLA-DQB1*				
rs9273479	*HLA-DQB1*				
rs9275141	*HLA-DQB1, HLA-DQA2*				
rs9469003	*MICA, HLA-X, HLA-B, HCP5*				

Table S9. HLA, mtDNA and Y-Chr genotypes for YCH.

Sample	mtDNA	Y-Chr	*HLA-A*	*HLA-B*	*HLA-C*	*HLA-DRB1*	*HLA-DRB3/4/5*	*HLA-DQA1*	*HLA-DQB1*	*HLA-DPA1*	*HLA-DPB1*
**YCH001**	B2l	Q1b1a1a	A*68:01	B*52:01	C*03:03	DRB1*04:11	DRB4*01:03	DQA1*03:01	DQB1*03:02	DPA1*01:03	DPB1*04:02
			A*02:06	B*40:02	C*03:04	DRB1*04:11	DRB4*01:03	DQA1*03:01	DQB1*03:02	DPA1*01:03	DPB1*04:02
**YCH002**	A2w1	Q1b1a2	A*24:02	B*39:02	C*07:02	DRB1*16:02	DRB5*02:02	DQA1*05:05	DQB1*03:01	DPA1*01:03	DPB1*04:02
			A*68:01	B*35:01	C*07:02	DRB1*04:07	DRB4*01:03	DQA1*03:01	DQB1*03:02	DPA1*01:03	DPB1*04:02
**YCH003**	A2q	Q1b1a1a	A*31:01	B*35:01	C*04:01	DRB1*04:10	DRB4*01:03	DQA1*03:03	DQB1*04:02	DPA1*01:03	DPB1*04:02
			A*31:01	B*35:20	C*15:09	DRB1*16:02	DRB5*02:02	DQA1*05:05	DQB1*03:01	DPA1*01:03	DPB1*04:02
**YCH004**	A2ap	Q1b1a1a	A*24:02	B*35:01	C*04:01	DRB1*14:06	DRB3*01:01	DQA1*05:03	DQB1*03:01	DPA1*01:03	DPB1*05:01
			A*31:01	B*35:01	C*04:01	DRB1*04:11	DRB4*01:03	DQA1*03:03	DQB1*04:02	DPA1*02:01	DPB1*04:02
**YCH006**	B2l	Q1b	A*24:02	B*39:06	C*07:02	DRB1*14:02	DRB3*01:01	DQA1*05:03	DQB1*03:01	DPA1*01:03	DPB1*04:01
			A*68:01	B*15:01	C*01:02	DRB1*04:04	DRB4*01:03	DQA1*03:01	DQB1*03:03	DPA1*01:03	DPB1*04:02
**YCH007**	A2u	Q1b1a1a	A*24:02	B*39:05	C*07:02	DRB1*04:07	DRB4*01:03	DQA1*03:01	DQB1*03:02	DPA1*01:03	DPB1*04:02
			A*68:05	B*35:23	C*04:01	DRB1*16:02	DRB5*02:02	DQA1*05:05	DQB1*03:01	DPA1*01:03	DPB1*04:02
**YCH008**	A2	Q1b1a1a1	A*02:01	B*35:01	C*07:02	DRB1*04:07	DRB4*01:03	DQA1*03:01	DQB1*03:02	DPA1*01:03	DPB1*03:01
			A*02:01	B*35:01	C*07:02	DRB1*08:02	NULL	DQA1*04:01	DQB1*04:02	DPA1*01:03	DPB1*04:02
**YCH010**	A2m	Q1b1a1a1g~	A*68:01	B*40:02	C*03:05	DRB1*04:04	DRB4*01:03	DQA1*03:01	DQB1*03:02	DPA1*01:03	DPB1*04:02
			A*24:02	B*40:08	C*03:04	DRB1*04:07	DRB4*01:03	DQA1*03:01	DQB1*03:02	DPA1*01:03	DPB1*04:02
**YCH011**	A2b1	Q1b1a1a1	A*24:02	B*35:01	C*04:01	DRB1*14:06	DRB3*01:01	DQA1*05:03	DQB1*03:01	DPA1*01:03	DPB1*04:02
			A*68:03	B*40:02	C*03:05	DRB1*04:07	DRB4*01:03	DQA1*03:01	DQB1*03:02	DPA1*01:03	DPB1*04:02
**YCH012**	A2g	Q1b1a1a1	A*24:02	B*40:02	C*03:05	DRB1*14:02	DRB3*01:01	DQA1*05:03	DQB1*03:01	DPA1*02:02	DPB1*05:01
			A*31:01	B*40:02	C*15:02	DRB1*04:07	DRB4*01:03	DQA1*03:01	DQB1*03:02	DPA1*01:03	DPB1*04:02
**YCH015**	A2	Q1b1a1a	A*31:01	B*40:02	C*03:04	DRB1*04:11	DRB4*01:03	DQA1*03:03	DQB1*04:02	DPA1*01:03	DPB1*04:02
			A*68:01	B*51:01	C*08:01	DRB1*14:02	DRB3*01:01	DQA1*05:03	DQB1*03:04	DPA1*01:03	DPB1*04:02
**YCH016**	A2w1	Q1b1a1a1	A*31:09	B*35:01	C*04:01	DRB1*04:11	DRB4*01:03	DQA1*03:03	DQB1*04:02	DPA1*01:03	DPB1*04:02
			A*02:06	B*35:01	C*04:01	DRB1*08:02	NULL	DQA1*04:01	DQB1*04:02	DPA1*01:03	DPB1*04:02
**YCH018**	A2ad	Q1b1a1a1	A*02:01	B*35:17	C*04:01	DRB1*04:11	DRB4*01:03	DQA1*03:03	DQB1*04:02	DPA1*01:03	DPB1*04:02
			A*24:02	B*39:02	C*03:04	DRB1*14:02	DRB3*01:01	DQA1*05:03	DQB1*03:01	DPA1*01:03	DPB1*04:02
**YCH020**	A2d	Q1b1a1a	A*24:02	B*15:01	C*01:02	DRB1*04:11	DRB4*01:03	DQA1*03:01	DQB1*03:02	DPA1*01:03	DPB1*04:01
			A*31:01	B*40:02	C*03:04	DRB1*04:04	DRB4*01:03	DQA1*03:01	DQB1*03:02	DPA1*01:03	DPB1*04:02
**YCH021**	A2r	Q1b1a1a1	A*68:01	B*40:08	C*03:04	DRB1*04:11	DRB4*01:03	DQA1*03:01	DQB1*03:02	DPA1*01:03	DPB1*04:02
			A*24:02	B*39:02	C*07:02	DRB1*16:02	DRB5*02:02	DQA1*05:05	DQB1*03:01	DPA1*01:03	DPB1*04:02
**YCH022**	A2ao	Q1b	A*68:01	B*35:01	C*04:01	DRB1*04:07	DRB4*01:03	DQA1*03:01	DQB1*03:03	DPA1*01:03	DPB1*04:02
			A*68:01	B*40:08	C*03:04	DRB1*14:02	DRB3*01:01	DQA1*05:03	DQB1*03:01	DPA1*01:03	DPB1*04:02
**YCH023**	A2a3	Q1b1a1a	A*02:06	B*35:01	C*07:02	DRB1*04:07	DRB4*01:03	DQA1*03:01	DQB1*03:02	DPA1*01:03	DPB1*03:01
			A*68:03	B*35:01	C*07:02	DRB1*04:07	DRB4*01:03	DQA1*05:03	DQB1*03:04	DPA1*01:03	DPB1*04:02
**YCH024**	C1c4	Q1b1a1a2b1~	A*02:01	B*35:17	C*04:01	DRB1*04:10	DRB4*01:03	DQA1*03:01	DQB1*03:02	DPA1*02:02	DPB1*05:01
			A*31:01	B*40:02	C*03:04	DRB1*04:11	DRB4*01:03	DQA1*03:03	DQB1*04:02	DPA1*02:01	DPB1*03:01
**YCH026**	D1	Q1b1a2	A*24:02	B*40:02	C*03:04	DRB1*04:11	DRB4*01:03	DQA1*03:03	DQB1*04:02	DPA1*01:03	DPB1*04:02
			A*68:01	B*35:20	C*04:01	DRB1*08:02	NULL	DQA1*04:01	DQB1*04:02	DPA1*01:03	DPB1*04:02
**YCH027**	B2b2	Q1b1a1a1	A*24:02	B*35:12	C*04:01	DRB1*14:02	DRB3*01:01	DQA1*05:03	DQB1*03:01	DPA1*02:01	DPB1*14:01

Sample	mtDNA	Y-Chr	*HLA-A*	*HLA-B*	*HLA-C*	*HLA-DRB1*	*HLA-DRB3/4/5*	*HLA-DQA1*	*HLA-DQB1*	*HLA-DPA1*	*HLA-DPB1*
			A*68:01	B*40:27	C*03:04	DRB1*04:04	DRB4*01:03	DQA1*03:01	DQB1*03:02	DPA1*01:03	DPB1*04:02
**YCH028**	A2	Q1b1a1a1e1c~	A*68:01	B*35:20	C*04:01	DRB1*04:07	DRB4*01:03	DQA1*03:01	DQB1*03:01	DPA1*01:03	DPB1*04:02
			A*24:02	B*40:02	C*03:04	DRB1*16:02	DRB5*02:02	DQA1*05:05	DQB1*03:02	DPA1*01:03	DPB1*04:02
**YCH030**	A2w1	Q1b1a2a~	A*24:02	B*15:01	C*01:02	DRB1*16:02	DRB5*02:02	DQA1*05:05	DQB1*03:01	DPA1*01:03	DPB1*14:01
			A*24:02	B*35:17	C*04:01	DRB1*04:03	DRB4*01:03	DQA1*03:01	DQB1*03:02	DPA1*02:01	DPB1*04:02
**YCH031**	A2r	Q1b1a1a	A*02:06	B*35:01	C*04:01	DRB1*04:03	DRB4*01:03	DQA1*03:01	DQB1*03:02	DPA1*01:03	DPB1*04:02
			A*31:01	B*35:01	C*07:02	DRB1*04:07	DRB4*01:03	DQA1*03:01	DQB1*03:02	DPA1*01:03	DPB1*04:02
**YCH032**	D1	Q1b1	A*68:01	B*40:02	C*03:04	DRB1*04:07	DRB4*01:03	DQA1*03:01	DQB1*03:02	DPA1*01:03	DPB1*04:02
			A*24:02	B*39:05	C*07:02	DRB1*14:02	DRB3*01:01	DQA1*05:01	DQB1*03:01	DPA1*01:03	DPB1*04:02
**YCH033**	A2	Q1b1a1a2b1~	A*68:01	B*40:02	C*03:04	DRB1*14:02	DRB3*01:01	DQA1*05:03	DQB1*03:01	DPA1*01:03	DPB1*04:02
			A*68:01	B*40:08	C*03:04	DRB1*16:02	DRB5*02:02	DQA1*05:05	DQB1*03:01	DPA1*01:03	DPB1*02:01
**YCH034**	A2w1	Q1b1a2~	A*68:01	B*40:02	C*03:05	DRB1*04:04	DRB4*01:03	DQA1*03:01	DQB1*03:02	DPA1*02:02	DPB1*04:02
			A*31:01	B*48:01	C*08:03	DRB1*04:04	DRB4*01:03	DQA1*03:01	DQB1*03:02	DPA1*01:03	DPB1*05:01
**YCH036**	A2	Q1b1a1a	A*24:02	B*35:01	C*04:01	DRB1*14:02	DRB3*01:01	DQA1*05:03	DQB1*03:01	DPA1*01:03	DPB1*04:02
			A*24:02	B*40:02	C*03:05	DRB1*14:02	DRB3*01:01	DQA1*05:03	DQB1*03:01	DPA1*01:03	DPB1*04:02
**YCH037**	A2a3	Q1b1a1a	A*31:01	B*35:01	C*04:01	DRB1*04:04	DRB4*01:03	DQA1*03:01	DQB1*03:02	DPA1*02:01	DPB1*04:02
			A*68:01	B*40:11	C*03:04	DRB1*04:07	DRB4*01:03	DQA1*03:01	DQB1*03:02	DPA1*01:03	DPB1*14:01
**YCH039**	A2r	Q1b1a1a	A*31:01	B*48:01	C*08:03	DRB1*16:02	DRB5*02:02	DQA1*05:05	DQB1*03:03	DPA1*01:03	DPB1*04:02
			A*24:02	B*35:17	C*03:03	DRB1*04:11	DRB4*01:03	DQA1*03:01	DQB1*03:02	DPA1*01:03	DPB1*04:02
**YCH040**	A2	Q1b1a1a	A*68:01	B*39:02	C*07:02	DRB1*04:11	DRB4*01:03	DQA1*03:03	DQB1*04:02	DPA1*01:03	DPB1*04:02
			A*68:01	B*39:05	C*03:04	DRB1*08:02	NULL	DQA1*04:01	DQB1*04:02	DPA1*01:03	DPB1*04:02
**YCH041**	A2w1	Q1b1a1a1m	A*31:01	B*40:02	C*03:05	DRB1*04:07	DRB4*01:03	DQA1*03:01	DQB1*03:02	DPA1*01:03	DPB1*04:02
			A*31:01	B*48:01	C*15:02	DRB1*04:03	DRB4*01:03	DQA1*03:01	DQB1*03:02	DPA1*01:03	DPB1*04:02
**YCH043**	A2w1	Q1b1a1a	A*24:02	B*35:01	C*07:02	DRB1*14:02	DRB3*01:01	DQA1*05:03	DQB1*03:01	DPA1*01:03	DPB1*04:02
			A*68:03	B*40:02	C*07:02	DRB1*14:02	DRB3*01:01	DQA1*05:03	DQB1*03:04	DPA1*01:03	DPB1*04:02
**YCH045**	C1c4	Q1b1a2	A*31:01	B*15:01	C*01:02	DRB1*04:11	DRB4*01:03	DQA1*03:01	DQB1*03:02	DPA1*01:03	DPB1*02:01
			A*24:02	B*35:01	C*04:01	DRB1*14:02	DRB3*01:01	DQA1*05:03	DQB1*03:01	DPA1*01:03	DPB1*04:02
**YCH046**	A2g	Q1b1a1a2	A*02:01	B*40:02	C*03:05	DRB1*04:04	DRB4*01:03	DQA1*03:01	DQB1*03:02	DPA1*01:03	DPB1*04:02
			A*31:01	B*15:01	C*01:02	DRB1*04:03	DRB4*01:03	DQA1*03:01	DQB1*03:02	DPA1*01:03	DPB1*04:02
**YCH048**	A2	Q1b1a2a~	A*24:02	B*52:01	C*03:04	DRB1*04:11	DRB4*01:03	DQA1*03:01	DQB1*03:02	DPA1*01:03	DPB1*04:02
			A*02:01	B*15:01	C*01:02	DRB1*04:11	DRB4*01:03	DQA1*03:01	DQB1*03:02	DPA1*01:03	DPB1*04:02
**YCH049**	D1	Q1b1a1a1	A*24:02	B*35:01	C*04:01	DRB1*04:07	DRB4*01:03	DQA1*03:01	DQB1*03:02	DPA1*01:03	DPB1*04:02
			A*24:02	B*40:02	C*03:04	DRB1*04:03	DRB4*01:03	DQA1*03:01	DQB1*03:02	DPA1*01:03	DPB1*04:02
**YCH050**	C1c	Q1b1a1a	A*68:01	B*39:05	C*07:02	DRB1*04:04	DRB4*01:03	DQA1*03:01	DQB1*03:02	DPA1*01:03	DPB1*04:02
			A*24:02	B*40:08	C*03:04	DRB1*04:11	DRB4*01:03	DQA1*03:03	DQB1*04:02	DPA1*01:03	DPB1*04:02
**YCH051**	N/D	Q1b1	A*24:02	B*35:01	C*07:02	DRB1*04:07	DRB4*01:03	DQA1*03:01	DQB1*03:02	DPA1*01:03	DPB1*04:02
			A*24:02	B*40:02	C*03:05	DRB1*14:02	DRB3*01:01	DQA1*05:03	DQB1*03:01	DPA1*02:01	DPB1*05:01
**YCH052**	A2af1b	Q1b1	A*68:03	B*35:01	C*07:02	DRB1*04:07	DRB4*01:03	DQA1*03:01	DQB1*03:02	DPA1*01:03	DPB1*04:02
			A*02:06	B*39:05	C*07:02	DRB1*04:07	DRB4*01:03	DQA1*03:01	DQB1*03:02	DPA1*01:03	DPB1*04:02
**YCH053**	A2w1	Q1b1a1a1e1c~	A*31:01	B*35:20	C*04:01	DRB1*04:10	DRB4*01:03	DQA1*03:03	DQB1*04:02	DPA1*01:03	DPB1*04:02
			A*24:02	B*40:08	C*03:04	DRB1*14:06	DRB3*01:01	DQA1*05:03	DQB1*03:01	DPA1*01:03	DPB1*04:02
**YCH055**	A2w1	Q1b1a~	A*02:06	B*39:02	C*03:04	DRB1*04:11	DRB4*01:03	DQA1*03:03	DQB1*04:02	DPA1*01:03	DPB1*04:02
			A*31:01	B*40:02	C*03:05	DRB1*04:17	DRB4*01:03	DQA1*03:01	DQB1*03:02	DPA1*02:02	DPB1*05:01

**Sample**	**mtDNA**	**Y-Chr**	***HLA-A***	***HLA-B***	***HLA-C***	***HLA-DRB1***	***HLA-DRB3/4/5***	***HLA-DQA1***	***HLA-DQB1***	***HLA-DPA1***	***HLA-DPB1***
**YCH056**	A2w1	Q1b1a2~	A*24:02	B*35:01	C*03:04	DRB1*14:06	DRB3*01:01	DQA1*05:03	DQB1*03:01	DPA1*02:01	DPB1*14:01
			A*68:01	B*40:08	C*03:04	DRB1*04:04	DRB4*01:03	DQA1*03:01	DQB1*03:02	DPA1*01:03	DPB1*04:02
**YCH057**	B2	Q1b1a2	A*24:02	B*35:17	C*03:04	DRB1*14:02	DRB3*01:01	DQA1*05:03	DQB1*03:01	DPA1*01:03	DPB1*04:02
			A*31:01	B*40:02	C*04:01	DRB1*04:03	DRB4*01:03	DQA1*03:01	DQB1*03:02	DPA1*01:03	DPB1*04:02
**YCH058**	C1b	Q1b1a1a	A*68:01	B*40:08	C*03:04	DRB1*04:03	DRB4*01:03	DQA1*03:01	DQB1*03:02	DPA1*01:03	DPB1*04:02
			A*68:03	B*40:02	C*03:04	DRB1*04:07	DRB4*01:03	DQA1*03:01	DQB1*03:02	DPA1*01:03	DPB1*04:02
**YCH059**	A2af1b	Q1b1a1a1f	A*68:01	B*35:12	C*04:01	DRB1*08:02	NULL	DQA1*04:01	DQB1*04:02	DPA1*01:03	DPB1*04:02
			A*24:02	B*39:02	C*07:02	DRB1*04:11	DRB4*01:03	DQA1*05:03	DQB1*03:01	DPA1*02:01	DPB1*05:01
**YCH060**	A2r	Q1b1a2a	A*68:01	B*40:02	C*03:05	DRB1*04:07	DRB4*01:03	DQA1*03:01	DQB1*03:02	DPA1*01:03	DPB1*04:02
			A*24:02	B*35:01	C*04:01	DRB1*04:10	DRB4*01:03	DQA1*03:03	DQB1*04:02	DPA1*01:03	DPB1*04:02
**YCH063**	C1c	Q1b1a2	A*24:02	B*35:01	C*04:01	DRB1*04:07	DRB4*01:03	DQA1*03:01	DQB1*03:02	DPA1*01:03	DPB1*04:02
			A*02:01	B*35:01	C*04:01	DRB1*04:07	DRB4*01:01	DQA1*03:01	DQB1*03:02	DPA1*01:03	DPB1*04:02

mtDNA: Mitochondrial DNA haplogroup; Y-Chr: Y chromosome haplotype; N/D: Not determined.

Table S10. HLA, mtDNA and Y-Chr genotypes for TIX.

Sample	mtDNA	Y-Chr	*HLA-A*	*HLA-B*	*HLA-C*	*HLA-DRB1*	*HLA-DRB3/4/5*	*HLA-DQA1*	*HLA-DQB1*	*HLA-DPA1*	*HLA-DPB1*
**TIX011**	A2u	Q1b1a1a1	A*02:01	B*35:12	C*04:01	DRB1*08:02	Null	DQA1*04:01	DQB1*04:02	DPA1*01:03	DPB1*04:02
			A*31:01	B*40:08	C*03:04	DRB1*04:07	DRB4*01:03	DQA1*03:01	DQB1*03:02	DPA1*01:03	DPB1*04:02
**TIX012**	A2ap	-	A*68:03	B*39:02	C*07:02	DRB1*04:07	DRB4*01:03	DQA1*03:01	DQB1*03:02	DPA1*01:03	DPB1*02:01
			A*68:03	B*39:05	C*07:02	DRB1*16:02	DRB5*02:02	DQA1*05:05	DQB1*03:01	DPA1*01:03	DPB1*04:02
**TIX013**	A2ad	Q1b1a1a	A*24:02	B*35:20	C*04:01	DRB1*04:07	DRB4*01:03	DQA1*03:01	DQB1*03:02	DPA1*01:03	DPB1*04:02
			A*24:02	B*40:08	C*03:04	DRB1*04:07	DRB4*01:03	DQA1*03:01	DQB1*03:02	DPA1*01:03	DPB1*04:02
**TIX014**	A2w1	-	A*24:02	B*40:08	C*03:04	DRB1*04:07	DRB4*01:03	DQA1*03:01	DQB1*03:02	DPA1*01:03	DPB1*04:02
			A*68:01	B*15:01	C*01:02	DRB1*04:04	DRB4*01:03	DQA1*03:01	DQB1*03:02	DPA1*01:03	DPB1*04:02
**TIX015**	C1b14	Q1b1a1a1	A*31:01	B*40:08	C*03:04	DRB1*04:07	DRB4*01:03	DQA1*03:01	DQB1*03:02	DPA1*01:03	DPB1*04:02
			A*68:01	B*39:05	C*07:02	DRB1*04:07	DRB4*01:03	DQA1*03:01	DQB1*03:02	DPA1*01:03	DPB1*04:02
**TIX016**	A2m	I2a1b1a2b1a2	A*68:01	B*35:12	C*07:02	DRB1*04:07	DRB4*01:03	DQA1*03:01	DQB1*03:02	DPA1*01:03	DPB1*04:02
			A*68:03	B*40:08	C*03:04	DRB1*04:25	DRB4*01:03	DQA1*03:01	DQB1*03:02	DPA1*01:03	DPB1*04:02
**TIX017**	A2af1b	G2a2b2a1a1c1a1a2a1a1a	A*23:01	B*15:03	C*02:10	DRB1*01:01	Null	DQA1*01:01	DQB1*02:02	DPA1*03:01	DPB1*105:01
			A*31:01	B*15:01	C*04:01	DRB1*09:01	DRB4*01:01	DQA1*03:03	DQB1*05:01	DPA1*01:03	DPB1*04:01
**TIX018**	A2u	Q1b1a1a1	A*32:01	B*51:01	C*01:02	DRB1*16:01	DRB5*02:02	DQA1*01:02	DQB1*05:02	DPA1*02:01	DPB1*14:01
			A*68:01	B*39:05	C*07:02	DRB1*04:07	DRB4*01:03	DQA1*03:01	DQB1*03:02	DPA1*01:03	DPB1*04:02
**TIX019**	A2	R1b1a1b1a1a2c1a	A*02:06	B*35:01	C*07:02	DRB1*04:07	DRB4*01:03	DQA1*03:01	DQB1*03:02	DPA1*01:03	DPB1*04:02
			A*02:06	B*35:17	C*04:01	DRB1*04:07	DRB4*01:03	DQA1*03:01	DQB1*03:02	DPA1*01:03	DPB1*04:02
**TIX020**	A2	-	A*68:01	B*35:12	C*04:01	DRB1*04:07	DRB4*01:03	DQA1*03:01	DQB1*03:02	DPA1*01:03	DPB1*04:02
			A*24:02	B*35:20	C*03:04	DRB1*14:02	DRB3*01:01	DQA1*05:03	DQB1*03:01	DPA1*01:03	DPB1*04:02
**TIX021**	A2w1	-	A*24:02	B*14:01	C*08:02	DRB1*13:03	DRB3*02:02	DQA1*02:01	DQB1*02:02	DPA1*01:03	DPB1*01:01
			A*32:01	B*53:01	C*04:01	DRB1*01:01	Null	DQA1*01:01	DQB1*05:01	DPA1*02:01	DPB1*04:01
**TIX022**	A2m	-	A*31:01	B*40:08	C*03:04	DRB1*04:04	DRB4*01:03	DQA1*03:01	DQB1*03:02	DPA1*01:03	DPB1*04:02
			A*68:01	B*45:01	C*06:02	DRB1*04:05	DRB4*01:03	DQA1*03:03	DQB1*03:02	DPA1*02:01	DPB1*11:01
**TIX023**	B2b	T1a1a1b2	A*03:01	B*35:01	C*04:01	DRB1*11:01	DRB3*02:02	DQA1*05:05	DQB1*03:19	DPA1*01:03	DPB1*05:01
			A*31:01	B*35:01	C*07:02	DRB1*04:07	DRB4*01:03	DQA1*03:01	DQB1*03:02	DPA1*02:01	DPB1*105:01
**TIX024**	A2	-	A*68:03	B*39:05	C*07:02	DRB1*04:07	DRB4*01:03	DQA1*03:01	DQB1*03:02	DPA1*01:03	DPB1*04:02
			A*68:03	B*39:05	C*07:02	DRB1*04:07	DRB4*01:03	DQA1*03:01	DQB1*03:02	DPA1*01:03	DPB1*04:02
**TIX025**	B2	-	A*68:03	B*40:02	C*04:01	DRB1*04:04	DRB4*01:03	DQA1*03:01	DQB1*03:02	DPA1*01:03	DPB1*04:02
			A*02:01	B*35:01	C*07:02	DRB1*14:02	DRB3*01:01	DQA1*05:03	DQB1*03:04	DPA1*01:03	DPB1*04:02
**TIX026**	A2ap	-	A*31:01	B*35:01	C*07:02	DRB1*04:03	DRB4*01:03	DQA1*03:01	DQB1*03:02	DPA1*01:03	DPB1*04:02
			A*24:02	B*51:01	C*15:09	DRB1*14:02	DRB3*01:01	DQA1*05:03	DQB1*03:01	DPA1*01:03	DPB1*04:02
**TIX027**	A2	-	A*24:02	B*39:05	C*07:02	DRB1*04:07	DRB4*01:03	DQA1*03:01	DQB1*03:02	DPA1*01:03	DPB1*04:02
			A*29:02	B*44:03	C*16:01	DRB1*11:03	DRB3*02:02	DQA1*05:05	DQB1*03:01	DPA1*01:03	DPB1*04:02
**TIX028**	A2ap	-	A*68:03	B*39:02	C*07:02	DRB1*04:03	DRB4*01:03	DQA1*03:01	DQB1*03:02	DPA1*01:03	DPB1*02:01
			A*31:01	B*35:01	C*07:02	DRB1*16:02	DRB5*02:02	DQA1*05:05	DQB1*03:01	DPA1*01:03	DPB1*04:02
**TIX029**	A2w1	-	A*68:03	B*39:05	C*07:02	DRB1*04:07	DRB4*01:03	DQA1*03:01	DQB1*03:02	DPA1*01:03	DPB1*04:02
			A*24:02	B*51:01	C*15:09	DRB1*14:02	DRB3*01:01	DQA1*05:03	DQB1*03:01	DPA1*01:03	DPB1*04:02
**TIX030**	A2ad	-	A*24:02	B*35:01	C*03:04	DRB1*14:02	DRB3*01:01	DQA1*05:03	DQB1*03:01	DPA1*01:03	DPB1*04:02
			A*68:01	B*35:17	C*04:01	DRB1*14:02	DRB3*01:01	DQA1*05:03	DQB1*03:04	DPA1*01:03	DPB1*04:02
**TIX031**	B2b	-	A*11:01	B*40:01	C*03:04	DRB1*04:04	DRB4*01:03	DQA1*03:01	DQB1*03:02	DPA1*01:03	DPB1*06:01
			A*68:03	B*39:05	C*07:02	DRB1*04:07	DRB4*01:03	DQA1*03:01	DQB1*03:02	DPA1*01:03	DPB1*04:02
**TIX032**	A2j	-	A*24:02	B*35:20	C*03:04	DRB1*04:07	DRB4*01:03	DQA1*03:01	DQB1*03:02	DPA1*01:03	DPB1*04:02

Sample	mtDNA	Y-Chr	*HLA-A*	*HLA-B*	*HLA-C*	*HLA-DRB1*	*HLA-DRB3/4/5*	*HLA-DQA1*	*HLA-DQB1*	*HLA-DPA1*	*HLA-DPB1*
			A*31:01	B*40:02	C*04:01	DRB1*13:02	DRB3*03:01	DQA1*01:02	DQB1*06:04	DPA1*01:03	DPB1*02:01
**TIX033**	C1c4	-	A*31:01	B*40:02	C*03:04	DRB1*08:02	Null	DQA1*04:01	DQB1*04:02	DPA1*01:03	DPB1*04:02
			A*68:03	B*35:12	C*07:02	DRB1*04:07	DRB4*01:03	DQA1*03:01	DQB1*03:02	DPA1*01:03	DPB1*04:02
**TIX034**	C1c4	Q1b1a1a	A*68:03	B*35:12	C*07:02	DRB1*04:07	DRB4*01:03	DQA1*03:01	DQB1*03:02	DPA1*01:03	DPB1*04:02
			A*68:03	B*39:05	C*07:02	DRB1*04:07	DRB4*01:03	DQA1*03:01	DQB1*03:02	DPA1*01:03	DPB1*04:02
**TIX035**	A2j	Q1b1a1a1	A*68:03	B*39:05	C*07:02	DRB1*04:07	DRB4*01:03	DQA1*03:01	DQB1*03:02	DPA1*02:02	DPB1*05:01
			A*31:01	B*40:02	C*03:04	DRB1*13:02	DRB3*03:01	DQA1*01:02	DQB1*06:04	DPA1*01:03	DPB1*02:01
**TIX036**	A2	-	A*02:01	B*35:12	C*04:01	DRB1*04:07	DRB4*01:03	DQA1*03:01	DQB1*03:02	DPA1*01:03	DPB1*04:02
			A*68:03	B*40:08	C*03:04	DRB1*04:11	DRB4*01:03	DQA1*03:01	DQB1*03:02	DPA1*01:03	DPB1*04:02
**TIX037**	A2u	-	A*68:01	B*48:01	C*08:03	DRB1*04:11	DRB4*01:03	DQA1*03:01	DQB1*03:02	DPA1*01:03	DPB1*04:02
			A*80:01	B*57:02	C*18:02	DRB1*10:01	Null	DQA1*01:04	DQB1*05:01	DPA1*02:02	DPB1*01:01
**TIX038**	A2ap	-	A*24:02	B*35:20	C*04:01	DRB1*04:07	DRB4*01:03	DQA1*03:01	DQB1*03:02	DPA1*01:03	DPB1*04:02
			A*24:02	B*51:01	C*15:09	DRB1*14:02	DRB3*01:01	DQA1*05:03	DQB1*03:01	DPA1*01:03	DPB1*04:02
**TIX039**	A2	-	A*31:01	B*39:05	C*07:02	DRB1*04:04	DRB4*01:03	DQA1*03:01	DQB1*03:02	DPA1*01:03	DPB1*04:02
			A*24:02	B*39:06	C*07:02	DRB1*14:06	DRB3*01:01	DQA1*05:03	DQB1*03:01	DPA1*01:03	DPB1*04:02
**TIX040**	A2m	-	A*31:01	B*40:08	C*03:04	DRB1*04:04	DRB4*01:03	DQA1*03:01	DQB1*03:02	DPA1*01:03	DPB1*04:02
			A*68:03	B*35:12	C*07:02	DRB1*04:07	DRB4*01:03	DQA1*03:01	DQB1*03:02	DPA1*01:03	DPB1*04:02
**TIX041**	A2w1	G2a2b2a	A*68:01	B*15:01	C*01:02	DRB1*04:07	DRB4*01:03	DQA1*03:01	DQB1*03:02	DPA1*01:03	DPB1*04:02
			A*68:03	B*39:05	C*07:02	DRB1*04:04	DRB4*01:03	DQA1*03:01	DQB1*03:02	DPA1*01:03	DPB1*04:02
**TIX042**	A2w1	-	A*68:01	B*39:05	C*07:02	DRB1*04:04	DRB4*01:03	DQA1*03:01	DQB1*03:02	DPA1*01:03	DPB1*04:01
			A*24:02	B*51:01	C*15:09	DRB1*14:02	DRB3*01:01	DQA1*05:03	DQB1*03:01	DPA1*01:03	DPB1*04:02
**TIX043**	A2	-	A*24:02	B*35:20	C*03:04	DRB1*04:07	DRB4*01:03	DQA1*03:01	DQB1*03:02	DPA1*01:03	DPB1*04:02
			A*68:03	B*39:05	C*07:02	DRB1*14:02	DRB3*01:01	DQA1*05:03	DQB1*03:01	DPA1*01:03	DPB1*04:02
**TIX044**	A2w1	-	A*31:01	B*35:01	C*07:02	DRB1*04:07	DRB4*01:03	DQA1*03:01	DQB1*03:02	DPA1*01:03	DPB1*04:02
			A*31:01	B*40:02	C*03:04	DRB1*04:11	DRB4*01:03	DQA1*03:01	DQB1*03:02	DPA1*02:01	DPB1*05:01
**TIX045**	B2t	Q1b1a1a	A*26:01	B*35:01	C*04:01	DRB1*04:02	DRB4*01:03	DQA1*03:01	DQB1*03:02	DPA1*01:03	DPB1*04:02
			A*68:03	B*35:12	C*07:02	DRB1*04:07	DRB4*01:03	DQA1*03:01	DQB1*03:02	DPA1*01:03	DPB1*04:02
**TIX046**	A2ad	-	A*31:01	B*35:01	C*07:02	DRB1*04:07	DRB4*01:03	DQA1*03:01	DQB1*03:02	DPA1*02:01	DPB1*05:01
			A*68:03	B*39:05	C*07:02	DRB1*04:07	DRB4*01:03	DQA1*03:01	DQB1*03:02	DPA1*01:03	DPB1*04:02
**TIX047**	A2	R1b1a1b1a1a2	A*26:01	B*45:01	C*06:02	DRB1*07:01	DRB4*01:01	DQA1*02:01	DQB1*02:02	DPA1*01:03	DPB1*04:02
			A*68:03	B*39:05	C*07:02	DRB1*04:07	DRB4*01:03	DQA1*03:01	DQB1*03:02	DPA1*01:03	DPB1*04:02
**TIX048**	C1c4	-	A*68:03	B*39:05	C*07:02	DRB1*04:07	DRB4*01:03	DQA1*03:01	DQB1*03:02	DPA1*01:03	DPB1*04:02
			A*24:02	B*39:06	C*07:02	DRB1*04:11	DRB4*01:03	DQA1*03:01	DQB1*03:02	DPA1*01:03	DPB1*04:02
**TIX049**	A2	-	A*68:03	B*35:43	C*01:02	DRB1*04:03	DRB4*01:03	DQA1*03:01	DQB1*03:02	DPA1*02:01	DPB1*14:01
			A*24:02	B*40:02	C*04:01	DRB1*04:03	DRB4*01:03	DQA1*03:01	DQB1*03:02	DPA1*01:03	DPB1*04:02
**TIX050**	A2r1	-	A*24:02	B*35:01	C*04:01	DRB1*04:07	DRB4*01:03	DQA1*03:01	DQB1*03:02	DPA1*01:03	DPB1*04:02
			A*11:01	B*35:03	C*04:01	DRB1*15:01	DRB5*01:01	DQA1*01:02	DQB1*06:02	DPA1*01:03	DPB1*04:02
**TIX051**	A2r1	G2a2b2a1a1c1a1a2a1a1a1	A*11:01	B*35:03	C*04:01	DRB1*15:01	DRB5*01:01	DQA1*01:02	DQB1*06:02	DPA1*01:03	DPB1*04:02
			A*24:02	B*39:06	C*07:02	DRB1*04:11	DRB4*01:03	DQA1*03:01	DQB1*03:02	DPA1*01:03	DPB1*04:02
**TIX052**	A2w1	-	A*24:02	B*15:01	C*01:02	DRB1*04:03	DRB4*01:03	DQA1*03:01	DQB1*03:02	DPA1*01:03	DPB1*04:02
			A*68:01	B*39:06	C*07:02	DRB1*04:04	DRB4*01:03	DQA1*03:01	DQB1*03:02	DPA1*01:03	DPB1*04:02
**TIX053**	B2	I2a1b1a2	A*31:01	B*40:08	C*03:04	DRB1*04:04	DRB4*01:03	DQA1*03:01	DQB1*03:02	DPA1*01:03	DPB1*04:02
			A*68:03	B*39:05	C*07:02	DRB1*04:07	DRB4*01:03	DQA1*03:01	DQB1*03:02	DPA1*01:03	DPB1*04:02
**TIX054**	D1	-	A*24:02	B*35:20	C*03:05	DRB1*04:03	DRB4*01:03	DQA1*03:01	DQB1*03:02	DPA1*01:03	DPB1*04:02
			A*24:02	B*40:02	C*03:04	DRB1*14:02	DRB3*01:01	DQA1*05:03	DQB1*03:01	DPA1*01:03	DPB1*04:02

Sample	mtDNA	Y-Chr	*HLA-A*	*HLA-B*	*HLA-C*	*HLA-DRB1*	*HLA-DRB3/4/5*	*HLA-DQA1*	*HLA-DQB1*	*HLA-DPA1*	*HLA-DPB1*
**TIX055**	D1	-	A*24:02	B*40:02	C*03:05	DRB1*04:04	DRB4*01:03	DQA1*03:01	DQB1*03:02	DPA1*01:03	DPB1*04:02
			A*68:03	B*39:05	C*07:02	DRB1*04:07	DRB4*01:03	DQA1*03:01	DQB1*03:02	DPA1*01:03	DPB1*04:02
**TIX056**	A2g	-	A*24:02	B*40:02	C*03:05	DRB1*04:07	DRB4*01:03	DQA1*03:01	DQB1*03:02	DPA1*01:03	DPB1*04:02
			A*24:02	B*40:02	C*03:05	DRB1*04:07	DRB4*01:03	DQA1*03:01	DQB1*03:02	DPA1*01:03	DPB1*04:02
**TIX057**	C1b14	G2a2b2a1a1c1a1a2a1a1a	A*31:01	B*40:08	C*03:04	DRB1*04:07	DRB4*01:03	DQA1*03:01	DQB1*03:02	DPA1*01:03	DPB1*04:02
			A*68:03	B*39:05	C*07:02	DRB1*04:07	DRB4*01:03	DQA1*03:01	DQB1*03:02	DPA1*01:03	DPB1*04:02
**TIX058**	A2	-	A*68:01	B*35:12	C*03:04	DRB1*04:07	DRB4*01:03	DQA1*03:01	DQB1*03:02	DPA1*01:03	DPB1*04:02
			A*24:02	B*35:20	C*04:01	DRB1*14:02	DRB3*01:01	DQA1*05:03	DQB1*03:01	DPA1*01:03	DPB1*04:02
**TIX059**	A2g	-	A*68:03	B*39:05	C*07:02	DRB1*04:07	DRB4*01:03	DQA1*03:01	DQB1*03:02	DPA1*01:03	DPB1*04:02
			A*24:02	B*35:12	C*04:01	DRB1*04:07	DRB4*01:03	DQA1*03:01	DQB1*03:02	DPA1*02:01	DPB1*14:01
**TIX060**	A2ap	-	A*31:01	B*35:01	C*07:02	DRB1*04:03	DRB4*01:03	DQA1*03:01	DQB1*03:02	DPA1*01:03	DPB1*04:02
			A*24:02	B*51:01	C*15:09	DRB1*14:02	DRB3*01:01	DQA1*05:03	DQB1*03:01	DPA1*01:03	DPB1*04:02
**TIX062**	A2ap	-	A*24:02	B*35:17	C*04:01	DRB1*14:02	DRB3*01:01	DQA1*05:03	DQB1*03:01	DPA1*01:03	DPB1*04:02
			A*24:02	B*51:01	C*15:09	DRB1*14:02	DRB3*01:01	DQA1*05:03	DQB1*03:01	DPA1*01:03	DPB1*04:02
**TIX063**	A2g	-	A*24:02	B*35:12	C*04:01	DRB1*04:07	DRB4*01:03	DQA1*03:01	DQB1*03:02	DPA1*01:03	DPB1*04:02
			A*68:01	B*40:02	C*03:05	DRB1*04:07	DRB4*01:03	DQA1*03:01	DQB1*03:02	DPA1*01:03	DPB1*04:02
**TIX064**	A2	-	A*68:01	B*35:12	C*04:01	DRB1*04:11	DRB4*01:03	DQA1*03:01	DQB1*03:02	DPA1*01:03	DPB1*04:02
			A*68:03	B*39:05	C*07:02	DRB1*08:02	Null	DQA1*04:01	DQB1*04:02	DPA1*01:03	DPB1*04:02
**TIX065**	A2ad	Q1b1a1a	A*24:02	B*35:20	C*04:01	DRB1*04:07	DRB4*01:03	DQA1*03:01	DQB1*03:02	DPA1*01:03	DPB1*04:02
			A*24:02	B*40:08	C*03:04	DRB1*04:07	DRB4*01:03	DQA1*03:01	DQB1*03:02	DPA1*01:03	DPB1*04:02
**TIX066**	A2w1	-	A*24:02	B*07:02	C*07:02	DRB1*03:01	DRB3*01:01	DQA1*05:01	DQB1*02:01	DPA1*01:03	DPB1*04:01
			A*31:01	B*35:20	C*04:01	DRB1*04:07	DRB4*01:03	DQA1*03:01	DQB1*03:02	DPA1*01:03	DPB1*04:02
**TIX067**	A2w1	-	A*68:01	B*35:20	C*04:01	DRB1*04:11	DRB4*01:03	DQA1*03:01	DQB1*03:02	DPA1*01:03	DPB1*04:02
			A*24:02	B*35:01	C*04:01	DRB1*04:25	DRB4*01:03	DQA1*03:03	DQB1*04:02	DPA1*01:03	DPB1*04:02
**TIX068**	C1c	Q1b1a2	A*68:01	B*39:05	C*07:02	DRB1*04:07	DRB4*01:03	DQA1*03:01	DQB1*03:02	DPA1*01:03	DPB1*04:02
			A*68:03	B*48:01	C*08:03	DRB1*04:04	DRB4*01:03	DQA1*03:01	DQB1*03:02	DPA1*01:03	DPB1*04:02
**TIX069**	A2m	-	A*31:01	B*40:08	C*03:04	DRB1*04:04	DRB4*01:03	DQA1*03:01	DQB1*03:02	DPA1*01:03	DPB1*04:02
			A*32:01	B*41:01	C*17:01	DRB1*03:01	DRB3*02:02	DQA1*05:01	DQB1*02:01	DPA1*01:03	DPB1*04:01
**TIX070**	D1j	-	A*02:06	B*35:01	C*07:02	DRB1*04:07	DRB4*01:03	DQA1*03:01	DQB1*03:02	DPA1*01:03	DPB1*04:02
			A*11:01	B*35:01	C*04:01	DRB1*01:01	Null	DQA1*01:01	DQB1*05:01	DPA1*01:03	DPB1*02:01
**TIX071**	A2ap	-	A*24:02	B*40:08	C*03:04	DRB1*04:07	DRB4*01:03	DQA1*03:01	DQB1*03:02	DPA1*01:03	DPB1*04:02
			A*24:02	B*51:01	C*15:09	DRB1*14:02	DRB3*01:01	DQA1*05:03	DQB1*03:01	DPA1*01:03	DPB1*04:02
**TIX072**	C1b14	-	A*68:01	B*39:05	C*07:02	DRB1*04:07	DRB4*01:03	DQA1*03:01	DQB1*03:02	DPA1*01:03	DPB1*04:02
			A*68:03	B*35:01	C*04:01	DRB1*13:02	DRB3*03:01	DQA1*01:02	DQB1*05:01	DPA1*02:01	DPB1*131:01
**TIX073**	A2g	-	A*68:03	B*35:01	C*07:02	DRB1*04:07	DRB4*01:03	DQA1*03:01	DQB1*03:02	DPA1*01:03	DPB1*04:02
			A*68:03	B*39:05	C*07:02	DRB1*04:07	DRB4*01:03	DQA1*03:01	DQB1*03:02	DPA1*01:03	DPB1*04:02
**TIX074**	A2g	-	A*68:03	B*35:01	C*07:02	DRB1*04:07	DRB4*01:03	DQA1*03:01	DQB1*03:02	DPA1*01:03	DPB1*04:02
			A*68:03	B*39:05	C*07:02	DRB1*04:07	DRB4*01:03	DQA1*03:01	DQB1*03:02	DPA1*01:03	DPB1*04:02
**TIX075**	C1c4	R1b1a1b1a1a2b	A*24:02	B*39:06	C*07:02	DRB1*04:11	DRB4*01:03	DQA1*03:01	DQB1*03:02	DPA1*01:03	DPB1*04:02
			A*26:01	B*45:01	C*06:02	DRB1*07:01	DRB4*01:01	DQA1*02:01	DQB1*02:02	DPA1*01:03	DPB1*04:02
**TIX076**	A2	-	A*68:03	B*35:12	C*07:02	DRB1*04:07	DRB4*01:03	DQA1*03:01	DQB1*03:02	DPA1*01:03	DPB1*04:02
			A*24:02	B*35:20	C*04:01	DRB1*04:07	DRB4*01:03	DQA1*03:01	DQB1*03:02	DPA1*01:03	DPB1*04:02
**TIX077**	A2ag	-	A*68:03	B*35:43	C*01:02	DRB1*04:03	DRB4*01:03	DQA1*03:01	DQB1*03:02	DPA1*01:03	DPB1*04:02
			A*02:01	B*35:12	C*04:01	DRB1*04:07	DRB4*01:03	DQA1*03:01	DQB1*03:02	DPA1*01:03	DPB1*04:02
**TIX078**	A2g	-	A*68:01	B*35:01	C*07:02	DRB1*04:07	DRB4*01:03	DQA1*03:01	DQB1*03:02	DPA1*01:03	DPB1*04:02

Sample	mtDNA	Y-Chr	*HLA-A*	*HLA-B*	*HLA-C*	*HLA-DRB1*	*HLA-DRB3/4/5*	*HLA-DQA1*	*HLA-DQB1*	*HLA-DPA1*	*HLA-DPB1*
			A*68:03	B*39:05	C*07:02	DRB1*04:07	DRB4*01:03	DQA1*03:01	DQB1*03:02	DPA1*01:03	DPB1*04:02

mtDNA: Mitochondrial DNA haplogroup; Y-Chr: Y chromosome haplotype.

Table S11. Frequencies of *HLA-A* alleles in Chichén Itzá and Tixcacaltuyub.

HLA allele	Chichén Itzá		Tixcacaltuyub		Significance	
	n= (2N=94)	Frequency	n= (2N=134)	Frequency	raw *p* value	p_{corr}
**A*02:01**	7	0.0745	4	0.0299	0.2073	1.0000
**A*02:06**	6	0.0638	3	0.0224	0.1666	1.0000
**A*03:01**	0	0.0000	1	0.0075	1.0000	1.0000
**A*11:01**	0	0.0000	4	0.0299	0.1449	1.0000
**A*23:01**	0	0.0000	1	0.0075	1.0000	1.0000
**A*24:02**	33	0.3511	39	0.2910	0.3858	1.0000
**A*26:01**	0	0.0000	3	0.0224	0.2699	1.0000
**A*29:02**	0	0.0000	1	0.0075	1.0000	1.0000
**A*31:01**	18	0.1915	20	0.1493	0.4710	1.0000
**A*31:09**	1	0.0106	0	0.0000	0.4123	1.0000
**A*32:01**	0	0.0000	3	0.0224	0.2699	1.0000
**A*68:01**	23	0.2447	18	0.1343	0.0367	0.5508
**A*68:03**	5	0.0532	36	0.2687	0.0000	**0.0003**
**A*68:05**	1	0.0106	0	0.0000	0.4123	1.0000
**A*80:01**	0	0.0000	1	0.0075	1.0000	1.0000

2N refers to the number of chromosomes analysed. p_{corr}: *p* values after correction. Significant p_{corr} values are in bold.

Table S12. Frequencies of *HLA-B* alleles in Chichén Itzá and Tixcacaltuyub.

HLA allele	Chichén Itzá		Tixcacaltuyub		Significance	
	n= (2N=94)	Frequency	n= (2N=134)	Frequency	raw *p* value	p_{corr}
**B*07:02**	0	0.0000	1	0.0075	1.0000	1.0000
**B*14:01**	0	0.0000	1	0.0075	1.0000	1.0000
**B*15:01**	6	0.0638	4	0.0299	0.3249	1.0000
**B*15:03**	0	0.0000	1	0.0075	1.0000	1.0000
**B*35:01**	25	0.2660	19	0.1418	0.0262	0.7062
**B*35:03**	0	0.0000	2	0.0149	0.5133	1.0000
**B*35:12**	2	0.0213	14	0.1045	0.0169	0.4573
**B*35:17**	5	0.0532	3	0.0224	0.2793	1.0000
**B*35:20**	4	0.0426	11	0.0821	0.2866	1.0000
**B*35:23**	1	0.0106	0	0.0000	0.4123	1.0000
**B*35:43**	0	0.0000	2	0.0149	0.5133	1.0000
**B*39:02**	6	0.0638	2	0.0149	0.0677	1.0000
**B*39:05**	5	0.0532	27	0.2015	0.0016	**0.0436**
**B*39:06**	1	0.0106	5	0.0373	0.4048	1.0000
**B*40:01**	0	0.0000	1	0.0075	1.0000	1.0000
**B*40:02**	23	0.2447	11	0.0821	0.0011	**0.0296**
**B*40:08**	8	0.0851	13	0.0970	0.8198	1.0000
**B*40:11**	1	0.0106	0	0.0000	0.4123	1.0000
**B*40:27**	1	0.0106	0	0.0000	0.4123	1.0000
**B*41:01**	0	0.0000	1	0.0075	1.0000	1.0000
**B*44:03**	0	0.0000	1	0.0075	1.0000	1.0000
**B*45:01**	0	0.0000	3	0.0224	0.2699	1.0000
**B*48:01**	3	0.0319	2	0.0149	0.4055	1.0000
**B*51:01**	1	0.0106	8	0.0597	0.0850	1.0000
**B*52:01**	2	0.0213	0	0.0000	0.1689	1.0000
**B*53:01**	0	0.0000	1	0.0075	1.0000	1.0000
**B*57:02**	0	0.0000	1	0.0075	1.0000	1.0000

2N refers to the number of chromosomes analysed. p_{corr}: p values after correction. Significant p_{corr} values are in bold.

Table S13. Frequencies of *HLA-C* alleles in Chichén Itzá and Tixcacaltuyub.

HLA allele	Chichén Itzá		Tixcacaltuyub		Significance	
	n= (2N=94)	Frequency	n= (2N=134)	Frequency	raw *p* value	p_{corr}
**C*01:02**	6	0.0638	6	0.0448	0.5582	1.0000
**C*02:10**	0	0.0000	1	0.0075	1.0000	1.0000
**C*03:03**	2	0.0213	0	0.0000	0.1689	1.0000
**C*03:04**	26	0.2766	23	0.1716	0.0715	1.0000
**C*03:05**	10	0.1064	5	0.0373	0.0555	0.8877
**C*04:01**	25	0.2660	30	0.2239	0.5300	1.0000
**C*06:02**	0	0.0000	3	0.0224	0.2699	1.0000
**C*07:02**	19	0.2021	53	0.3955	0.0023	**0.0369**
**C*08:01**	1	0.0106	0	0.0000	0.4123	1.0000
**C*08:02**	0	0.0000	1	0.0075	1.0000	1.0000
**C*08:03**	2	0.0213	2	0.0149	1.0000	1.0000
**C*15:02**	2	0.0213	0	0.0000	0.1689	1.0000
**C*15:09**	1	0.0106	7	0.0522	0.1448	1.0000
**C*16:01**	0	0.0000	1	0.0075	1.0000	1.0000
**C*17:01**	0	0.0000	1	0.0075	1.0000	1.0000
**C*18:02**	0	0.0000	1	0.0075	1.0000	1.0000

2N refers to the number of chromosomes analysed. p_{corr}: *p* values after correction. Significant p_{corr} values are in bold.

Table S14. Frequencies of *HLA-DRB1* alleles in Chichén Itzá and Tixcacaltuyub.

HLA allele	Chichén Itzá		Tixcacaltuyub		Significance	
	n= (2N=94)	Frequency	n= (2N=134)	Frequency	raw *p* value	p_{corr}
**DRB1*01:01**	0	0.0000	3	0.0224	0.2699	1.0000
**DRB1*03:01**	0	0.0000	2	0.0149	0.5133	1.0000
**DRB1*04:02**	0	0.0000	1	0.0075	1.0000	1.0000
**DRB1*04:03**	7	0.0745	8	0.0597	0.7874	1.0000
**DRB1*04:04**	10	0.1064	13	0.0970	0.8266	1.0000
**DRB1*04:05**	0	0.0000	1	0.0075	1.0000	1.0000
**DRB1*04:07**	22	0.2340	62	0.4627	0.0005	**0.0114**
**DRB1*04:10**	4	0.0426	0	0.0000	0.0278	0.6674
**DRB1*04:11**	18	0.1915	8	0.0597	0.0028	0.0662
**DRB1*04:17**	1	0.0106	0	0.0000	0.4123	1.0000
**DRB1*04:25**	0	0.0000	2	0.0149	0.5133	1.0000
**DRB1*07:01**	0	0.0000	2	0.0149	0.5133	1.0000
**DRB1*08:02**	5	0.0532	3	0.0224	0.2793	1.0000
**DRB1*09:01**	0	0.0000	1	0.0075	1.0000	1.0000
**DRB1*10:01**	0	0.0000	1	0.0075	1.0000	1.0000
**DRB1*11:01**	0	0.0000	1	0.0075	1.0000	1.0000
**DRB1*11:03**	0	0.0000	1	0.0075	1.0000	1.0000
**DRB1*13:02**	0	0.0000	3	0.0224	0.2699	1.0000
**DRB1*13:03**	0	0.0000	1	0.0075	1.0000	1.0000
**DRB1*14:02**	15	0.1596	15	0.1119	0.3237	1.0000
**DRB1*14:06**	4	0.0426	1	0.0075	0.1623	1.0000
**DRB1*15:01**	0	0.0000	2	0.0149	0.5133	1.0000
**DRB1*16:01**	0	0.0000	1	0.0075	1.0000	1.0000
**DRB1*16:02**	8	0.0851	2	0.0149	0.0176	0.4227

2N refers to the number of chromosomes analysed. p_{corr}: *p* values after correction. Significant p_{corr} values are in bold.

Table S15. Frequencies of *HLA-DRB3/4/5* alleles in Chichén Itzá and Tixcacaltuyub.

HLA allele	Chichén Itzá		Tixcacaltuyub		Significance	
	n= (2N=94)	Frequency	n= (2N=134)	Frequency	raw *p* value	p_{corr}
**DRB3*01:01**	19	0.2021	17	0.1269	0.1420	0.4259
**DRB3*02:02**	0	0.0000	4	0.0299	0.1449	0.4347
**DRB3*03:01**	0	0.0000	3	0.0224	0.2699	0.8096
**DRB4*01:01**	1	0.0106	3	0.0224	0.6448	1.0000
**DRB4*01:03**	61	0.6489	95	0.7090	0.3858	0.7716
**DRB5*01:01**	0	0.0000	2	0.0149	0.5133	1.0000
**DRB5*02:02**	8	0.0851	3	0.0224	0.0547	0.1093
**Null**	5	0.0532	7	0.0522	1.0000	1.0000

2N refers to the number of chromosomes analysed. p_{corr}: *p* values after correction. Significant p_{corr} values are in bold.

Table S16. Frequencies of *HLA-DQA1* alleles in Chichén Itzá and Tixcacaltuyub.

HLA allele	Chichén Itzá		Tixcacaltuyub		Significance	
	n= (2N=94)	Frequency	n= (2N=134)	Frequency	raw *p* value	p_{corr}
**DQA1*01:01**	0	0.0000	3	0.0224	0.2699	1.0000
**DQA1*01:02**	0	0.0000	6	0.0448	0.0438	0.4377
**DQA1*01:04**	0	0.0000	1	0.0075	1.0000	1.0000
**DQA1*02:01**	0	0.0000	3	0.0224	0.2699	1.0000
**DQA1*03:01**	48	0.5106	93	0.6940	0.0057	0.0575
**DQA1*03:03**	12	0.1277	3	0.0224	0.0022	**0.0218**
**DQA1*04:01**	5	0.0532	3	0.0224	0.2793	1.0000
**DQA1*05:01**	1	0.0106	2	0.0149	1.0000	1.0000
**DQA1*05:03**	20	0.2128	16	0.1194	0.0661	0.6615
**DQA1*05:05**	8	0.0851	4	0.0299	0.0771	0.7707

2N refers to the number of chromosomes analysed. p_{corr}: *p* values after correction. Significant p_{corr} values are in bold.

Table S17. Frequencies of *HLA-DQB1* alleles in Chichén Itzá and Tixcacaltuyub.

HLA allele	Chichén Itzá		Tixcacaltuyub		Significance	
	n= (2N=94)	Frequency	n= (2N=134)	Frequency	raw *p* value	p_{corr}
**DQB1*02:01**	0	0.0000	2	0.0149	0.5133	1.0000
**DQB1*02:02**	0	0.0000	4	0.0299	0.1449	1.0000
**DQB1*03:01**	25	0.2660	17	0.1269	0.0093	0.1115
**DQB1*03:02**	46	0.4894	94	0.7015	0.0015	**0.0177**
**DQB1*03:03**	3	0.0319	0	0.0000	0.0688	0.8251
**DQB1*03:04**	3	0.0319	2	0.0149	0.4055	1.0000
**DQB1*03:19**	0	0.0000	1	0.0075	1.0000	1.0000
**DQB1*04:02**	17	0.1809	4	0.0299	0.0001	**0.0016**
**DQB1*05:01**	0	0.0000	5	0.0373	0.0791	0.9496
**DQB1*05:02**	0	0.0000	1	0.0075	1.0000	1.0000
**DQB1*06:02**	0	0.0000	2	0.0149	0.5133	1.0000
**DQB1*06:04**	0	0.0000	2	0.0149	0.5133	1.0000

2N refers to the number of chromosomes analysed. p_{corr}: *p* values after correction. Significant p_{corr} values are in bold.

Table S18. Frequencies of *HLA-DPA1* alleles in Chichén Itzá and Tixcacaltuyub.

HLA allele	Chichén Itzá		Tixcacaltuyub		Significance	
	n= (2N=94)	Frequency	n= (2N=134)	Frequency	raw *p* value	p_{corr}
**DPA1*01:03**	82	0.8723	122	0.9104	0.3861	1.0000
**DPA1*02:01**	8	0.0851	9	0.0672	0.6183	1.0000
**DPA1*02:02**	4	0.0426	2	0.0149	0.2329	0.9315
**DPA1*03:01**	0	0.0000	1	0.0075	1.0000	1.0000

2N refers to the number of chromosomes analysed. p_{corr}: *p* values after correction. Significant p_{corr} values are in bold.

Table S19. Frequencies of *HLA-DPB1* alleles in Chichén Itzá and Tixcacaltuyub.

HLA allele	Chichén Itzá		Tixcacaltuyub		Significance	
	n= (2N=94)	Frequency	n= (2N=134)	Frequency	raw p value	p_{corr}
**DPB1*01:01**	0	0.0000	2	0.0149	0.5133	1.0000
**DPB1*02:01**	2	0.0213	5	0.0373	0.7028	1.0000
**DPB1*03:01**	3	0.0319	0	0.0000	0.0688	0.7563
**DPB1*04:01**	2	0.0213	5	0.0373	0.7028	1.0000
**DPB1*04:02**	76	0.8085	110	0.8209	0.8630	1.0000
**DPB1*05:01**	7	0.0745	7	0.0522	0.5789	1.0000
**DPB1*06:01**	0	0.0000	1	0.0075	1.0000	1.0000
**DPB1*11:01**	0	0.0000	1	0.0075	1.0000	1.0000
**DPB1*14:01**	4	0.0426	3	0.0224	0.4507	1.0000
**DPB1*105:01**	0	0.0000	2	0.0149	0.5133	1.0000
**DPB1*131:01**	0	0.0000	1	0.0075	1.0000	1.0000

2N refers to the number of chromosomes analysed. p_{corr}: p values after correction. Significant p_{corr} values are in bold.

Supplementary methods: Non-overlapping HLA associations.

Contact researcher: Bridget Penman.

Here we present examples of individual non-overlapping HLA associations analyses, to further illustrate the results related to this test. In the first case (Fig. S7), we present patterns obtained for the class I genes *HLA-B* and *HLA-C*, for which f^*_{adj} is high in both YCH and TIX populations, as illustrated in figure 4 in the main text. *HLA-B* and *HLA-C* are so close together that non overlapping associations between them (whilst perhaps driven by pathogens) are also unsurprising. It can be seen in Fig. S7 that whilst the level of non-overlap between *HLA-B* and *HLA-C* is similar for YCH and TIX, the highest frequency allelic associations are different in the two populations.

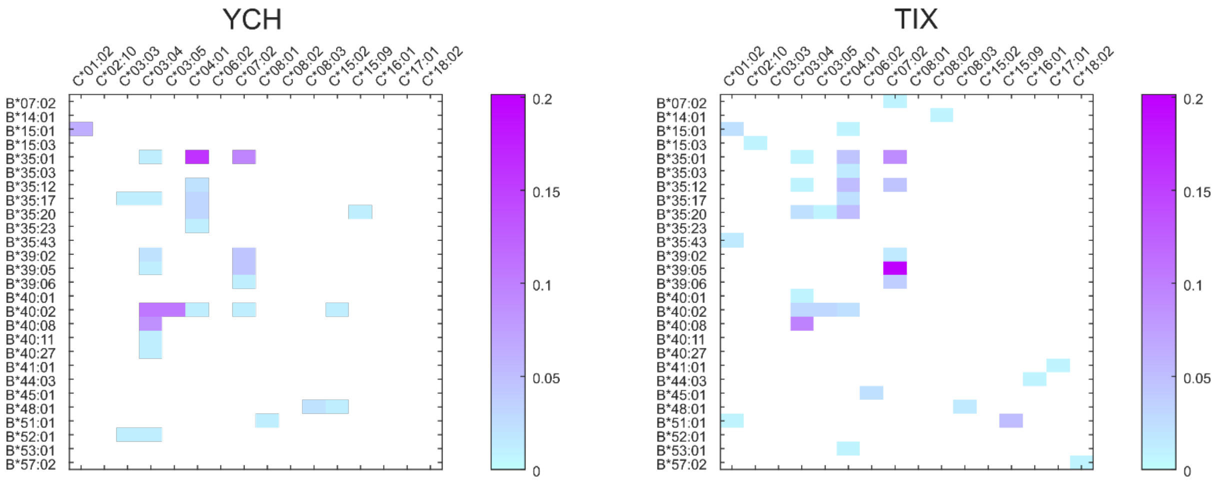

Figure S7. Frequencies of *HLA-B* and *HLA-C* associations in the ancient (YCH) and modern (TIX) populations.

Associations between *HLA-B* and *HLA-DRB1*, or between *HLA-B* and *HLA-DRB3/4/5* are relatively overlapping in the YCH population (lower f^*_{adj}) but non overlapping in the TIX population (higher f^*_{adj}), as shown in Fig. 4 of the main text. In Fig. S8, we show the specific allelic combinations present in the two populations, and use green ovals to highlight some of the features that are driving this pattern, i.e., high frequency alleles which share associations at relatively similar frequencies (overlap) in the YCH population, and high frequency alleles which do not share such associations, or where the shared associations are at very low frequencies (non-overlap) in the TIX population.

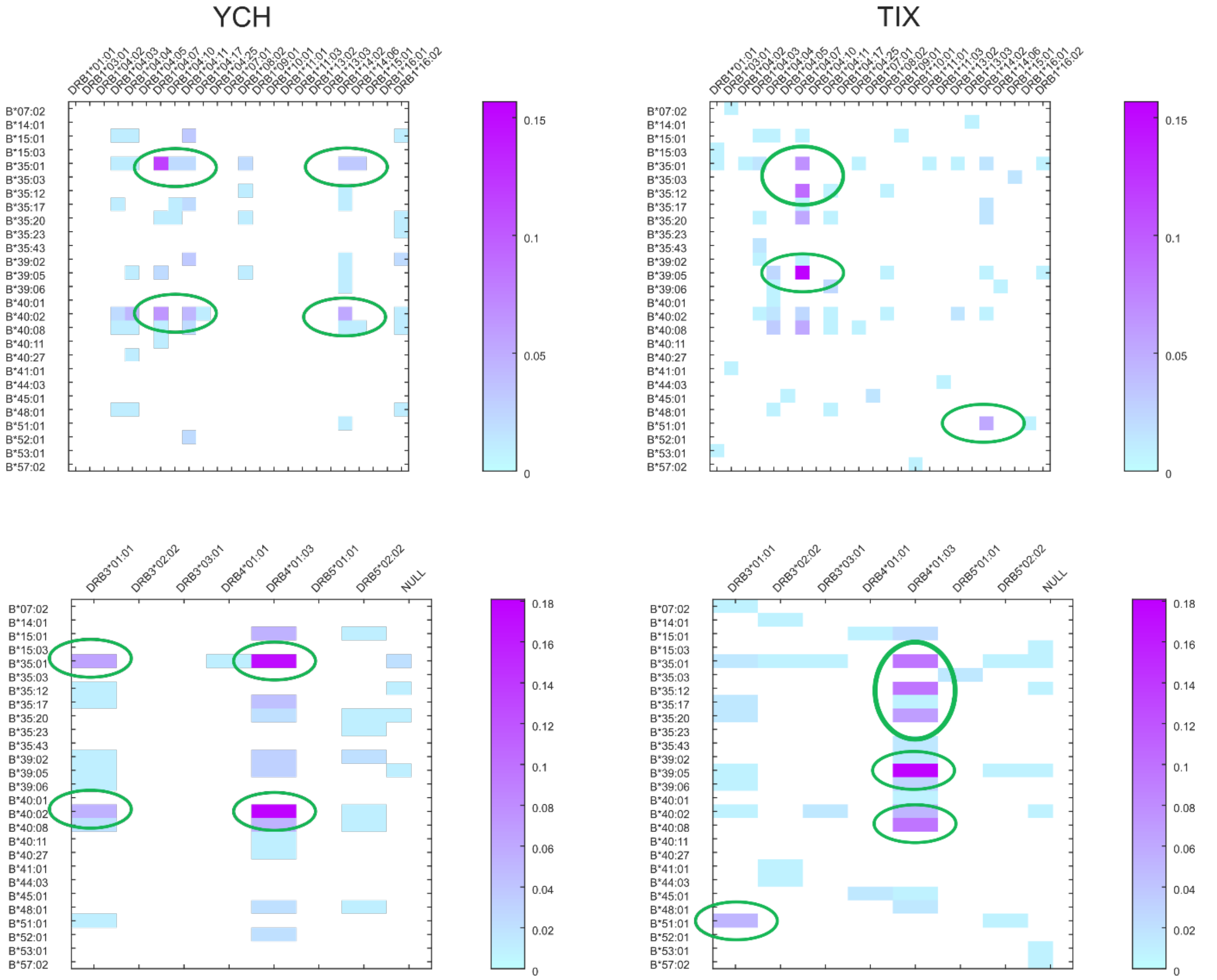

Figure S8. Frequencies of *HLA-B* and *HLA-DRB1* and *HLA-B* and *DRB3/4/5* associations in the ancient (YCH) and present day (TIX) populations.

Supplementary methods: *In-silico* binding prediction assays.

Contact researcher: Rodrigo Barquera.

Peptide-binding predictions between HLA molecules and peptides have been used previously to assess the role of HLA in the susceptibility or resistance to specific pathogens[70–72]. Here, we use this approach to analyse the binding strength of *Salmonella spp.* derived peptides to HLA class II alleles found in both ancient (YCH) and modern (TIX) Native Americans from the Maya region. For this test, we analysed the binding patterns of peptides (varying in length from 12-mers to 18-mers) derived from 18 proteins from *Salmonella spp.* against 25 HLA class II molecules. These proteins have been shown to produce an immunogenic response in the human body by means of the production of antibodies[73–79].

The sequence of the following proteins were extracted from the UniProt database[80] (primary UniProt accession number in parentheses) and included in our analyses: Secreted effector protein SseB (Q7BVH7), Flagellin (P06178), 60 kDa chaperonin (C0Q6A2), Outer membrane protein A (Q8Z7S0), Secreted effector protein SseJ (Q9FD10), Peptidoglycan-associated protein (A0A3Z2B3K0), Cytolysin A (B6E461), Nucleoside-specific channel-forming protein Tsx (P0A262), VacJ lipoprotein (C0PZX2), Outer membrane porin protein OmpD (Q5PHY0), Outer membrane protease OmpX (A0A3Y4CN55), Outer membrane protein W (Q8Z7E2), Outer membrane protein S1 (Q56110), Maltoporin (P26466), Sucrose porin (P22340), Outer membrane porin F (P37432), Outer membrane porin PhoE (P30705), and Outer membrane porin C (P0A264). This set of proteins was presented *in silico* against HLA molecules using netMHCIIpan 4.0[81]. The lengths of the peptides to be obtained were set to 12 to 18 residues, resulting in a set of 41,404 unique peptides. Due to the lack of inhibitory concentration (IC_{50}) values for some alleles, we used a ranking system as previously reported[72,81] to fairly compare all analysed alleles. For each HLA molecule, the percentile rank of the predicted binding score was used for detecting strong peptide binders and weak peptide binders by the recommended thresholds (%Rank < 2% for strong binders and 2% ≤ %Rank < 10% for weak binders, for HLA class II molecules).

Table S20. Binding prediction results for the *Salmonella enterica* peptides presented by HLA class II molecules.

HLA molecule	Strong binding		Weak binding	
	n=	%	n=	%
**DRB1*04:03**	355	9.96	1680	10.23
**DRB1*04:04**	187	5.25	1014	6.17
**DRB1*04:05**	150	4.21	1347	8.20
**DRB1*04:07**	542	15.21	2062	12.55
**DRB1*04:10**	226	6.34	1679	10.22
**DRB1*04:11**	274	7.69	1209	7.36
**DRB1*04:17**	421	11.81	1924	11.71
**DRB1*08:02**	220	6.17	1023	6.23
**DRB1*14:02**	561	15.74	1628	9.91
**DRB1*14:06**	196	5.50	926	5.64
**DRB1*16:02**	432	12.12	1936	11.78
**HLA molecule**				
**DRB3*01:01**	435	34.74	2312	41.28
**DRB4*01:01**	232	18.53	883	15.77
**DRB4*01:03**	197	15.73	908	16.21
**DRB5*02:02**	388	30.99	1498	26.75
**HLA molecule**				
**HLA-DQA1*03:01/DQB1*03:01**	859	11.78	2475	10.66
**HLA-DQA1*03:01/DQB1*03:02**	177	2.43	1566	6.75
**HLA-DQA1*03:01/DQB1*03:03**	917	12.57	2184	9.41
**HLA-DQA1*03:03/DQB1*04:02**	460	6.31	1919	8.27
**HLA-DQA1*04:01/DQB1*04:02**	206	2.82	1624	7.00
**HLA-DQA1*05:01/DQB1*03:01**	1192	16.34	4216	18.16
**HLA-DQA1*05:01/DQB1*03:04**	847	11.61	2454	10.57
**HLA-DQA1*05:05/DQB1*03:01**	824	11.30	2461	10.60
**HLA-DQA1*05:05/DQB1*03:02**	848	11.63	2121	9.14
**HLA-DQA1*05:05/DQB1*03:03**	964	13.22	2192	9.44

Strong binding: Rank value is on the top 2.0%. Weak binding: Rank value is between the top 2.0% and the top 10%. n: number of peptides presented by that specific allele. %: percentage of the total peptides presented by the HLA class II alleles studied, that are presented by that specific allele. Total number of peptides strongly bound to *HLA-DRB1* alleles: 3564. Total number of peptides strongly bound to *HLA-DRB3/4/5* alleles: 1252. Total number of peptides strongly bound to HLA-DQ molecules: 7294. Total number of peptides weakly bound to *HLA-DRB1* alleles: 16,428. Total number of peptides weakly bound to *HLA-DRB3/4/5* alleles: 5601. Total number of peptides weakly bound to HLA-DQ molecules: 23,212.

Supplementary Information: source populations for the population genetics analyses.

Category	Population or individual	Reference
Circum Arctic populations	Eskimo_ChaplinSireniki	82
	Eskimo_Naukan	83
	Greenland_Saqqaq.SG	84
	Aleut	82
	Alaskan_Athabskan.SG	85
	Canada_400BP.SG	86
	Canada_6000BP.SG	86
	Canada_BigBar_5700BP.SG	87
	Canada_MDorset_published	88
	Thule.SG	86
	Tlingit	82
	Tsimshian.SG	86
	USA_AK_Ancient_Athabaskan_1100BP	88
	USA_AK_NeoAleut_published	88
	USA_AK_PaleoAleut_published	88
	USA_AK_Prehistoric.SG	89
	USA_Alaska_TrailCreek_9000BP.SG	87
	USA_Ancient_Beringian.SG	90
	USA_Anzick_realigned.SG	91
SW USA	USA_CA_Early_SanNicolas.SG	85
	USA_CA_Late_SanNicolas.SG	85
	USA_Nevada_LovelockCave_1850BP.SG	87
	USA_Nevada_LovelockCave_600BP.SG	87
	USA_Nevada_SpiritCave_11000BP.SG	87
	USA_NM_Chaco	92
	USA_WA_Kennewick.SG	93
	Baja_Mexico.SG	85
	USA_CA_Late_SanNicolas.SG	85
	Island_Chumash_SanCruz.SG	85
	Island_Chumash_SanMiguel.SG	85
	Mainland_Chumash_NewCuyama.SG	85
	Mainland_Chumash.SG	85
Pima	Pima	94
Northern Mexico	Mexico_Pericues.SG	86
	Mexico_Pericues.SG_lc	86
	Mexico_PreColumbian_CH_MOM6.SG_lc	86
	Mexico_PreColumbian.SG_lc	86
Oaxaca	Mixe	82

Category	Population or individual	Reference
	Mixtec	82
Huichol	Huichol.SG	86
Mayan	Mayan	94
Mayan	Kaqchikel	95
Bahamas	Bahamas_AbacoIsl_Ceramic	96
	Bahamas_CrookedIsl_Ceramic	96
	Bahamas_EleutheraIsl_Ceramic	96
	Bahamas_LongIsl_Ceramic	96
	Bahamas_SouthAndros_Ceramic	96
	Bahamas_Taino.SG	97
Belize	Belize_EArchaic_published	98
Cuba	Cuba_CanimarAbajo_Archaic	99
	Cuba_CuevaCalero_Archaic	99
	Cuba_CuevaEsqueletos_Ceramic	99
	Cuba_CuevaPerico_Archaic	99
	Cuba_ElMorrillo_Ceramic	99
	Cuba_GuayaboBlanco_Archaic	99
	Cuba_LasCarolinas_Archaic	99
	Cuba_Manuelito_Archaic	99
	Cuba_PlayadelMango_Archaic	99
Curaçao	Curacao_deSavaan_Ceramic	96
	Curacao_SantaCruz_Ceramic	96
Dominican Republic	Dominican_Andres_Archaic	96
	Dominican_Andres_Ceramic	96
	Dominican_Atajadizo_Ceramic	96
	Dominican_Atajadizo_Ceramic_1d.rel.17903	96
	Dominican_Ceramic_JuanDolio	96
	Dominican_CuevaJuana_Ceramic	96
	Dominican_CuevaRoja_Archaic	96
	Dominican_EdilioCruz_Ceramic	96
	Dominican_ElFrances_Ceramic	96
	Dominican_ElSoco_Ceramic	96
	Dominican_LaCaleta_Ceramic	96
	Dominican_LaUnion_Ceramic	96
	Dominican_LomaPerenal_Ceramic	96
	Dominican_LosCorniel_Ceramic	96
	Dominican_LosMuertos_Ceramic	96
	Dominican_Macao_Ceramic	96

Category	Population or individual	Reference
Guadeloupe	Guadeloupe_AnseGourde_Ceramic	99
Haiti	Haiti_Diale1_Ceramic	96
St. Lucia	StLucia_Lavoutte_Ceramic	99
Puerto Rico	PuertoRico_CaboRojo11_Ceramic	96
	PuertoRico_CanasColloresMonserrate_Ceramic	96
	PuertoRico_Collores_Ceramic	96
	PuertoRico_LosIndios_Ceramic	99
	PuertoRico_Monserrate_Ceramic	96
	PuertoRico_PasodelIndio_Ceramic	99
	PuertoRico_PuntaCandelero_Ceramic	99
	PuertoRico_SantaElena_Ceramic	96
	PuertoRico_Tibes_Ceramic	99
Venezuela	Venezuela_LasLocas_Ceramic	99
	Piapoco	94
Bolivia	Aymara.SG	86
	Bolivia_MH_Iroco_1050BP	100
	Bolivia_MH_Miraflores	100
	Bolivia_MH_Tiwanaku	100
	Bolivian	82
Peru	Peru_Campanayuq_MH_1000BP_lc	82
	Peru_Chanka_LIP	100
	Peru_Cuncaicha_3300BP	101
	Peru_Cuncaicha_4200BP	101
	Peru_Cuncaicha_9000BP	101
	Peru_EIP_Moche	100
	Peru_ElBrujo_EIP_1300BP	100
	Peru_Highlands_Chinchawas_MH	100
	Peru_Highlands_LIP_Chimu	100
	Peru_HuacaPrieta_LA_4500BP	100
	Peru_Kaillachuro_Unknown.SG	102
	Peru_LaGalgada_4100BP	101
	Peru_Laramate_900BP	101
	Peru_Lauricocha_3500BP	101
	Peru_Lauricocha_5800BP	101
	Peru_Lauricocha_8600BP	101
	Peru_LH_Inca	100
	Peru_Lima_EIP_1450BP	100
	Peru_Lima_LIP_650BP	100
	Peru_LimaCoast_MH_1000BP	100

Category	Population or individual	Reference
	Peru_LIP_La_Galgada_600BP	100
	Peru_LIP_Ychsma	100
	Peru_MH_LIP_ElBrujo_850BP	100
	Peru_MH_LIP_Lambayeque	100
	Peru_Palpa_LIP_550BP	100
	Peru_Palpa_MH_950BP	100
	Peru_Paracas_EH_2250BP	100
	Peru_RioUncallane_1800BP.SG	102
	Peru_SanSebastian_LH_500BP	100
	Peru_SanSebastian_LIP_600BP	100
	Peru_SoroMikayaPatjxa_6800BP.SG	102
	Peru_Ullujaya_EIP_1350BP	100
	Peru_Ullujaya_MH_950BP	100
	Peru_WariHighlands_MH_lc	100
	Quechua	82
Brazil	Karitiana	103
	Brazil_Botocudo.SG	104
	Brazil_Enoque_HG.SG	86
	Brazil_Jabuticabeira2_2100BP	101
	Brazil_LapaDoSanto_9600BP	101
	Brazil_Laranjal_6700BP	101
	Brazil_Moraes_5800BP	101
	Brazil_Sumidouro_10100BP.SG	87
Argentina	Argentina_Aconcagua_Inca_500BP.SG	87
	Argentina_ArroyoSeco2_7700BP	101
	Argentina_BeagleChannel_Yamana_100BP.SG	86
	Argentina_LagunaChica_1600BP	101
	Argentina_LagunaChica_6800BP_published	101
	Argentina_LagunaToro_2400BP	100
	Argentina_NorthTierradelFiego_Selknam_100BP.SG	86
	Argentina_NorthTierradelFuego_LaArcillosa2_6000BP	86
	Argentina_Tierra_del_Fuego_brother.I12367	86
Chile	Chile_CaletaHuelen_MH_1100BP	100
	Chile_Chinchorro_LA.SG_lc	86
	Chile_Conchali_700BP	101
	Chile_LIP_Pukara_600BP	101
	Chile_LIP_Pukara_700BP	101
	Chile_LosRieles_12000BP.SG	101
	Chile_LosRieles_5100BP	101

Category	Population or individual	Reference
	Chile_PicaOcho_700BP	101
	Chile_PuntaSantaAna_7300BP.SG	87
	Chile_StraitOfMagellan_Kaweskar_100BP.SG	86
	Chile_WesternArchipelago_Ayayema_4700BP.SG	87
	Chile_WesternArchipelago_Kaweskar_800BP.SG	105
	Chile_Yamana_BeagleChannel_800BP.SG	105
	Yamana_BeagleChannel_Grouped_1900-500BP	105
	Haush_MitrePeninsula_Grouped_700BP	100
	Selknam_FaroMendez_100BP	86
	Selknam_NorthTierradelFuego_Grouped_500BP	86
	Aonikenk_SouthContinent_CerroJohnny_400BP	100

Supplementary methods: Community engagement activities.

Contact researchers: Rodrigo Barquera, Oana del Castillo-Chávez, Julio César Lara-Riegos, María Ermila Moo-Mezeta, Julio César Torres-Romero, Christina Warinner.

In April and May, 2023, a group of local researchers was joined by Rodrigo Barquera, María Ermila Moo-Mezeta and Julio César Lara-Riegos to take part in different activities with the goal of delivering the results of the analyses done for the present work, as well as to collect information on the views of different communities from Yucatán, Mexico, about the results presented. Such activities involved the presentation of the results to the students and teachers at the Autonomous University of Yucatan (UADY), and to students (junior high and high school levels) and participants from Tixcacaltuyub. Among other activities, the group delivered an introduction to the field of archaeogenetics, explained what motivated their research in this context and shared the main findings of the present work. To keep a dynamic that would allow for the exchange of thoughts on the findings and how are they perceived by the community, different strategies were used. Among the activities carried out, reciting a poem expressing the meaning of identity for the author, both in Mayan and Spanish, storytelling about what drove researchers and collaborators to work for the joint project, the distribution among students of colouring books[106] (please see: https://ecoevocommunity.nature.com/posts/29206-adventures-in-archaeological-science-a-colouring-book-for-young-scientists, for further information) both in Spanish and Yucatan Mayan as an introduction to bioarchaeology and archaeogenetics, and asking the audience about their perceptions of the results, can be listed as the ones that qualitatively yielded the most engagement from the audience. Among the reported perceptions, we could record overall excitement from the participants and students about the genetic continuity between the ancient individuals from Chichén Itzá and the participants from the community of Tixcacaltuyub, and questions about whether this genetic signature from Chichén Itzá could be found in other communities (whether close to Chichén Itzá or not) were raised, which could be further explored in future collaborations. Another set of colouring books was shared with students visiting the facilities of the Centro INAH Yucatán (Mérida, Yucatán, Mexico), in this case with more emphasis on the archaeological implications for these studies, led by Oana del Castillo-Chávez.

But political aspects are also involved in the work with communities which have contributed to research. As María Ermila Moo-Mezeta points out, "Being a research professor of indigenous origin has allowed me to have a global vision without sectarianizing the social reality of the municipality where I was born, I have closely felt the contradictions of a reality that is lived in Mayan communities and a system that does not respond to what is necessary to guarantee the rights of each person. I had the opportunity to have grown in the Mayan worldview, being clear about the social processes that occur within it, the particular characteristics of this population to transmit their knowledge, their customs and the way to perpetuate culture, I am part of a historical and collective memory of the Mayan people, my biggest challenge has been to establish strategies that allow me a critical look, a deep analysis of the population to which I belong to be able to contribute to academia and generate knowledge of what yesterday was mere daily life."

We consider that incorporating these views as part of our final results is fundamental, as these are the actual descendants from the people that once lived in these regions, and they are willing to reconcile our views and results with their own cosmogony: "In indigenous ways of knowing, we say that a thing cannot be understood until it is known by all four aspects of our being: mind, body, emotion and spirit"[107]. Inspired by the efforts of others[108,109], we translated (the translation is not peer-reviewed by the journal) the final version of the article into Spanish in order to make our research more accessible for communities involved in this and other research projects in the region.

Local researchers and collaborators leading the science communication activities: Oana del Castillo-Chávez (co-author), Julio Lara Riegos (co-author), María Ermila Moo-Mezeta (co-author), Pilar Márquez Vega (photographic documentation and logistic support), Mirna Canul Aké (translation of the colouring book into Yucatec Mayan); Margarita Zarco Salgado (Head of the Social Projects Unit, UADY; organisation, through those responsible for social projects developing in the community of Tixcacaltuyub, of the information delivered to the participants), Lifter Omar Ricalde Cab (responsible for the organisation of the delivery of information to students in the community), Miguel Güemez Pineda (responsible for the communication of information in the Mayan language to the adult participants of the study), Ramón Peniche Lara (research coordinator at UADY; management of resources for the trip to the community), José Esparza Bautista (community manager of Tixcacaltuyub; organisation with the commissioner of Tixcacaltuyub and municipal president of Yaxcabá of the meeting with the study participants), Marian Gabriel (social pedagogue; responsible for the intercultural dissemination strategy), and Lizbeth Carrillo Can (Mayan poet; declamation in Mayan and Spanish of the poem "Teen"/"Yo"/"I").

Supplementary references.

1. Hoggarth, J. A. *et al.* The political collapse of Chichén Itzá in climatic and cultural context. *Glob Planet Change* **138**, 25–42 (2016).
2. Ringle, W. M. On the political organization of Chichen Itza. *Ancient Mesoamerica* **15**, 167–218 (2004).
3. Tiesler, V. Cráneos perforados y tzompantlis en Chichén Itzá. *Arqueología Mexicana* **25**, 46–51 (2017).
4. Tiesler, V. & Cucina, A. Procedures in human heart extraction and ritual meaning: A taphonomic assessment of anthropogenic marks in Classic Maya skeletons. *Latin American Antiquity* **17**, 493–510 (2006).
5. Miller, V. E. Skeletons, skulls, and bones in the art of Chichén Itzá. in *New Perspectives on Human Sacrifice and Ritual Body Treatments in Ancient Maya Society* (eds. Tiesler, V. & Cucina, A.) 165–189 (Springer New York, New York, NY, 2007). doi:10.1007/978-0-387-48871-4_7.
6. Graña-Behrens, D., Prager, C. & Wagner, E. The hieroglyphic inscription of the 'High Priest's Grave' at Chichén Itzá, Yucatán, Mexico. *Mexicon* **21**, 61–66 (1999).
7. Dahlin, B. H. Climate change and the end of the Classic period in Yucatán. Resolving a paradox. *Ancient Mesoamerica* **13**, 327–340 (2002).
8. Roys, R. L. *The Book of Chilam Balam of Chumayel*. (University of Oklahoma Press, Norman, 1967).
9. Tozzer, A. M. *Chichen Itza and Its Cenote of Sacrifice: A Comparative Study of Contemporaneous Maya and Toltec. Memoirs of the Peabody Museum of Archaeology and Ethnology*. (Harvard University, Cambridge, 1957).
10. Anda, G. D., Tiesler, V. & Zabala, P. Cenotes, espacios sagrados y la práctica del sacrificio humano en Yucatán. in *Los Investigadores de la Cultura Maya* (ed. Universidad Autónoma de Campeche) vol. 12 376–386 (Universidad Autónoma de Campeche, Campeche, 2004).
11. Duncan, W. N. Bioarchaeological analysis of sacrificial victims from a Postclassic Maya temple from Ixlú, El Petén, Guatemala. *Latin American Antiquity* **22**, 549–572 (2011).
12. Hare, T., Masson, M. & Russell, B. High-Density LiDAR Mapping of the Ancient City of Mayapán. *Remote Sensing* vol. 6 9064–9085 Preprint at https://doi.org/10.3390/rs6099064 (2014).
13. Luzzadder-Beach, S. Water resources of the Chunchucmil Maya. *Geogr Rev* **90**, 493–510 (2000).
14. Luzzadder-Beach, S., Beach, T. P. & Dunning, N. P. Wetland fields as mirrors of drought and the Maya abandonment. *Proceedings of the National Academy of Sciences* **109**, 3646–3651 (2012).
15. Márquez Morfín, L. Sacrificio de niños en Chichen Itzá o práctica funeraria. in *Los niños como actores sociales ignorados. Levantando el velo, una mirada al pasado* (ed. Márquez Morfín, L.) 253–282 (ENAN-INAH; Conaculta, Mexico City, 2006).
16. Prout, M. G. & Brady, J. E. Paleodemographics of Child Sacrifice at Midnight Terror Cave: Reformulating the Emphasis of Maya Sacrificial Practices. *Archaeological Discovery* **06**, 1–20 (2018).
17. Ardren, T. Empowered children in Classic Maya sacrificial rites. *Childhood in the Past* **4**, 133–145 (2011).
18. Leod, B. mac & Puleston, D. E. Pathways into darkness: the search for the road to Xibalbá. in *Tercera Mesa Redonda de Palenque* (eds. Robinson, M. G. & Jeffers, D. C.) 71–77 (Herald Printers, Monterey, 1978).
19. Moyes, H. & Brady, J. E. Caves as sacred space in Mesoamerica. in *Sacred darkness: a global perspective on the ritual use of caves* (ed. Moyes, H.) 151–170 (University Press of Colorado, Boulder, 2014).
20. Márquez Morfín, L. & Schmidt, P. Osario infantil en un chultún en Chichén Itzá. in *Investigaciones recientes en el área maya. Vol. II. Memorias de la XVII Mesa Redonda (1981)* (ed. Sociedad Mexicana de Antropología) 89–104 (Sociedad Mexicana de Antropología; XVII Mesa Redonda, San Cristobal de las Casas, 1984).
21. Lee-Thorp, J. A. On isotopes and old bones*. *Archaeometry* **50**, 925–950 (2008).
22. Vogel, J. C. & van der Merwe, N. J. Isotopic Evidence for Early Maize Cultivation in New York State. *Am Antiq* **42**, 238–242 (1977).
23. Roberts, P. Isotope analysis in archaeology grand challenge. *Frontiers in Environmental Archaeology* **1**, (2022).
24. Błaszczyk, D. *et al.* Social status and diet. Reconstruction of diet of individuals buried in some early medieval chamber graves from Poland by carbon and nitrogen stable isotopes analysis. *J Archaeol Sci Rep* **38**, 103103 (2021).
25. Pérez-Ramallo, P. *et al.* Stable isotope analysis and differences in diet and social status in northern Medieval Christian Spain (9th–13th centuries CE). *J Archaeol Sci Rep* **41**, 103325 (2022).
26. Ambrose, S. H. & Norr, L. Experimental evidence for the relationship of the carbon isotope ratios of whole diet and dietary protein to those of bone collagen and carbonate. in *Prehistoric human bone: archaeology at the molecular level* (eds. Lambert, J. B. & Grupe, G.) 1–37 (Springer-Verlag, Berlin, 1993).
27. Smith, B. N. & Epstein, S. Two categories of 13C/12C ratios for higher plants. *Plant Physiol* **47**, 380–384 (1971).

28. Farquhar, G. D., Ehleringer, J. R. & Hubick, K. T. Carbon Isotope Discrimination and Photosynthesis. *Annu Rev Plant Physiol Plant Mol Biol* **40**, 503–537 (1989).
29. Tieszen, L. L. Natural variations in the carbon isotope values of plants: Implications for archaeology, ecology, and paleoecology. *J Archaeol Sci* **18**, 227–248 (1991).
30. DeNiro, M. J. & Epstein, S. Influence of diet on the distribution of nitrogen isotopes in animals. *Geochimicia et Cosmochimica Acta* **45**, 341–351 (1981).
31. O'Leary, M. H. Carbon Isotopes in Photosynthesis. *Bioscience* **38**, 328–336 (1988).
32. Sponheimer, M. *et al.* Isotopic Evidence for Dietary Variability in the Early Hominin Paranthropus robustus. *Science (1979)* **314**, 980–982 (2006).
33. Ambrose, S. H. Effects of diet, climate and physiology on nitrogen isotope abundances in terrestrial foodwebs. *J Archaeol Sci* **18**, 293–317 (1991).
34. Hedges, R. E. M. & Reynard, L. M. Nitrogen isotopes and the trophic level of humans in archaeology. *J Archaeol Sci* **34**, 1240–1251 (2007).
35. Dufour, E., Bocherens, H. & Mariotti, A. Palaeodietary implications of isotopic variability in Eurasian lacustrine fish. *J Archaeol Sci* **26**, 617–627 (1999).
36. Schoeninger, M. J. & DeNiro, M. J. Nitrogen and carbon isotopic composition of bone collagen from marine and terrestrial animals. *Geochim Cosmochim Acta* **48**, 625–639 (1984).
37. Richards, M. P. & Hedges, R. E. M. Stable isotope evidence for similarities in the types of marine foods used by late Mesolithic humans at sites along the Atlantic coast of Europe. *J Archaeol Sci* **26**, 717–722 (1999).
38. Kieffer Nail, C. L. The structural violence of Maya sacrifice: a case study of ritualized human sacrifice at Midnight Terror Cave, Belize. (The University of New Mexico, Alburquerque, 2018).
39. Wright, L. E. & White, C. D. Human biology in the Classic Maya collapse: Evidence from paleopathology and paleodiet. *J World Prehist* **10**, 147–198 (1996).
40. Scherer, A. K. Bioarchaeology and the Skeletons of the Pre-Columbian Maya. *Journal of Archaeological Research* **25**, 133–184 (2017).
41. Somerville, A. D., Fauvelle, M. & Froehle, A. W. Applying new approaches to modeling diet and status: isotopic evidence for commoner resiliency and elite variability in the Classic Maya lowlands. *J Archaeol Sci* **40**, 1539–1553 (2013).
42. North, B. V, Curtis, D. & Sham, P. C. A Note on the Calculation of Empirical P Values from Monte Carlo Procedures. *The American Journal of Human Genetics* **71**, 439–441 (2002).
43. Reimer, P. J. *et al.* The IntCal20 Northern Hemisphere Radiocarbon Age Calibration Curve (0–55 cal kBP). *Radiocarbon* **62**, 725–757 (2020).
44. Wright, L. E. The sacrifice of the Earth? : diet, health, and inequality in the Pasión Maya lowlands. (University of Chicago, Chicago, 1994).
45. White, C. D., Pendergast, D. M., Longstaffe, F. J. & Law, K. R. Social Complexity and Food Systems at Altun Ha, Belize: The Isotopic Evidence. *Latin American Antiquity* **12**, 371–393 (2001).
46. Gerry, J. P. & Joyce, R. Diet and status among the Classic Maya: An isotopic perspective. *ProQuest Dissertations and Theses* (United States -- Massachusetts, 1993).
47. Ebert, C. E., Peniche May, N., Culleton, B. J., Awe, J. J. & Kennett, D. J. Regional response to drought during the formation and decline of Preclassic Maya societies. *Quat Sci Rev* **173**, 211–235 (2017).
48. Ebert, C. E., Hoggarth, J. A., Awe, J. J., Culleton, B. J. & Kennett, D. J. The Role of Diet in Resilience and Vulnerability to Climate Change among Early Agricultural Communities in the Maya Lowlands. *Curr Anthropol* **60**, 589–601 (2019).
49. Ebert, C. E. *et al.* Sulfur isotopes as a proxy for human diet and mobility from the preclassic through colonial periods in the Eastern Maya lowlands. *PLoS One* **16**, e0254992- (2021).
50. Rand, A. J. Prehispanic and colonial Maya subsistence and migration: contributions from stable sulfur isotope analysis. (Memorial University of Newfoundland, St. John's, 2021).
51. Parker, D. Y. R. Late and Terminal Classic Maya Subsistence: Stable Isotope Analysis at Chac Balam and San Juan on Northern Ambergris Caye, Belize. (University of Texas at Arlington, Arlington, 2011).
52. Metcalfe, J. Z. *et al.* Isotopic Evidence for Diet at Chau Hiix, Belize: Testing Regional Models of Hierarchy and Heterarchy. *Latin American Antiquity* **20**, 15–36 (2009).
53. López, C. M., Núñez, L. F., Morales, P., Cienfuegos, E. & Otero, F. Diet and health at Chinikihá, Chiapas, Mexico: some preliminary results. *Environmental Archaeology* **16**, 82–96 (2011).
54. White, C. D. & Schwarcz, H. P. Ancient Maya diet: as inferred from isotopic and elemental analysis of human bone. *J Archaeol Sci* **16**, 451–474 (1989).
55. Williams, J. S., White, C. D. & Longstaffe, F. J. Maya Marine Subsistence: Isotopic Evidence from Marco Gonzalez and San Pedro, Belize. *Latin American Antiquity* **20**, 37–56 (2009).
56. Kennett, D. J. *et al.* Drought-Induced Civil Conflict Among the Ancient Maya. *Nat Commun* **13**, 3911 (2022).

57. Kennett, D. J., Masson, M. A., Serafin, S., Culleton, B. J. & Lope, C. P. War and Food Production at the Postclassic Maya City of Mayapán. in *The Archaeology of Food and Warfare: Food Insecurity in Prehistory* (eds. VanDerwarker, A. M. & Wilson, G. D.) 161–192 (Springer International Publishing, Cham, 2016). doi:10.1007/978-3-319-18506-4_9.
58. Williams, J. S., Stronge, S. M., Iannone, G. & Longstaffe, F. J. Examining chronological trends in ancient Maya diet at Minanha, Belize, using the stable isotopes of carbon and nitrogen. *Latin American Antiquity* **28**, 269–287 (2017).
59. White, C. D., Healy, P. F. & Schwarcz, H. P. Intensive Agriculture, Social Status, and Maya Diet at Pacbitun, Belize. *J Anthropol Res* **49**, 347–375 (1993).
60. Scherer, A. K., Wright, L. E. & Yoder, C. J. Bioarchaeological Evidence for Social and Temporal Differences in Diet at Piedras Negras, Guatemala. *Latin American Antiquity* **18**, 85–104 (2007).
61. Mansell, E. B., Tykot, R. H., Freidel, D. A., Dahlin, B. H. & Ardren, T. Early to Terminal Classic Maya diet in the Northern Lowlands of the Yucatán (Mexico). in *Histories of Maize: Multidisciplinary Approaches to the Prehistory, Linguistics, Biogeography, Domestication, and Evolution of Maize* (eds. Staller, J., Tykot, R. & Benz, B.) 173–185 (Elsevier, Amsterdam, 2010).
62. Ramsey, C. B. Bayesian analysis of radiocarbon dates. *Radiocarbon* **51**, 337–360 (2009).
63. Rohrlach, A. B., Tuke, J., Popli, D. & Haak, W. BREADR: An R Package for the Bayesian Estimation of Genetic Relatedness from Low-coverage Genotype Data. *bioRxiv* 2023.04.17.537144 (2023) doi:10.1101/2023.04.17.537144.
64. Gansauge, M.-T. & Meyer, M. Single-stranded DNA library preparation for the sequencing of ancient or damaged DNA. *Nat Protoc* **8**, 737 (2013).
65. Gansauge, M.-T. & Meyer, M. A method for single-stranded ancient DNA library preparation. in *Ancient DNA: Methods and Protocols* (eds. Shapiro, B. et al.) 75–83 (Springer New York, New York, NY, 2019). doi:10.1007/978-1-4939-9176-1_9.
66. Schraiber, J. G. Assessing the relationship of ancient and modern populations. *Genetics* **208**, 383–398 (2018).
67. Koren, S. *et al.* Pan troglodytes isolate AG18354, whole genome shotgun sequencing project. https://www.ncbi.nlm.nih.gov/nuccore/JAQQLN000000000.1.
68. Korneliussen, T. S., Albrechtsen, A. & Nielsen, R. ANGSD: Analysis of Next Generation Sequencing Data. *BMC Bioinformatics* **15**, 356 (2014).
69. Purcell, S. *et al.* PLINK: A Tool Set for Whole-Genome Association and Population-Based Linkage Analyses. *The American Journal of Human Genetics* **81**, 559–575 (2007).
70. Nguyen, A. *et al.* Human Leukocyte Antigen Susceptibility Map for Severe Acute Respiratory Syndrome Coronavirus 2. *J Virol* **94**, (2020).
71. Barquera, R. *et al.* Binding affinities of 438 HLA proteins to complete proteomes of seven pandemic viruses and distributions of strongest and weakest HLA peptide binders in populations worldwide. *HLA* **96**, 277–298 (2020).
72. Di, D., Simon Thomas, J., Currat, M., Nunes, J. M. & Sanchez-Mazas, A. Challenging Ancient DNA Results About Putative HLA Protection or Susceptibility to Yersinia pestis. *Mol Biol Evol* **39**, msac073 (2022).
73. Reynolds, C. J. *et al.* The serodominant secreted effector protein of Salmonella, SseB, is a strong CD4 antigen containing an immunodominant epitope presented by diverse HLA class II alleles. *Immunology* **143**, 438–446 (2014).
74. Wu, J. Y., Newton, S., Judd, A., Stocker, B. & Robinson, W. S. Expression of immunogenic epitopes of hepatitis B surface antigen with hybrid flagellin proteins by a vaccine strain of Salmonella. *Proc Natl Acad Sci U S A* **86**, 4726–4730 (1989).
75. Panchanathan, V. *et al.* Immunogenic epitopes of Salmonella typhi GroEL heat shock protein reactive with both monoclonal antibody and patients sera. *Immunol Lett* **62**, 105–109 (1998).
76. Pocanschi, C. L., Popot, J.-L. & Kleinschmidt, J. H. Folding and stability of outer membrane protein A (OmpA) from Escherichia coli in an amphipathic polymer, amphipol A8-35. *European Biophysics Journal* **42**, 103–118 (2013).
77. Alaniz, R. C., Deatherage, B. L., Lara, J. C. & Cookson, B. T. Membrane Vesicles Are Immunogenic Facsimiles of Salmonella typhimurium That Potently Activate Dendritic Cells, Prime B and T Cell Responses, and Stimulate Protective Immunity In Vivo. *The Journal of Immunology* **179**, 7692 LP – 7701 (2007).
78. Singh, R. *et al.* Low molecular weight proteins of outer membrane of Salmonella typhimurium are immunogenic in Salmonella induced reactive arthritis revealed by proteomics. *Clin Exp Immunol* **148**, 486–493 (2007).
79. Ortiz, V., Isibasi, A., García-Ortigoza, E. & Kumate, J. Immunoblot detection of class-specific humoral immune response to outer membrane proteins isolated from Salmonella typhi in humans with typhoid fever. *J Clin Microbiol* **27**, 1640 LP – 1645 (1989).

80. The UniProt Consortium. UniProt: the universal protein knowledgebase in 2021. *Nucleic Acids Res* **49**, D480–D489 (2021).
81. Reynisson, B., Alvarez, B., Paul, S., Peters, B. & Nielsen, M. NetMHCpan-4.1 and NetMHCIIpan-4.0: improved predictions of MHC antigen presentation by concurrent motif deconvolution and integration of MS MHC eluted ligand data. *Nucleic Acids Res* **48**, W449–W454 (2020).
82. Lazaridis, I. *et al.* Ancient human genomes suggest three ancestral populations for present-day Europeans. *Nature* **513**, 409–413 (2014).
83. Mallick, S. *et al.* The Simons Genome Diversity Project: 300 genomes from 142 diverse populations. *Nature* **538**, 201–206 (2016).
84. Rasmussen, M. *et al.* Ancient human genome sequence of an extinct Palaeo-Eskimo. *Nature* **463**, 757–762 (2010).
85. Scheib, C. L. *et al.* Ancient human parallel lineages within North America contributed to a coastal expansion. *Science (1979)* **360**, 1024–1027 (2018).
86. Raghavan, M. *et al.* Genomic evidence for the Pleistocene and recent population history of Native Americans. *Science (1979)* **349**, 1–20 (2015).
87. Moreno-Mayar, J. V. *et al.* Early human dispersals within the Americas. *Science (1979)* **362**, eaav2621 (2018).
88. Flegontov, P. *et al.* Palaeo-Eskimo genetic ancestry and the peopling of Chukotka and North America. *Nature* (2019) doi:10.1038/s41586-019-1251-y.
89. Lindo, J. *et al.* Ancient individuals from the North American Northwest Coast reveal 10,000 years of regional genetic continuity. *Proc Natl Acad Sci U S A* **114**, 4093–4098 (2017).
90. Moreno-Mayar, J. V. *et al.* Terminal Pleistocene Alaskan genome reveals first founding population of Native Americans. *Nature* **553**, 203–207 (2018).
91. Rasmussen, M. *et al.* The genome of a Late Pleistocene human from a Clovis burial site in western Montana. *Nature* **506**, 225–229 (2014).
92. Kennett, D. J. *et al.* Archaeogenomic evidence reveals prehistoric matrilineal dynasty. *Nat Commun* **8**, 14115 (2017).
93. Rasmussen, M. *et al.* The ancestry and affiliations of Kennewick Man. *Nature* **523**, 455–458 (2015).
94. Patterson, N. *et al.* Ancient admixture in human history. *Genetics* **192**, 1065–1093 (2012).
95. Reich, D., Patterson, N. & Campbell, D. Reconstructing native American population history. *Nature* **488**, 370–374 (2012).
96. Fernandes, D. M. *et al.* A genetic history of the pre-contact Caribbean. *Nature* **590**, 103–110 (2021).
97. Schroeder, H. *et al.* Origins and genetic legacies of the Caribbean Taino. *Proceedings of the National Academy of Sciences* **115**, 201716839 (2018).
98. Kennett, D. J. *et al.* South-to-north migration preceded the advent of intensive farming in the Maya region. *Nat Commun* **13**, 1530 (2022).
99. Nägele, K. *et al.* Genomic insights into the early peopling of the Caribbean. *Science (1979)* **369**, 456–460 (2020).
100. Nakatsuka, N. *et al.* A paleogenomic reconstruction of the deep population history of the Andes. *Cell* **181**, 1131-1145.e21 (2020).
101. Posth, C. *et al.* Reconstructing the Deep Population History of Central and South America. *Cell* **175**, 1185-1197.e22 (2018).
102. Lindo, J. *et al.* The genetic prehistory of the Andean highlands 7000 years BP though European contact. *Sci Adv* **4**, eaau4921 (2018).
103. Bergström, A. *et al.* Insights into human genetic variation and population history from 929 diverse genomes. *Science (1979)* **367**, (2020).
104. Malaspinas, A.-S. S. *et al.* Two ancient human genomes reveal Polynesian ancestry among the indigenous Botocudos of Brazil. *Current Biology* **24**, R1035–R1037 (2014).
105. de la Fuente, C. *et al.* Genomic insights into the origin and diversification of late maritime hunter-gatherers from the Chilean Patagonia. *Proc Natl Acad Sci U S A* **115**, E4006–E4012 (2018).
106. Warinner, C. & Hendy, J. *Adventures in Archaeological Science*. (Max Planck Institute for the Science of Human History, Jena, 2017).
107. Kimmerer, R. W. *Gathering Moss: A Natural and Cultural History of Mosses* . (Oregon State University Press, Oregon, 2003).
108. Jeong, C. *et al.* A Dynamic 6,000-Year Genetic History of Eurasia’s Eastern Steppe. *Cell* **183**, 890-904.e29 (2020).
109. Arango-Isaza, E. *et al.* Bridging the gap: returning genetic results to indigenous communities in Latin America. *Front Genet* **14**, (2023).
