# Supplementary material for: Ancient genomes reveal insights into ritual life at Chichén Itzá
Source: Nature. 2024 Jun 12;630(8018):912–9. doi: 10.1038/s41586-024-07509-7 (PMC11208145; doi:10.1038/s41586-024-07509-7)
Supplement: Supplementary file 2 — Reporting Summary [file 41586_2024_7509_MOESM2_ESM.pdf]

nature portfolio

Corresponding author(s): Rodrigo Barquera, Oana del Castillo-Chávez, Johannes Krause.

Last updated by author(s): 11/03/2024

Reporting Summary

Nature Portfolio wishes to improve the reproducibility of the work that we publish. This form provides structure for consistency and transparency in reporting. For further information on Nature Portfolio policies, see our Editorial Policies and the Editorial Policy Checklist.

Statistics

For all statistical analyses, confirm that the following items are present in the figure legend, table legend, main text, or Methods section.

n/a	Confirmed	
☐	☒	The exact sample size (*n*) for each experimental group/condition, given as a discrete number and unit of measurement
☐	☒	A statement on whether measurements were taken from distinct samples or whether the same sample was measured repeatedly
☐	☒	The statistical test(s) used AND whether they are one- or two-sided<br>*Only common tests should be described solely by name; describe more complex techniques in the Methods section.*
☐	☒	A description of all covariates tested
☐	☒	A description of any assumptions or corrections, such as tests of normality and adjustment for multiple comparisons
☐	☒	A full description of the statistical parameters including central tendency (e.g. means) or other basic estimates (e.g. regression coefficient) AND variation (e.g. standard deviation) or associated estimates of uncertainty (e.g. confidence intervals)
☐	☒	For null hypothesis testing, the test statistic (e.g. *F*, *t*, *r*) with confidence intervals, effect sizes, degrees of freedom and *P* value noted<br>*Give P values as exact values whenever suitable.*
☒	☐	For Bayesian analysis, information on the choice of priors and Markov chain Monte Carlo settings
☒	☐	For hierarchical and complex designs, identification of the appropriate level for tests and full reporting of outcomes
☒	☐	Estimates of effect sizes (e.g. Cohen's *d*, Pearson's *r*), indicating how they were calculated

Our web collection on statistics for biologists contains articles on many of the points above.

Software and code

Policy information about availability of computer code

Data collection	nf-core/eager ver. 2.3.4<br>Nextflow v20.10.0<br>FastQC v0.11.9<br>MultiQC v1.9<br>AdapterRemoval v2.3.1<br>fastP v0.20.1<br>BWA v0.7.12<br>Bowtie2 v2.4.1<br>mapDamage 2.0<br>Samtools v1.9<br>endorS.py v0.4<br>DeDup v0.12.7<br>Picard MarkDuplicates v2.22.9<br>Qualimap v2.2.2-dev<br>Preseq v2.0.3<br>pileupCaller ver. 8.2.2
Data analysis	Oxcal ver. 4.3<br>HaploGrep2<br>HAPLOFIND<br>biomaRt Release 3.15<br>OptiType, tag GRG<br>NetMHCIIpan-4.0<br>Smartpca ver. 16000

ADMIXTURE ver. 1.3.0
Plink ver. 1.90
Xerxes CLI software v. 0.3.0.0
Poseidon v. 2.5.0
qpWave ver. 420

For manuscripts utilizing custom algorithms or software that are central to the research but not yet described in published literature, software must be made available to editors and reviewers. We strongly encourage code deposition in a community repository (e.g. GitHub). See the Nature Portfolio guidelines for submitting code & software for further information.

Data

Policy information about availability of data

All manuscripts must include a data availability statement. This statement should provide the following information, where applicable:

- Accession codes, unique identifiers, or web links for publicly available datasets
- A description of any restrictions on data availability
- For clinical datasets or third party data, please ensure that the statement adheres to our policy

All of the genomic data (including nuclear DNA, mtDNA, and HLA alignment sequences) for the ancient Chichén Itzá individuals (YCH) are archived in the European Nucleotide Archive (ENA) database (accession number: PRJEB73567). Present-day genomic data of Tixcacaltuyub individuals (TIX) are archived in the (EGA) database (accession number: 10489) and will be made available on request to R.B., J.C.L.R. and J.K., and subject to a signed agreement to restrict usage to anonymised studies of population history. All HLA data from our sample sets, both frequencies and individual genotypes, can be found at The Allele Frequency Net Database website (www.allelefrequencies.net) under accession numbers: 3791 (YCH) and 3790 (TIX).

Field-specific reporting

Please select the one below that is the best fit for your research. If you are not sure, read the appropriate sections before making your selection.

☒ Life sciences ☐ Behavioural & social sciences ☐ Ecological, evolutionary & environmental sciences

For a reference copy of the document with all sections, see nature.com/documents/nr-reporting-summary-flat.pdf

Life sciences study design

All studies must disclose on these points even when the disclosure is negative.

Sample size	No statistical methods were used to determine ancient DNA sample size a priori. The number of genomes analyzed in this study depends on available human remains associated with hunter-gatherer individuals with signature of preserved ancient DNA. Those specimens are very limited because of the scarce availability and poor molecular preservation of human remains from that period.
Data exclusions	For ancient DNA screening analysis, libraries with less than ~0.1% human DNA and/or with no ancient DNA damage pattern are not carried on for SNPs capture. For alignment, sequencing reads with fragment length <30bp and mapping quality/base quality <30 are excluded from genotyping. For ancient DNA authentication, libraries with indication of substantial contamination levels or individually-analyzed libraries with marginal contamination levels are filtered to analyze only sequencing reads that carry signs of post-mortem DNA damage. For whole genome analysis, individuals with less than 20,000 usable SNPs are excluded.
Replication	Multiple libraries (1-6) are prepared for a subset of samples as replication. The genotypes from different libraries (both single stranded and double stranded) are merged for downstream analysis after confirming of similar statistical behaviour. Each sample is analyzed for up to 1.24 million markers across the human genome that represent an internal replication of the findings.
Randomization	Randomization is not relevant to this study. Samples are grouped based on sampling locations, dates and genetic affinities.
Blinding	Blinding is not applicable for ancient specimens as the sampling locations and dates are known as prior. In downstream data analysis blinding is also not relevant since the newly generated ancient genomes are co-analyzed with previously published present-day and ancient human genomes.

Reporting for specific materials, systems and methods

We require information from authors about some types of materials, experimental systems and methods used in many studies. Here, indicate whether each material, system or method listed is relevant to your study. If you are not sure if a list item applies to your research, read the appropriate section before selecting a response.

Materials & experimental systems

n/a	Involved in the study
☒	☐ Antibodies
☒	☐ Eukaryotic cell lines
☐	☒ Palaeontology and archaeology
☒	☐ Animals and other organisms
☐	☒ Human research participants
☒	☐ Clinical data
☒	☐ Dual use research of concern

Methods

n/a	Involved in the study
☒	☐ ChIP-seq
☒	☐ Flow cytometry
☒	☐ MRI-based neuroimaging

Palaeontology and Archaeology

Specimen provenance

The ancient samples used in this study belonged to individuals whose skeletons were recovered from the archaeological excavations of a chultún (cistern) connected to a natural cave within the archaeological site of Chichén Itzá, Yucatán, Mexico.

Specimen deposition

The skeletal remains analyzed in this study derive from a skeletal collection curated by O.C.C. and her team, stored at the Centro INAH Yucatan, in Merida, Yucatan, Mexico. The genetic libraries generated from DNA extracted from the skeletal remains are stored at the Max Planck Institute for Evolutionary Anthropology (MPI-EVA) in Jena and Leipzig, Germany.

Dating methods

According to the report issued by The Curt-Engelhorn-Centre for Archaeometry (Mannheim, Germany), the portions of the petrous bone of 26 YCH samples were pre-treated and analysed using a standardised procedure. Collagen was extracted from the bone samples (approx. 1 g, using a modified version of the Longin method), purified by ultrafiltration (fraction >30kD) and freeze-dried. The collagen was then combusted to CO2 in an Elemental Analyzer (EA). The CO2 was then converted catalytically to graphite and analysed using a MICADAS-type AMS system. The isotopic ratios 14C/12C and 13C/12C of samples, calibration standard (Oxalic Acid-II), blanks and control standards were measured simultaneously in the AMS system. 14C-ages are normalised to delta13C=-25‰120 with a typical uncertainty of 2‰, and calibrated using the dataset IntCal20 and the software Oxcal (ver. 4.3.2).

☒ Tick this box to confirm that the raw and calibrated dates are available in the paper or in Supplementary Information.

Ethics oversight

No ethical approval or guidance was required for the analyses carried on the archaeological material.

Note that full information on the approval of the study protocol must also be provided in the manuscript.

Human research participants

Policy information about studies involving human research participants

Population characteristics

The village of Tixcacaltuyub, part of the municipality of Yaxcabá, is located 90 km southeast of Merida, 16 km off Sotuta, and is located 55 Km southeast of Chichén Itzá. The community of Tixcacaltuyub self-identifies as a Mayan community and has been in a years-long cooperative relationship with the Chemistry and Nursing Faculties of the Universidad Autónoma de Yucatán (UADY), Mérida, Yucatán, following projects investigating the relationship between health and lifestyle in the community.

Recruitment

Ethical approval for the collection of blood samples from the individuals from Tixcacaltuyub, Yucatán, Mexico, was granted by the Committee of Ethics and Research, Autonomous University of Yucatán (UADY), Mexico (Project: Bienestar Comunitario: Proyecto de capacitación para la autogestión de la salud de personas con DT2 y sus familias, en la comunidad de Tixcacaltuyub y Yaxcabá; official notice number: F-FENC-SAC-14/REV: 04; Registry number: 09/17), and performed according to the requisites of the Helsinki Declaration (2008) and the General Health Law of Mexico. All subjects were informed about the objectives and methods used, and signed an informed consent form.

Ethics oversight

Committee of Ethics and Research, Autonomous University of Yucatán (UADY), Mexico.

Note that full information on the approval of the study protocol must also be provided in the manuscript.
