# Supplementary material for: Ancient genomes reveal insights into ritual life at Chichén Itzá
Source: Nature. 2024 Jun 12;630(8018):912–9. doi: 10.1038/s41586-024-07509-7 (PMC11208145; doi:10.1038/s41586-024-07509-7)
Supplement: Supplementary file 3 — The non-peer-reviewed translation of the main article into Spanish. [file 41586_2024_7509_MOESM3_ESM.pdf]

Genomas antiguos revelan información sobre la vida ritual en Chichén Itzá

Rodrigo Barquera[1,2,§,*], Oana Del Castillo-Chávez[3,§,*], Kathrin Nägele[1,§], Patxi Pérez-Ramallo[4,5,6,§,#], Diana Iraíz Hernández-Zaragoza[1,2,§], András Szolek[7,8], Adam Benjamin Rohrlach[1,9], Pablo Librado[10], Ainash Childebayeva[1,±], Raffaela Angelina Bianco[1], Bridget S. Penman[11], Victor Acuña-Alonzo[2], Mary Lucas[4,6], Julio César Lara-Riegos[12], María Ermila Moo-Mezeta[13], Julio César Torres-Romero[12], Patrick Roberts [4,6], Oliver Kohlbacher[7,14,15,16], Christina Warinner[1,17], Johannes Krause[1]*.

La antigua ciudad de Chichén Itzá en Yucatán, México fue uno de los más grandes e influyentes asentamientos mayas durante el periodo Clásico Tardío y Terminal (600-1000 AD) y continúa siendo uno de los sitios arqueológicos más estudiados en Mesoamérica. Sin embargo, muchas cuestiones acerca del uso social y cultural de espacios ceremoniales, así como su afinidad genética con otros pueblos mesoamericanos, permanecen sin respuesta. Aquí presentamos datos genómicos de 64 individuos sub-adultos que datan de aproximadamente 500-900 AD, encontrados en un entierro masivo subterráneo cerca del Cenote Sagrado en el centro ceremonial de Chichén Itzá. Los análisis genéticos revelaron que todos los individuos analizados eran varones y que varios individuos estaban estrechamente relacionados, incluidos dos pares de gemelos monocigóticos. Los gemelos destacan en la mitología maya y mesoamericana en general, pues personifican cualidades de dualidad entre las deidades y los héroes, pero hasta ahora no habían sido identificados en contextos funerarios mayas antiguos. La comparación genética con población actual de la región muestra continuidad genética con los antiguos habitantes de Chichén Itzá, excepto en ciertos loci genéticos relacionados con la inmunidad humana, incluido el sistema HLA, sugiriendo señales de adaptación debido a enfermedades infecciosas introducidas en la región durante la época colonial.

[1]Departamento de Arqueogenética, Instituto Max-Planck de Antropología Evolutiva (MPI-EVA), Leipzig, Alemania. [2]Laboratorio de Genética Molecular, Escuela Nacional de Antropología e Historia (ENAH), Ciudad de México, México. [3]Centro INAH Yucatán, Instituto Nacional de Antropología e Historia (INAH), Mérida, Yucatán, México. [4]Grupo de Investigación isoTROPIC, Instituto Max Planck de Geoantropología, Jena, Alemania. [5]Universidad del País Vasco (EHU), San Sebastián-Donostia, España. [6]Departamento de Arqueología, Instituto Max Planck de Geoantropología, Jena, Alemania. [7]Bioinformática Aplicada, Departamento de Informática, Universidad de Tübingen, Tübingen, Alemania. [8]Departamento de Inmunología, Instituto Interfacultativo de Biología Celular, Universidad de Tübingen, Tübingen, Alemania. [9]Escuela de Ciencias Informáticas y Matemáticas, Universidad de Adelaide, Adelaide, SA, Australia. [10]Institut de Biología Evolutiva (CSIC – Universitat Pompeu Fabra), Barcelona, España. [11]Instituto Zeeman y Facultad de Ciencias de la Vida, Universidad de Warwick, Coventry, Reino Unido. [12]Facultad de Química, Universidad Autónoma de Yucatán (UADY), Mérida, Yucatán, México. [13]Facultad de Enfermería, Universidad Autónoma de Yucatán (UADY), Mérida, Yucatán, México. [14]Instituto de Bioinformática e Informática Médica, Universidad de Tübingen, Tübingen, Alemania. [15]Centro de Biología Cuantitativa, Universidad de Tübingen, Tübingen, Alemania. [16]Bioinformática Traslacional, Hospital Universitario de Tübingen, Tübingen, Alemania. [17]Departamento de Antropología, Universidad de Harvard, Cambridge, MA, EE.UU.

§ Estos autores contribuyeron igualmente al presente trabajo. # Adscripción actual: Department of Archaeology and Cultural History, University Museum, Norwegian University of Science and Technology (NTNU), Trondheim, Norway. ± Adscripción actual: Department of Anthropology, University of Texas at Austin, Austin, Texas, USA.

***Correspondencia: Rodrigo Barquera.** Email: rodrigo_barquera@eva.mpg.de; **Oana Del Castillo-Chávez.** Email: oana_delcastillo@inah.gob.mx; **Johannes Krause.** Email: krause@eva.mpg.de.

Principal

La antigua ciudad Maya de Chichén Itzá, ubicada en la parte centro-norte de la península de Yucatán (Fig. 1A-1B) se encuentra entre los sitios arqueológicos más grandes e icónicos en Mesoamérica, pero mucho sobre sus orígenes e historia continúa siendo vagamente comprendido[1,2]. El primer periodo de esplendor fue durante el periodo Clásico Tardío (600-800 AD), Chichén Itzá se convirtió en el centro político dominante de las tierras bajas mayas durante el Clásico Terminal (800-1000 AD), un periodo en el que la mayoría de los otros sitios mayas Clásicos en las tierras bajas del sur y del norte experimentaron un colapso político. La mayoría de las fechas del calendario inscritas en monumentos tallados en Chichén Itzá se encuentran entre los años 850 y 875 AD, y el centro ceremonial al norte de sitio, conocido como el "Nuevo Chichén", fue construido en su mayoría después del 900 AD e incluye la estructura más grande del sitio, *El Castillo*, también conocido como el Templo de Kukulkan. Un *Sacbé* (camino de piedra caliza) fue construido para conectar Nuevo Chichén con el Cenote Sagrado[6,] un enorme cenote que contiene abundantes ofrendas rituales, incluyendo los restos de más de doscientos individuos sacrificados ritualmente, la mayoría de los cuales son niños [1,3,7]. La evidencia de sacrificios rituales es extensa a lo largo del sitio de Chichén Itzá, e incluye tanto restos físicos de los individuos sacrificados como representaciones en arte monumental[8]. La actividad de élite en Chichen Itzá disminuyó durante el siglo XI AD, con una última fecha calendárica inscrita en 998 AD [9,10,] pero el sitio continuó siendo un prominente centro ritual y de peregrinación a lo largo del periodo colonial e incluso después de éste[11–13].

En 1967, un chultún que contenía más de un centenar de sub-adultos fue descubierto cerca del Cenote Sagrado (Fig. 1C-1D) [4,14,15]. Al igual que los cenotes (cisternas subterráneas), los chultunes están asociados con el almacenamiento de agua y también con la actividad ritual [16,17] y comparten un simbolismo con las cuevas[18]. Tales características subterráneas han sido durante largo tiempo asociadas con el agua, la lluvia y el sacrificio infantil, y son ampliamente consideradas como puntos de acceso al inframundo Maya. Dada la ubicación y el contexto del chultún en Chichén Itzá, que también estaba conectado a una pequeña cueva subterránea, se ha especulado que contiene sacrificios infantiles para apoyar los ciclos agrícolas del maíz[19] o entregados como ofrendas a la deidad maya de la lluvia, Chaac[20]. Los relatos coloniales del s. XVI y las investigaciones de principios del s. XX tras el desagüe del Cenote Sagrado popularizaron la idea de que principalmente se sacrificaban mujeres jóvenes y niñas en el sitio[21,22], pero análisis osteológicos recientes indican que los cuerpos tanto de hombres como de mujeres fueron depositados en el Cenote Sagrado[7,23]. Investigaciones sistemáticas de conjuntos de sacrificios en toda la región maya han confirmado que tanto hombres como mujeres fueron sujetos a sacrificios rituales, pero dado que la mayoría de los individuos sacrificados en sitios del Clásico Maya son jóvenes, las distribuciones de sexo precisas no pueden determinarse únicamente mediante enfoques osteológicos convencionales[9,23]. Fuentes españolas del siglo XVI registran que estos niños se obtenían localmente mediante secuestro, compra e intercambio de regalos[19,24,25], aunque estudios isotópicos recientes sugieren que al menos algunos individuos dentro del Cenote Sagrado no eran locales y quizá eran originarios de lugares tan lejanos como Honduras o el centro de México[1]. Sin embargo, a pesar de más de un siglo de investigación, mucho sobre el sacrificio infantil y el uso ceremonial de elementos subterráneos como entierros rituales masivos en Chichén Itzá continúa siendo desconocido.

Para comprender mejor el origen y las relaciones biológicas de los niños sacrificados, así como su relación con los habitantes actuales de la región, utilizamos un enfoque combinado de bioarqueología y genómica para investigar 64 sub-adultos enterrados dentro de un chultún cerca del Cenote Sagrado (Fig. 1C) y compararlos con 68 habitantes mayas actuales del cercano pueblo de Tixcacaltuyub, así como con otros datos genéticos antiguos y contemporáneos disponibles de la región. La comunidad de Tixcacaltuyub ha colaborado con nuestro equipo de investigación durante muchos años, y sus perspectivas y preguntas fueron tomadas en cuenta para el desarrollo de este manuscrito (para más información, consulte Supplementary methods: Community engagement activities). Nuestro análisis, que comprende datos genéticos humanos antiguos, análisis de isótopos estables de carbono y

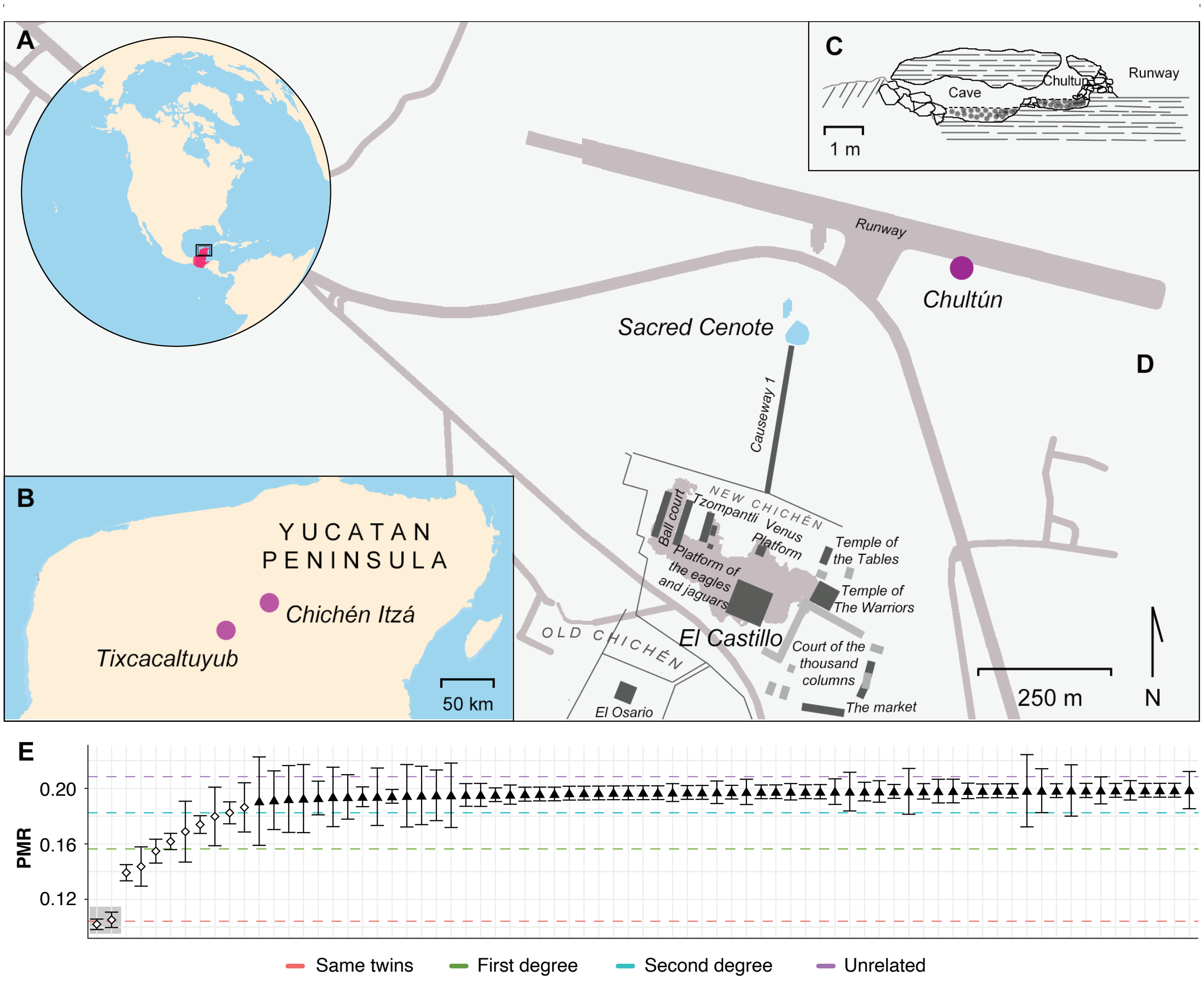

Figura 1: Contexto geográfico de los grupos analizados y parentesco biológico en el chultún. A. *Ubicación de la región maya en América.* ***B.*** *Ubicación geográfica de Chichén Itzá y Tixcacaltuyub en la Península de Yucatán.* ***C.*** *Estratigrafía del chultún y la cueva adyacente en la que se encontró el entierro (adaptado de [19]). El sombreado horizontal indica roca, mientras que el sombreado diagonal indica secciones no excavadas.* ***D.*** *Ubicación del chultún dentro del sitio arqueológico de Chichén Itzá y su relación con El Castillo (adaptado de [21]). Las carreteras modernas están marcadas en gris claro; el chultún linda con la pista de un aeropuerto.* ***E.*** *La tasa de desajuste genético por pares (PMR) para parejas de niños dentro del chultún identifica once pares de parientes cercanos (rombos huecos), incluidos dos pares de gemelos monocigóticos (resaltados en gris). Una PMR general baja para individuos no relacionados (triángulos negros) confirma una baja diversidad genética en la población; En el gráfico solo se visualizan los pares con un PMR <0,20. Consulte la Fig. S2 para ver anotaciones individuales. Las figuras 1A y 1B están adaptadas de Google Earth (Google Inc. 2010; ver. 5.2.1.1588).*

nitrógeno del colágeno óseo, y datación por radiocarbono (Tabla S1), revela que todos los sub-adultos del chultún eran varones y que en el entierro masivo estaban presentes parientes cercanos, incluidos dos pares de gemelos monocigóticos. El análisis de isótopos estables indica que los niños relacionados consumían dietas más similares, y que en general la dieta de los niños de Chichén Itzá era comparable a la de otras poblaciones del período Clásico en las Tierras Bajas Mayas (Tabla S2; Fig. S4). La comparación genética con otros pueblos antiguos y actuales muestra una continuidad genética a largo plazo en la región maya, pero indica cambios en la frecuencia alélica en genes de inmunidad en el locus HLA de clase II, específicamente un aumento en los alelos HLA-DR4 que proporcionan una mayor resistencia a la infección por *Salmonella enterica*, el agente causal de una fiebre entérica previamente identificada en una fosa común de la época colonial en Oaxaca, al sur de México, que se asoció con la pandemia de *cocoliztli* de 1545 [26].

Los tres hallazgos principales de este estudio son: i) la continuidad genética entre los mayas actuales y antiguos, ii) el sexo cromosómico de todos los

individuos sacrificados analizados es masculino, lo que desafía las interpretaciones clásicas de los sacrificios de niños en la región, y iii) la huella genómica dejada por los patógenos introducidos durante la época colonial, aumentando la frecuencia de variantes genéticas que brindaban protección contra ellos.

Resultados

Generación de datos sobre genoma y genes inmunes

Se recolectaron, procesaron y analizaron muestras de huesos de individuos antiguos encontrados en el entierro del chultún en Chichén Itzá (en adelante, YCH) de acuerdo con protocolos diseñados para el análisis de ADN antiguo (aDNA) en instalaciones especializadas[27]. Dado que no todos los elementos esqueléticos pudieron asignarse inequívocamente a un solo individuo, solo se recolectaron los huesos petrosos izquierdos para evitar muestrear a los individuos más de una vez. El análisis de carbono-14 (^{14}C) (n=26) mostró que el chultún estuvo en uso durante al menos 500 años desde el florecimiento inicial del sitio a principios del siglo VII d.C., pasando por su apogeo durante el siglo X AD. hasta mediados del siglo XII (Fig.S1 y Tabla S1). Se logró recuperar ADN antiguo de los 64 individuos de YCH. Además, se recolectó ADN a partir de muestras de sangre obtenidas de 68 habitantes modernos del pueblo de Tixcacaltuyub, Yucatán, México (en adelante TIX), con el fin de comparar a los habitantes modernos y antiguos de la región. El material genómico extraído se incorporó a bibliotecas genómicas tratadas con uracilo ADN glicosilasa (UDG) (para YCH) o no tratadas con UDG (para TIX) y se secuenció a una profundidad de ~5-11 millones de lecturas para evaluar la preservación y autenticidad del ADN. Después, construimos 11 bibliotecas de hebras simples tratadas con UDG para aumentar aún más la cantidad de datos analizables para un subconjunto de individuos YCH (ver *Supplementary methods: Preparation of single stranded libraries*). Se realizó una evaluación de control de calidad (QC) para garantizar niveles de contaminación aceptables (<5%) con dos métodos implementados como parte del pipeline nf-core/Eager[28]. Todos los individuos TIX y 56 YCH proporcionaron suficiente ADN humano para el análisis (>0,1 % de ADN humano), y después de un procedimiento de reacondicionamiento, enriquecimos aún más estas bibliotecas de ADN para obtener un panel de 1.2 millones de polimorfismos de un solo nucleótido informativos sobre la ascendencia ("1240K"), el genoma mitocondrial (ADNmt) y un panel de genes inmunitarios[19 – 31]. Después de secuenciar las bibliotecas genómicas enriquecidas, obtuvimos ~40 millones de lecturas/biblioteca; a partir de estos datos, realizamos un control de calidad adicional y llevamos a cabo el análisis de genética de poblaciones y tipificación de HLA.

Parentesco genético y gemelos en el Chultún

Las comparaciones de cobertura de los SNP de los cromosomas X y Y asignaron a todos los individuos subadultos de YCH como genéticamente masculinos y confirmaron el sexo registrado para todos los participantes de TIX[32]. Los análisis de tasa de discrepancia por pares[33] (Fig. 1E y S2) para YCH respaldan la presencia de dos pares de gemelos idénticos (YCH018-YCH019, YCH033-YCH054) y otros nueve pares de parientes cercanos (YCH016-YCH-17, YCH017-YCH018, YCH017 -YCH019, YCH034-YCH041, YCH036-YCH038, YCH042-YCH049, YCH047-YCH057, YCH049-YCH057, YCH059-YCH060). En general, el 25% (n=16) de los niños analizados en el entierro ritual están estrechamente relacionados con otro niño dentro del chultún.

Patrones isotópicos en la dieta de los niños

Las mediciones de $\delta^{13}C$ y $\delta^{15}N$ del colágeno óseo (Tabla S1 y Fig. S3; ver también Supplementary methods: Stable isotopes analyses) proporcionaron valores de $\delta^{13}C$ entre -13,9 y -7,6 ‰ (media y DE = -9,9 ± 1,5 ‰) y valores de $\delta^{15}N$ que oscilan de 5,9 a 14,0‰ (media y DE = 9,7±1,5‰). En general, estos valores son similares a los reportados en otros sitios de las tierras bajas mayas del periodo Clásico (Tabla S2 y Fig. S4), y son consistentes con investigaciones arqueológicas previas de evidencia dietética en la región, así como con observaciones preliminares de restos arqueológicos en la Península de Yucatán[34,35] y otros sitios del Clásico Maya[36–39]. Es posible que las dietas de algunos individuos con valores más altos de $\delta^{15}N$ y más bajos de $\delta^{13}C$ (por ejemplo, YCH004, YCH008, YCH023, YCH039, YCH047, and YCH061) hayan incluido recursos acuáticos[34], pero también podrían indicar otras variaciones dietéticas relacionadas con el estatus social[40] o resultar de la influencia de la lactancia materna[41]. Sin información contextual adicional o datos isotópicos de referencia locales de la fauna asociada, sigue siendo un desafío determinar con mayor precisión las dietas individuales. Sin embargo, la comparación de nuestros

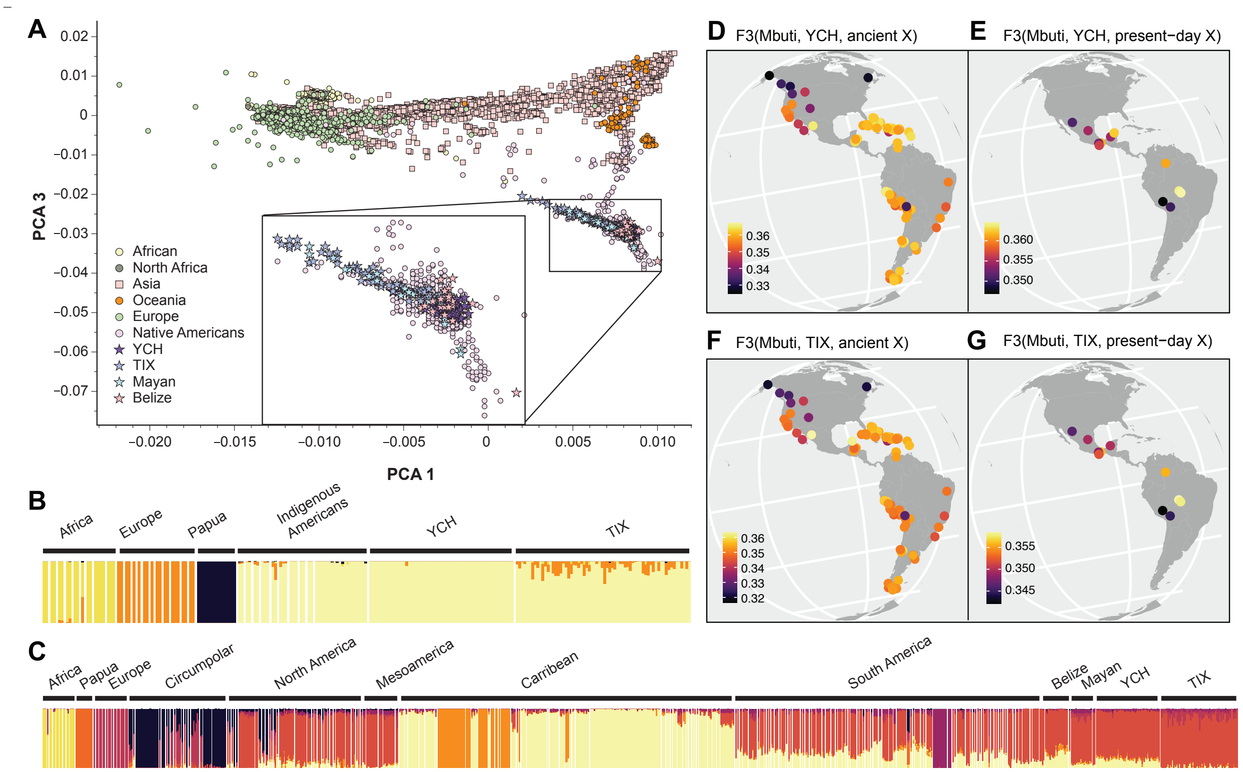

*Figura 2: **Similitudes genéticas entre Chichén Itzá, Tixcacaltuyub y los grupos indígenas americanos actuales y antiguos. A.** Análisis de Componentes Principales (PCA) que muestra los individuos de la antigua Chichén Itzá (YCH) y el actual Tixcacaltuyub (TIX) en un gráfico de PCA mundial. **B.** Análisis de mezclas que muestran el agrupamiento de k=4 para YCH, TIX y diferentes fuentes genéticas continentales, y **C.** el agrupamiento de las muestras recién producidas en k=8 (errores de validación cruzada más bajos), con una lista más completa de Indígenas. poblaciones americanas. Estadísticas del grupo externo F3 en la forma f3(Mbuti; YCH, X) donde **D.** X son grupos actuales de América y **E.** grupos e individuos antiguos publicados. Las similitudes altas se indican con colores más cálidos, las similitudes bajas con colores más oscuros. Estadísticas del grupo externo F3 en la forma f3(Mbuti; TIX, X) donde **F.** X son grupos actuales de América y **G.** publicaron grupos e individuos antiguos. Las similitudes altas se indican con colores más cálidos, las similitudes bajas con colores más oscuros. Puede encontrar una lista de las poblaciones/individuos utilizados en estos análisis en la sección Material complementario: poblaciones de origen para los análisis de genética de poblaciones.*

datos con los resultados publicados (más de 450 individuos estudiados de los períodos Clásico Tardío y Clásico Terminal)[42–44] y un $\delta^{15}N$ notablemente variable sugieren que los 54 individuos chultún consumieron cantidades significativas de recursos terrestres C_4, así como probablemente cantidades variables de proteínas C_3 terrestres y recursos de agua dulce y/o marinos. Esto es consistente con investigaciones arqueológicas previas centradas en dietas documentadas del Clásico Maya [35,36,38,39,45,46]. Los valores isotópicos de individuos emparentados son cercanos entre sí, lo que sugiere una similitud dietética (Fig. S5).

Continuidad genética en la región Maya

Se realizó un análisis de componentes principales (PCA) basado en poblaciones mundiales e individuos actuales de las Américas[52] (Fig. 2). Como se esperaba para las poblaciones mesoamericanas, los individuos YCH se agrupan estrechamente en un PCA mundial con poblaciones indígenas americanas sin contribución genética de poblaciones no americanas. Algunos individuos de TIX se han desplazado hacia los europeos, lo que sugiere un mestizaje genético (Fig. 2). Cuando tanto YCH como TIX se proyectan sobre poblaciones indígenas americanas actuales de América del Norte, Central y del Sur[47], se agrupan con los mayas actuales (Fig. 2b). Los análisis de mestizaje no supervisado utilizando un subconjunto de poblaciones de África, Europa, Oceanía y América (Fig. 2D y 2E) no mostraron signos de mestizaje en los individuos YCH y contribuciones bajas de ascendencia europea y africana para los individuos TIX, con algunos de ellos (n=18) sin indicaciones de contribuciones genéticas americanas no indígenas. Es interesante señalar que un componente genético maximizado en poblaciones antiguas de la región del Caribe está presente tanto en los antiguos

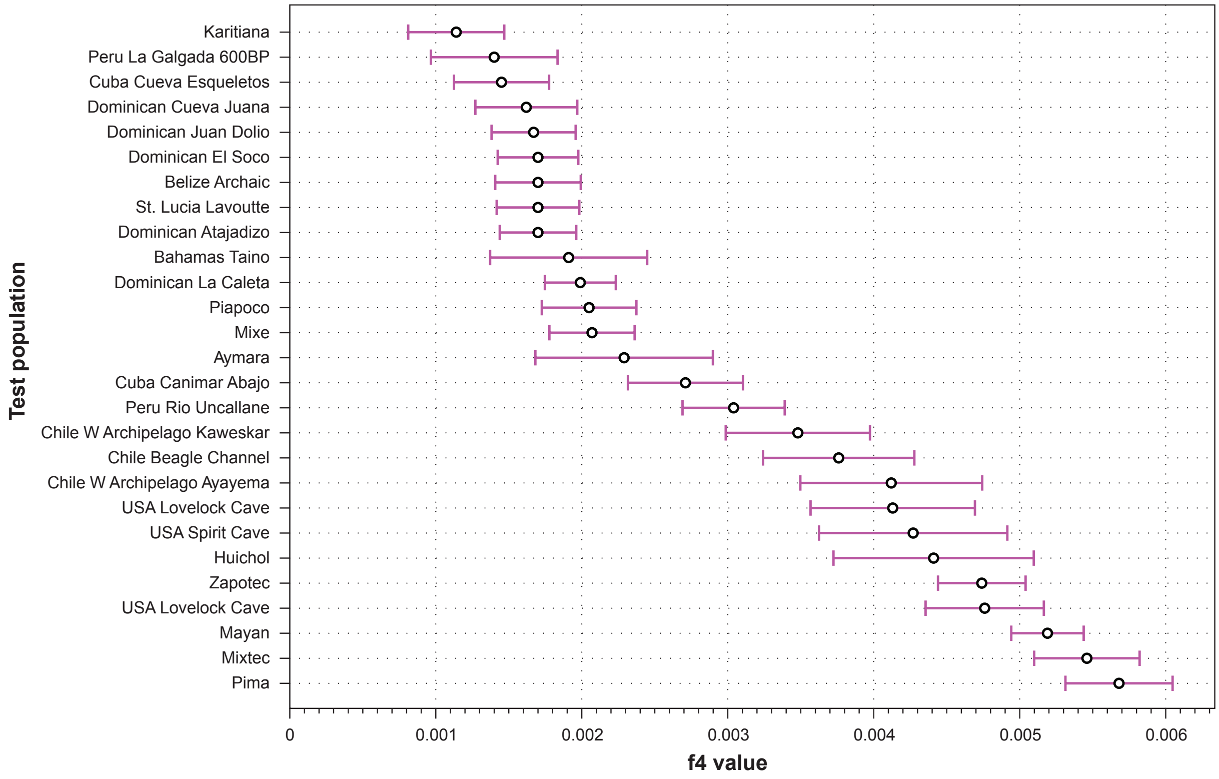

Figura 3: Prueba F4 de la forma f4(Mbuti, YCH; prueba, TIX). *Los valores se trazan (círculo) con sus correspondientes barras de error (en morado). Para todas las poblaciones analizadas, el valor de la prueba es positivo, lo que indica que YCH y TIX compartieron más alelos entre ellos que cualquiera de las poblaciones nativas americanas analizadas.*

mayas de Belice[48,49] como en YCH, pero está casi ausente en la composición genética de los mayas actuales[50] y de TIX. El mestizaje con otras poblaciones de Mesoamérica (donde el componente aún no ha sido detectado) o la deriva genética podrían explicar la desaparición de este componente en los mayas actuales.

Para evaluar las afinidades genéticas con poblaciones americanas actuales y antiguas, calculamos un estadístico–F_3 de grupo externo de la forma f_3 (grupo externo, objetivo, prueba), utilizando a Mbuti del África subsahariana[50,51] como grupo externo, un panel de poblaciones indígenas americanas como objetivo, y ya sea TIX o YCH como prueba. Las similitudes genéticas más altas detectadas tanto para YCH como para TIX (Fig. 2) incluyeron grupos de América Central y del Sur. Los individuos TIX comparten la mayor deriva genética con los individuos antiguos de Chichén Itzá (Fig. 2). Entre las poblaciones antiguas analizadas, un individuo de 9.300 años de antigüedad del sitio Mayahak Cab Pek en las Montañas Mayas en el sur de Belice[49], y otros individuos analizados, también de Belice pero de un contexto más reciente[48], son genéticamente más similares a los individuos antiguos de Chichén. Itzá, lo que sugiere una continuidad genética a largo plazo en la región Maya. Probamos si YCH y TIX están más cerca entre sí que de otras poblaciones indígenas americanas utilizando un estadístico–F f_4 (Mbuti, TIX, objetivo, YCH), utilizando un subconjunto de las poblaciones indígenas americanas con mayor puntuación F_3. Nuestros resultados sugieren que algunos miembros de las poblaciones indígenas americanas objetivo que probamos están más estrechamente relacionados con TIX (Fig. 3). Esto puede sugerir que, aunque los individuos TIX están genéticamente relacionados con los antiguos habitantes de Chichén Itzá, la población genéticamente más cercana a Chichén Itzá puede que ya no exista, o aún no haya sido muestreada.

Luego aplicamos *qpWave*[47], un enfoque de modelado de composición de ascendencia que evalúa la probabilidad de que dos individuos/grupos deriven de la misma fuente de ascendencia en relación con un conjunto determinado de referencias de población. Los valores *p* resultantes

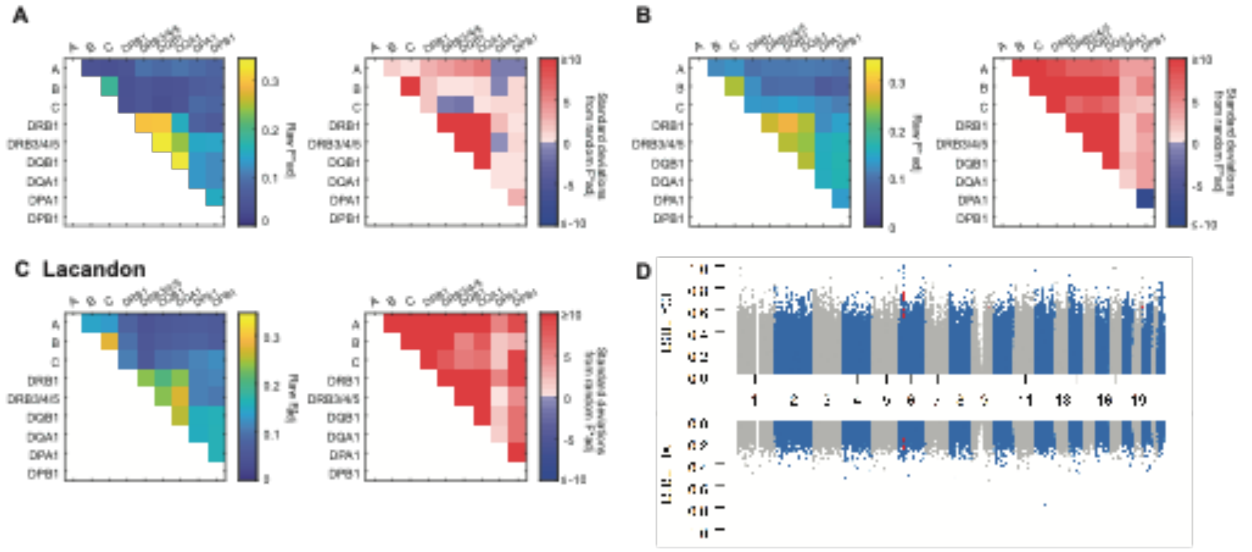

Figura 4: Evidencia de selección en la región HLA. *Patrón de asociaciones no superpuestas para los genes en la región HLA medido por* F^*_{adj} *(mapas de calor a la izquierda) y la diferencia entre los niveles observados de asociaciones alélicas no superpuestas y aleatorias, en unidades de desviación estándar (mapas de calor a la derecha), para* ***A.*** *Mayas antiguos de Chichén Itzá (YCH),* ***B.*** *Mayas actuales de Tixcacaltuyub (TIX) y* ***C.*** *Mayas lacandones actuales de Chiapas (SE de México)*[77]*.* ***D.*** *Gráficos de Manhattan de valores de longitud de rama específica del locus (LSBL) de todo el genoma para YCH (panel superior) y TIX (panel inferior). Los loci HLA se muestran en rojo. Se indican el 0,1% (línea roja) y el 0,5% (línea azul) superiores de todas las estadísticas.*

(kaqchikel: 0,08, "maya" [de Ref. [52]): 0,755] sugieren que los YCH y los grupos mayas actuales comparten la misma ascendencia. Se puede representar a TIX como un mestizaje de YCH, españoles y yoruba; el modelo de trabajo (p=0,11) sugiere una composición de 92% de componente indígena americano, 7% de contribución genética europea y 0,03% de ascendencia africana. Utilizando una prueba de máxima verosimilitud para la continuidad genética[53], podríamos formalmente probar que TIX es una población genéticamente descendiente directa de YCH (deriva genética promedio entre individuos: ~0,5, valor p ~1,0 para cada individuo TIX). Para 53 individuos YCH y todos los 68 individuos TIX, se pudo determinar el haplogrupo de ADN mitocondrial (ADNmt), y la frecuencia de los haplogrupos (A, B, C, D) es casi idéntica entre ambos grupos. Sin embargo, a partir de los haplotipos, que representan un mayor nivel de resolución genética, está claro que la diversidad de ADNmt es mayor en YCH que en TIX (Fig. S6). Los haplogrupos de ADNmt y los linajes de haplotipos corresponden a los reportados previamente para los mayas antiguos[54,55] y actuales[56,57]. Todos los haplotipos del cromosoma Y (Y-Chr) (Fig. S6) recuperados de los individuos de Chichén Itzá (n = 51) son parte de la familia Q (prevalente entre los indígenas americanos), mientras que más de la mitad de los cromosomas Y de TIX (n=19) son europeos (47,37%) y del Medio Oriente (5,26%), lo que sugiere un fuerte sesgo sexual en el proceso de mestizaje durante y después del período colonial, como se ha descrito previamente en otras poblaciones latinoamericanas[58–61].

Genómica de las vías metabólicas en los mayas

Utilizando los datos de SNP generados para ambas poblaciones, calculamos longitudes de ramas específicas del locus (LSBL)[62,63] para evaluar la selección a nivel genómico tanto para YCH como para TIX. Realizamos dos comparaciones de LSBL: primero, YCH vs. íberos de España (del proyecto 1000 Genomas[64]) y Han de China (del proyecto 1000 Genomas); y segundo, TIX vs. íberos y YCH para realizar pruebas de selección separada de YCH e íberos. Entre el top 0,5% de los SNPs anotados, encontramos 29 genes implicados en el metabolismo de los lípidos para YCH, incluidos los genes de desaturasas de ácidos grasos (FAD, por sus siglas en inglés) previamente reportados[65], y encontramos 20 genes para TIX, incluidos la dioxigenasa dependiente de alfa-cetoglutarato (FTO) FTO y el Factor de transcripción 7 tipo 2 (TCF7L2), los cuales se han asociado con rasgos metabólicos en poblaciones latinoamericanas, y particularmente mayas [66–68] (Tablas S3-S5). Los SNP de ciertos genes, como los

que pertenecen a la familia de la adenilato ciclasa (ADCY), también se encontraban entre el 0,5 % superior para TIX, lo que es consistente con reportes previos[69], pero no entre YCH, lo que puede indicar una selección diferencial antes y después del período colonial. Luego, buscamos vías de ontología génica (GO) enriquecidas utilizando GoWinda[70]. Si bien ambos grupos muestran términos GO enriquecidos (p <0,05 ajustado) asociados con vías metabólicas (Tablas S6 y S7), YCH muestra un aumento en los procesos biológicos asociados con la fertilidad (como la ovogénesis, la vía de señalización mediada por hormonas esteroides, el ciclo de ovulación y el ciclo estral, entre otros), mientras que los términos de vías metabólicas del colesterol y los lípidos (como la regulación negativa del proceso biosintético de los lípidos, la homeostasis del colesterol y la homeostasis de los esteroles) aparecen más prominentemente en TIX.

Genes HLA apuntan a un cambio en inmunidad

Con respecto a los genes implicados en la inmunidad, pudimos detectar quince y siete SNPs de la región del antígeno leucocitario humano (HLA) entre el top 0.5% de los SNPs anotados para YCH y TIX, respectivamente, mostrando signos de selección positiva (Fig. 4). Ninguno de los SNPs fue compartido por YCH y TIX, y ninguno se encontró bajo selección en un estudio previo en indígenas americanos antiguos y modernos de la costa noroeste de América del Norte[71]. Los SNPs encontrados en los individuos YCH están ubicados en los genes *HLA-B, -DRB1, -DQA1, -DQA2, -S, -X, HLA-DOA* y *-DQB1* o en regiones cercanas, mientras que los SNPs de TIX se encuentran en o alrededor de los genes *HLA-C, -DQA1, -DQA2* y *-DQB1* (Tabla S8). Utilizando un modelo multilocus de coevolución huésped-patógeno con inmunidad adaptativa específica de alelo, se ha demostrado que si la selección de un patógeno mantiene asociaciones entre los loci de reconocimiento del huésped (como en el sistema HLA), los alelos en esos loci no solo estar en desequilibrio de ligamiento (LD), sino que también podrían exhibir asociaciones no superpuestas[72]. Por esa razón, analizamos el patrón de asociaciones no superpuestas[72,73] (Fig. 4, S7 y S8) para evaluar la selección impulsada por patógenos en las asociaciones HLA en YCH, TIX y los mayas lacandones previamente analizados de las tierras altas de Chiapas en el sureste de México[74]. Medimos la métrica f_{adj} (un parámetro utilizado para clasificar la fuerza de las asociaciones no superpuestas; Supplementary Methods: Non-overlapping HLA associations)[72] entre diferentes pares de loci HLA. También medimos la diferencia entre nuestros niveles observados de no superposición y los que se observarían para asociaciones alélicas aleatorias, en unidades de desviaciones estándar.

Al comparar con los datos antiguos, los datos mayas actuales parecen tener un mayor grado de no superposición (Fig. 4A-4C), lo que sugeriría que las poblaciones modernas analizadas han experimentado selección en el locus HLA, potencialmente debido a la exposición a patógenos. Cuando comparamos las frecuencias alélicas de HLA (Tablas S9-S19) de YCH y TIX, detectamos un cambio estadísticamente significativo (p_{corr} <0,05) en ocho alelos: tres alelos de clase I de HLA y cinco alelos de clase II de HLA, después de corregir por comparaciones múltiples. Se observa una disminución significativa en la frecuencia de los alelos HLA-B*40:02 (0.2447 vs. 0.0821; p_{corr}=0.0296), HLA-DQA1*03:03 (0.1277 vs. 0.0224; p_{corr}<0.0218), y HLA-DQB1*04:02 (0.1809 vs. 0.0299; p_{corr}=0.0249), mientras que HLA-A*68:03 (0.0532 vs. 0.2687; p_{corr}=0.0003), HLA-B*39:05 (0.0532 vs. 0.2687; p_{corr}=0.0437), HLA-C*07:02 (0.2021 vs. 0.3955; p_{corr}=0.0369), HLA-DQB1*03:02 (0.4894 vs. 0.7015; p_{corr}=0.0177), y HLA-DRB1*04:07 (0.2340 vs. 0.4627; p_{corr}=0.0114) aumentaron en frecuencia en TIX en comparación con YCH. Para TIX, encontramos que el 88% de los haplotipos HLA se han reportado previamente en poblaciones indígenas americanas, el 10% de los cuales son probablemente de origen europeo y el 2% representan haplotipos africanos. Todos los haplotipos de HLA de YCH son consistentes con los encontrados en poblaciones indígenas americanas, particularmente entre los mayas [74–76].

La región HLA de clase II ha sido previamente implicada en eventos de selección antes y después del contacto con los europeos en el siglo XVI en las Américas[71], y en la resistencia a la infección por *Salmonella enterica*[77–79]. Por lo tanto, nos interesó probar cómo reaccionarían los alelos para los cuales hay un cambio significativo a los péptidos derivados de *Salmonella*. Para hacerlo, seleccionamos 18 proteínas de *Salmonella* para las cuales hay evidencia de inmunogenicidad en humanos y presentamos péptidos *in silico* derivados de ellas que se encuentran tanto en YCH como en TIX (ver Tabla S20 e Supplementary information: In silico binding prediction assays) utilizando el método de predicción de unión

NetMHCIIPan[80] implementado en IEDB Analysis Resource (http://tools.iedb.org/mhcii). Una unión fuerte significa que es más probable que el péptido unido inicie una respuesta inmune contra ese péptido, mientras que las uniones más débiles no tienen tanto éxito en provocar una respuesta inmune. Las mejores uniones fuertes de HLA-DR fueron HLA-DRB1*14:02, HLA-DRB1*04:07, HLA-DRB1*16:02, y HLA-DRB1*04:17; mientras que las mejores uniones fuertes para HLA-DQ fueron HLA-DQA1*05:01/DQB1*03:01, HLA-DQA1*05:05/DQB1*03:03 y HLA-DQA1*03:01/DQB1*03:03. Las moléculas HLA con el menor número de péptidos fuertemente unidos HLA-DRB1*14:06, HLA-DRB1*04:04, HLA-DRB1*04:05 para HLA-DR, y HLA-DQA1*03:03/DQB1*04:02, HLA-DQA1*04:01/DQB1*04:02 y HLA-DQA1*03:01/DQB1*03:02 para HLA-DQ. En general, el menor número de péptidos, ya sea fuertemente o débilmente presentados, por las moléculas de clase II de HLA corresponde a los alelos HLA-DR DRB1*08:02, DRB1*04:04 y DRB1*14:06, y las mismas moléculas HLA-DQ enlistadas previamente. A grandes rasgos, las frecuencias de HLA-DRB1*04:07 (unión fuerte) y HLA-DQB1*03:02 (unión débil, en LD con HLA-DRB1*04:07) aumentan en TIX, mientras que HLA-DQA1* 03:03 y HLA-DQB1*04:02 disminuyen en TIX y presentan uniones débiles.

Discusión

La arqueogenética ofrece una oportunidad para investigar aspectos de las prácticas rituales mayas del pasado, el parentesco biológico, el sexo y la historia genética que pueden ser difíciles de inferir utilizando métodos arqueológicos más convencionales. Aquí investigamos los restos de 64 individuos sub-adultos que fueron enterrados ritualmente durante un período de 500 años en un chultún del período Clásico ubicado cerca del Cenote Sagrado en la antigua ciudad maya de Chichén Itzá. En contraste con los restos humanos en el Cenote Sagrado, encontramos que todos los sub-adultos en el chultún eran varones, lo que demuestra una fuerte preferencia por el sacrificio ritual de niños varones en este contexto. El análisis genético también reveló la presencia de individuos emparentados dentro del chultún, incluidos dos pares de gemelos monocigóticos y otros nueve pares de parientes cercanos. Dado que los gemelos idénticos ocurren espontáneamente en sólo el 0,4% de la población general[81], la presencia de dos pares de gemelos idénticos en el chultún es mucho mayor de lo que se esperaría por casualidad. En general, el 25% de los niños tenían un pariente cercano dentro del grupo, lo que sugiere que los niños sacrificados pueden haber sido seleccionados específicamente por su parentesco biológico cercano. Además, esto puede subestimar el número real de parientes presentes en el chultún, ya que sólo 64 de los 106 individuos estimados en el chultún tenían una porción petrosa preservada del hueso temporal izquierdo disponible para análisis. El hallazgo adicional de que los niños estrechamente relacionados en cada grupo parecen haber consumido una dieta similar y haber muerto a una edad similar sugiere que fueron sacrificados durante el mismo evento ritual como un sacrificio de pareja o de gemelos.

Los gemelos son especialmente auspiciosos dentro de la mitología maya, y el sacrificio de gemelos es un tema central en el sagrado Libro del Consejo maya K'iche', el Popol Vuh[82], un libro cuyos antecedentes se remontan al período Preclásico maya[83]. En el Popol Vuh, los gemelos Hun Hunapu y Vucub Hunahpu descienden al inframundo y son sacrificados por los dioses tras su derrota en un juego de pelota. Luego, la cabeza de Hun Hunapu es colgada en un árbol de calabaza, donde fecunda a una doncella que da a luz a un segundo par de gemelos, Hunapu e Ixbalanqué. Estos gemelos, conocidos como los Héroes Gemelos, luego vengan a su padre y a su tío sometiéndose a repetidos ciclos de sacrificio y resurrección para burlar a los dioses del inframundo. Los Héroes Gemelos y sus aventuras están ampliamente representados en el arte maya del Clásico[5], y dado que las estructuras subterráneas eran vistas como entradas al inframundo, los sacrificios de gemelos y parientes dentro del chultún en Chichén Itzá pueden evocar rituales relacionados con los Héroes Gemelos.

Al comparar los sub-adultos del chultún con otras poblaciones antiguas y actuales de la región maya, encontramos evidencia de continuidad genética a largo plazo, lo que también sugiere que los niños y pares de hermanos sacrificados en Chichén Itzá se obtuvieron de comunidades mayas antiguas cercanas. Entre los individuos actuales en TIX, detectamos evidencia de mestizaje europeo y africano desde el período de Contacto. Aunque las contribuciones de ascendencia de fuentes no indígenas americanas son bajas a nivel genómico, son fuertemente asimétricas con respecto a

los marcadores uniparentales. Mientras que todos los haplogrupos de ADNmt son indígenas americanos, más de la mitad de los Y-Chr en TIX son no indígenas americanos (en su mayoría de origen europeo y del Medio Oriente/Mediterráneo, según reportes previos[8,59]) lo que indica que se produjo un fuerte sesgo masculino hacia las ascendencias americanas no indígenas durante el período de contacto.

La similitud genética entre los grupos mayas determinada en los análisis genéticos poblacionales (perfiles de mestizaje, F_3 y F_4, y prueba de continuidad genética) nos permitió explorar cambios en las regiones genómicas que codifican variantes funcionales para probar la selección en los mayas antiguos (YCH) y actuales (TIX). Nuestros hallazgos respaldan hipótesis previas de que tanto el metabolismo de los lípidos[84,85] como la fertilidad[84] son rasgos que se han seleccionado recientemente en los indígenas americanos, probablemente debido a los fuertes cuellos de botella y restricciones calóricas que experimentaron estas poblaciones durante los primeros períodos coloniales y de asentamiento[84,86]. La desviación estándar observada de los valores de δ^{15}N (SD = 1.5) obtenidos de los individuos analizados en Chichén Itzá es la más alta de todos los sitios mayas del Clásico Tardío y del Clásico Terminal analizados hasta la fecha (ver Fig. S3, S4 y Tabla S2). El panorama general de la reconstrucción de la paleodieta en la región maya revela el consumo de cantidades significativas de alimentos C_4, muy probablemente maíz, pero con variaciones geográficas reflejadas en diferencias microambientales en los alimentos disponibles y en la variabilidad de las redes comerciales[87,88]. Para explicar esta variabilidad, es posible que una parte considerable de los individuos sacrificados en Chichén Itzá fueran mayas no locales que consumían dietas ligeramente diferentes. Alternativamente, investigaciones previas indican que los patrones dietéticos de la élite maya del Clásico tienden a mostrar una mayor variabilidad que los de la población general a lo largo del tiempo[88–90]. Esta variabilidad también se refleja en las desviaciones estándar de δ^{13}C y δ^{15}N observadas en otros lugares, como Altun Ha o Baking Pot (ver Fig. S4 y Tabla S2). Por lo tanto, las diferencias en la ingesta de proteínas observadas en los individuos de Chichén Itzá estudiados también podrían indicar variaciones en el estatus social. Tampoco se puede descartar que parte de la variabilidad observada en δ^{15}N sea resultado de la influencia de la lactancia materna[91], ya que los restos muestreados provienen de individuos que se estima tienen entre 3 y 6 años de edad. Por lo tanto, se debe tener precaución al interpretar variaciones dietéticas específicas en ausencia de otra información contextual. Curiosamente, nuestros datos indican que aquellos individuos cuyo ADN reveló una relación genética cercana tenían valores de δ^{13}C y δ^{15}N más similares, lo que sugiere que pueden haber tenido un origen compartido y haber sido criados en una red familiar extensa que brindó cuidados y alimentación similares (Fig. S3, Figura S5; Tabla S1).

Es importante destacar que los valores de deriva genética obtenidos de la prueba de continuidad genética significan que los ancestros de TIX sufrieron una grave disminución de la población en algún momento de los últimos ~1000 años. A lo largo del siglo XVI, se ha demostrado que las guerras, las hambrunas y las epidemias provocaron una disminución de la población, potencialmente tan alta como el 90%, de 10 a 20 millones de indígenas que vivían en México en el momento del contacto europeo a solo 2 millones de personas a finales del siglo XVI[92–97]. Enfermedades infecciosas, como viruela, sarampión, paperas, influenza, *tabardillo* o *matlalzahuatl* (tifus), fiebre tifoidea y entérica, rubéola, tos ferina, *garrotillo* (difteria grave), disentería endémica, *fiebres terciarias* (malaria) y sífilis[26,98–102] se discute que causaron brotes a gran escala en la Mesoamérica colonial, contribuyendo significativamente a la disminución de la población y posiblemente causando selección en loci relacionados con el sistema inmunológico. Se ha informado anteriormente que la región del HLA clase II sufrió eventos de selección durante el período colonial en las Américas[71]. Resultó de interés encontrar que tres de los alelos cuyas frecuencias cambian cuando comparamos YCH con TIX son parte de la región del HLA clase II, un hallazgo que está respaldado por los SNP encontrados en los análisis de LSBL. Uno de esos alelos (HLA-DRB1*04:07) pertenece a un grupo de alelos (HLA-DR4) que se ha informado previamente que está asociado con la resistencia a la fiebre entérica causada por la subespecie de *Salmonella enterica* en América del Sur[79] y el este de Asia[78]. Recientemente, un estudio arqueogenético identificó la presencia de S. *enterica* sp. Paratyphi C en un entierro masivo asociado con la pandemia de cocoliztli de 1545 d. C., lo que sugiere que fue al menos uno de los agentes causantes de esta pandemia[26], que tuvo la mayor mortalidad de todas las

epidemias registradas de la era colonial. Además, el análisis genómico de cepas antiguas de S. *enterica* respalda firmemente una introducción en América durante el siglo XVI[103].

El aumento observado del grupo de alelos HLA-DR4 entre los mayas actuales y los mexicanos en general[104] es consistente con la selección causada por un evento epidémico y la posterior exposición sostenida al patógeno. Un examen más detallado de las asociaciones no superpuestas entre los alelos HLA también coincide con que las poblaciones mayas actuales se han sometido a una selección de patógenos, lo que ha llevado a que sus asociaciones HLA se superpongan menos que YCH. Los ensayos de predicción de unión *in-silico* también apuntan a HLA-DRB1*04:07 como una unión fuerte para los péptidos derivados de *Salmonella*, mientras que HLA-DQB1*04:02 y HLA-DQB1*03:03, significativamente reducidos en la población maya actual, presentan una unión más débil para los mismos péptidos. Consideradas en conjunto, cada línea de evidencia apunta a eventos de selección que ocurren dentro de la región HLA en respuesta a un evento epidémico durante el período colonial. Tal selección sería esperable dada la intensidad de la pandemia de cocoliztli de 1545 y el elevado número de eventos epidémicos documentados en la región maya desde principios del siglo XVI[105-107]. Aunque no se puede descartar que los haplotipos con combinaciones de alelos no superpuestos hayan sobrevivido por casualidad a un cuello de botella no relacionado con una enfermedad, tal escenario probablemente resultaría en una mayor frecuencia de alelos ya presentes en la población antigua, que no es lo que observamos.

Nuestro estudio revela un retrato íntimo de los niños mayas del Clásico Tardío y Terminal en Chichén Itzá, sugiriendo que el legado genómico de los antiguos habitantes de este sitio todavía está presente en las comunidades que habitan la región que rodea esta antigua ciudad. El descubrimiento de dos pares de gemelos idénticos, así como de otros parientes cercanos, en un entierro ritual masivo de niños varones sugiere que los niños masculinos pueden haber sido seleccionados para el sacrificio debido a su parentesco biológico y la importancia de los gemelos en la mitología maya. Mostramos que, a nivel de todo el genoma, los mayas actuales de Tixcacaltuyub exhiben continuidad genética con los antiguos mayas que alguna vez habitaron Chichén Itzá, y demostramos a través de múltiples líneas de evidencia la participación de la región HLA en eventos de selección impulsados por patógenos probablemente causados por enfermedades infecciosas introducidas en las Américas por los europeos durante el período colonial.

Referencias:

1. Price, T. D., Tiesler, V. & Freiwald, C. Place of origin of the sacrificial victims in the sacred Cenote, Chichén Itzá, Mexico. *Am J Phys Anthropol* **170**, 98–115 (2019).
2. Kristan-Graham, C. *et al. Twin Tollans: Chichén Itzá, Tula, and the Epiclassic to Early Postclassic Mesoamerican World*. (Dumbarton Oaks, 2007).
3. Beck, L. A. & Sievert, A. K. Mortuary pathways leading to the Cenote at Chichén Itzá. in *Interacting with the Dead: Perspectives on Mortuary Archaeology for the New Millennium* (eds. Rakita, G., JE, B., LA, B. & Williams, S.) 290–304 (University Press of Florida, 2005).
4. Márquez Morfín, L. & Schmidt, P. Osario infantil en un chultún en Chichén Itzá. in *Investigaciones recientes en el área maya. Vol. II. Memorias de la XVII Mesa Redonda (1981)* (ed. Sociedad Mexicana de Antropología) 89–104 (Sociedad Mexicana de Antropología; XVII Mesa Redonda, 1984).
5. Coe, M. D. The Hero Twins: Myth and image. in *The Maya Vase Book: A Corpus of Rollout Photographs of Maya Vases* (eds. Kerr, B. & Kerr, J.) 161–184 (Kerr Associates, 1989).
6. Cobos, R. & Winemiller, T. L. The late and terminal classic-period causeway systems of Chichen Itza, Yucatan, Mexico. *Ancient Mesoamerica* **12**, 283–291 (2001).
7. Tiesler, V. What can the bones really tell us? The study of human skeletal remains from cenotes. in *Stone houses and earth lords: Maya religion in the cave context* (eds. Prufer, K. M. & Brady, J. E.) 341–364 (The University Press of Colorado, 2005).
8. Miller, V. E. Skeletons, skulls, and bones in the art of Chichén Itzá. in *New Perspectives on Human Sacrifice and Ritual Body Treatments in Ancient Maya Society* (eds. Tiesler, V. & Cucina, A.) 165–189 (Springer New York, 2007). doi:10.1007/978-0-387-48871-4_7.
9. Graña-Behrens, D., Prager, C. & Wagner, E. The hieroglyphic inscription of the 'High Priest's Grave' at Chichén Itzá, Yucatán, Mexico. *Mexicon* **21**, 61–66 (1999).
10. Dahlin, B. H. Climate change and the end of the Classic period in Yucatán. Resolving a paradox. *Ancient Mesoamerica* **13**, 327–340 (2002).

11. Roys, R. L. *The Book of Chilam Balam of Chumayel*. (University of Oklahoma Press, 1967).

12. Tozzer, A. M. *Chichen Itza and its Cenote of Sacrifice: A comparative study of contemporaneous Maya and Toltec. Memoirs of the Peabody Museum of Archaeology and Ethnology*. (Harvard University, 1957).

13. Anda, G. D., Tiesler, V. & Zabala, P. Cenotes, espacios sagrados y la práctica del sacrificio humano en Yucatán. in *Los Investigadores de la Cultura Maya* (ed. Universidad Autónoma de Campeche) vol. 12 376–386 (Universidad Autónoma de Campeche, 2004).

14. Márquez Morfín, L. Sacrificio de niños en Chichen Itzá o práctica funeraria. in *Los niños como actores sociales ignorados. Levantando el velo, una mirada al pasado* (ed. Márquez Morfín, L.) 253–282 (ENAN-INAH; Conaculta, 2006).

15. Baudez, C.-F. & Latsanopoulos, N. Political structure, military training, and ideology at Chichen Itza. *Ancient Mesoamerica* **21**, 1–20 (2010).

16. Cagnato, C. Underground pits (chultunes) in the southern Maya lowlands: excavation results from Classic period Maya sites in northwestern Petén. *Ancient Mesoamerica* **28**, 75–94 (2017).

17. Puleston, D. E. An experimental approach to the function of Classic Maya chultuns. *Am Antiq* **36**, 322–335 (1971).

18. Brady, J. & Layco, W. Maya cultural landscapes and the subterranean: Assessing a century of chultun research. *International Journal of Archaeology* **6**, 46–55 (2018).

19. Prout, M. G. & Brady, J. E. Paleodemographics of Child Sacrifice at Midnight Terror Cave: Reformulating the Emphasis of Maya Sacrificial Practices. *Archaeological Discovery* **06**, 1–20 (2018).

20. del Castillo Chávez, O. & Williams-Beck, L. *Rituales k'ex al Dios del Maíz en Chichén Itzá y Mayapán: una tradición ritual del Clásico al Postclásico*. (2020).

21. Willard, T. A. *The city of the sacred well*. (The Century Co., 1926).

22. Arnold, C. & Frost, F. J. T. *The American Egypt: a record of travel in Yucatan*. (Hutchinson & Co., 1909).

23. Cucina, A. & Tiesler, V. Mortuary pathways and ritual meanings related to Maya human bone deposits in subterranean contexts. in *The Bioarchaeology of Space and Place* (ed. Wrobel, G. D.) 225–254 (Springer Press, 2014).

24. de Landa, D., Tozzer, A. M. & Bowditch, C. P. *Landa's Relación de Las Cosas de Yucatan: A Translation*. (Harvard University, 1941).

25. Clendinnen, I. *Ambivalent Conquests. Maya and Spaniard in Yucatan, 1517–1570*. (Cambridge University Press, 2003).

26. Vågene, Å. J. *et al.* Salmonella enterica genomes from victims of a major sixteenth-century epidemic in Mexico. *Nat Ecol Evol* **2**, 520–528 (2018).

27. Llamas, B. *et al.* From the field to the laboratory: Controlling DNA contamination in human ancient DNA research in the high-throughput sequencing era. *STAR: Science & Technology of Archaeological Research* **3**, 1–14 (2017).

28. Fellows Yates, J. A. *et al.* Reproducible, portable, and efficient ancient genome reconstruction with nf-core/eager. *PeerJ* **9**, e10947 (2021).

29. Mathieson, I. *et al.* Genome-wide patterns of selection in 230 ancient Eurasians. *Nature* **528**, 499–503 (2015).

30. Immel, A. *et al.* Analysis of Genomic DNA from Medieval Plague Victims Suggests Long-Term Effect of Yersinia pestis on Human Immunity Genes. *Mol Biol Evol* (2021) doi:10.1093/molbev/msab147.

31. Fu, Q. *et al.* A revised timescale for human evolution based on ancient mitochondrial genomes. *Current Biology* **23**, 553–559 (2013).

32. Lamnidis, T. C. *et al.* Ancient Fennoscandian genomes reveal origin and spread of Siberian ancestry in Europe. *Nat Commun* **9**, 5018 (2018).

33. Lamnidis, T. C. pMMRCalculator. https://github.com/TCLamnidis/pMMRCalculator (2020).

34. Götz, C. M. La alimentación de los mayas prehispánicos vista desde la zooarqueología. *An Antropol* **48**, 167–199 (2014).

35. Herrera Flores, D. A. & Markus Götz, C. La alimentación de los antiguos mayas de la península de Yucatán: consideraciones sobre la identidad y la cuisine en la época prehispánica. *Estudios de Cultura Maya* **XLIII**, 69–98 (2014).

36. Kennett, D. J. *et al.* Early isotopic evidence for maize as a staple grain in the Americas. *Sci Adv* **6**, eaba3245 (2020).

37. Tykot, R. H. Isotope Analyses and the Histories of Maize. in *Histories of Maize in Mesoamerica* (eds. Staller, J., Tykot, R. & Benz, B.) 12 (2010).

38. Fernández Souza, L., Toscano, L. & Zimmermann, M. De maíz y de cacao: aproximaciones a la cocina de las élites mayas en tiempos prehispánicos. *Estética y poder en la ciencia y la tecnología, Mérida, UAdY* 107–130 (2014).

39. Zimmermann, M. Starch grain extraction in lime-plastered archaeological floors. *STAR: Science & Technology of Archaeological Research* **7**, 31–42 (2021).

40. Somerville, A. D., Fauvelle, M. & Froehle, A. W. Applying new approaches to modeling diet and status: isotopic evidence for commoner resiliency and elite variability in the Classic Maya lowlands. *J Archaeol Sci* **40**, 1539–1553 (2013).

41. White, C. D. & Schwarcz, H. P. Ancient Maya diet: as inferred from isotopic and elemental analysis of human bone. *J Archaeol Sci* **16**, 451–474 (1989).

42. Wright, L. E. & White, C. D. Human biology in the Classic Maya collapse: evidence from paleopathology and paleodiet. *J World Prehist* **10**, 147–198 (1996).

43. Tykot, R. H. Contribution of stable isotope analysis to understanding dietary variation among the Maya. in *Archaeological Chemistry* vol. 831 214–230 (American Chemical Society, 2002).

44. Scherer, A. K. Bioarchaeology and the skeletons of the Pre-Columbian Maya. *Journal of Archaeological Research* **25**, 133–184 (2017).

45. Mansell, E. B., Tykot, R. H., Freidel, D. A., Dahlin, B. H. & Ardren, T. Early to Terminal Classic Maya diet in the Northern Lowlands of the Yucatán (Mexico). in *Histories of Maize: Multidisciplinary Approaches to the Prehistory, Linguistics, Biogeography, Domestication, and Evolution of Maize* (eds. Staller, J., Tykot, R. & Benz, B.) 173–185 (Elsevier, 2010).

46. Götz, C. M. La alimentación de los mayas prehispánicos vista desde la zooarqueología. *An Antropol* **48**, 167–199 (2014).

47. Reich, D. *et al.* Reconstructing Native American population history. *Nature* **488**, 370–374 (2012).

48. Kennett, D. J. *et al.* South-to-north migration preceded the advent of intensive farming in the Maya region. *Nat Commun* **13**, 1530 (2022).

49. Posth, C. *et al.* Reconstructing the Deep Population History of Central and South America. *Cell* **175**, 1185-1197.e22 (2018).

50. Patterson, N. *et al.* Ancient admixture in human history. *Genetics* **192**, 1065–1093 (2012).

51. Bergström, A. *et al.* Insights into human genetic variation and population history from 929 diverse genomes. *Science (1979)* **367**, (2020).

52. Mallick, S. *et al.* The Simons Genome Diversity Project: 300 genomes from 142 diverse populations. *Nature* **538**, 201–206 (2016).

53. Schraiber, J. G. Assessing the relationship of ancient and modern populations. *Genetics* **208**, 383–398 (2018).

54. González-Oliver, A., Márquez-Morfín, L., Jiménez, J. C. & Torre-Blanco, A. Founding Amerindian mitochondrial DNA lineages in ancient Maya from Xcaret, Quintana Roo. *Am J Phys Anthropol* **116**, 230–235 (2001).

55. Ochoa-Lugo, M. I. *et al.* Genetic Affiliation of Pre-Hispanic and Contemporary Mayas Through Maternal Linage. *Hum Biol* **88**, 136–167 (2016).

56. Mizuno, F. *et al.* Characterization of complete mitochondrial genomes of indigenous Mayans in Mexico. *Ann Hum Biol* **44**, 652–658 (2017).

57. Bodner, M. *et al.* The mitochondrial DNA landscape of modern Mexico. *Genes (Basel)* **12**, 1453 (2021).

58. Ruiz-Linares, A. *et al.* Admixture in Latin America: Geographic Structure, Phenotypic Diversity and Self-Perception of Ancestry Based on 7,342 Individuals. *PLoS Genet* **10**, e1004572 (2014).

59. Chacón-Duque, J. C. *et al.* Latin Americans show wide-spread Converso ancestry and imprint of local Native ancestry on physical appearance. *Nat Commun* **9**, (2018).

60. Ongaro, L. *et al.* Evaluating the Impact of Sex-Biased Genetic Admixture in the Americas through the Analysis of Haplotype Data. *Genes* vol. 12 Preprint at https://doi.org/10.3390/genes12101580 (2021).

61. Ongaro, L. *et al.* The genomic impact of European colonization of the Americas. *Current Biology* **29**, 3974-3986.e4 (2019).

62. Shriver, M. D. *et al.* The genomic distribution of population substructure in four populations using 8,525 autosomal SNPs. *Hum Genomics* **1**, 274 (2004).

63. Duforet-Frebourg, N., Luu, K., Laval, G., Bazin, E. & Blum, M. G. B. Detecting genomic signatures of natural selection with Principal Component Analysis: application to the 1000 Genomes data. *Mol Biol Evol* **33**, 1082–1093 (2016).

64. Auton, A. *et al.* A global reference for human genetic variation. *Nature* **526**, 68–74 (2015).

65. Amorim, E. *et al.* Genetic signature of natural selection in first Americans. *Proceedings of the National Academy of Sciences* **114**, 2195–2199 (2017).

66. Villalobos-Comparán, M. *et al.* The FTO gene is associated with adulthood obesity in the Mexican population. *Obesity (Silver Spring)* **16**, 2296–2301 (2008).

67. Ortega, P. E. N. *et al.* Association of rs9939609-FTO with metabolic syndrome components among women from Mayan communities of Chiapas, Mexico. *J Physiol Anthropol* **40**, 11 (2021).

68. Lara-Riegos, J. C. *et al.* Diabetes susceptibility in Mayas: Evidence for the involvement of polymorphisms in HHEX, HNF4α, KCNJ11, PPARγ, CDKN2A/2B, SLC30A8, CDC123/CAMK1D, TCF7L2, ABCA1 and SLC16A11 genes. *Gene* **565**, 68–75 (2015).

69. Ojeda-Granados, C. *et al.* Dietary, cultural, and pathogens-related selective pressures shaped differential adaptive evolution among Native Mexican populations. *Mol Biol Evol* **39**, msab290 (2022).

70. Kofler, R. & Schlötterer, C. Gowinda: unbiased analysis of gene set enrichment for genome-wide association studies. *Bioinformatics* **28**, 2084–2085 (2012).

71. Lindo, J. *et al.* A time transect of exomes from a Native American population before and after European contact. *Nat Commun* **7**, (2016).

72. Penman, B. S., Ashby, B., Buckee, C. O. & Gupta, S. Pathogen selection drives nonoverlapping associations between HLA loci. *Proceedings of the National Academy of Sciences* **110**, 19645–19650 (2013).

73. Buckee, C. O., Gupta, S., Kriz, P., Maiden, M. C. J. & Jolley, K. A. Long-term evolution of antigen repertoires among carried meningococci. *Proceedings of the Royal Society B: Biological Sciences* **277**, 1635–1641 (2010).

74. Barquera, R. *et al.* Diversity of HLA Class I and Class II blocks and conserved extended haplotypes in Lacandon Mayans. *Sci Rep* **10**, 3248 (2020).

75. Gonzalez-Galarza, F. F. *et al.* Allele frequency net database (AFND) 2020 update: gold-standard data classification, open access genotype data and new query tools. *Nucleic Acids Res* **48**, D783–D788 (2020).

76. Gómez-Casado, E. *et al.* Origin of Mayans according to HLA genes and the uniqueness of Amerindians. *Tissue Antigens* **61**, 425–436 (2003).

77. Dunstan, S. J. *et al.* Genes of the Class II and Class III Major Histocompatibility Complex Are Associated with Typhoid Fever in Vietnam. *J Infect Dis* **183**, 261–268 (2001).

78. Dunstan, S. J. *et al.* Variation at HLA-DRB1 is associated with resistance to enteric fever. *Nat Genet* **46**, 1333–1336 (2014).

79. de Vries, R. R. P., Meera Khan, P., Bernini, L. F., van Loghem, E. van & van Rood, J. J. Genetic control of survival in epidemics. *J Immunogenet* **6**, 271–287 (1979).

80. Reynisson, B., Alvarez, B., Paul, S., Peters, B. & Nielsen, M. NetMHCpan-4.1 and NetMHCIIpan-4.0: improved predictions of MHC antigen presentation by concurrent motif deconvolution and integration of MS MHC eluted ligand data. *Nucleic Acids Res* **48**, W449–W454 (2020).

81. Fuchs, K. M. & D'Alton, M. E. Chorionicity of Multiple Gestations. in *Obstetric Imaging: Fetal Diagnosis and Care* (eds. Copel, J. A. et al.) 639-641.e1 (Elsevier, 2018). doi:https://doi.org/10.1016/B978-0-323-44548-1.00158-3.

82. Christenson, A. J. *Popol Vuh. Sacred Book of the Quiché Maya People (Transl.)*. (University of Oklahoma Press, 2007).

83. Saturno, W. A., Stuart, D. & Taube, K. The Identification of the West Wall Figures at Pinturas Sub-1, San Bartolo, Petén. in *XVIII Simposio de Investigaciones Arqueológicas en Guatemala* (Museo Nacional de Guatemala, 2004).

84. Acuña-Alonzo, V. *et al.* A functional ABCA1 gene variant is associated with low HDL-cholesterol levels and shows evidence of positive selection in Native Americans. *Hum Mol Genet* **19**, 2877–2885 (2010).

85. León-Mimila, P. *et al.* Genome-Wide Association Study identifies a functional SIDT2 variant associated with HDL-C (High-Density Lipoprotein Cholesterol) levels and premature coronary artery disease. *Arterioscler Thromb Vasc Biol* **41**, 2494–2508 (2021).

86. Fritz, J., Lopez-Ridaura, R., Choudhry, S., Razo, C. & Lamadrid-Figueroa, H. The association of Native American genetic ancestry and high-density lipoprotein cholesterol: A representative study of a highly admixed population. *American Journal of Human Biology* **32**, e23426 (2020).

87. Wright, L. E. & White, C. D. Human biology in the Classic Maya collapse: Evidence from paleopathology and paleodiet. *J World Prehist* **10**, 147–198 (1996).

88. Scherer, A. K. Bioarchaeology and the Skeletons of the Pre-Columbian Maya. *Journal of Archaeological Research* **25**, 133–184 (2017).

89. Somerville, A. D., Fauvelle, M. & Froehle, A. W. Applying new approaches to modeling diet and status: isotopic evidence for commoner resiliency and elite variability in the Classic Maya lowlands. *J Archaeol Sci* **40**, 1539–1553 (2013).

90. Scherer, A. K., Wright, L. E. & Yoder, C. J. Bioarchaeological Evidence for Social and Temporal Differences in Diet at Piedras Negras, Guatemala. *Latin American Antiquity* **18**, 85–104 (2007).

91. Wright, L. E. & Schwarcz, H. P. Stable Carbon and Oxygen Isotopes in Human Tooth Enamel: Identifying Breastfeeding and Weaning in Prehistory. *Am J Phys Anthropol* **106**, 1–18 (1998).

92. Acuña-Soto, R., Stahle, D. W., Therrell, M. D., Griffin, R. D. & Cleaveland, M. K. When half of the population died: The epidemic of hemorrhagic fevers of 1576 in Mexico. *FEMS Microbiol Lett* **240**, 1–5 (2004).

93. Cook, S. F. & Simpson, L. B. *The population of Central Mexico in the sixteenth century*. vol. 31 (University of California Press, 1948).

94. Marr, J. S. & Kiracofe, J. B. Was the Huey Cocoliztli a Haemorrhagic Fever? *Med Hist* **44**, 341–362 (2000).

95. Cook, N. *Born to Die: Disease and New World Conquest, 1492-1650*. (Cambridge University Press, 1998).

96. Cook, S. F. & Borah, W. *The Indian population of central Mexico, 1531-1610*. (University of California Press, 1960).

97. Zambardino, R. A. Mexico's Population in the Sixteenth Century: Demographic Anomaly or Mathematical Illusion? *J Interdiscip Hist* **11**, 1–27 (1980).

98. Crosby, A. W. Virgin soil epidemics as a factor in the aboriginal depopulation in America. *William Mary Q* **33**, 289–299 (1976).

99. Crosby Jr., A. W. *The Columbian exchange: biological and cultural consequences of 1492*. (Greenwood Press, 1972).

100. Real Academia de la Historia & del Paso y Troncoso, F. *Relaciones geograficas de la diocesis de Mexico: Manuscritos de la Real Academia de la historia de Madrid y del Archivo de Indias en Sevilla. Anos 1579-1582*. (Est. tipografico 'Sucesores de Rivadeneyra,' 1905).

101. Viesca, C. Hambruna y epidemia en Anáhuac (1450-1454) en la época de Moctezuma Ilhuicamina. in *Ensayos sobre la historia de las epidemias en México* (eds. Florescano, E. & Elsa, M.) 157–165 (Instituto Mexicano del Seguro Social, 1982).

102. Márquez Morfín, L. La sífilis y su carácter endémico en la ciudad de México. *Historia Mexicana* vol. 64 1099–1161 Preprint at (2015).

103. Key, F. M. *et al.* Emergence of human-adapted Salmonella enterica is linked to the Neolithization process. *Nat Ecol Evol* **4**, 324–333 (2020).

104. Barquera, R. *et al.* The immunogenetic diversity of the HLA system in Mexico correlates with underlying population genetic structure. *Hum Immunol* **81**, 461–474 (2020).

105. de Buenaventura, J., Guerrero, G., Solís Robleda, Gabriela. & Bracamonte y Sosa, Pedro. *Historias de la conquista del Mayab, 1511-1697*. (Universidad Autónoma de Yucatán, Facultad de Ciencias Antropológicas, 1994).

106. Góngora-Biachi, R. A. La fiebre amarilla en Yucatán durante las épocas precolombina y colonial. *Revista Biomédica* **11**, 301–307 (2000).

107. Patch, R. W. Sacraments and disease in Mérida, Yucatán, Mexico, 1648-1727. *The Historian* **58**, 731–743 (1996).

Métodos

Contexto arqueológico y geográfico

Chichén Itzá es una de las más grandes e influyentes ciudades Mayas del periodo Clásico Terminal y hoy se erige como un sitio arqueológico icónico entre los más representativos de la arquitectura monumental prehispánica en América (Fig. 1). La arquitectura del sitio consta de varios estilos diferentes, y algunas de las estructuras se asemejan a las encontradas en el centro de México (Teotihuacán, Tula) y en las regiones Puuc y Chenes (noreste y centro de Yucatán, respectivamente) de las tierras bajas mayas[108]. El estilo maya Puuc representa la segunda fase del período Florecimiento en la Península de Yucatán (750-800 d.C.)[108,109]. Los tipos cerámicos y los datos epigráficos y radiométricos sugieren que la ciudad surgió como un importante centro político regional en la primera mitad del siglo IX y permaneció como tal hasta probablemente la primera mitad del siglo XI [109,110]. Las muestras antiguas utilizadas en este estudio pertenecían a individuos cuyos esqueletos fueron recuperados de las excavaciones arqueológicas de un chultún (cisterna) conectado a una cueva natural dentro del sitio arqueológico de Chichén Itzá, Yucatán, México. Los chultunes se utilizaron principalmente desde el Preclásico Medio hasta el Clásico Tardío-Postclásico (1000-1600 d.C.) en Mesoamérica, desde las tierras bajas mayas del norte de la península de Yucatán hasta los actuales Belice y Guatemala en las tierras bajas mayas del sur[16,111]. Los esqueletos muestreados, que han sido estudiados mediante pautas de antropometría y osteología en el Centro INAH Yucatán, fueron recuperados entre abril y junio de 1967, durante la construcción de una nueva pista cercana a El Castillo ("El Castillo", principal basamento del conjunto arqueológico y un edificio de especial significado calendárico[4,112]) y 300 m al noreste del Cenote Sagrado de Chichén Itzá. El chultún tenía piedras que habían sido trabajadas en el estilo que caracterizó el período Florescente, lo que indicaría que fue construido alrededor del Clásico Tardío o Terminal de Chichén Itzá[4,111]. La cámara y la cueva adyacente contenían numerosos restos óseos, algunos de ellos en posición anatómica. El elevado número de esqueletos y la posición en la que fueron encontrados, así como el hecho de que estuvieran cubiertos por corteza y polvo de piedra caliza en gran parte intactos[4], son en su mayoría consistentes con un entierro secundario/primario mixto con remoción periódica, lo que indica que el entierro tiene un carácter cultural, probablemente como ofrenda[4] relacionada con una ceremonia importante y recurrente[4,14]. Hubo un mínimo de 106 esqueletos de infantes y niños pequeños (de acuerdo al número de huesos temporales) sin ningún orden aparente ni contexto anatómico, excepto por un grupo de cráneos y bóvedas craneales alineadas de norte a sur de el muro separando las cámara y la cueva del límite sur de la cueva[14]. También, numerosos objetos de cerámica y barro, así como restos de huesos animales, se encontraron en la cueva[4]. El estilo cerámico y el contexto arqueológico indican que el chultún fue usado durante el periodo Florescente, contemporáneo al uso de otra cueva ritual cerca de Chichén Itzá, conocida como Balankanché (860 ± 130 AD)[4,113]. Una prueba de radiocarbono en una muestra de la corteza que cubre los huesos del entierro del chultún también sugirió una fecha de uso de ca. 920 ± 60 AD[111]. Sin embargo, nuestro fechamiento de carbono–14 (Fig. S2) sugiere que el entierro ritual se realizó tanto antes (~600 AD) como después (~1100 AD) del período Florescente. Según la determinación de la edad mediante el método de erupción dental[114], se encontró que la mayoría (~50%) de los individuos subadultos pertenecían al grupo de edad de 3 a 6 años[4]. El hecho de que la edad de los niños difiera del grupo de edad normalmente relacionado con la muerte en niños asociados con enfermedades infecciosas según la paleodemografía[19,114-116] apoya aún más la interpretación de este entierro como una ofrenda de sacrificio[4].

Las muestras biológicas de los mayas actuales se obtuvieron de individuos con ascendencia maya nativa americana autodeclarada de Tixcacaltuyub. El pueblo de Tixcacaltuyub, perteneciente al municipio de Yaxcabá, se ubica a 90 km al sureste de Mérida, a 16 km de Sotuta, y a 55 km al sureste de Chichén Itzá (Fig. 1). Tiene alrededor de 2100 habitantes. La aprobación ética para la recolección de muestras de sangre de individuos de Tixcacaltuyub, Yucatán, México, fue otorgada por el Comité de Ética e Investigación de la Universidad Autónoma de Yucatán (UADY), México. Todos los sujetos fueron informados sobre los objetivos y métodos utilizados y firmaron un formulario de consentimiento informado. Para evitar posibles sesgos, se excluyeron a los participantes con cualquier condición clínica asociada a HLA o cáncer[117,118].

Inclusión y ética

La comunidad de Tixcacaltuyub se autoidentifica como una comunidad maya y desde hace años mantiene una relación de cooperación con las Facultades de Química y Enfermería de la Universidad Autónoma de Yucatán (UADY), Mérida, Yucatán, como parte de proyectos que investigan la relación entre salud y estilo de vida en la comunidad. J.C.L.R, M.E.M.M. y J.C.T.R., junto con Claudia Tzec-Puch, han estado trabajando en colaboración con la comunidad para comunicar los resultados de pruebas clínicas y desarrollar intervenciones de salud en Tixcacaltuyub. Como resultado de estas intervenciones se han identificado barreras y oportunidades en el cuidado de la salud y actualmente se trabaja con la participación activa de la comunidad para implementar un modelo corresponsable en dicho campo. En abril de 2023, J.C.L.R. y R.B. visitaron Tixcacaltuyub para mantener reuniones con participantes y estudiantes de primaria, secundaria y UADY para devolver los resultados de los hallazgos genéticos y recopilar comentarios de la comunidad, incluyendo cómo concilian estos resultados con sus propios puntos de vista. Los comentarios de estas interacciones se han incorporado en el manuscrito final (para obtener más información, consulte *Supplementary methods: Community engagement activities*).

Individuos y muestras

Analizamos el material recuperado de los huesos petrosos izquierdos de 64 individuos (YCH) de la colección Chichén Itzá Chultún. Las muestras óseas fueron recolectadas en condiciones controladas en el Laboratorio de Osteología del Centro INAH Yucatán con un protocolo destinado a minimizar la contaminación. Agradecemos al Consejo de Arqueología, Instituto Nacional de Antropología e Historia (INAH, Ciudad de México, México) por el permiso otorgado para analizar a estos individuos (Proyecto: Caracterización genética de una muestra de población de Chichén Itzá para el período Clásico Tardío a partir del análisis del ADN; oficio número: 401.1S.3-2017/482; 03.10.2017). Se mantuvieron registros fotográficos de las muestras durante todo el procedimiento. El ADN moderno se extrajo de muestras de sangre obtenidas de 67 individuos adultos no emparentados (TIX) de Tixcacaltuyub, Yucatán, México, bajo un protocolo aprobado por el Comité de Ética e Investigación de la Universidad Autónoma de Yucatán (UADY, Mérida, Yucatán, México) que otorga permiso para recolectar muestras de sangre y realizar análisis del material genético obtenido de dichas muestras (Proyecto: Bienestar Comunitario: Proyecto de capacitación para la autogestión de la salud de personas con DT2 y sus familias, en la comunidad de Tixcacaltuyub y Yaxcabá; oficial número de aviso: F-FENC-SAC-14/REV: 04; número de registro: 09/17), y realizado de acuerdo con los requisitos de la Declaración de Helsinki (2008) y la Ley General de Salud de México. Para YCH, el polvo de hueso (~90 mg) se obtuvo de la parte más densa de cada hueso petroso en instalaciones especiales para protocolos de ADN antiguo y después de un protocolo de lejía/enjuague y descontaminación UV27. Para TIX, se obtuvieron 5 ml de sangre periférica mediante venopunción después de que cada participante fuera informado sobre el procedimiento, el objetivo del protocolo y los riesgos potenciales, y después de firmar una carta de consentimiento. Para TIX, el ADN se extrajo en un laboratorio exclusivo de la UADY. Sus muestras fueron anonimizadas a partir de este momento.

Fechamiento por Carbono – 14

Según el informe emitido por *The Curt-Engelhorn-Centre for Archaeometry* (Mannheim, Alemania), las porciones del hueso petroso de 26 muestras de YCH fueron pretratadas y analizadas mediante un procedimiento estandarizado. Se extrajo colágeno de las muestras óseas (aproximadamente 1 g, utilizando una versión modificada del método Longin[119]), se purificó mediante ultrafiltración (fracción >30 kD) y se liofilizó. Luego, el colágeno se quemó hasta

obtener CO_2 en un analizador elemental (EA). Después, el CO_2 se convirtió catalíticamente en grafito y se analizó utilizando un sistema AMS tipo MICADAS (Ionplus AG, Dietikon, Suiza). Las relaciones isotópicas $^{14}C/^{12}C$ y $^{13}C/^{12}C$ de las muestras, el estándar de calibración (ácido oxálico-II), los controles blanco y los estándares se midieron simultáneamente en el sistema AMS. Las edades de ^{14}C se normalizan a to $\delta^{13}C$=-25‰[120] con una incertidumbre típica de 2‰ y se calibran utilizando el conjunto de datos IntCal20 y el software Oxcal (ver. 4.3.2) [121,122].

Análisis de isótopos estables.

La información sobre las tendencias dietéticas entre poblaciones pasadas permite investigar las conexiones entre la dieta y el estatus social, las costumbres culturales vinculadas a los alimentos, los impactos ambientales en la subsistencia y quizás incluso la movilidad individual[123,124]. Las mediciones de colágeno óseo $\delta^{13}C$ y $\delta^{15}N$ reflejan desproporcionadamente el componente proteico de la dieta de un individuo durante el período de formación de tejido y, en menor medida, las fuentes de lípidos y carbohidratos[125]. Para reconstruir la dieta de los individuos sub-adultos de Chichén Itzá estudiados, analizamos el colágeno óseo de los huesos temporales y petrosos (Fig. S4). El colágeno se extrajo mediante un procedimiento estándar[126] (más información en *Supplementary methods*). Se utilizó la relación atómica C:N junto con los rendimientos de colágeno para determinar la calidad de la conservación del colágeno. Los rendimientos de colágeno superiores al 1% en peso se consideran aceptables para los valores de carbono y nitrógeno[127], mientras que la relación C:N debe oscilar entre 2,9 y 3,6 para las muestras arqueológicas[128] (Tabla S1).

Extracción de ADN

Para YCH, el polvo de hueso se descalcificó y las proteínas se digirieron mediante una incubación durante la noche (>16 horas) a 37°C en una solución que contenía EDTA y proteinasa K[27]. El ADN se purificó del sobrenadante mediante un método basado en columnas de sílice para ensayos de grandes volúmenes (High Pure Viral Nucleic Acid Large Volume Kit, Roche Molecular Systems, Inc.; Pleasanton, CA, EE. UU.). El ADN se eluyó en 100µl de TET (Tris 10 mM, EDTA 1 mM y Tween al 0,05%) y se congeló a -20 °C hasta la preparación de la biblioteca[129]. Para los participantes contemporáneos, se recolectaron 5 ml de sangre periférica en tubos de recolección de sangre BD Vacutainer® que contenían K_2-EDTA (Becton, Dickinson and Company, Franklin Lakes, Nueva Jersey, EE. UU.) y el ADN se extrajo utilizando el kit Quick-DNA Miniprep Plus. (Zymo Research Corporation, Irvine, CA, EE. UU.) siguiendo las instrucciones de los desarrolladores. Dado que nuestros protocolos están optimizados para ADN antiguo de longitud corta, y para evitar posibles sesgos a través de métodos de laboratorio, cortamos el ADN extraído de individuos modernos mediante corte ultrasónico de ADN a una longitud promedio comparable a la del ADNa. Por lo tanto, se cortaron 50 µl de una dilución de 50 ng/µl de cada una de las muestras de ADN modernas hasta una longitud de fragmento promedio de 150 pb utilizando un *Covaris M220 Focused ultrasonicator* (Covaris Ltd., Brighton, Reino Unido).

Preparación de la biblioteca

Construimos bibliotecas tratadas con ADN glicosilasa sin uracilo (no UDG) utilizando 15 µL de cada extracto de YCH para evaluar la autenticidad del ADN extraído después de obtener los patrones de daño característicos asociados con el ADN antiguo[130]. Luego, utilizamos 20µL de cada extracto de YCH para construir bibliotecas UDG-half con adaptadores específicos de Illumina siguiendo un protocolo de preparación de bibliotecas bicatenarias modificado como se describió anteriormente[131]. Construimos bibliotecas no tratadas con UDG para extractos TIX utilizando 20µL de cada extracto de ADN moderno. Tanto las bibliotecas YCH como TIX se cuantificaron mediante qPCR con los primers IS7 e IS8 en un ensayo de cuantificación utilizando un kit qPCR *DyNAmo SYBR Green* (Thermo Fisher Scientific Inc.; Waltham, MA) en la plataforma *LightCycler® 96* (Roche Diagnostics Inc.; Risch -Rotkreuz, Suiza).

Para los extractos de ADN de los individuos para los cuales los datos obtenidos mediante la secuenciación de las bibliotecas tratadas con UDG no fueron suficientes, empleamos un nuevo protocolo de biblioteca que utiliza el enlace direccional acotada de los adaptadores P5 y P7 de Illumina para convertir nativamente el ADN monocatenario y el ADN doble cadena desnaturalizado por calor en bibliotecas de secuenciación en una única reacción enzimática

utilizando la versión totalmente automatizada del protocolo[132-134]. Seguimos el protocolo descrito por los autores utilizando 25µL de extracto de ADN para cada individuo (más información en *Supplementary methods: Preparation of single stranded libraries*).

Cada biblioteca se identificó con el par de índices respectivo en reacciones dobles de 100µL utilizando *PfuTurbo DNA Polymerase* (Agilent Technologies, Inc.; Santa Clara, CA, EE. UU.) para las bibliotecas de doble cadena y *AccuPrime™ Pfx DNA Polymerase* (Thermo Fisher Scientific Inc., Waltham, MA, EE. UU.). Los productos indexados para cada biblioteca se combinaron, se purificaron en columnas de sílice usando *MinElute PCR Purification Kit* (QIAGEN N.V.; Hilden, Alemania), se eluyeron en 44 µl de TET y nuevamente se cuantificaron por qPCR, ahora usando los primers IS5 e IS6. El acondicionamiento para la secuenciación incluyó la amplificación del producto purificado en reacciones de 4x100 µL usando Herculase II Fusion DNA Polymerase (Agilent Technologies, Inc.; Santa Clara, CA, USA) siguiendo las especificaciones del fabricante con 0,3 µM de cada primer IS5/IS6, siguiendo un purificación sobre columnas de sílice también utilizando el kit de purificación por PCR MinElute y elución en un volumen final de 22 µL de TET. Se diluyeron dos microlitros del producto acondicionado 1:10 y se cuantificaron utilizando el protocolo *Agilent 2100 Bioanalyzer DNA 1000* (Agilent Technologies, Inc.; Santa Clara, CA, EE. UU.). Independientemente, se prepararon combinaciones equimolares (concentración final de 10 mM) para las bibliotecas YCH, bibliotecas TIX y bibliotecas blanco de extracción y bibliotecas para secuenciación directa en la plataforma Illumina HiSeq 4000 Systems (Illumina, Inc., San Diego, CA, EE. UU.)[135,136]. Las bibliotecas YCH se secuenciaron con una profundidad de 5 millones de lecturas, mientras que las bibliotecas TIX se secuenciaron con una profundidad de 11 millones de lecturas y posteriormente, todas se analizaron para obtener parámetros básicos de control de calidad utilizando nf-core/eager ver. 2.3.4[28].

Capturas de genes inmunes y del genoma completo.

Utilizando un enfoque de captura en solución basado en secuencias de sonda inmortalizadas modificadas[137], se enriquecieron secuencias blanco de genes de inmunidad, ADN mitocondrial, cromosoma-Y o un panel de 1,237,207 polimorfismos de un solo nucleótido (SNP) a partir del ADN total en las bibliotecas de secuenciación[29,30,138–141]. Después del enriquecimiento, los grupos de bibliotecas capturados se secuenciaron en pares en la plataforma Illumina Hiseq 4000 (Illumina, Inc., San Diego, CA, EE. UU.) con 75 ciclos que proporcionaron ~20 millones de lecturas por ensayo.

Control de calidad y procesamiento de datos.

Para las bibliotecas YCH y TIX, realizamos análisis de los datos de secuenciación capturados utilizando nf-core/eager ver. 2.3.4[28]. Se utilizó AdapterRemoval v.2[142] para remover las secuencias de adaptadores y eliminar dímeros de adaptadores y lecturas de secuencias de baja calidad (longitud mínima = 30; calidad de base mínima = 20). Las secuencias pre-procesadas se mapearon al ensamblaje del genoma humano GRCh37 (hg19) del Genome Reference Consortium[143] utilizando *BWA* ver. 0.7.12[144] y una seed length de 32. En el caso de las muestras antiguas, las frecuencias de incorporación errónea de C a T típicas del ADNa se obtuvieron utilizando *mapDamage* 2.0[145] para evaluar la autenticidad de los fragmentos de ADN antiguos de las bibliotecas tratadas con UDG-half. El sexo genético de los individuos analizados se asignó utilizando datos de captura de SNP calculando la relación entre la cobertura promedio de los cromosomas X y Y y la cobertura autosómica promedio normalizada por la longitud del cromosomica en los SNP objetivo[32]. Muestras con una tasa en X entre 0.35 y 0.55 y la tasa en Y entre 0.4 y 7 se confirmaron como masculinos. Se utilizó *ANGSD* para estimar la contaminación nuclear, ya que se espera que los individuos masculinos sean homocigotos en cada posición del cromosoma X149. Usamos *samtools pileup* (parámetros –q 30 –Q 30 –B) para generar un archivo de acumulación a partir de los datos de secuencia fusionados de cada individuo, y usamos un script personalizado (pileupCaller ver. 8.2.2[147]) para genotipar los individuos, usando un enfoque pseudo – haploide aleatorio. Para cada posición en nuestro panel de captura, se seleccionó una lectura aleatoria para cada individuo y se asumió que el alelo de esa lectura era el genotipo homocigótico del individuo en esa posición. Para comparar con los datos disponibles de las poblaciones de Mesoamérica y

Centroamérica, fusionamos nuestros SNPs con los 593,124 SNPs del conjunto de datos de Human Origins[50]. Solo los individuos que retuvieron >20 000 SNP en este ensayo se mantuvieron para los análisis posteriores.

Marcadores uniparentales

Los haplogrupos de ADNmt se determinaron mapeando lecturas con la secuencia de referencia revisada de Cambridge[148]. Para las secuencias resultantes, utilizamos HaploGrep2[149] y HAPLOFIND[150] para asignar y confirmar los haplogrupos de ADNmt correspondientes (Fig. S6). Para las asignaciones del haplogrupo Y, creamos acumulaciones de lecturas para cada individuo que se asignaron a los SNPs del cromosoma Y (Y-Chr) como se enumera en ISOGG Y-DNA Haplogroup Tree (v15.73; https://isogg.org/tree/). Luego asignamos manualmente haplogrupos Y-Chr para cada individuo en función del SNP downstream recuperado después de evaluar la presencia de mutaciones upstream a lo largo de la filogenia del haplogrupo Y-Chr (Fig. S6). Los haplogrupos completos de ADNmt y los genotipos Y-Chr se pueden encontrar en las Tablas S9 (YCH) y S10 (TIX).

Exploraciones de selección de todo el genoma

Utilizando los datos genómicos antiguos (YCH; N=64) y actuales (TIX; N=68), realizamos dos comparaciones de LSBL: primero YCH versus íberos de España (N=102) y Han de China (N=103) del proyecto 1000 Genomas[64], y luego TIX vs íberos y YCH para realizar pruebas de selección por separado de YCH e íberos. Después del filtrado de control de calidad, eliminamos el YCH046 individual según su posición en la PCA. No se analizaron los cromosomas sexuales, se excluyeron los SNP con frecuencias de alelos menores inferiores a 0,01, así como los individuos con más del 97% de datos faltantes, y finalmente delimitamos los SNP para determinar el desequilibrio de ligamiento (LD) usando el siguiente comando: --indep-pairwise 200 25 0,4. El 0,5 % de los SNP principales se anotaron con *biomaRt* (versión 3.15)[151]. Las longitudes de rama específicas del lugar *x, y* y *z* se calculan utilizando distancias F_{ST} por pares, d_{AB}, d_{BC} y d_{AC}, donde $x = (d_{AB} + d_{AC} – d_{BC})/2$, $y = (d_{AB} + d_{BC} - d_{AC})/2$, $z = (d_{AC} + d_{BC} - d_{AB})/2$ y A, B y C son las tres poblaciones bajo consideración (Extensión de Fig. 01).

Asignación de alelos HLA, reconstrucción de haplotipos, análisis estadísticos y ensayos de predicción de unión in silico.

Aplicamos una versión de desarrollo de OptiType[152] en datos de secuencia de bibliotecas de inmunidad capturadas, mapeados contra un panel de referencia HLA personalizado que contiene 1025 alelos con designación CIWD 3.0 "común" o "intermedia" (https://github.com/FRED-2 /OptiType, tag GRG). El número de posibles mejores coincidencias se fijó en infinito. De esta forma obtuvimos alelos HLA clase I y clase II para los genes HLA-A, HLA-B, HLA-C, HLA-DRB1, HLA-DQA1, HLA-DQB1, HLA-DPA1 y HLA-DPB1. Setenta y dos de las 882 llamadas de alelos sin procesar (8,1%) con patrones de cobertura anómalos inducidos por lecturas cruzadas en múltiples loci fueron anuladas a favor de predicciones de alelos secundarios. Los haplotipos se asignaron en función de frecuencias y propiedades previamente informadas, como el desequilibrio de ligamiento[75,153,154] y el parentesco cuando fue posible. Para comparar las frecuencias de HLA entre Chichén Itzá antes del contacto y Tixcacaltuyub después del contacto, realizamos pruebas exactas de Fisher[155] y ajustamos los valores de p para múltiples comparaciones de acuerdo con la corrección de Benjamini y Hochberg[156] que controla la tasa de descubrimiento falso (FDR). Utilizamos el ajuste FDR ya que, en comparación con métodos como la corrección de Bonferroni, el enfoque FDR controla una proporción baja de falsos positivos, en lugar de garantizar la inexistencia de falsos positivos, lo que resulta en un mayor poder estadístico[156]. Se ha argumentado[72] que ciertos tipos de asociación no superpuesta entre los loci HLA pueden ser una característica de la selección de patógenos. El valor f^*_{adj}[72,73,157] se calculó para cada combinación de pares de loci HLA en el conjunto de datos, utilizando haplotipos YCH y TIX para los cuales teníamos datos para cada locus HLA. Para proporcionar un punto de comparación para la puntuación f^*_{adj} de cada par de locus tanto para YCH como para TIX, generamos 5000 permutaciones aleatorias del orden de los alelos totales en uno de los loci de cada par y volvimos a calcular f^*_{adj}para cada conjunto de datos aleatorios. Generamos distribuciones de posibles puntuaciones f^*_{adj} para cada par de loci en el conjunto de datos que se obtendrían si los alelos en esos loci se asociaran completamente al azar.

Luego calculamos la diferencia entre el valor de f^*_{adj}calculado a partir de cada conjunto de datos para cada par de loci y la media de las puntuaciones de f^*_{adj} calculadas a partir de los datos aleatorios para ese par de loci, luego dividimos esa diferencia por la desviación estándar de f^*_{adj} calculado a partir de datos aleatorizados. Las puntuaciones resultantes nos permitieron clasificar los pares HLA en orden de qué tan extremo era el nivel de no superposición que mostraban en relación con asociaciones completamente aleatorias entre los mismos alelos (más información en *Supplementary methods: Non-overlapping associations*). Para evaluar más a fondo el papel de los alelos HLA clase II en la resistencia a la infección por *Salmonella enterica*, realizamos predicciones de unión *in-silico* para los alelos *HLA-DRB1/3/4/5* y los pares de alelos *-DQA1/DQB1* encontrados en individuos YCH y péptidos derivados de 18 proteínas de *Salmonella spp.* que previamente se ha informado que son altamente inmunogénicas en humanos (consulte la sección *Supplementary methods: In silico binding prediction assays*) usando NetMHCIIpan-4.0[80] tal como se implementa en Immune Epitope Database (IEDB) y Analysis Resource virtual machine image [158 ,159]. Las uniones se clasificaron como fuertes (rango <2%) o débiles (2% ≤ rango ≤ 10%) según los valores de rango ajustados recomendados por los autores y las investigaciones publicadas anteriormente.

Análisis de componentes principales.

Se utilizó Smartpca (versión 16000) del paquete Eigensoft[161] para calcular los componentes principales (PC) de variación en el conjunto de datos, utilizando las opciones "lsqproject: YES" y "shrinkmode: YES". Inicialmente, proyectamos los individuos antiguos en PC calculados sobre la variación genética en 371 poblaciones mundiales, para acceder a las ascendencias a nivel continental de los individuos antiguos. Luego proyectamos a los individuos antiguos en PC calculando la variación de 172 poblaciones nativoamericanas, tanto modernas como antiguas (más información en Supplementary methods).

Análisis de ADMIXTURE.

Usamos ADMIXTURE ver. 1.3.0[162] (un algoritmo de agrupamiento basado en máxima verosimilitud), para estimar la estructura genética presente en nuestras muestras, después de excluir variantes con una frecuencia de alélica menor de 0.01 y después de la delimitación de LD usando Plink (ver. 1.90) con un tamaño de paso de 5, un tamaño de ventana de 200 y un umbral R2 de 0,5[162]. Para K=2 a K=13, estimamos el error de validación cruzada (CV) con 100 réplicas de bootstrap en un modelo no supervisado utilizando un panel de poblaciones que se componen de cuatro grupos de ascendencia de origen africano, europeo, papú y americano basado en el conjunto de datos del Simons Genome Diversity Project (SGDP)[52] más otras poblaciones relevantes o un conjunto de las poblaciones antes mencionadas más un conjunto de 66 poblaciones nativas americanas modernas y antiguas para dar cuenta de diferentes fuentes de contribución genética (más información en *Supplementary methods*). Los errores CV más bajos correspondieron a K=4 y K=8, respectivamente.

Estadísticos–F y pruebas de modelado de ascendencia.

Para evaluar las relaciones genéticas y los patrones de mezclas sugeridos por el análisis PCA y ADMIXTURE, llevamos a cabo análisis estadísticos–F utilizando el software *Xerxes* CLI (v. 0.3.0.0) del marco Poseidon (v. 2.5.0)[163]. Calculamos estadísticos–F_3 de la forma f_3 (Grupo externo; objetivo, X) para medir la cantidad de deriva genética compartida de las poblaciones objetivo y X después de su divergencia de un grupo externo africano; donde X es ya sea YCH o TIX, y grupo externo corresponde a Mbuti del Congo[50]. Luego utilizamos un subconjunto de poblaciones de nativos americanos (aquellas con las puntuaciones F_3 más altas de nuestra prueba anterior) para realizar pruebas F_4 de la forma f_4(Mbuti, YCH; prueba, TIX) para investigar si los grupos YCH y TIX están más estrechamente relacionados entre sí o compartieron un excedente de alelos con cualquier población en la prueba de posición. Un valor negativo implica que YCH y la prueba, o TIX y Mbuti comparten más alelos de los esperados según la hipótesis nula de una relación simétrica entre YCH y TIX. Por otro lado, un valor positivo sugiere que YCH y TIX comparten un excedente de alelos entre sí. Para probar si los individuos recién secuenciados eran suficientemente similares genéticamente como para agruparlos en un grupo, utilizamos qpWave[47] ver. 420, un enfoque de modelado que evalúa la probabilidad de que dos individuos deriven de la misma ascendencia, en relación con un conjunto dado de referencias que incluye poblaciones de todo el mundo. Un valor inferior a 0,05 indica una probabilidad baja, de lo que

concluimos que uno de los individuos tiene un perfil de ascendencia diferente. Para la mayoría de las pruebas, los individuos respectivos son consistentes con derivar de la misma ascendencia analizada en relación con las poblaciones Mbuti.DG, Onge.DG, Papuan.DG, Han.DG, Russia_MA1_HG.SG, USA_Ancient_Beringian.SG, USA_Anzick.SG, Mixe.DG, Mexico_Zapotec.DG, Belize_MayahakCabPek_9300BP, Karitiana.DG, Piapoco.DG, Peru_LaGalgada_4100BP, Pima, Cabecar. Hay pocas excepciones en las que un individuo muestra una baja probabilidad de compartir ascendencia con otros múltiples individuos. altas tasas de pares especialmente con baja probabilidad fueron producidos por los individuos YCH006, YCH011, YCH037, YCH046, YCH59 e YCH064. Para investigar qué ascendencias diferencian al individuo respectivo de la mayoría de los individuos, calculamos las afinidades diferenciales utilizando una estadística F_4 de la forma f_4(Mbuti.DG, X; YCH Group, Y), donde *X* sería el individuo respectivo e *Y* en los grupos actuales[47] y grupos e individuos antiguos publicados[49,164–173]. En esta prueba, asumiríamos una puntuación positiva si el grupo o individuo en Y tiene una mayor afinidad con el individuo evaluado (X) en comparación con el grupo. Inesperadamente, no existe ningún grupo o individuo antiguo o moderno que pueda explicar el comportamiento en el análisis de qpWave. Para probar si los hablantes mayas actuales ("maya" según The Simons Genome Diversity Project[52], kaqchikel) eran consistentes con derivar de la misma ascendencia que los antiguos individuos de Chichén Itzá, los probamos en relación con el conjunto de referencia de poblaciones Mbuti.DG, Onge.DG, Papuan.DG, Han.DG, Russia_MA1_HG.SG, USA_Ancient_Beringian.SG, USA_Anzick.SG, Mixe.DG, Mexico_Zapotec.DG, Belize_MayahakCabPek_9300BP, Karitiana.DG en ambos casos, el resultado de los valores de *p* (Kaqchikel: 0.08, "Mayan": 0.755) sugieren que los grupos antiguos y actuales comparten la misma ancestría. La misma prueba no fue exitosa para otras poblaciones actuales tales como los hablantes de Gen-Pano-Carib o Chibchan-Paezan así como para los individuos aparentemente no mestizos de Tixcacaltuyub, posiblemente atribuible a herencia europea no detectada en los análisis de agrupamiento basados en modelos (Fig. 2 & 3). Al modelar su composición de ascendencia en relación con las poblaciones Mbuti.DG, Onge.DG, Papuan.DG, Han.DG, Russia_MA1_HG.SG, USA_Ancient_Beringian.SG, USA_Anzick.SG, Mixe.DG, Mexico_Zapotec.DG, Belize_MayahakCabPek_9300BP, Karitiana.DG como un mestizaje de Yoruba.DG, Spanish.DG y "Maya", el modelo de trabajo (p=0,11) sugiere una composición de 92% de ascendencia maya, 7% española y 0,03% yoruba.

Referencias:

108. Potter, D. F. Prehispanic Architecture and Sculpture in Central Yucatan. Am Antiq 41, 430–448 (1976).
109. Andrews IV, E. W. Archaeology and prehistory in the northern Maya lowlands: An introduction. in Handbook of Middle American Indians, Volume 13: Guide to Ethnohistorical Sources, Part Two (ed. Wauchope, R.) vol. 13 288–330 (University of Texas Press, 1964).
110. Andrews, A. P., Andrews, E. W. & Castellanos, F. R. The northern Maya collapse and its aftermath. Ancient Mesoamerica 14, 151–156 (2003).
111. Hermes, B. & Calderón, Z. Chultunes. in El sitio maya de Topoxté. Investigaciones en una isla del lago Yaxhá, Petén, Guatemala (ed. Wurster, W. W.) 66–74 (Verlag Philipp von Zabern, 2000).
112. Milbrath, S. Astronomical images and orientations in the architecture of Chichen Itza. in New Directions in American Archaeoastronomy (Proceedings of the 46th International Congress of Americanists: Amsterdam, Netherlands, 1988) (ed. Aveni, A. F.) 57–79 (BAR International Series 454, 1988).
113. Andrews, E. W. Balankanche, throne of the tiger priest. (Middle American Research Institute, Tulane University, 1970).
114. Ubelaker, D. H. Human skeletal remains: excavation, analysis, interpretation. (Aldine Pub. Co, 1978).
115. Hernández Espinosa, P. O. La regulación del crecimiento de la población en el México prehispánico. (Instituto Nacional de Antropología e Historia (INAH), 2006).
116. Verdugo, C. et al. Implications of age and sex determinations of ancient Maya sacrificial victims at Midnight Terror Cave. Int J Osteoarchaeol 30, 458–468 (2020).
117. Karnes, J. H. et al. Phenome-wide scanning identifies multiple diseases and disease severity phenotypes associated with HLA variants. Sci Transl Med 9, 1–13 (2017).

118. Fernández-Torres, J., Flores-Jiménez, D., Arroyo-Pérez, A., Granados, J. & López-Reyes, A. HLA-B*40 allele plays a role in the development of Acute Leukemia in Mexican population: a case-control study. Biomed Res Int 2013, 705862 (2013).

119. Brown, T. A., Nelson, D. E., Vogel, J. S. & Southon, J. R. Improved Collagen Extraction by Modified Longin Method. Radiocarbon 30, 171–177 (1988).

120. Stuiver, M. & Polach, H. A. Discussion Reporting of 14 C Data . Radiocarbon 19, 355–363 (1977).

121. Reimer, P. J. et al. The IntCal20 Northern Hemisphere Radiocarbon Age Calibration Curve (0–55 cal kBP). Radiocarbon 62, 725–757 (2020).

122. Ramsey, C. B. & Lee, S. Recent and Planned Developments of the Program OxCal. Radiocarbon 55, 720–730 (2013).

123. Błaszczyk, D. et al. Social status and diet. Reconstruction of diet of individuals buried in some early medieval chamber graves from Poland by carbon and nitrogen stable isotopes analysis. J Archaeol Sci Rep 38, 103103 (2021).

124. Pérez-Ramallo, P. et al. Stable isotope analysis and differences in diet and social status in northern Medieval Christian Spain (9th–13th centuries CE). J Archaeol Sci Rep 41, 103325 (2022).

125. Ambrose, S. H. & Norr, L. Experimental evidence for the relationship of the carbon isotope ratios of whole diet and dietary protein to those of bone collagen and carbonate. in Prehistoric Human Bone: Archaeology at the Molecular Level (eds. Lambert, J. B. & Grupe, G.) 1–37 (Springer Berlin Heidelberg, 1993). doi:10.1007/978-3-662-02894-0_1.

126. Richards, M. P. & Hedges, R. E. M. Stable isotope evidence for similarities in the types of marine foods used by late Mesolithic humans at sites along the Atlantic coast of Europe. J Archaeol Sci 26, 717–722 (1999).

127. van Klinken, G. J. Bone collagen quality indicators for palaeodietary and radiocarbon measurements. J Archaeol Sci 26, 687–695 (1999).

128. DeNiro, M. J. Postmortem preservation and alteration of in vivo bone collagen isotope ratios in relation to palaeodietary reconstruction. Nature 317, 806–809 (1985).

129. Rohland, N. & Hofreiter, M. Comparison and optimization of ancient DNA extraction. Biotechniques 42, 343–352 (2007).

130. Dabney, J. et al. Complete mitochondrial genome sequence of a Middle Pleistocene cave bear reconstructed from ultrashort DNA fragments. Proc Natl Acad Sci U S A 110, 15758–15763 (2013).

131. Briggs, A. W. et al. Patterns of damage in genomic DNA sequences from a Neandertal. Proc Natl Acad Sci U S A 104, 14616–14621 (2007).

132. Gansauge, M.-T. & Meyer, M. Single-stranded DNA library preparation for the sequencing of ancient or damaged DNA. Nat Protoc 8, 737 (2013).

133. Gansauge, M.-T. & Meyer, M. A method for single-stranded ancient DNA library preparation. in Ancient DNA: Methods and Protocols (eds. Shapiro, B. et al.) 75–83 (Springer New York, 2019). doi:10.1007/978-1-4939-9176-1_9.

134. Gansauge, M.-T., Aximu-Petri, A., Nagel, S. & Meyer, M. Manual and automated preparation of single-stranded DNA libraries for the sequencing of DNA from ancient biological remains and other sources of highly degraded DNA. Nat Protoc 15, 2279–2300 (2020).

135. Meyer, M. & Kircher, M. Illumina sequencing library preparation for highly multiplexed target capture and sequencing. Cold Spring Harb Protoc 5, pdb.prot5448 (2010).

136. Rohland, N., Harney, E., Mallick, S., Nordenfelt, S. & Reich, D. Partial uracil-DNA-glycosylase treatment for screening of ancient DNA. Philosophical Transactions of the Royal Society B: Biological Sciences 370, 20130624–20130624 (2014).

137. Gnirke, A. et al. Solution hybrid selection with ultra-long oligonucleotides for massively parallel targeted sequencing. Nat Biotechnol 27, 182–189 (2009).

138. Fu, Q. et al. DNA analysis of an early modern human from Tianyuan Cave, China. Proc Natl Acad Sci U S A 110, 2223–2227 (2013).

139. Fu, Q. et al. An early modern human from Romania with a recent Neanderthal ancestor. Nature 524, 216–219 (2015).

140. Barquera, R. et al. Origin and health status of first-generation Africans from early Colonial Mexico. Current Biology 30, 2078-2091.e11 (2020).

141. Rohrlach, A. B. et al. Using Y-chromosome capture enrichment to resolve haplogroup H2 shows new evidence for a two-path Neolithic expansion to Western Europe. Sci Rep 11, 15005 (2021).

142. Schubert, M., Lindgreen, S. & Orlando, L. AdapterRemoval v2: Rapid adapter trimming,

identification, and read merging. BMC Res Notes 9, 1–7 (2016).

143. Lander, E. S. et al. Initial sequencing and analysis of the human genome. Nature 409, 860–921 (2001).

144. Li, H. & Durbin, R. Fast and accurate long-read alignment with Burrows-Wheeler transform. Bioinformatics 26, 589–595 (2009).

145. Jónsson, H., Ginolhac, A., Schubert, M., Johnson, P. L. F. & Orlando, L. MapDamage2.0: Fast approximate Bayesian estimates of ancient DNA damage parameters. Bioinformatics 29, 1682–1684 (2013).

146. Korneliussen, T. S., Albrechtsen, A. & Nielsen, R. ANGSD: Analysis of Next Generation Sequencing Data. BMC Bioinformatics 15, 356 (2014).

147. Schiffels, S. sequenceTools. https://github.com/stschiff/sequenceTools.git (2018).

148. Andrews, R. M. et al. Reanalysis and revision of the cambridge reference sequence for human mitochondrial DNA. Nat Genet 23, 147 (1999).

149. Weissensteiner, H. et al. HaploGrep 2: mitochondrial haplogroup classification in the era of high-throughput sequencing. Nucleic Acids Res 44, W58–W63 (2016).

150. Vianello, D. et al. HAPLOFIND: A new method for high-throughput mtDNA haplogroup assignment. Hum Mutat 34, 1189–1194 (2013).

151. Durinck, S., Spellman, P. T., Birney, E. & Huber, W. Mapping identifiers for the integration of genomic datasets with the R/Bioconductor package biomaRt. Nat Protoc 4, 1184–1191 (2009).

152. Szolek, A. et al. OptiType: Precision HLA typing from next-generation sequencing data. Bioinformatics 30, 3310–3316 (2014).

153. Cao, K. et al. Analysis of the frequencies of HLA-A, B, and C alleles and haplotypes in the five major ethnic groups of the United States reveals high levels of diversity in these loci and contrasting distribution patterns in these populations. Hum Immunol 62, 1009–1030 (2001).

154. Yunis, E. J. et al. Single Nucleotide Polymorphism Blocks and Haplotypes: Human MHC Block Diversity. Encyclopedia of Molecular Cell Biology and Molecular Medicine vol. 13 192–215 (2006).

155. R Core Team. R: A language and environment for statistical computing. Preprint at https://www.r-project.org/ (2021).

156. Benjamini, Y., Kenigsberg, E., Reiner, A. & Yekutieli, D. FDR adjustments of microarray experiments (FDR-AME). MathTauAcIl 1, 1–3 (2005).

157. Penman, B. S. & Gupta, S. Detecting signatures of past pathogen selection on human HLA loci: are there needles in the haystack? Parasitology 145, 731–739 (2018).

158. Wang, P. et al. Peptide binding predictions for HLA DR, DP and DQ molecules. BMC Bioinformatics 11, 568 (2010).

159. Moutaftsi, M. et al. A consensus epitope prediction approach identifies the breadth of murine T(CD8+)-cell responses to vaccinia virus. Nat Biotechnol 24, 817–819 (2006).

160. Di, D., Simon Thomas, J., Currat, M., Nunes, J. M. & Sanchez-Mazas, A. Challenging Ancient DNA Results About Putative HLA Protection or Susceptibility to Yersinia pestis. Mol Biol Evol 39, msac073 (2022).

161. Bryc, K. et al. Genome-wide patterns of population structure and admixture among Hispanic/Latino populations. Proc Natl Acad Sci U S A 107, 8954–8961 (2010).

162. Alexander, D. H., Novembre, J. & Lange, K. Fast model-based estimation of ancestry in unrelated individuals. Genome Res 19, 1655–1664 (2009).

163. Schiffels, S. Xerxes CLI software. github.io https://github.com/poseidon-framework/poseidon-framework.github.io/blob/master/xerxes.md (2022).

164. Nägele, K. et al. Genomic insights into the early peopling of the Caribbean. Science (1979) 369, 456–460 (2020).

165. Nakatsuka, N. et al. A paleogenomic reconstruction of the deep population history of the Andes. Cell 181, 1131-1145.e21 (2020).

166. Scheib, C. L. et al. Ancient human parallel lineages within North America contributed to a coastal expansion. Science (1979) 360, 1024–1027 (2018).

167. Fernandes, D. M. et al. A genetic history of the pre-contact Caribbean. Nature 590, 103–110 (2021).

168. John, L. et al. The genetic prehistory of the Andean highlands 7000 years BP though European contact. Sci Adv 4, eaau4921 (2018).

169. Capodiferro, M. R. et al. Archaeogenomic distinctiveness of the Isthmo-Colombian area. Cell 184, 1706-1723.e24 (2021).

170. Moreno-Mayar, J. V. et al. Terminal Pleistocene Alaskan genome reveals first founding population of Native Americans. Nature 553, 203–207 (2018).

171. Moreno-Mayar, J. V. et al. Early human dispersals within the Americas. Science (1979) 362, eaav2621 (2018).

172. Rasmussen, M. et al. The ancestry and affiliations of Kennewick Man. Nature 523, 455–458 (2015).

173. Schroeder, H. et al. Origins and genetic legacies of the Caribbean Taino. Proceedings of the National Academy of Sciences 115, 201716839 (2018).

Disponibilidad de datos

Todos los datos genómicos (incluidas las secuencias de alineación de ADN nuclear, ADNmt y HLA) de los antiguos individuos de Chichén Itzá (YCH) están archivados en la base de datos del Archivo Europeo de Nucleótidos (ENA) (número de acceso PRJEB73567). Los datos genómicos actuales de los individuos de Tixcacaltuyub (TIX) estarán disponibles previa solicitud a R.B., J.C.L.R. y J.K., y sujeto a un acuerdo firmado para restringir el uso a estudios anónimos de la historia de la población [base de datos del Archivo Europeo Genoma-Fenóma (EGA); número de acceso: EGAD50000000426].

Todos los datos de HLA de nuestros conjuntos de muestras, tanto frecuencias como genotipos individuales, se pueden encontrar en el sitio web The Allele Frequency Net Database[75] (www.allelefrequencies.net) con los números de acceso: 3791 (YCH; https://www.allelefrequencies.net/pop6001c.asp?pop_id=3791) y 3790 (TIX; https://www.allelefrequencies.net/pop6001c.asp?pop_id=3790).

Agradecimientos

Los autores desean agradecer a Claudia Tzec Puch, Komathi Sree Ponnandai Shanmugavel, Martha Pimienta, Alfonso Gallardo, Evelyn Collen, Franziska Aron, Raphaela Stahl, Cäcilia Freund, Rita Radzeviciute, Marta Burri, Antje Wissgott, Sabrina Bodling, Elena Essel, Stephan Schiffels, Guido Brandt y Stephen Clayton por su apoyo técnico y analítico. Agradecemos a Lourdes Márquez Morfín, Erika González Zapata, John Walden, Anthi Tiliakou, Laura Curiel, Aida Andrades Valtueña, James Fellows Yates, Thiseas C. Lamnidis, Vanessa Villalba, Cosimo Posth y Julio Granados por los comentarios y discusiones que forman parte de la versión terminada de este manuscrito. Queremos agradecer a Michelle O'Reilly por su apoyo en el diseño gráfico de nuestras figuras. Gracias a todos los participantes que amablemente aceptaron beneficiar nuestra investigación con su muestra y tiempo, ¡Jach nib óolal! Este trabajo fue apoyado financieramente por la Sociedad Max Planck. A.S. recibió financiación de la Fundación Alemana de Investigación (Subvención ID We-4195/18-1 “ImMiGeNe”) a través de la colaboración con Alexander Weber, Departamento de Inmunología, Tübingen, Alemania. A.B.R. recibió el apoyo de la Sociedad Max Planck y el Consejo Europeo de Investigación (ERC) en el marco de la subvención 771234-PALEoRIDER del Programa de Investigación e Innovación Horizonte 2020 de la Unión Europea.

Contribuciones de autor

R.B., O.C.C., K.N., D.I.H.Z., C.W. y J.K. concibieron el proyecto. R.B., O.C.C. y D.I.H.Z. realizaron el registro y grabación fotográfica para YCH. O.C.C. realizó los análisis osteológicos para YCH. R.B., O.C.C. y D.I.H.Z. realizaron el trabajo de campo de muestreo de YCH. J.C.L.R., M.E.M.M., J.C.T.R. y C.I.T.P. realizaron el muestreo y extracción de ADN para TIX. R.B. y R.A.B. realizó el trabajo de laboratorio de ADN antiguo y la preparación de la biblioteca para TIX. R.B., O.C.C., R.A.B., P.P.R., P.R. y C.W. analizaron y discutieron los datos arqueológicos, incluidas las fechas de radiocarbono. P.P.R., M.L., A.B.R., P.R. y C.W. realizaron y discutieron los análisis de isótopos estables. R.B., K.N., A.B.R. y P.L. realizaron las pruebas de continuidad genética. R.B., D.I.H.Z., B.S.P., A.S., A.B.R., A.C. y O.K. realizó la selección y los análisis HLA. B.S.P. contribuyó al análisis de no superposición de HLA. R.B., O.C.C.,

K.N., D.I.H.Z., A.B.R., R.A.B., V.A.A., C.W. y J.K. analizaron los datos de genética de poblaciones y los resultados de marcadores uniparentales. Todos los autores discutieron los resultados. R.B., O.C.C., K.N., D.I.H.Z., P.P.R., C.W. y J.K. escribieron el artículo con contribuciones de todos los autores. Todos los autores leyeron y aprobaron la versión final. R.B., O.C.C., K.N., D.I.H.Z. y V.A.A. escribieron y presentaron el informe final (en español) para el Consejo de Arqueología (INAH) basado en el presente manuscrito, y tradujeron el artículo al español (disponible como archivo complementario).

Hay información complementaria disponible para este artículo. Los autores pusieron a disposición una traducción al español (no revisada por pares) de este artículo.
